# A chromosome-scale genome assembly of the pioneer plant *Stylosanthes angustifolia*: insights into genome evolution and drought adaptation

**DOI:** 10.1093/gigascience/giae118

**Published:** 2025-01-24

**Authors:** Chun Liu, Jianyu Zhang, Ranran Xu, Jinhui Lv, Zhu Qiao, Mingzhou Bai, Shancen Zhao, Lijuan Luo, Guodao Liu, Pandao Liu

**Affiliations:** School of Tropical Agriculture and Forestry & Sanya Institute Breeding and Multiplication, Hainan University, Haikou/Sanya 570228/572025, China; Tropical Crops Genetic Resources Institute, Chinese Academy of Tropical Agricultural Sciences (CATAS), Haikou 571101, China; Key Laboratory of Crop Gene Resources and Germplasm Enhancement in Southern China, Ministry of Agriculture and Rual Affairs, Haikou 571101, China; Key Laboratory of Tropical Crops Germplasm Resources Genetic Improvement and Innovation of Hainan Province, Haikou 571101, China; School of Tropical Agriculture and Forestry & Sanya Institute Breeding and Multiplication, Hainan University, Haikou/Sanya 570228/572025, China; Tropical Crops Genetic Resources Institute, Chinese Academy of Tropical Agricultural Sciences (CATAS), Haikou 571101, China; Key Laboratory of Crop Gene Resources and Germplasm Enhancement in Southern China, Ministry of Agriculture and Rual Affairs, Haikou 571101, China; Key Laboratory of Tropical Crops Germplasm Resources Genetic Improvement and Innovation of Hainan Province, Haikou 571101, China; School of Tropical Agriculture and Forestry & Sanya Institute Breeding and Multiplication, Hainan University, Haikou/Sanya 570228/572025, China; Tropical Crops Genetic Resources Institute, Chinese Academy of Tropical Agricultural Sciences (CATAS), Haikou 571101, China; Key Laboratory of Crop Gene Resources and Germplasm Enhancement in Southern China, Ministry of Agriculture and Rual Affairs, Haikou 571101, China; Key Laboratory of Tropical Crops Germplasm Resources Genetic Improvement and Innovation of Hainan Province, Haikou 571101, China; School of Tropical Agriculture and Forestry & Sanya Institute Breeding and Multiplication, Hainan University, Haikou/Sanya 570228/572025, China; Tropical Crops Genetic Resources Institute, Chinese Academy of Tropical Agricultural Sciences (CATAS), Haikou 571101, China; Key Laboratory of Crop Gene Resources and Germplasm Enhancement in Southern China, Ministry of Agriculture and Rual Affairs, Haikou 571101, China; Key Laboratory of Tropical Crops Germplasm Resources Genetic Improvement and Innovation of Hainan Province, Haikou 571101, China; Guangxi Key Laboratory of Medicinal Resources Protection and Genetic Improvement/Guangxi Engineering Research Center of TCM Resource Intelligent Creation, Guangxi Botanical Garden of Medicinal Plants, Nanning 530023, China; Department of Biotechnology and Biomedicine, Technical University of Denmark, Kongens Lyngby 2800, Denmark; Beijing Life Science Academy, Beijing 102200, China; School of Tropical Agriculture and Forestry & Sanya Institute Breeding and Multiplication, Hainan University, Haikou/Sanya 570228/572025, China; Tropical Crops Genetic Resources Institute, Chinese Academy of Tropical Agricultural Sciences (CATAS), Haikou 571101, China; Tropical Crops Genetic Resources Institute, Chinese Academy of Tropical Agricultural Sciences (CATAS), Haikou 571101, China; Key Laboratory of Crop Gene Resources and Germplasm Enhancement in Southern China, Ministry of Agriculture and Rual Affairs, Haikou 571101, China; Key Laboratory of Tropical Crops Germplasm Resources Genetic Improvement and Innovation of Hainan Province, Haikou 571101, China

**Keywords:** *Stylosanthes angustifolia*, pioneer plant, *de novo* assembly, multiomics, drought tolerance

## Abstract

**Background:**

Drought is a major limiting factor for plant survival and crop productivity. *Stylosanthes angustifolia*, a pioneer plant, exhibits remarkable drought tolerance, yet the molecular mechanisms driving its drought resistance remain largely unexplored.

**Results:**

We present a chromosome-scale reference genome of *S. angustifolia*, which provides insights into its genome evolution and drought tolerance mechanisms. The assembled genome is 645.88 Mb in size, containing 319.98 Mb of repetitive sequences and 36,857 protein-coding genes. The high quality of this genome assembly is demonstrated by the presence of 99.26% BUSCO and a 19.49 long terminal repeat assembly index. Evolutionary analyses revealed that *S. angustifolia* shares a whole-genome duplication (WGD) event with other legumes but lacks recent WGD. Additionally, *S. angustifolia* has undergone gene expansion through tandem duplication approximately 12.31 million years ago. Through integrative multiomics analyses, we identified 4 gene families—namely, *xanthoxin dehydrogenase, 2-hydroxyisoflavanone dehydratase, patatin-related phospholipase A*, and *stachyose synthetase*—that underwent tandem duplication and were significantly upregulated under drought stress. These gene families contribute to the biosynthesis of abscisic acid, genistein, daidzein, jasmonic acid, and stachyose, thereby enhancing drought tolerance.

**Conclusions:**

The genome assembly of *S. angustifolia* represents a significant advancement in understanding the genetic mechanisms underlying drought tolerance in this pioneer plant species. This genomic resource provides critical insights into the evolution of drought resistance and offers valuable genetic information for breeding programs aimed at improving drought resistance in crops.

## Introduction

Drought represents one of the most significant environmental challenges, drastically impairing plant survival and considerably reducing annual crop yields [1, 2]. Plants have developed a range of physiological, biochemical, and morphological mechanisms to respond to drought stress. These include stomatal closure to minimize transpiration, alterations in root architecture to optimize water uptake, and increased biosynthesis of compounds such as abscisic acid (ABA), osmoprotectants, flavonoids, and isoflavonoids [1, 3–6]. Additionally, the accumulation of nonreducing sugars, such as raffinose, plays a crucial role in drought adaptation [7, 8]. Understanding the genes and molecular pathways that underpin these adaptive responses is essential for advancing the development of drought-tolerant crop cultivars [9–11]. Therefore, comprehensive research on the genetic mechanisms that enable plants to withstand drought stress is urgently needed.

The genus *Stylosanthes* (family Leguminosae) comprises approximately 50 species. These include diploid (2n = 2x = 20), tetraploid (2n = 4x = 40), and hexaploid (2n = 6x = 60) species, distributed across tropical and subtropical regions [12, 13]. This genus is a pioneer plant in acid soils [14], exhibiting superior adaptability to frequent abiotic stresses such as low phosphorus availability [15, 16], aluminum toxicity [17, 18], manganese toxicity [19, 20], and low pH [21]. Among the species of the *Stylosanthes* genus, *Stylosanthes guianensis* (2n = 2x = 20) stands out as the most widely domesticated and utilized species, serving as both forage and green manure [22]. Numerous cultivars of *S. guianensis* have been developed across different countries: the cultivars “Bandeirante,” “Mineirão,” and “IRI 1022” in Brazil; the cultivars “Schofield,” “Endeavour,” and “Cook” in Australia; and the cultivars “Reyan No. 2,” “Reyan No. 5,” and “Stylo 907” in China [14, 23, 24]. The significance of utilizing wild relatives in genomic and genetic research is immense, as they offer invaluable genetic diversity and traits that are crucial for enhancing cultivars [25–27]. *Stylosanthes angustifolia*, a diploid species (2n = 2x = 20), is a wild relative of *S. guianensis* [28]. We observed that *S. angustifolia* exhibits good adaptability in the dry-hot valley regions of southwestern China, which frequently experience seasonal drought. However, the potential mechanisms underlying its drought tolerance remain unclear. This study aims to elucidate the molecular basis of drought-tolerant traits in *S. angustifolia* through genomic sequencing, thereby providing genetic resources for breeding drought-adapted *Stylosanthes* cultivars.

Gene duplication, including tandem duplication, plays a pivotal role in generating genetic diversity and driving plant evolution and adaptation [29–31]. Recent researches have highlighted the role of tandem duplicated genes (TDGs) in environmental adaptation, as demonstrated in species like pigeonpea (*Cajanus cajan*) [32, 33], grapevine (*Vitis vinifera*) [34], and silver birch (*Betula pendula*) [35]. Lineage-specific TDGs are particularly important for the adaptive evolution of plants in rapidly changing environments [36]. Whole-genome duplication (WGD) and tandem duplication have also been linked to the expansion of salinity adaptation genes in the halophyte *Tamarix chinensis* [37]. Genome-wide identification of TDGs, combined with multiomics analyses, is essential for understanding how these genes contribute to plant evolution and environmental adaptation [33, 37–39].

In this study, we present a high-quality, *de novo* assembly of the leguminous pioneer plant *S. angustifolia* (NCBI:txid79067), utilizing Oxford Nanopore Technologies (ONT), next-generation sequencing (NGS), and high-through chromosome conformation capture (Hi-C) technologies. Comparative genomics reveals the evolutionary position and divergence of *S. angustifolia*. Integrating comparative genomics, transcriptomics, and metabolomics, we demonstrate the critical role of TDGs in *S. angustifolia* genome evolution and its adaptation to drought stress.

## Results

### Sequencing and assembly of the *S. angustifolia* genome

**Figure 1:**
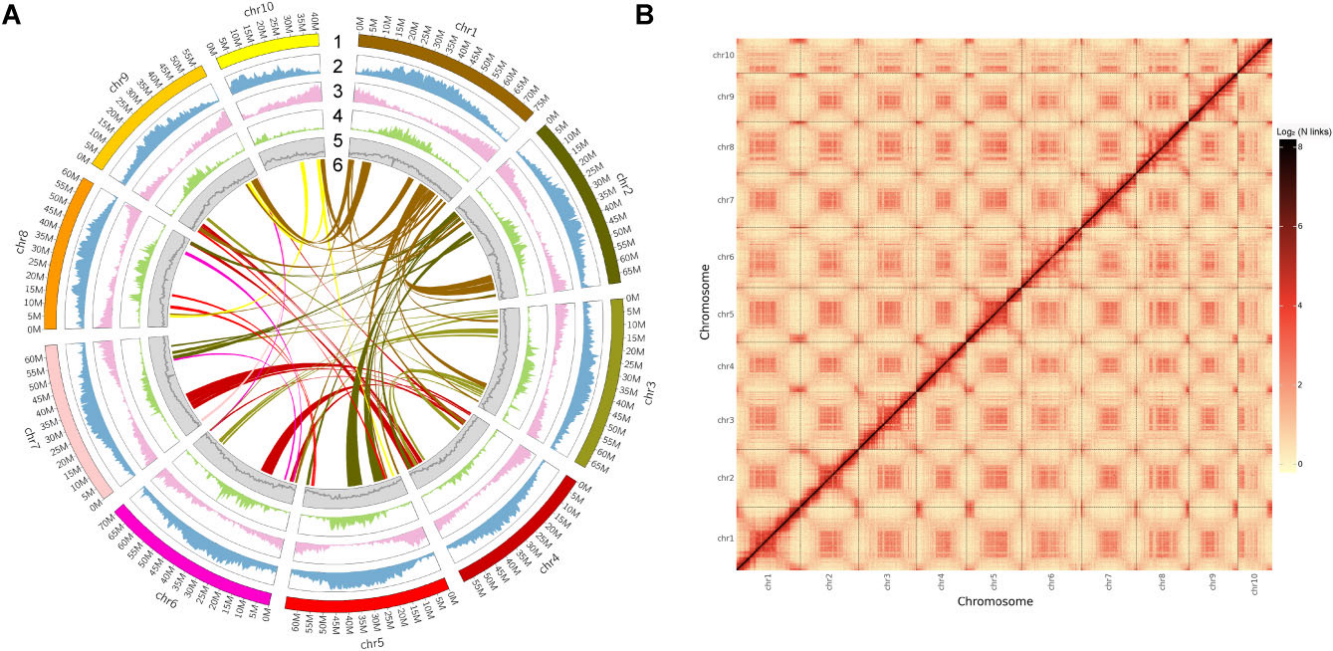
Genomic features of *S. angustifolia*. (A) Features of assembled *S. angustifolia* genome. From 1 to 6: chromosomes, repeat element density, gene density, noncoding RNA density, GC content, and intraspecific collinearity between chromosomes. The contents of 2 to 5 were calculated using a nonoverlapping window size of 500 Kb. (b) Hi-C interactions among 10 chromosomes of the *S. angustifolia* genome. Dark red indicates strong interactions and yellow indicates weak interactions.

In this study, we employed NGS and ONT technologies for the whole-genome sequencing of *S. angustifolia* (Germplasm number: TF0003 and Supplementary Fig. S1). We generated a total of 35.08 Gb of NGS data (∼53.02× coverage) and 104.72 Gb of ONT data (∼158.29× coverage) (Supplementary Tables S1 and S2). Based on *k*-mer analysis of the NGS data, we estimated the genome size of *S. angustifolia* to be 661.55 Mb (Supplementary Fig. S2). We used the software NextDenovo to correct and assemble the raw ONT data, resulting in an initial assembly of 167 contigs with a total length of 645.87 Mb and an N50 of 14.98 Mb. Subsequently, we employed NextPolish to polish the initial contigs using both ONT and NGS data to produce high-quality contigs. To further achieve chromosomal-level assembly, we used Hi-C technology, generating 53.89 Gb of clean data (∼81.47× coverage) (Supplementary Table S3). By analyzing the Hi-C data with Juicer and 3-dimensional *de novo* assembly (3D-DNA) software, we were able to align and organize the high-quality contigs into chromosomes. This achieved a chromosomal-level assembly of the *S. angustifolia* genome, spanning 631.54 Mb (97.78% of the assembled genome) across 10 chromosomes and containing 96 gaps. The final assembled genome had a total length of 645.88 Mb, a GC content of 35.52%, and a contig N50 of 14.99 Mb (Fig. 1 and Table 1). Single nucleotide polymorphism (SNP) analysis using a genome analysis toolkit (GATK) identified 379,931 heterozygous SNPs, resulting in a heterozygosity rate of 0.06%. We evaluated the completeness of the assembled genome using BUSCO, which yielded a completeness score of 99.26%, and the long terminal repeat (LTR) assembly index (LAI), which provided a score of 19.49 (Table 1 and Supplementary Table S4). Additionally, *k*-mer completeness analysis showed a score of 96.37% for the *S. angustifolia* genome (Table 1). The mapping rates of the NGS and ONT reads to the assembled genome were 99.15% and 99.69%, respectively, with average depths of 54.16× for NGS reads and 156.20× for ONT reads. In total, 95.16% of the genome was covered by NGS and 99.96% by ONT reads.

**Table 1:** Statistics of genomic features of *S. angustifolia*

Terms	*S. angustifolia*
Estimated genome size (Mb)	661.55
Assembled genome size (Mb)	645.88
Contig N50 (Mb)	14.99
GC content (%)	35.52
BUSCO (%)	99.26
LTR assembly index	19.49
*K*-mer completeness (%)	96.37
Number of chromosomes	10
Repeat content (Mb)	319.98
Repeat ratio (%)	49.54
Number of protein-coding genes	36,857
Mean exon length (bp)	242.95
Mean intron length (bp)	465.57

### Genome annotation of the *S. angustifolia* genome

We applied both *de novo* and homology-based approaches to annotate the repetitive sequence in the *S. angustifolia* genome. This analysis identified a total of 319.98 Mb of repetitive sequences, which constitutes 50.70% of the *S. angustifolia* genome (Table 1). Among these sequences, LTRs were the most abundant, comprising 42.91% of the genome, followed by DNA transposons (accounting for 2.49% of the genome) and long interspersed nuclear elements (LINEs) (representing 1.64% of the genome) (Table 1, Supplementary Tables S5 and S6).

To facilitate the prediction of protein-coding genes, transcriptome sequencing was performed on the *S. angustifolia* roots, stems, leaves, flowers, and seeds, generating 40.40 Gb of clean data (Supplementary Table S7). Transcriptome assembly was performed using both reference-based and *de novo* approaches, and the transcripts from both methods were incorporated into the prediction of protein-coding genes. Using a combination of *ab initio* prediction, homology, and transcriptomic evidence, we identified 36,857 protein-coding genes in the *S. angustifolia* genome, with an average exon length of 242.95 base pairs (bp) and an average intron length of 465.57 bp (Table 1). The completeness of the gene set was assessed using BUSCO, revealing a completeness score of 97.71% (92.50% single-copy BUSCOs and 5.20% duplicated BUSCOs) (Supplementary Table S8). Furthermore, functional annotation indicated that 98.94% of the predicted genes were annotated, with 68.80% and 65.07% being annotated in the Kyoto Encyclopedia of Genes and Genomes (KEGG) and Gene Ontology (GO) databases, respectively (Supplementary Table S9). Additionally, we identified noncoding RNAs (ncRNAs) within the *S. angustifolia* genome, including 97 microRNAs (miRNAs), 3,048 small nuclear RNAs (snRNAs), and 573 transfer RNAs (tRNAs) (Supplementary Table S10).

### Comparative genomic analysis among leguminous plants and Arabidopsis

**Figure 2:**
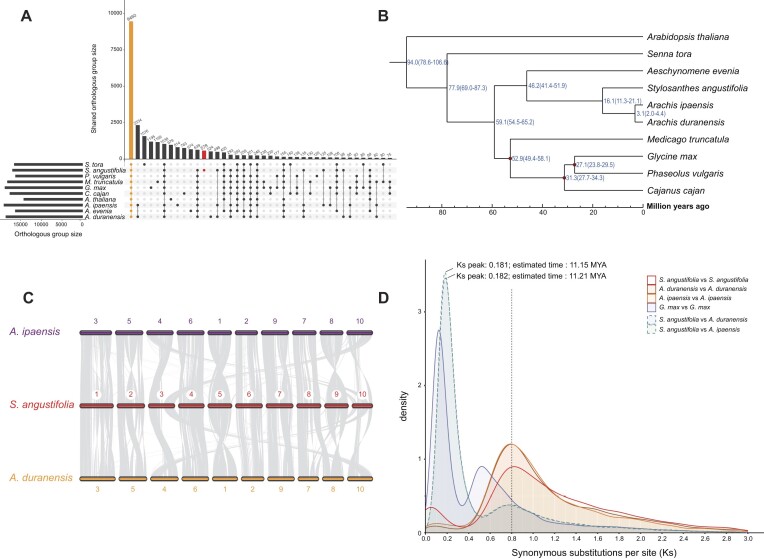
Comparative genomic analyses of *S. angustifolia* and other plant species. (A) Orthologous groups (OGs) and shared OGs of studied Fabaceae species and Arabidopsis. The red circle represents *S. angustifolia*–specific OGs. (B) Phylogenetic trees and divergence time analysis of the studied species based on single-copy OGs. (C) Genomic synteny comparisons between *S. angustifolia, A. duranensis*, and *A. ipaensis*. (D) Ks distribution of collinear gene pairs within and between *S. angustifolia, A. duranensis, A. ipaensis*, and *G. max*.

To investigate the evolutionary relationships of *S. angustifolia*, we conducted gene family and phylogenetic analyses across 9 leguminous species and the model plant Arabidopsis. Gene clustering analysis identified a total of 31,810 orthologous groups (OGs) across the studied species. Of these, 9,460 OGs were found to be shared by all species. Additionally, 633 OGs were identified as single-copy across all species, while 578 OGs were found to be specific to *S. angustifolia* (Fig. 2A).

The phylogenetic tree based on the single-copy OGs showed that *S. angustifolia* is closely related to the wild peanut relatives, *Arachis duranensis* and *Arachis ipaensis*, with these species forming sister clades (Fig. 2B). This relationship further supports the evolutionary position of *S. angustifolia* within the subtribe *Stylosanthinae* (Benth.) of the legume family. To further explore the dynamics of gene families, we performed gene family expansion and contraction analyses on 6 well-annotated and extensively studied legume species. The analysis identified 2,089 gene families that have expanded in *S. angustifolia*, of which 158 OGs showed statistically significant expansions (*P* < 0.05) (Supplementary Fig. S3). These 158 OGs comprise 3,071 genes, primarily associated with KEGG pathways involved in “carbohydrate metabolism,” “biosynthesis of other secondary metabolites,” and “lipid metabolism” (Supplementary Fig. S4).

We also identified collinear gene blocks within and between *S. angustifolia*, soybean, *A. duranensis*, and *A. ipaensis*. The genomes of *S. angustifolia, A. duranensis*, and *A. ipaensis* exhibit extensive genomic rearrangements, particularly on chromosomes 3, 9, and 10. However, some chromosomal regions maintained strong synteny, such as chromosomes 1, 6, and 8 (Fig. 2C). Analysis of the synonymous substitution rate (Ks) distribution among collinear gene pairs revealed that *S. angustifolia* shared the ancestral Papilionoideae whole-genome duplication event (PWGD) with soybean, *A. duranensis*, and *A. ipaensis* (Fig. 2D). Similar Ks peaks observed in *S. angustifolia, A. duranensis*, and *A. ipaensis* suggest that this duplication event occurred approximately 49.26 million years ago (MYA) (Fig. 2D). Furthermore, the divergence time between *S. angustifolia* and the 2 *Arachis* species were estimated to be 11 MYA, consistent with divergence time derived from single-copy gene family analyses (Fig. 2B, D).

### Transcriptome analysis of *S. angustifolia* in response to drought stress

**Figure 3:**
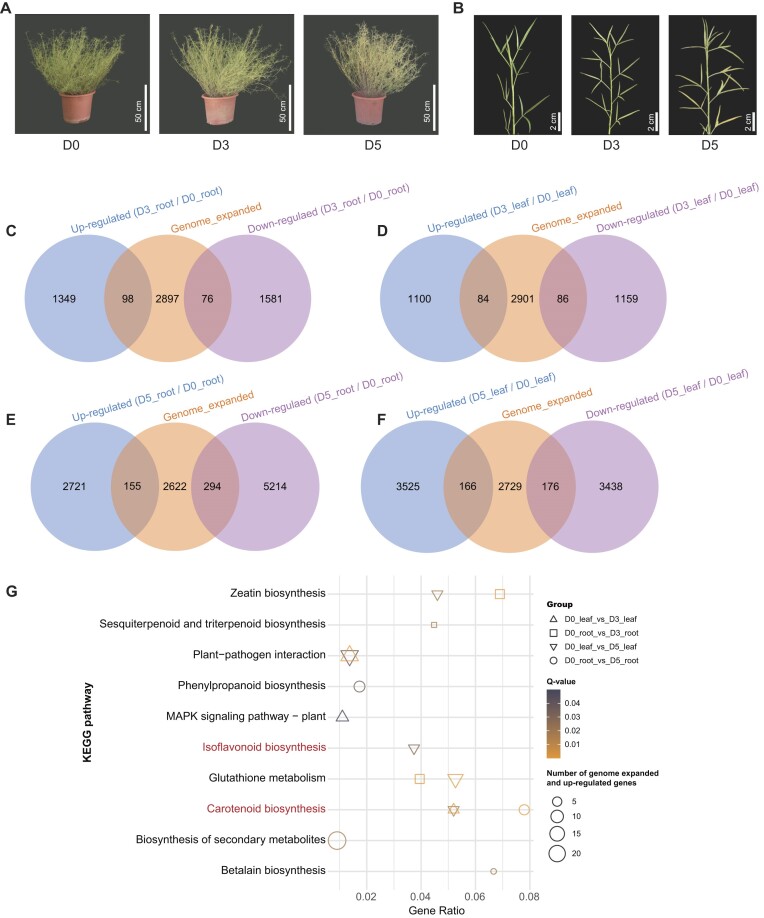
Transcriptome analysis and genome evolution of *S. angustifolia* in adaptation to drought stress. Plant phenotypes (A) and leaf characteristics (B) of *S. angustifolia* after 0 days (D0), 3 days (D3), and 5 days (D5) of drought treatment. Intersection analysis of genome-expanded genes and differentially expressed genes (DEGs) in roots (C) and leaves (D) of *S. angustifolia* at D3 compared to D0. Intersection analysis of genome-expanded genes and DEGs in roots (E) and leaves (F) of *S. angustifolia* at D5 compared to D0. (G) KEGG pathway enrichment analysis of genome-expanded genes that are upregulated by drought stress in roots and leaves of *S. angustifolia* (*Q* < 0.05).

To assess the response of *S. angustifolia* to drought stress, 60-day-old seedlings were subjected to drought treatments for 0 days (D0, control), 3 days (D3), and 5 days (D5) under pot conditions. As the duration of drought treatment increased, the leaves of *S. angustifolia* progressively turned yellow (Fig. 3A, B), accompanied by a gradual decrease in chlorophyll a and b content (Supplementary Fig. S5A, B), as well as in shoot water content (Supplementary Fig. S5C). Additionally, soil water content decreased by 62.16% at D3 and by 81.91% at D5 relative to D0 (Supplementary Fig. S5D). To further understand the gene expression dynamics under drought stress, transcriptome sequencing was conducted on root and leaf samples collected at D0, D3, and D5 (Fig. 3A). This sequencing yielded a total of 130.05 Gb clean data (average 7.22 Gb per sample) with Q30 greater than 93.54% (Supplementary Table S11). Gene expression analysis revealed that 29,215 genes were expressed throughout the drought treatment period. Furthermore, differential gene expression analysis showed that a greater number of genes were differentially expressed in roots and leaves at D5 than at D3 (Supplementary Fig. S6). Among these differentially expressed genes (DEGs), 384 genes were upregulated in both roots and leaves at D3, while 1,246 genes were upregulated in roots and leaves at D5 (Supplementary Fig. S7).

**Figure 4:**
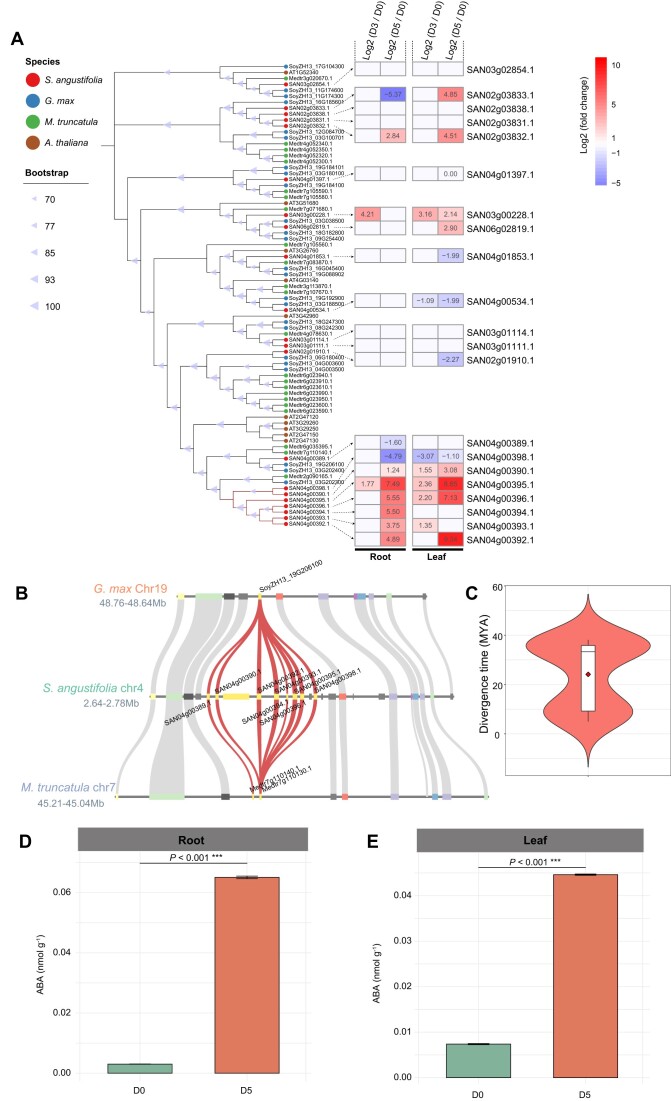
Evolution and expansion of *xanthoxin dehydrogenase* (*ABA2*) genes in the *S. angustifolia* genome and their response to drought stress. (A) Phylogenetic tree of *ABA2* genes in *S. angustifolia, G. max, M. truncatula*, and *A. thaliana*. The heatmap illustrates the changes in the expression of the *ABA2* genes after drought treatment for 3 days (D3) or 5 days (D5), compared to the control (0 days, D0). Differentially expressed genes (DEGs) are defined as those with a |log_2_ fold change| > 1 and *P*adj < 0.05. (B) Microcollinearity of *ABA2* genes in *S. angustifolia* compared with *G. max* and *M. truncatula*. Red curves represent the correspondence of *ABA2* genes across different species. (C) The divergence time of *ABA2* genes occurred by tandem duplication. The quantification of ABA contents in roots (D) and leaves (E) of *S. angustifolia*. Asterisks indicate significant differences between D5 and D0, as determined by Student’s *t*-test: ***P < 0.001.

An intersection analysis of the expanded gene families and the DEGs revealed 98 upregulated genes in roots and 84 in leaves at D3 (Fig. 3C, D). At D5, 155 genes were upregulated in roots and 166 in leaves (Fig. 3E, F). Enrichment analysis of these upregulated and expanded genes revealed significant enrichment in the “carotenoid biosynthesis pathway” (map00906, *Q* < 0.05) in both roots and leaves (Fig. 3G, Supplementary Tables S12–S15). Further investigation highlighted the critical role of *xanthoxin dehydrogenase* (*ABA2*, K09841), a key gene family involved in ABA biosynthesis. This gene family exhibited significant upregulated in both roots and leaves under drought stress (Supplementary Tables S13–S15). Genome-wide identification of *ABA2* genes in *S. angustifolia*, soybean, barrel medic, and Arabidopsis indicated a higher number of *ABA2* genes in the studied leguminous plants compared to Arabidopsis (Supplementary Table S16). Notably, the *ABA2* gene underwent tandem duplication, resulting in the expansion of 7 *ABA2* genes on chromosome 4 in *S. angustifolia* (Fig. 4A, B). Divergence time analysis indicated these duplications occurred approximately 24.13 MYA, with the most recent duplication around 5.01 MYA (Supplementary Table S17). Among the 7 tandem-duplicated *ABA2* genes, 6 were upregulated in roots and 4 in leaves at D5 (Fig. 4A). Quantification of ABA content revealed a 20.43-fold increase in roots and a 5.05-fold increase in leaves at D5 compared to D0, suggesting that *ABA2* gene expansion and upregulation contributed to ABA biosynthesis under drought stress (Fig. 4D, E).

**Figure 5:**
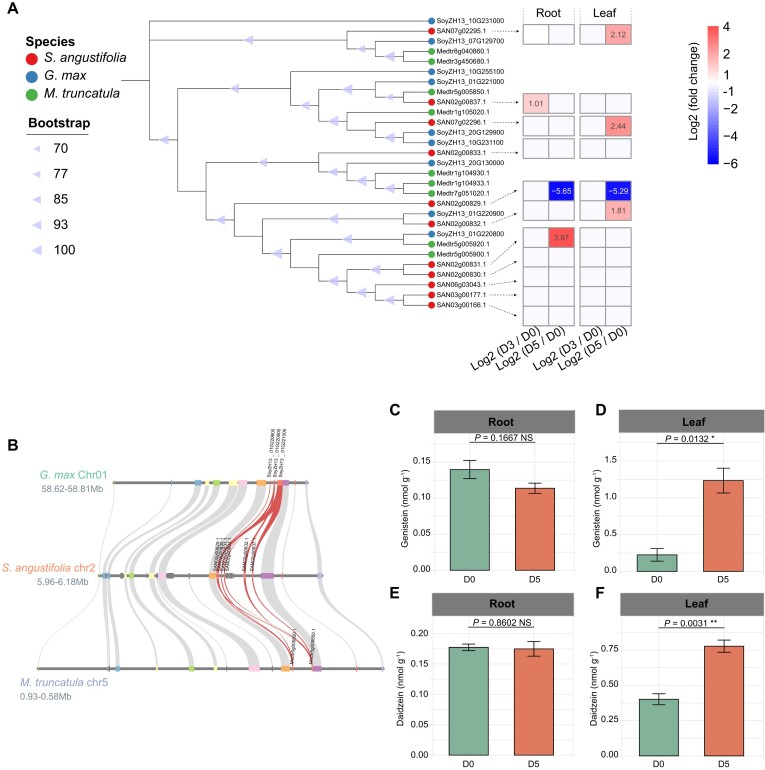
Evolution and expansion of *2-hydroxyisoflavanone dehydratase* (*HIDH*) genes in the *S. angustifolia* genome and their response to drought stress. (A) Phylogenetic tree of *HIDH* genes in *S. angustifolia, G. max*, and *M. truncatula*. The heatmap illustrates the changes in the expression of the *HIDH* genes after drought treatment for 3 days (D3) or 5 days (D5), compared to the control (0 days, D0). Differentially expressed genes (DEGs) are defined as those with a |log_2_ fold change| > 1 and *P*adj < 0.05. (B) Microcollinearity of *HIDH* genes in *S. angustifolia* compared with *G. max* and *M. truncatula*. Red curves represent the correspondence of *HIDH* genes across different species. The quantification of genistein contents in roots (C) and leaves (D) of *S. angustifolia*. The quantification of daidzein contents in roots (E) and leaves (F) of *S. angustifolia*. Asterisks indicate significant differences between D5 and D0, as determined by Student’s *t*-test: * 0.01 ≤ *P* < 0.05, **P < 0.01. NS, not significant.

In addition, genes that were expanded and upregulated in leaves at D5 showed significant enrichment in the “isoflavonoid biosynthesis pathway” (map00943) (*Q* < 0.05) (Fig. 3G). Further analysis identified three *2-hydroxyisoflavanone dehydratase* (*HIDH*, K13258) genes involved in genistein and daidzein biosynthesis (Supplementary Table S15). Phylogenetic and microsynteny analyses indicated a specific expansion of *HIDH* genes on chromosome 2 through tandem duplication (Fig. 5A, B, Supplementary Table S18), with the most recent duplication occurring at 11.11 MYA (Supplementary Table S19). Three *HIDH* genes were upregulated in leaves at D5 (Fig. 5A). Consistent with the transcriptome findings, genistein and daidzein contents in leaves at D5 increased by 448% and 94% at D5, respectively, compared to D0 (Fig. 5D, F). However, genistein and daidzein contents in roots showed no significant differences between D0 and D5 (Fig. 5C, E).

### Contribution of TDGs to drought tolerance of *S. angustifolia*

**Figure 6:**
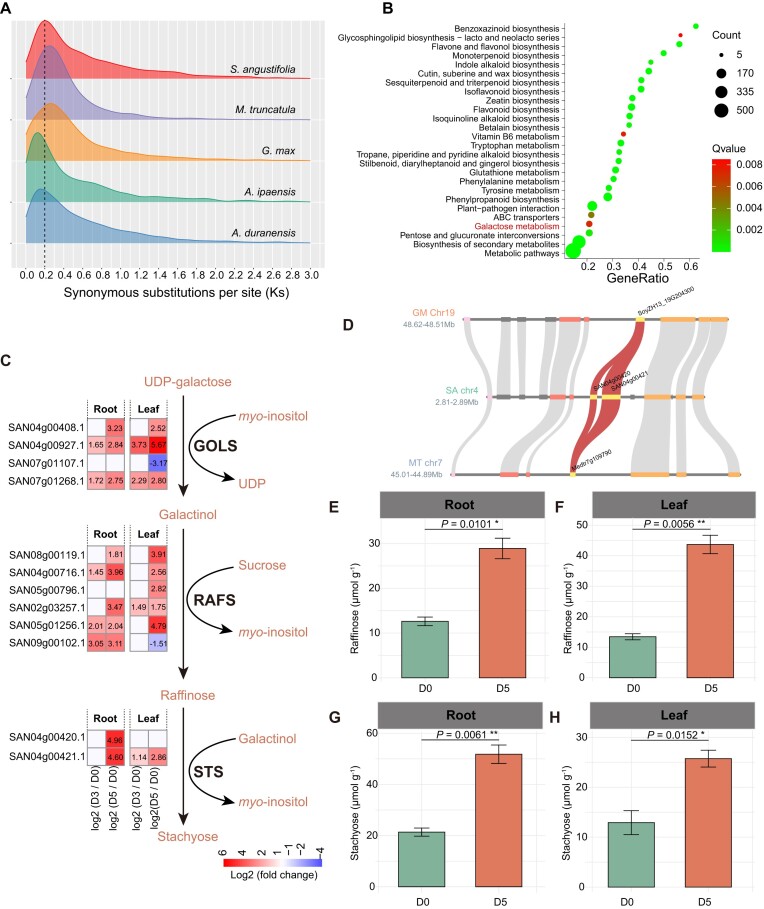
Analysis of tandem duplicated genes (TDGs) and the biosynthesis pathway of raffinose and stachyose in *S. angustifolia* under drought stress. (A) Ks distribution of TDGs in *S. angustifolia, A. duranensis, A. ipaensis, G. max*, and *M. truncatula*. (B) KEGG pathway enrichment analysis of TDGs in *S. angustifolia*. The KEGG pathways depicted in the figure are associated with a *Q* < 0.05. (C) Gene expression changes in the raffinose and stachyose biosynthesis pathway after 3 days (D3) and 5 days (D5) of drought treatment compared to the control (0 days, D0). Differentially expressed genes (DEGs) are defined as those with a |log_2_ fold change| > 1 and *P*adj < 0.05. (D) Microcollinearity of *STS* genes in *S. angustifolia* compared with *G. max* and *M. truncatula*. Red curves represent the correspondence of *STS* genes across different species. The quantification of raffinose contents in roots (E) and leaves (F) of *S. angustifolia*. The quantification of stachyose contents in roots (G) and leaves (H) of *S. angustifolia*. Asterisks indicate significant differences between D5 and D0, as determined by Student’s *t*-test: * 0.01 ≤ *P* < 0.05, **P < 0.01.

The *S. angustifolia* genome shows no evidence of recent WGDs (Fig. 2D), and gene family analysis indicated that tandem duplication plays an important role in gene expansion within the *S. angustifolia* genome (Figs 3–5). Therefore, we performed a genome-wide identification of TDGs and investigated their response to drought stress in *S. angustifolia*. We identified 3,634 TDGs in the *S. angustifolia* genome, which is more than in *A. duranensis* (2,735) and *A. ipaensis* (3,449) but fewer than in soybean (5,022) and barrel medic (7,032). Analysis of the Ks distribution of TDGs revealed a substantial expansion in *S. angustifolia* approximately 12.31 MYA, with a Ks peak at about 0.2 (Fig. 6A).

TDGs in *S. angustifolia* were significantly enriched in KEGG pathways such as “biosynthesis of secondary metabolites,” “flavonoid biosynthesis,” “isoflavonoid biosynthesis,” and “galactose metabolism” (*Q* < 0.05) (Fig. 6B). Transcriptomic analysis revealed that 3 gene families involved in raffinose and stachyose biosynthesis in the galactose metabolism pathway—inositol 3-alpha-galactosyltransferases (*GOLSs*), raffinose synthases (*RAFSs*), and stachyose synthetases (*STSs*)—exhibited significantly increased expression after 3 and 5 days of drought treatment. The most pronounced upregulation was observed at D5 (Fig. 6C and Supplementary Table S20). Notably, the 2 *STS* genes, significantly upregulated in roots at D5, were expanded through tandem duplication (Fig. 6C, D).

Consistent with gene expression patterns, raffinose content increased by 129% in roots and 225% in leaves at D5 compared to D0 (Fig. 6E, F). Similarly, stachyose content increased by 142% in roots and 99% in leaves at D5 compared to D0 (Fig. 6G, H).

### Lipid metabolism in *S. angustifolia* in response to drought stress

**Figure 7:**
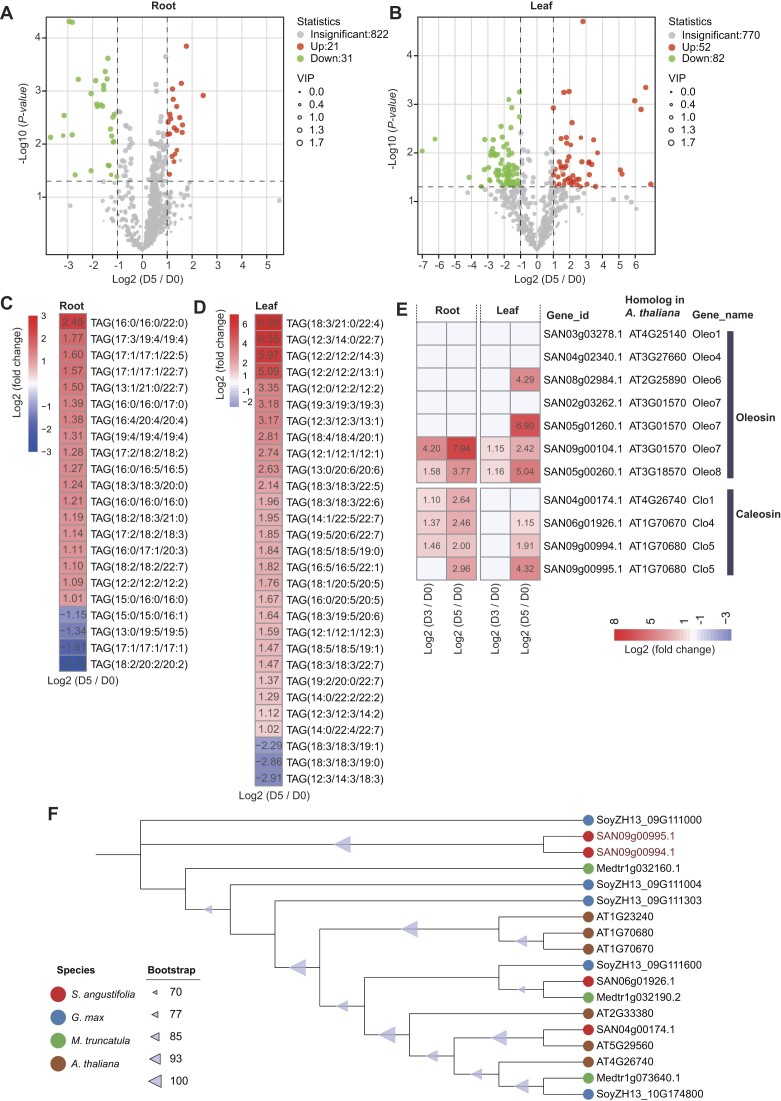
Lipidomics analysis of roots and leaves of *S. angustifolia* in response to drought stress. The volcano plots show lipid profiles in roots (A) and leaves (B) after 5 days (D5) of drought treatment compared to the control (0 days, D0). The heatmap illustrates the differentially accumulated triacylglycerols (TAGs) in roots (C) and leaves (D) at D5 compared to D0. (E) Changes in gene expression of the *oleosin* and *caleosin* families at D3 and D5 compared to D0. Differentially expressed genes (DEGs) are defined as those with a |log_2_ fold change| > 1 and *P*adj < 0.05. (F) Phylogenetic tree of caleosins in *S. angustifolia, G. max, M. truncatula*, and *A. thaliana*.

Comparative genomic analysis revealed that significantly expanded gene families in *S. angustifolia* are involved in the lipid metabolism pathway (Supplementary Fig. S4). To assess the effects of drought stress on lipid metabolism in *S. angustifolia*, we conducted a lipidomic analysis on samples from both roots and leaves collected on D0 and D5. A total of 874 lipids were identified in roots and 904 lipids in leaves, spanning 6 major lipid classes (Fig. 7A, B, Supplementary Tables S21 and S22). Among these, 52 lipids in roots and 134 lipids in leaves were identified as differentially accumulated lipids (DALs) at D5 compared to D0 (Fig. 7A, B). Notably, 22 triacylglycerols (TAGs) were detected in roots and 29 TAGs in leaves, with 81.81% of TAGs in roots and 89.66% in leaves showing upregulated at D5 (Fig. 7C, D). In plants, TAGs associate with membrane protein families, including oleosins, caleosins, and steroleosins, to form subcellular organelles known as oil bodies, which play a crucial role in regulating lipid metabolism and maintaining lipid homeostasis [40]. Following TAG accumulation at D5, 2 of the 7 oleosin family genes were upregulated in roots of *S. angustifolia*, while 4 were upregulated in leaves (Fig. 7E, Supplementary Table S23). Similarly, after 5 days of drought treatment, all 4 oleosin family genes in *S. angustifolia* were upregulated in roots, while 3 of the 4 were upregulated in leaves (Fig. 7E). Notably, 2 of these upregulated oleosin genes originated from tandem duplications (Fig. 7F).

**Figure 8:**
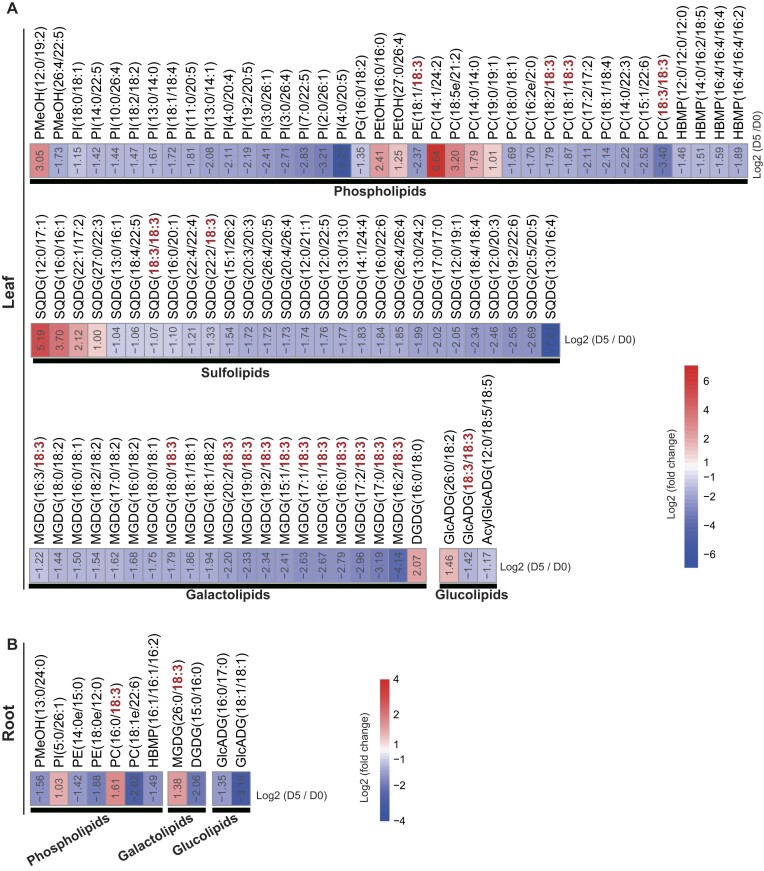
Differential accumulation of phospholipids, sulfolipids, galactolipids, and glucolipids in leaves (A) and roots (B) of *S. angustifolia* after 5 days (D5) of drought treatment compared to the control (0 days, D0).

**Figure 9:**
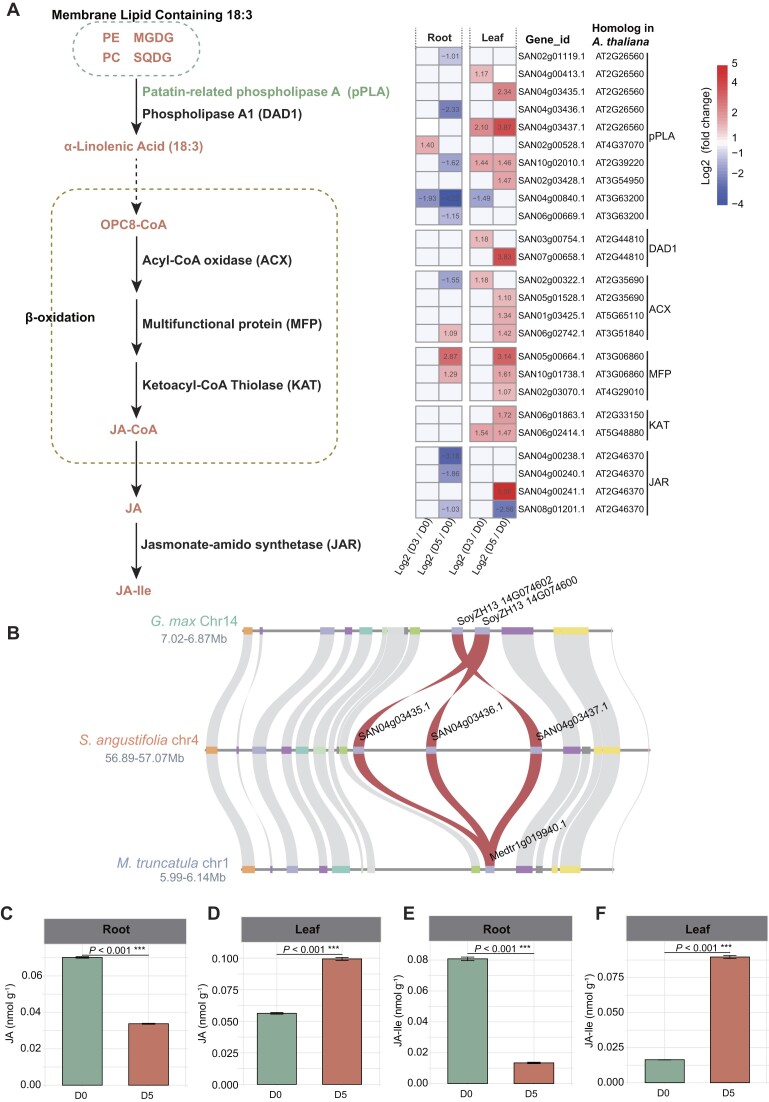
Biosynthesis of jasmonic acid (JA) and jasmonoyl-L-isoleucine (JA-Ile) in *S. angustifolia* in response to drought stress. (A) Gene expression changes in the JA biosynthesis pathway after 3 days (D3) and 5 days (D5) of drought treatment compared to the control (0 days, D0). Differentially expressed genes (DEGs) are defined as those with a |log_2_ fold change| > 1 and *P*adj < 0.05. (B) Microcollinearity of *pPLA* genes in *S. angustifolia* compared with *G. max* and *M. truncatula*. Red curves represent the correspondence of *pPLA* genes across different species. The quantification of JA contents in roots (C) and leaves (D) of *S. angustifolia*. The quantification of JA-Ile contents in roots (E) and leaves (F) of *S. angustifolia*. Asterisks indicate significant differences between D5 and D0, as determined by Student’s *t*-test: ***P < 0.001.

In plants, the degradation of membrane lipids containing C18:3 chains lead to the production of α-linolenic acid, a precursor in the biosynthesis of jasmonic acid (JA) [41]. Lipidomic analyses revealed a significant reduction in 4 phospholipids, 2 sulfolipids, 12 galactolipids, and 1 glucolipid containing C18:3 chains in leaves after 5 days of drought stress (Fig. 8A). In contrast, the roots exhibited a significant decrease in only 1 phospholipid and 1 galactolipid containing C18:3 chains after 5 days of drought stress (Fig. 8B). Consistent with these lipidomic changes, drought treatment for 5 days led to the upregulation of several gene families involved in the biosynthesis of JA and jasmonoyl-L-isoleucine (JA-Ile) in leaves (Fig. 9A, Supplementary Table S24). Specifically, 4 members of *patatin-related phospholipase A* (*pPLA*), 1 member of *phospholipase A1* (*DAD1*), 3 members of *acyl-CoA oxidase* (*ACX*), 3 members of *multifunctional protein* (*MFP*), 2 members of *ketoacyl-CoA thiolase* (*KAT*), and 1 member of *jasmonate-amido synthetase* (*JAR*) were upregulated (Fig. 9A). Interestingly, the *pPLA* gene underwent tandem duplication approximately 18.67 MYA (Ks = 0.30) (Fig. 9B). Quantitative measurements of JA and JA-Ile levels showed substantial increases in leaves, with JA levels increasing by 77% and JA-Ile levels increasing by 447% at D5 compared to D0 (Fig. 9D, F). In contrast, JA and JA-Ile levels were significantly reduced in roots at D5 compared to D0 (Fig. 9C, E).

## Discussion

Exploring the mechanisms by which pioneer plants adapt to harsh environmental conditions provides valuable insights for enhancing stress tolerance traits in crops [42, 43]. Despite the pioneer plant *S. angustifolia* being recognized for its exceptional drought tolerance, the absence of high-quality genomic resources has considerably impeded a comprehensive understanding of its molecular mechanisms underlying drought resistance. In this study, we assembled a high-quality genome of *S. angustifolia* by integrating NGS, ONT, and Hi-C sequencing technologies, yielding a genome size of 645.88 Mb. Our assembly demonstrates high integrity and accuracy, as confirmed by multiple quality assessment metrics (Table 1 and Fig. 1). Notably, the *S. angustifolia* genome shows low heterozygosity (0.06%). This high-quality genome resource will facilitate evolutionary, genetic, and functional studies, especially those aimed at advancing molecular breeding programs for drought tolerance.

Gene duplication is essential in plant evolution and adaptation to environmental challenges [29, 30]. In particular, tandem duplication is an important feature in the pathways of secondary metabolite biosynthesis and responses to biotic and abiotic stresses [44, 45]. For instance, the expansion of sugar metabolism-related genes, including *α-amylase* (*AMY3*) and *β-fructofuranosidase* (*CWINV1*), in *Sophora moorcroftiana* contributes to high sucrose content, promoting long root growth and enhancing drought tolerance [46]. In orchardgrass (*Dactylis glomerata*), tandem duplication of DgMADS-box genes has promoted longer root lengths and higher survival rates under various abiotic stresses [47]. Similarly, in Pearl millet (*Pennisetum glaucum*), expansion of the RWP-RK gene family enables rapid responses to heat stress by regulating the expression of endoplasmic reticulum (ER)–related gene expression [48]. These examples highlight the significance of TDGs in environmental adaptation.

In *S. angustifolia*, TDGs played a crucial role in its drought adaptation, as demonstrated by an integrative comparative genomics, transcriptomics, and metabolomics analysis. ABA, a key phytohormone involved in the drought stress response [49–51], exhibited significantly increased accumulation in leaves and roots of *S. angustifolia* after 5 days of drought treatment (Fig. 4D, E). The expansion of *ABA2* genes through tandem duplication in the *S. angustifolia* genome may enhance ABA biosynthesis under drought stress (Fig. 4A, B). Additionally, JA and its active conjugate, JA-Ile, are widely distributed plant hormones involved in resistance to biotic and abiotic stresses, including drought [6, 52–56]. Our findings indicate that genes encoding *pPLAs*, which degrade C18:3-branched membrane lipids, have expanded through tandem duplication, likely promoting JA and JA-Ile biosynthesis in *S. angustifolia* leaves under drought conditions (Fig. 9).

Raffinose family oligosaccharides (RFOs), including raffinose, stachyose, and verbascose, are *α*-galactosyl derivatives of sucrose that are common in plants and play important roles in regulating plant responses to abiotic stress [57]. In maize, drought conditions increase raffinose accumulation, and overexpression of *ZmRAFS*, a gene responsible for raffinose biosynthesis, enhances drought tolerance in transgenic plants [7, 8]. Similar results were observed in *S. angustifolia*, where *RAFS* family genes were upregulated in both leaves and roots under drought stress, leading to increased raffinose accumulation (Fig. 6C, E, F). Raffinose can be further synthesized into stachyose. We found that stachyose accumulation increased in *S. angustifolia* leaves and roots under drought conditions (Fig. 6G, H). Additionally, the *STS* genes responsible for stachyose biosynthesis expanded via tandem duplication and were upregulated under drought conditions (Fig. 6C, D), which may contribute to the drought tolerance mechanism characteristic of *S. angustifolia*. Apart from RFOs, isoflavonoids, such as genistein, have been shown to contribute to enhancing plant drought resistance [4, 5]. In *S. angustifolia, HIDH* genes, responsible for the biosynthesis of genistein and daidzein, underwent expansion through tandem duplication (Fig. 5B). After 5 days of drought treatment, genistein and daidzein levels in *S. angustifolia* leaves increased (Fig. 5D, F), likely due to the upregulation of the *HIDH* genes (Fig. 5A), suggesting their active role in drought tolerance.

In conclusion, the genome assembly of *S. angustifolia* provides new insights into the genetic basis of drought tolerance. Our study highlights the significant role of tandem duplication in the expansion of key gene families involved in phytohormone biosynthesis, secondary metabolism, and osmoprotection, which collectively

enhance the drought adaptation of *S. angustifolia*. These findings advance our understanding of the molecular mechanisms underlying drought tolerance and offer valuable genomic resources for breeding drought-tolerant crops.

## Methods

### Plant materials

*S. angustifolia* (Germplasm number: TF0003, Supplementary Fig. S1) was provided by the National Tropical Plants Germplasm Resource Center (Hainan, China). Young leaves were harvested for DNA extraction using the CTAB method for both NGS and ONT sequencing. Root, stem, leaf, flower, and seed samples were collected for RNA extraction and transcriptome sequencing.

### Genome sequencing and assembly

High-quality genomic DNA was extracted for the construction of ONT and NGS libraries. Sequencing was performed at the Genome Center of Grandomics (Wuhan, China). The ONT library was processed using the ONT PromethlON platform, while the NGS library was sequenced using the MGI-SEQ 2000 platform (MGI Tech). Hi-C technology was adopted for chromosome-level genome assembly. To achieve chromosome-level assembly, Hi-C technology was employed. Hi-C library construction and sequencing were also performed at the Genome Center of Grandomics (Wuhan, China) with DPN II as the restriction enzyme. The ONT reads were processed with guppy (v6.5.7). The quality control of raw NGS and Hi-C reads was performed using SOAPnuke (v2.1.7; RRID:SCR_015025) using parameters “-n 0.01 -l 20 -q 0.3 –polyX 50.” ONT reads were corrected and assembled into initial contigs using NextDenovo (v2.3.0; RRID:SCR_025033) [58], with “read_cutoff” set to 2k to ensure the high-quality assembly results. Additionally, both NGS and ONT reads were incorporated into further polishing of initial contigs using NextPolish (v1.4.1; RRID:SCR_025232) [59]. The clean Hi-C reads were aligned to the polished contigs using the Burrows–Wheeler Aligner (BWA, v0.7.17; RRID:SCR_010910), and then Hi-C contact maps were generated using Juicer (v1.6). The 3D-DNA (v180922; RRID:SCR_017227) pipeline was adopted for chromosomal grouping, sorting, and orientation. Visualization and manual curation of Hi-C maps were conducted using Juicebox (v1.11.08; RRID:SCR_021172) to ensure the accuracy of chromosome assembly. The completeness of the genome assembly was evaluated using BUSCO (v5.3.2; RRID:SCR_015008) based on embryophyta_odb10 database and LAI analysis using LTR_retriever (v2.9.0; RRID:SCR_017623) [60]. Additionally, merqury (v1.3; RRID:SCR_022964) was also adopted for assembly completeness evaluation using an efficient *k*-mer set. Reads were aligned to the genome using minimap2 (v2.17-r941; RRID:SCR_018550) for ONT and BWA (v0.7.17; RRID:SCR_010910) for NGS. SAMtools (v1.9; RRID:SCR_002105) and PanDepth (v2.19) were adopted for genome mapping ratios and coverage statistics. Based on the NGS mapping results, SNPs were detected using GATK (v4.1.2.0; RRID:SCR_001876), and the heterozygosity rate of *S. angustifolia* was calculated by dividing the number of heterozygous SNPs by the total effective genome bases, then multiplying by 100 to obtain a percentage.

### Genome annotation

We employed both *de novo* and homology-based approaches for the identification of repetitive elements. Extensive *de novo* TE Annotator (EDTA, v2.0.1; RRID:SCR_022063) [61] was adopted for *de novo* identification of transposable elements (TEs). Additionally, known repetitive elements from RepBase (v21.12; RRID:SCR_021169) were identified using RepeatMasker (v4.1.4; RRID:SCR_012954). Tandem Repeat Finder (TRF, v. 4.09.1; RRID:SCR_022193) was utilized to detect tandem repeats.

Protein-coding gene prediction was performed based on the repeat-masked genome by employing *de novo–*based prediction, RNA sequencing–based prediction, and homologue-based prediction. We employed GALBA (v1.0.8) [62] for automated training and prediction of protein-coding genes, utilizing AUGUSTUS (v3.5.0; RRID:SCR_008417) [63] and miniport (v0.13) [64] with default parameters. SNAP (v. 2013-02-16; RRID:SCR_007936) was also employed for *de novo* gene prediction. Transcriptome sequencing reads from roots, stems, leaves, flowers, and seeds were aligned to the *S. angustifolia* genome using HISAT2 (v2.2.1; RRID:SCR_015530), and transcript construction was conducted using StringTie (v2.2.1; RRID:SCR_016323) [65]. RNA *de novo* assembly was performed using Trinity (v2.15.1; RRID:SCR_013048). Coding regions of the predicted transcripts were identified using TransDecoder (v 5.7.0; RRID:SCR_017647). For homology‐based prediction, proteins from 5 species—namely, *Glycine max, Medicago truncatula, A. ipaensis, Senna tora*, and *Arabidopsis thaliana*—were aligned to the *S. angustifolia* genome using tBLASTn (v2.13.0; RRID:SCR_011822), and gene structure prediction was performed by miniport (v0.13). EVidenceModeler (v2.1.0; RRID:SCR_014659) [66] was adopted for the identification of nonredundance consensus genes from all available evidence. Furthermore, PASA (v2.5.3; RRID:SCR_014656) was adopted to refine gene structure and annotate untranslated regions (UTRs) based on transcriptome data. The predicted protein-coding genes were aligned to various known databases, including NCBI Non-Redundant Protein Sequence Database (NR), KEGG, Eukaryotic Orthologous Groups of Protein (KOG), Swiss-Prot, TrEMBL, and InterPro databases, for functional annotation. BLASTp (v2.13.0; RRID:SCR_001010) was employed for homology searches against NR, KEGG, KOG, Swiss-Prot, and TrEMBL using the parameters “-outfmt 6 -evalue 1e-10.” Blast2GO (v6.0; RRID:SCR_005828) was adopted for GO annotation based on the NR annotation. The best hit for each gene was retained for subsequent analysis. Additionally, ncRNAs were predicted by BLASTn (v2.13.0; RRID:SCR_001598) and INFERNAL (v1.0; RRID:SCR_011809) based on the Rfam database (v12.0; RRID:SCR_007891).

### Comparative genomic analysis

Nine legumes, including *S. angustifolia, A. ipaensis, A. duranensis, Aeschynomene evenia, G. max, M. truncatula, Phaseolus vulgaris, Cajanus cajan*, and *Senna tora*, along with model species *Arabidopsis thaliana*, were utilized for comparative analysis. Protein sequences from these species were compared through all-versus-all alignments using BLASTp (v2.2.23, e-value set to 1e-5; RRID:SCR_001010). OrthoFinder (v2.5.4; RRID:SCR_017118) was utilized to identify orthologous groups (OGs) and construct phylogenetic tree using parameters “-S diamond -M msa -A mafft.” Divergence times between *G. max, M. truncatula, C. cajan*, and *P. vulgaris* were queried on TimeTree (http://www.timetree.org/) as known divergence times. Based on single-copy OGs, the substitution rates were estimated using the MCMCTREE program within the PAML (v4.5; RRID:SCR_014932) software package, which was further used to calculate the divergence times between species. MCScanX (RRID:SCR_022067) [67] was employed for intra- and interspecies gene collinearity analysis. First, protein sequences were aligned using BLASTp (v2.2.23, e-value set to 1e-5; RRID:SCR_001010), and MCScanX (RRID:SCR_022067) [67] was employed to identify collinear regions. For intraspecies analysis, we employed the “duplicate_gene_classifier” from MCScanX to classify paralogous genes into categories such as single-copy genes, dispersed duplicated genes, proximal duplicated genes, TDGs, and whole-genome or segmental duplicated genes. Microcollinearity of TDGs in the *S. angustifolia* genome was compared with that of soybean and barrel medic using the MCscan (Python version) [68]. Additionally, the nonsynonymous (Ka) and synonymous (Ks) substitution rates of gene pairs within the collinear regions, as well as the Ka and Ks of TDGs, were calculated using PAML (v4.9e; RRID:SCR_014932) and PAL2NAL (v14) using the Nei–Gojobori (NG) method [69]. The R platform (v4.0.2; RRID:SCR_001905) was adopted for Ks distribution visualization and peak identification. The divergence time of gene pairs was calculated using the formula T = Ks/2r, where the neutral substitution rate r was selected as 8.12 × 10^−9^ in this study [70].

### Gene identification and phylogenetic analysis

Protein sequences from *S. angustifolia*, soybean, and barrel medic were aligned against The Arabidopsis Information Resource database. Genes with an identity greater than 35% and coverage exceeding 50% were retained for further analysis of metabolic pathways. For the phylogenetic analysis, protein sequences from studied gene families were aligned using MUSCLE (v5.2; RRID:SCR_011812), and a phylogenetic tree was conducted using FastTree version (v2.1.11; RRID:SCR_015501) with the approximately maximum likelihood method. The resulting phylogenetic trees were visualized using iTOL (v6; RRID:SCR_018174) [71].

### Drought treatment on *S. angustifolia*

After seed germination, *S. angustifolia* seeds were planted in pots (30 cm in height, 25 cm in diameter) filled with sandy soil. The drought treatment was initiated when the seedlings reached 60 days old by withholding watering. Leaf and root samples were collected at day 0 (D0) for the control group and at days 3 (D3) and 5 (D5) after the initiation of drought treatment.

Soil water content was determined using previously reported methods [72–74]. Briefly, the total soil in each pot was recorded to obtain the wet weight (Ww) after plant harvesting. The soil was then dried at 65°C for 72 hours to obtain the dry weight (Wd). The soil water content was calculated using the following formula: (Ww − Wd)/Ww × 100%. The soil water content at D0 was normalized to 100% of pot capacity, while the corresponding soil water contents at D3 and D5 were expressed as percentages of this normalized value. Shoot water content was measured following previously described methods [75]. In brief, the shoot was harvested and weighed to obtain the fresh weight (FW). After drying at 65°C until a constant weight was reached, the tissues were weighed again to determine the dry weight (DW). The water content, expressed as a percentage of fresh weight (% FW), was calculated using the following formula: (FW − DW)/FW × 100%. Chlorophyll content was measured according to previously published methods [76]. For RNA sequencing, metabolite quantification, and lipidomics analysis, 3 biological replicates of root and leaf samples were collected.

### Transcriptome sequencing and bioinformatics analysis

RNA was extracted from root and leaf samples collected at D0, D3, and D5 during the drought treatment for transcriptome sequencing. Each treatment group in this experiment consisted of 3 biological replicates, with each biological replicate containing 20 seedlings. Transcriptomes were sequenced on the MGI-SEQ 2000 platform (MGI Tech), and SOAPnuke (v2.1.7; RRID:SCR_015025) [77] was adopted for quality control of the raw sequencing reads. Clean reads were mapped onto the *S. angustifolia* genome using HISAT2 (v2.2.1; RRID:SCR_015530). The featureCounts (v2.0.6; RRID:SCR_012919) was employed for the calculation of gene read counts, and an in-house Perl script was adopted for the calculation of transcripts per million. DEGs were identified using DESeq2 (v3.19; RRID:SCR_015687), and the Benjamini–Hochberg method was adopted for false discovery rate calculation. Genes with a |log_2_ fold change| > 1 and an adjusted *P* value (*P*adj) < 0.05 were considered DEGs. KEGG pathway enrichment analysis of DEGs was performed by the phyper and *p.adjust* functions under the R platform (v4.0.2; RRID:SCR_001905), and KEGG pathways with a *Q* value <0.05 were considered significantly enriched pathways. Gene expression heatmaps were generated under the R platform (v4.0.2; RRID:SCR_001905).

### Lipidomics analysis of *S. angustifolia* under drought stress

Lipidomics analysis was conducted on root and leaf samples of *S. angustifolia* by Biotree Biomedical Technology Co., Ltd. Sample extraction was performed with slight modifications to a previously reported method [78]. Briefly, freeze-dried samples were extracted using the flowing extracting solution: MTBE:MeOH = 5:1 (v/v) containing an isotope-labeled internal standard. Subsequently, 100 μL of the extracted supernatant was transferred to the injection bottle for lipid metabolite detection.

The chromatographic separation of the target compounds was performed using a Phenomenex Kinetex C18 column (2.1 × 100 mm, 2.6 μm) on a Vanquish ultra-performance liquid chromatograph (Thermo Fisher Scientific). The mobile phase A consisted of 40% water and 60% acetonitrile with 10 mmol/L ammonium formate, while phase B comprised 10% acetonitrile and 90% isopropanol, supplemented with 50 mL of 10 mmol/L ammonium formate aqueous solution per liter. The injection volume was set at 2 μL. Mass spectrometric analysis was performed on an Orbitrap Exploris 120, allowing for both primary and secondary mass spectrometry data acquisition using Xcalibur (v4.4). The operational parameters were as follows: sheath gas flow rate at 30 Arb, auxiliary gas flow rate at 10 Arb, capillary temperature at 320°C (both positive and negative modes), full mass spectrometry (MS) resolution at 60,000, MS/MS resolution at 15,000, collision MS resolution at 15,000, collision energy at 15/30/45 in NCE mode, and spray voltage at 3.8 kV (positive) or −3.4 kV (negative).

The raw mass spectrum data were converted to mzXML format using ProteoWizard (RRID:SCR_012056) software. XCMS was then used for retention time correction, peak identification, extraction, integration, and alignment. The minimum fraction (minfrac) was set to 0.5, and the cutoff was set to 0.3. Lipid identification was performed through a spectral match using the LipidBlast library within the XCMS (RRID:SCR_015538) software [79].

MetaboAnalyst (v6.0; RRID:SCR_015539) [80] was adopted for lipids analysis. Lipids with a fold change of ≥2 or ≤0.5 in relative abundance between D5 and D0, along with variable importance for projection score >1 and an adjusted *P* value (*P*adj) < 0.05, were identified as DALs. Each experimental group had 3 biological replicates.

### Determination of ABA, JA, JA-Ile, genistein, daidzein, raffinose, and stachyose

Quantitative assays of plant hormones (ABA, JA, and JA-Ile) in roots and leaves of *S. angustifolia* were conducted by Biotree Biomedical Technology Co., Ltd. A total of 100 mg of the freeze-dried samples was weighed and extracted using 1 mL of ice-cold 50% acetonitrile (ACN) aqueous solution. The sample was sonicated at 4°C for 3 minutes, followed by extraction at 4°C for an additional 30 minutes. The mixture was centrifuged at 12,000 rpm for 10 minutes at 4°C, and the supernatant was collected. The sample was then passed through an RP-SPE column: 1 mL of 100% methanol (MeOH) and 1 mL of deionized water were added, and then the column was equilibrated with 50% ACN aqueous solution. The sample was loaded onto the column, which was washed with 1 mL of 30% ACN, and the eluent was collected. The sample was evaporated to dryness under a nitrogen stream, dissolved in 200 μL of 30% ACN, and transferred to a sample vial with an insert.

The data acquisition system primarily consisted of ultra-high-performance liquid chromatography (Vanquish; Thermo) coupled with a high-resolution mass spectrometer (Q Exactive; Thermo). The liquid chromatography parameters were set as follows: chromatographic column: Waters HSS T3 (50 × 2.1 mm, 1.8 μm); mobile phase: phase A was ultrapure water (containing 0.1% acetic acid), and phase B was acetonitrile (containing 0.1% acetic acid); flow rate: 0.3 mL/min; column temperature: 40°C; injection volume: 2 μL; and elution gradient: 0 minutes water/acetonitrile (90:10, v/v), 1 minute water/acetonitrile (90:10, v/v), 5 minutes water/acetonitrile (10:90, v/v), 7 minutes water/acetonitrile (10:90, v/v), 7.1 minutes water/acetonitrile (90:10, v/v), and 9 minutes water/acetonitrile (90:10, v/v). During the entire analysis, samples were kept in an autosampler at 4°C. To avoid signal fluctuation impacts, samples were analyzed in random sequence. Quality control samples were inserted into the sample queue to monitor and evaluate system stability and data reliability. Data acquisition was performed using the Q Exactive high-resolution mass spectrometer (Thermo Fisher Scientific). The electrospray ionization conditions were as follows: sheath gas, 40 arb; auxiliary gas, 10 arb; spray voltage, 3,000 V; temperature, 350°C; and ion transfer tube temperature, 320°C. The scan mode was set to single ion monitoring in positive ion mode, with a primary scan *m/z* range of 100–500. Mass spectrometry data were processed using TraceFinder software.

Quantitative assays of raffinose and stachyose in roots and leaves of *S. angustifolia* were conducted by Biotech-Pack-Analytical Inc. A total of 300 mg of freeze-dried samples was weighed and extracted twice with 5 mL of 80% ethanol at 85°C for 30 minutes each time. Following each extraction, the mixture was centrifuged at 12,000 rpm for 5 minutes. The combined supernatants were collected and evaporated to dryness using a vacuum centrifuge. The resulting dried residue was resuspended in 300 μL of distilled water and centrifuged again at 12,000 rpm for 10 minutes. The final supernatant was collected for high-performance liquid chromatography (HPLC) analysis. The liquid chromatography analysis was performed using a Waters 2695 HPLC coupled with a Waters 2424 evaporative light-scattering detector. The chromatographic conditions were as follows: column temperature, 40°C; flow rate, 1.0 mL/min; injection volume, 3 μL of sample; chromatographic column, Sepax HP-Amino (4.6 × 250 mm, 5 μm, 120 Å); mobile phase, acetonitrile/water (70:30) with isocratic elution; and total run time, 20 minutes.

Quantitative assays of genistein and daidzein in leaves and roots of *S. angustifolia* were conducted as described in a previous report [78].

## Additional Files

**Supplementary Fig. S1**. The field phenotype of *S. angustifolia*.

**Supplementary Fig. S2**. Genomic survey analysis of *S. angustifolia*.

**Supplementary Fig. S3**. Gene family expansion and contraction analyses of 7 studied plant species.

**Supplementary Fig. S4**. KEGG classification of *S. angustifolia* expanded orthologous groups (OGs).

**Supplementary Fig. S5**. Leaf chlorophyll a (A), leaf chlorophyll b (B), soil water content (C), and shoot water content (D) of *S. angustifolia* after drought treatment for 0 days (D0), 3 days (D3), and 5 days (D5) under pot conditions.

**Supplementary Fig. S6**. Volcano plot of differentially expressed genes (DEGs) in roots and leaves of *S. angustifolia* under drought stress.

**Supplementary Fig. S7**. (A) Intersection analysis of DEGs between roots and leaves at D3 compared to D0. (B) Intersection analysis of DEGs between roots and leaves at D5 compared to D0.

**Supplementary Table S1**. Statistics of Oxford Nanopore (ONT) sequencing data of *S. angustifolia*.

**Supplementary Table S2**. Statistics of next-generation sequencing (NGS) data of *S. angustifolia*.

**Supplementary Table S3**. Statistics of high-through chromosome conformation capture (Hi-C) sequencing data of *S. angustifolia*.

**Supplementary Table S4**. BUSCO assessment of assembled genome.

**Supplementary Table S5**. Statistics of repetitive sequence in the assembled genome.

**Supplementary Table S6**. Classification of repetitive sequence in the assembled genome.

**Supplementary Table S7**. Statistics of RNA-seq data from different tissues.

**Supplementary Table S8**. BUSCO assessment of predicted gene set.

**Supplementary Table S9**. Functional annotation of the predicted genes.

**Supplementary Table S10**. Statistics of noncoding RNAs in the assembled genome.

**Supplementary Table S11**. Statistics of RNA-seq data of *S. angustifolia* after drought treatment for 0 days (D0), 3 days (D3), and 5 days (D5).

**Supplementary Table S12**. KEGG pathway enrichment analysis of the *S. angustifolia* expanded genes that were upregulated in roots at D3 compared to D0.

**Supplementary Table S13**. KEGG pathway enrichment analysis of the *S. angustifolia* expanded genes that were upregulated in leaves at D3 compared to D0.

**Supplementary Table S14**. KEGG pathway enrichment analysis of the *S. angustifolia* expanded genes that were upregulated in roots at D5 compared to D0.

**Supplementary Table S15**. KEGG pathway enrichment analysis of the *S. angustifolia* expanded genes that were upregulated in leaves at D5 compared to D0.

**Supplementary Table S16**. Identification of *ABA2* (K09841) genes in *S. angustifolia*, soybean, barrel medic, and Arabidopsis based on the KEGG database.

**Supplementary Table S17**. Calculation of divergence time of the tandem duplicated *ABA2* genes in the *S. angustifolia* genome.

**Supplementary Table S18**. Identification of *HIDH* (K13258) genes in *S. angustifolia*, soybean, and barrel medic based on the KEGG database.

**Supplementary Table S19**. Calculation of divergence time of the tandem duplicated *HIDH* genes in the *S. angustifolia* genome.

**Supplementary Table S20**. The expression changes of genes involved in the biosynthesis of raffinose and stachyose in *S. angustifolia* at D3 or D5 compared to D0.

**Supplementary Table S21**. Lipid profiles in roots of *S. angustifolia* at D5 and D0.

**Supplementary Table S22**. Lipid profiles in leaves of *S. angustifolia* at D5 and D0.

**Supplementary Table S23**. Identification of oleosin and caleosin encoding genes in *S. angustifolia*, soybean, and barrel medic based on Arabidopsis database.

**Supplementary Table S24**. The expression changes of genes involved in the biosynthesis of JA and JA-Ile in *S. angustifolia* at D3 or D5 compared to D0.

giae118_Supplemental_Figures_and_Tables

giae118_GIGA-D-24-00294_Original_Submission

giae118_GIGA-D-24-00294_Revision_1

giae118_GIGA-D-24-00294_Revision_2

giae118_Response_to_Reviewer_Comments_Original_Submission

giae118_Response_to_Reviewer_Comments_Revision_1

giae118_Reviewer_1_Report_Original_Submission Feng Cheng -- 8/16/2024

giae118_Reviewer_1_Report_Revision_1 Feng Cheng -- 10/28/2024

giae118_Reviewer_1_Report_Revision_2 Feng Cheng -- 11/18/2024

giae118_Reviewer_2_Report_Original_Submission Fangyuan Zhang -- 8/21/2024

## Abbreviations

ABA: abscisic acid; ABA2: xanthoxin dehydrogenase; ACX: acyl-CoA oxidase; AMY3: α-amylase; Bp: base pair; BUSCO: Benchmarking Universal Single-Copy Orthologs; CWINV1: β-fructofuranosidase; DAD1: phospholipase A1; DAL: differentially accumulated lipid; DEG: differentially expressed gene; EDTA: extensive de novo TE annotator; GATK: genome analysis toolkit; Gb: gigabase; GO: Gene Ontology; GOLS: inositol 3-alpha-galactosyltransferases; Hi-C: high-through chromosome conformation capture; HIDH: 2-hydroxyisoflavanone dehydratase; JA: jasmonic acid; JA-Ile: jasmonoyl-L-isoleucine; JAR: jasmonate-amido synthetase; Ka: nonsynonymous; KAT: ketoacyl-CoA thiolase; KEGG: Kyoto Encyclopedia of Genes and Genomes; KOG: Eukaryotic Orthologous Groups of Protein; Ks: synonymous; LAI: long terminal repeat assembly index; LTR: long terminal repeat; Mb: megabase; MFP: multifunctional protein; MYA: million years ago; NGS: next-generation sequencing; NR: NCBI Non-Redundant Protein Sequence Database; OG: orthologous group; ONT: Oxford Nanopore Technologies; pPLA: patatin-related phospholipase A; RAFS: raffinose synthases; STS: stachyose synthetases; TAG: triacylglycerol; TDG: tandem duplicated gene; UTR: untranslated region; WGD: whole-genome duplication.

## Data Availability

The raw genomic sequencing data, including ONT, NGS, and Hi-C data, as well as transcriptome data, have been deposited in the National Genomics Data Center (NGDC) [81] under BioProject PRJCA027610. The raw sequence data have been deposited in the Genome Sequence Archive in the National Genomics Data Center, China National Center for Bioinformation/Beijing Institute of Genomics, Chinese Academy of Sciences (GSA: CRA017744) [82]. The assembly and annotation of *S. angustifolia* have been deposited in the Genome Warehouse in the National Genomics Data Center under accession number GWHFHGI00000000.1 [83]. The raw sequencing data can also be accessed through NCBI under the accession number PRJNA1140667. All supporting data and materials are available in the *GigaScience* database, GigaDB [84].
