# Supplementary figures and images for: A chromosome-scale genome assembly of the pioneer plant Stylosanthes angustifolia: insights into genome evolution and drought adaptation
Source: Gigascience. 2025 Jan 24;14:giae118. doi: 10.1093/gigascience/giae118 (PMC11758145; doi:10.1093/gigascience/giae118)

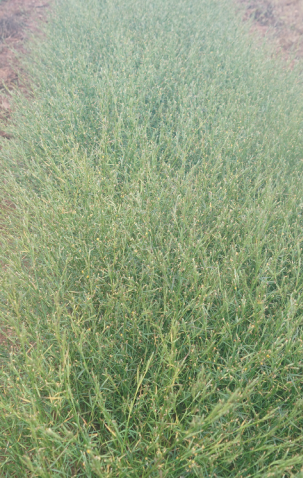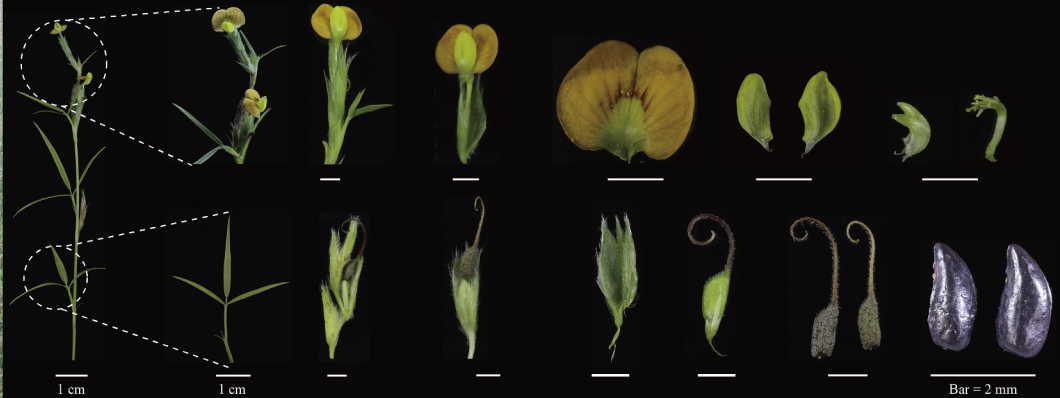

Supplement: giae118_Supplemental_Figures_and_Tables [file giae118_supplemental_figures_and_tables.zip › Figure S1.pdf]

# GenomeScope Profile

len:661,554,698bp uniq:39.5% het:0.342% kcov:21.8 err:0.24% dup:1.43% k:21

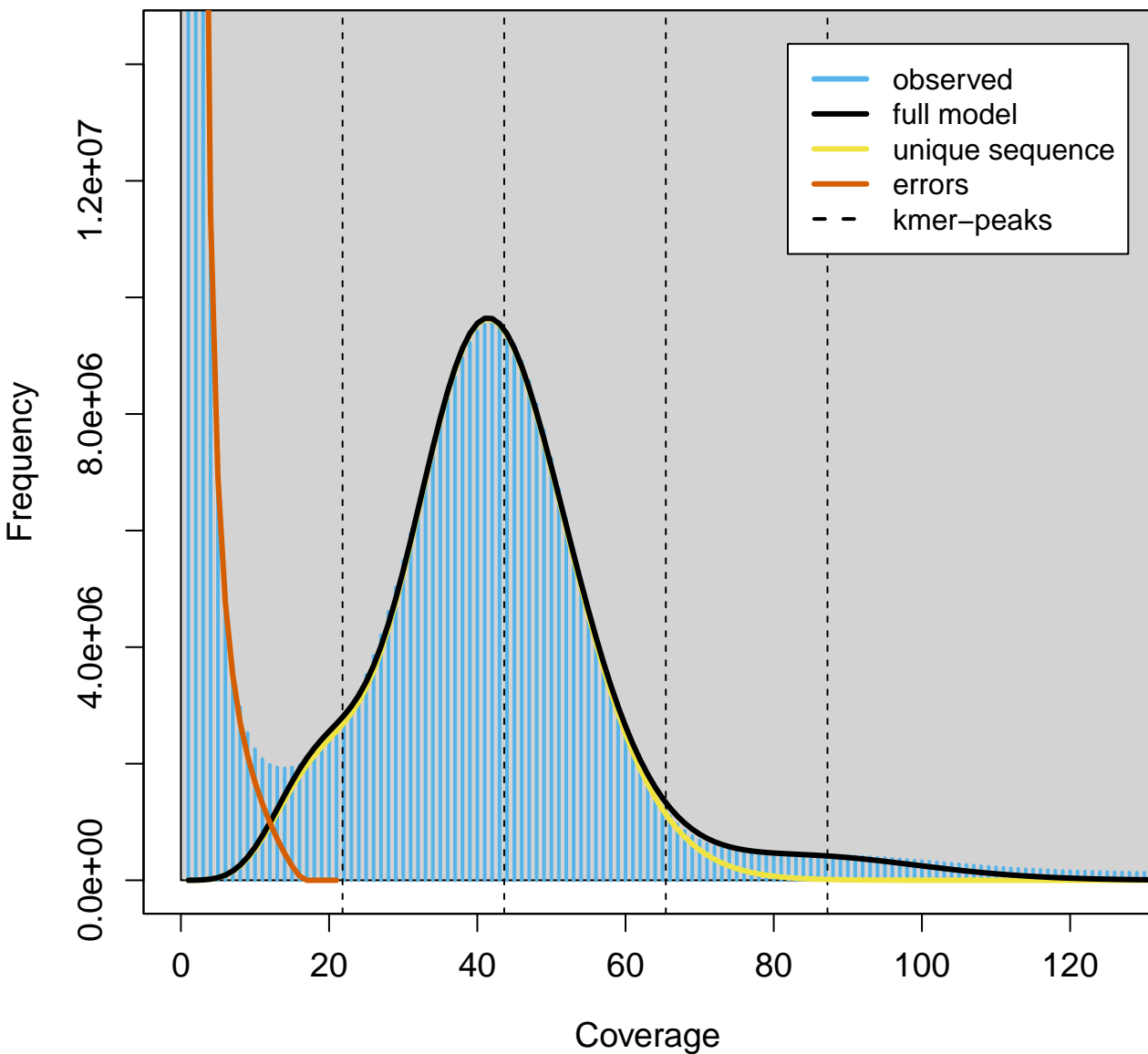

Supplement: giae118_Supplemental_Figures_and_Tables [file giae118_supplemental_figures_and_tables.zip › Figure S2.pdf]

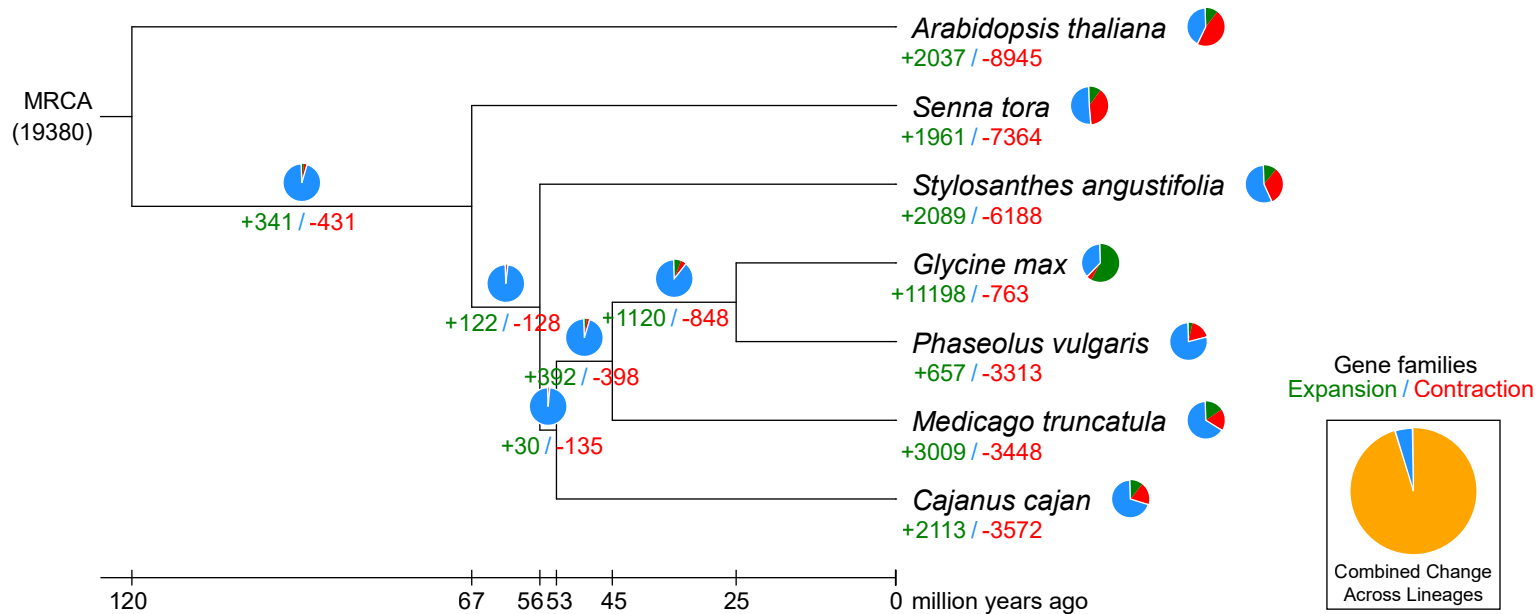

Supplement: giae118_Supplemental_Figures_and_Tables [file giae118_supplemental_figures_and_tables.zip › Figure S3.pdf]

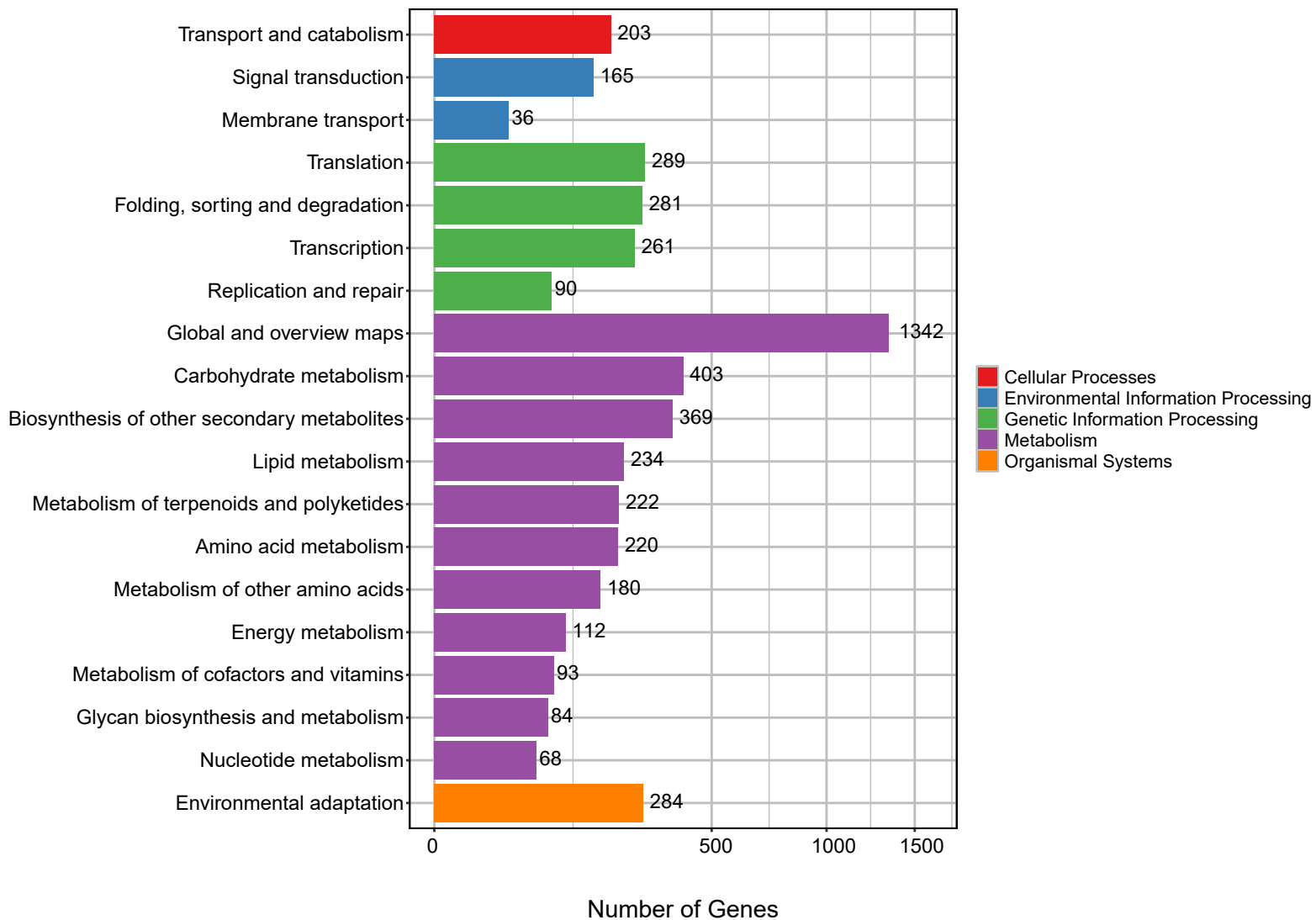

Supplement: giae118_Supplemental_Figures_and_Tables [file giae118_supplemental_figures_and_tables.zip › Figure S4.pdf]

**a**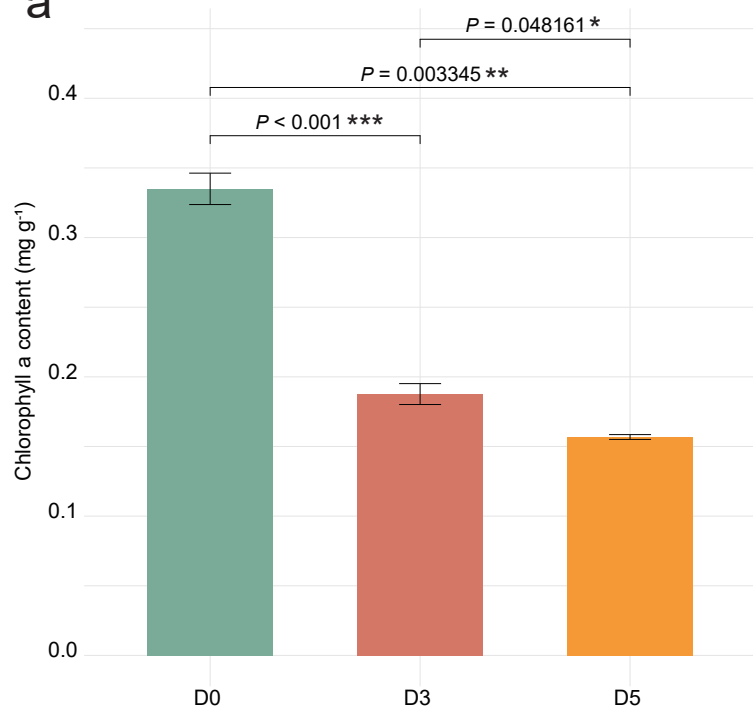**b**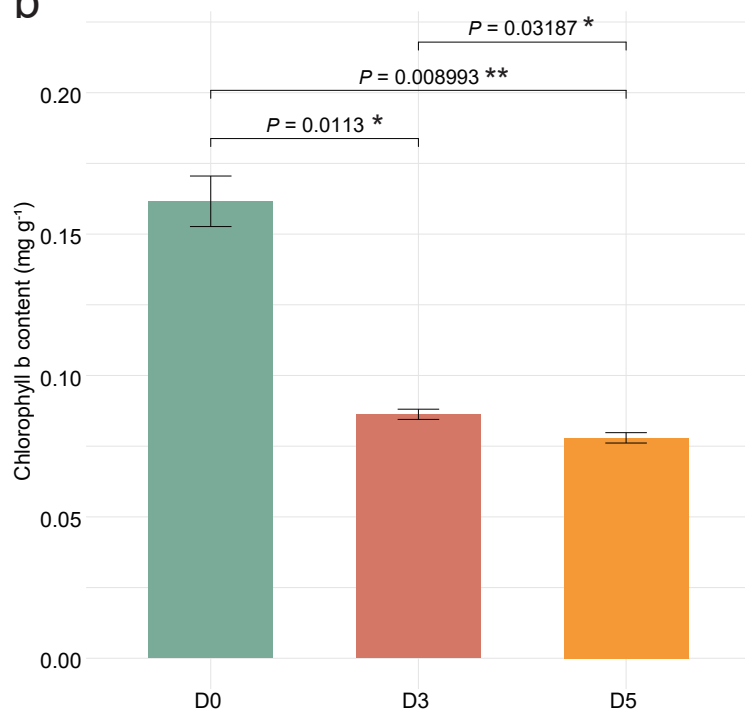**c**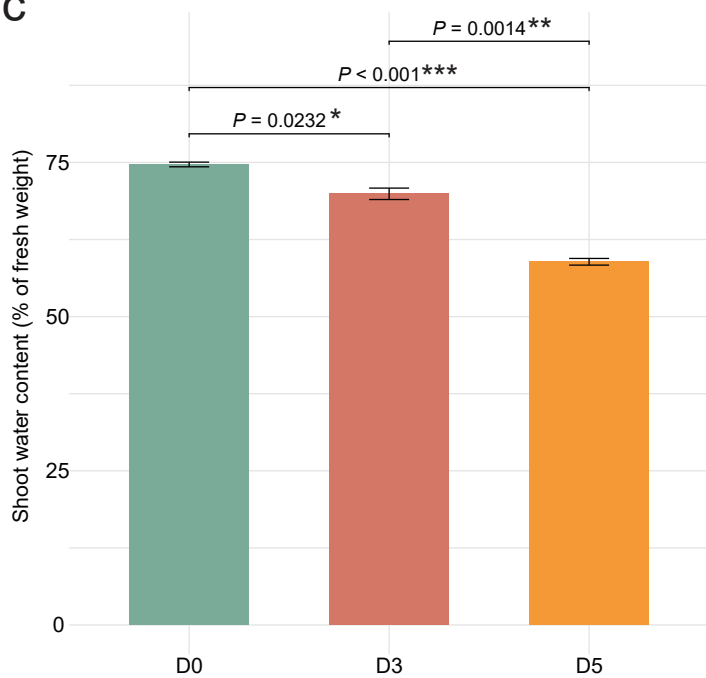**d**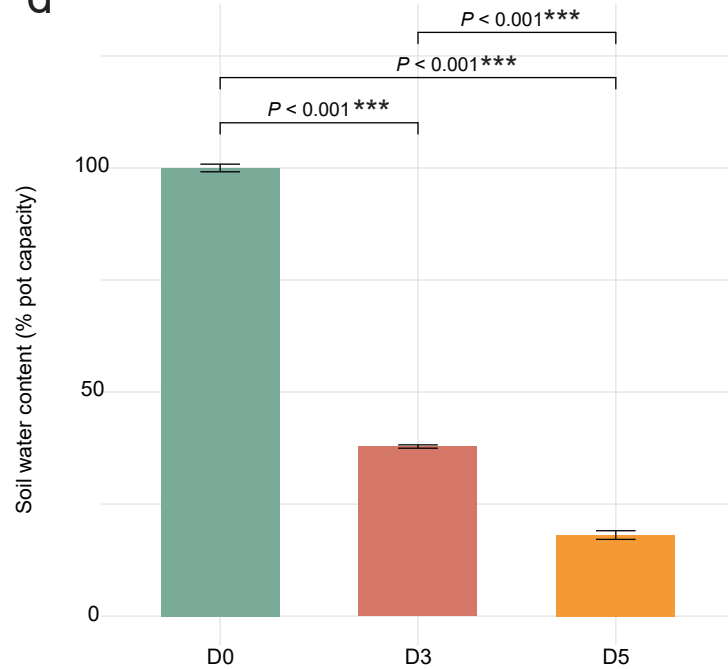

Supplement: giae118_Supplemental_Figures_and_Tables [file giae118_supplemental_figures_and_tables.zip › Figure S5.pdf]

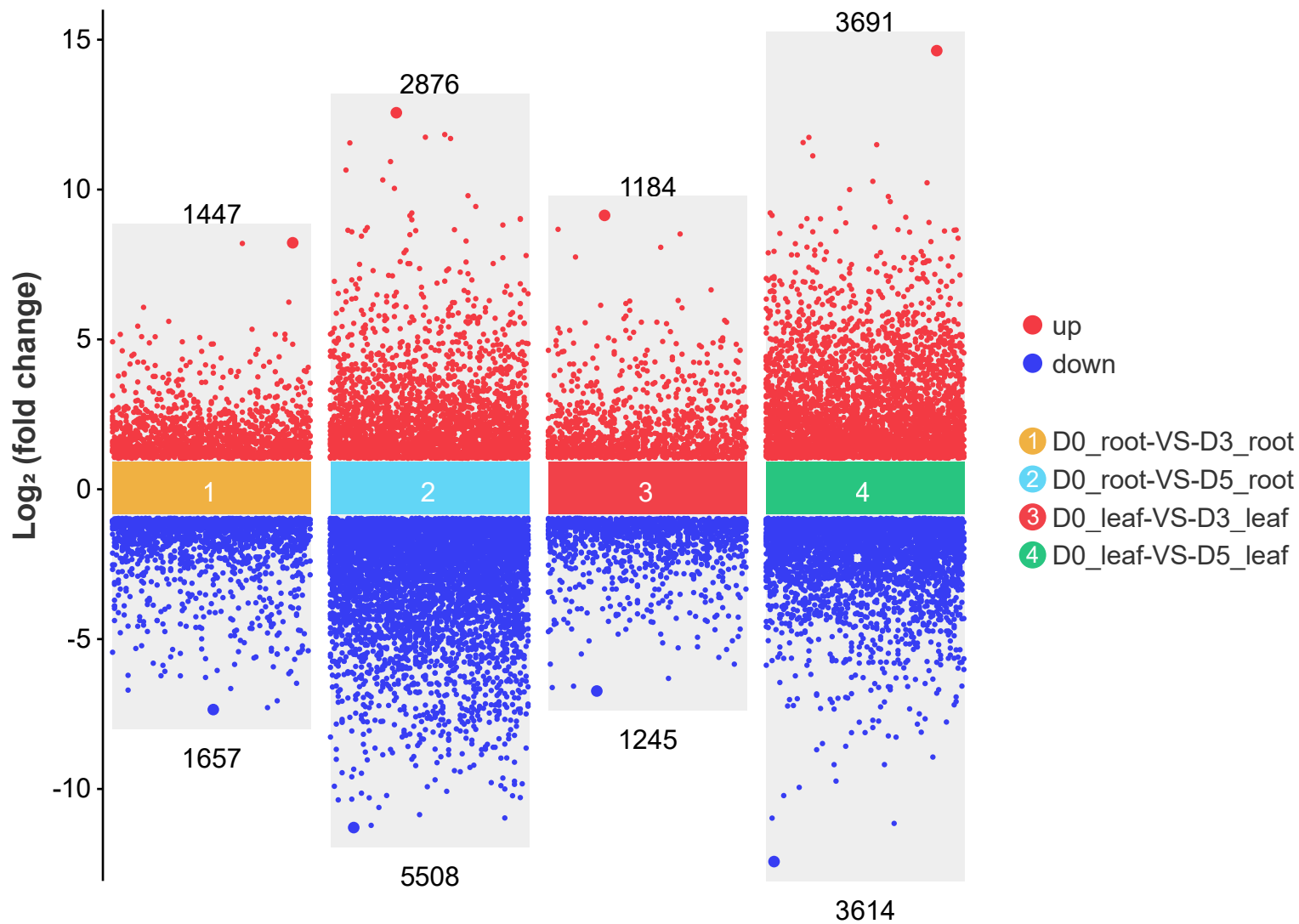

Supplement: giae118_Supplemental_Figures_and_Tables [file giae118_supplemental_figures_and_tables.zip › Figure S6.pdf]

a

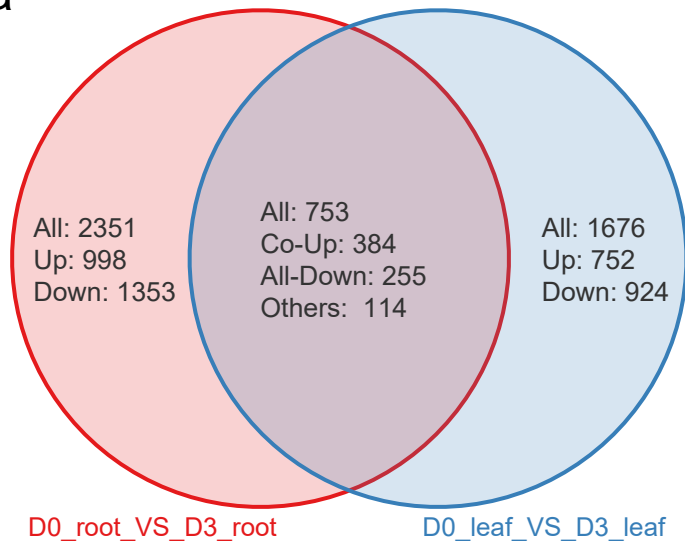

b

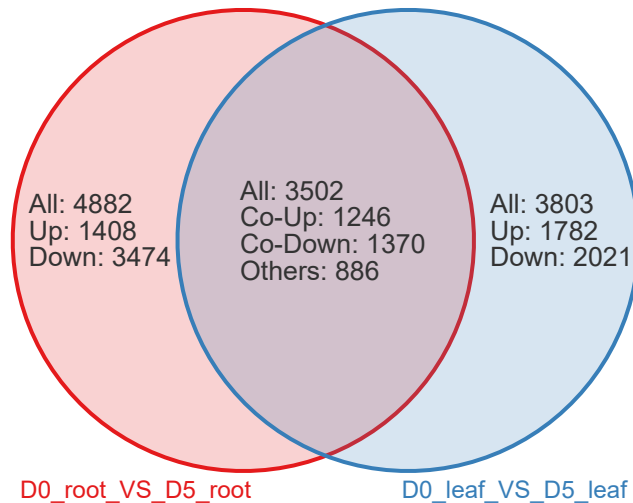

Supplement: giae118_Supplemental_Figures_and_Tables [file giae118_supplemental_figures_and_tables.zip › Figure S7.pdf]
