# Supplementary material for: A chromosome-scale genome assembly of the pioneer plant Stylosanthes angustifolia: insights into genome evolution and drought adaptation
Source: Gigascience. 2025 Jan 24;14:giae118. doi: 10.1093/gigascience/giae118 (PMC11758145; doi:10.1093/gigascience/giae118)
Supplement: giae118_GIGA-D-24-00294_Original_Submission [file giae118_giga-d-24-00294_original_submission.pdf]

GigaScience

The telomere-to-telomere (T2T) genome of the pioneer plant Stylosanthes angustifolia provides insight into its genome evolution and drought adaptation mechanisms

--Manuscript Draft--

**Manuscript Number:**	GIGA-D-24-00294
**Full Title:**	The telomere-to-telomere (T2T) genome of the pioneer plant Stylosanthes angustifolia provides insight into its genome evolution and drought adaptation mechanisms
**Article Type:**	Research
**Funding Information:**	Natural Science Foundation of Hainan Province (323CXTD387) — Prof. Pandao Liu<br>National Natural Science Foundation of China (32371769) — Prof. Pandao Liu<br>Earmarked Fund for China Agriculture Research System (CARS-34 and CARS-22) — prof. Guodao Liu<br>Central Public-interest Scientific Institution Basal Research Fund for CATAS (1630032022023) — Prof. Pandao Liu<br>Agricultural Research Outstanding Talents and Innovation Team of MARA (13210268) — Prof. Pandao Liu<br>Guangxi Special Project for Innovation-driven Development (Guike AA18242040) — Dr. Zhu Qiao

Funding Information:

Funder	Recipient
Natural Science Foundation of Hainan Province (323CXTD387)	Prof. Pandao Liu
National Natural Science Foundation of China (32371769)	Prof. Pandao Liu
Earmarked Fund for China Agriculture Research System (CARS-34 and CARS-22)	prof. Guodao Liu
Central Public-interest Scientific Institution Basal Research Fund for CATAS (1630032022023)	Prof. Pandao Liu
Agricultural Research Outstanding Talents and Innovation Team of MARA (13210268)	Prof. Pandao Liu
Guangxi Special Project for Innovation-driven Development (Guike AA18242040)	Dr. Zhu Qiao

Abstract:

Background

Drought is a significant limiting factor affecting plant survival and crop production. Stylosanthes angustifolia is a pioneer plant with drought-tolerant traits, but the molecular mechanisms underlying its drought tolerance remain poorly understood.

Results

Here, we report the first telomere-to-telomere (T2T) genome assembly of S. angustifolia, aiming to elucidate its genome evolution and drought tolerance mechanisms. The assembled genome spans 631.17 Mb, containing 319.98 Mb of repetitive sequences and 36,857 protein-coding genes. Notably, we detected 19 telomeric sequences and 10 centromeric sequences across 10 chromosomes within the S. angustifolia genome. The high quality of this genome is evidenced by the presence of 99.26% Benchmarking Universal Single-Copy Orthologs, a 19.49 LTR assembly index, and 96.37% k-mer completeness. Evolutionary analyses revealed that S. angustifolia shares whole-genome duplication event (WGD) with other legumes but lacks recent WGD. Additionally, S. angustifolia experienced gene expansion through tandem duplication (TD) approximately 12.31 million years ago. By combining multi-omics analyses, we identified four gene families, namely xanthoxin dehydrogenase, 2-hydroxyisoflavanone dehydratase, patatin-related phospholipase A, and raffinose synthase, that underwent TD and were significantly up-regulated under drought stress in the leaves of S. angustifolia. These gene families contributed to the biosynthesis of abscisic acid, genistein, daidzein, jasmonoyl-isoleucine, and raffinose, thereby enhancing drought tolerance.

Conclusions

Our T2T genome assembly of S. angustifolia represents an unprecedented achievement for the pioneer plant genus Stylosanthes. It provides a molecular basis for understanding the evolution of drought-tolerant traits in S. angustifolia and offers valuable genetic resources for improving drought resistance in other crops.

Corresponding Author: Pandao Liu
Chinese Academy of Tropical Agricultural Sciences Tropical Crops Genetic Resources

Powered by Editorial Manager® and ProduXion Manager® from Aries Systems Corporation

	Institute<br>Haikou, CHINA
**Corresponding Author Secondary Information:**	
**Corresponding Author's Institution:**	Chinese Academy of Tropical Agricultural Sciences Tropical Crops Genetic Resources Institute
**Corresponding Author's Secondary Institution:**	
**First Author:**	Chun Liu
**First Author Secondary Information:**	
**Order of Authors:**	Chun Liu
	Jianyu Zhang
	Ranran Xu
	Jinhui Lv
	Zhu Qiao
	Mingzhou Bai
	Shancen Zhao
	Lijuan Luo
	Guodao Liu
	Pandao Liu
**Order of Authors Secondary Information:**	
**Additional Information:**	
**Question**	**Response**
Are you submitting this manuscript to a special series or article collection?	No
**Experimental design and statistics**<br><br>Full details of the experimental design and statistical methods used should be given in the Methods section, as detailed in our Minimum Standards Reporting Checklist. Information essential to interpreting the data presented should be made available in the figure legends.<br><br>Have you included all the information requested in your manuscript?	Yes
**Resources**<br><br>A description of all resources used, including antibodies, cell lines, animals and software tools, with enough	Yes

Powered by Editorial Manager® and ProduXion Manager® from Aries Systems Corporation

information to allow them to be uniquely identified, should be included in the Methods section. Authors are strongly encouraged to cite Research Resource Identifiers (RRIDs) for antibodies, model organisms and tools, where possible.<br><br>Have you included the information requested as detailed in our Minimum Standards Reporting Checklist?	
**Availability of data and materials**<br><br>All datasets and code on which the conclusions of the paper rely must be either included in your submission or deposited in publicly available repositories (where available and ethically appropriate), referencing such data using a unique identifier in the references and in the “Availability of Data and Materials” section of your manuscript.<br><br>Have you have met the above requirement as detailed in our Minimum Standards Reporting Checklist?	Yes

Powered by Editorial Manager® and ProduXion Manager® from Aries Systems Corporation

Manuscript
Click here to access/download;Manuscript;manuscript-SAN.docx

The telomere-to-telomere (T2T) genome of the pioneer plant *Stylosanthes angustifolia* provides insight into its genome evolution and drought adaptation mechanisms

Chun Liu[1,2,3,4], Jianyu Zhang[1,2,3,4], Ranran Xu[1,2,3,4], Jinhui Lv[1,2,3,4], Zhu Qiao[5], Mingzhou Bai[6], Shancen Zhao[7], Lijuan Luo[1], Guodao Liu[2,*], and Pandao Liu[2,3,4,*]

[1] School of Tropical Agriculture and Forestry & Sanya Institute Breeding and Multiplication, Hainan University, Haikou/Sanya 570228/572025, China.

[2] Tropical Crops Genetic Resources Institute, Chinese Academy of Tropical Agricultural Sciences (CATAS), Haikou 571101, China

[3] Key Laboratory of Crop Gene Resources and Germplasm Enhancement in Southern China, Ministry of Agriculture and Rual Affairs, Haikou 571101, China.

[4] Key Laboratory of Tropical Crops Germplasm Resources Genetic Improvement and Innovation of Hainan Province, Haikou 571101, China.

[5] Guangxi Key Laboratory of Medicinal Resources Protection and Genetic Improvement/ Guangxi Engineering Research Center of TCM Resource Intelligent Creation, Guangxi Botanical Garden of Medicinal Plants, Nanning 530023, China.

[6] Department of Biotechnology and Biomedicine, Technical University of Denmark, Kongens Lyngby 2800, Denmark.

[7] Beijing Life Science Academy, Beijing 102200, China.

E-mail addresses of all authors
Chun Liu: xiaoyaoma@live.cn
Jianyu Zhang: jianyuzhang@hainanu.edu.cn
Ranran Xu: 2512891913@qq.com
Jinhui Lv: lvjinhuixue@163.com
Zhu Qiao: qiaozhu@gxyyzwy.com
Mingzhou Bai: mingbai@dtu.dk
Shancen Zhao: zhaosc@blsa.com.cn
Lijuan Luo: luoljd@126.com
Guodao Liu: Guodao_Liu@163.com
Pandao Liu: liupandao2019@163.com

*Correspondence addresses.
Pandao Liu, CATAS, West Xueyuan Road, Haikou 571101, China. E-mail: liupandao2019@163.com, ORCID: 0000-0003-2296-9130;
Guodao Liu, CATAS, West Xueyuan Road, Haikou 571101, China. E-mail: Guodao_Liu@163.com, ORCID: 0000-0003-4189-9959.

Abstract

Background: Drought is a significant limiting factor affecting plant survival and crop production. *Stylosanthes angustifolia* is a pioneer plant with drought-tolerant traits, but the molecular mechanisms underlying its drought tolerance remain poorly understood.

Results: Here, we report the first telomere-to-telomere (T2T) genome assembly of *S. angustifolia*, aiming to elucidate its genome evolution and drought tolerance mechanisms. The assembled genome spans 631.17 Mb, containing 319.98 Mb of repetitive sequences and 36,857 protein-coding genes. Notably, we detected 19 telomeric sequences and 10 centromeric sequences across 10 chromosomes within the *S. angustifolia* genome. The high quality of this genome is evidenced by the presence of 99.26% Benchmarking Universal Single-Copy Orthologs, a 19.49 LTR assembly index, and 96.37% k-mer completeness. Evolutionary analyses revealed that *S. angustifolia* shares whole-genome duplication event (WGD) with other legumes but lacks recent WGD. Additionally, *S. angustifolia* experienced gene expansion through tandem duplication (TD) approximately 12.31 million years ago. By combining multi-omics analyses, we identified four gene families, namely *xanthoxin dehydrogenase*, *2-hydroxyisoflavanone dehydratase*, *patatin-related phospholipase A*, and *raffinose synthase*, that underwent TD and were significantly up-regulated under drought stress in the leaves of *S. angustifolia*. These gene families contributed to the biosynthesis of abscisic acid, genistein, daidzein, jasmonoyl-isoleucine, and raffinose, thereby enhancing drought tolerance.

Conclusions: Our T2T genome assembly of *S. angustifolia* represents an unprecedented achievement for the pioneer plant genus *Stylosanthes*. It provides a molecular basis for understanding the evolution of drought-tolerant traits in *S. angustifolia* and offers valuable genetic resources for improving drought resistance in other crops.

Keywords: *Stylosanthes angustifolia*, Pioneer plant, T2T genome, Multi-omics, Drought tolerance

Introduction

Drought is a severe environmental challenge that significantly impairs plant survival and substantially reduces annual crop yields [1,2]. Plants have developed a range of physiological, biochemical, and morphological mechanisms to respond to drought stress. These mechanisms

include stomatal closure to reduce transpiration, changes in root architecture to optimize water uptake, and increased biosynthesis of abscisic acid (ABA), osmoprotectants, flavonoids, and isoflavonoids [1,3–5]. Additionally, the accumulation of non-reducing sugars, such as raffinose, plays a crucial role in drought adaptation [6]. Understanding the genes and molecular pathways underlying these adaptive responses is essential for advancing our ability to develop drought-tolerant crop cultivars [7–9]. Consequently, comprehensive research into the genetic and molecular mechanisms that enable plants to withstand drought stress is urgently needed.

The genus *Stylosanthes*, belonging to the Leguminosae family, comprises approximately 50 species of diploids, tetraploids, and hexaploids, which are distributed across tropical and subtropical regions [10]. This genus of plants exhibits strong adaptability to frequent stress conditions in acid soils of tropical and subtropical areas, including low phosphorus availability [11,12], aluminum toxicity [13,14], manganese toxicity [15,16], low pH [17], and drought [18]. Given these outstanding traits, this genus is often planted as a "pioneer plant" on newly reclaimed arable or marginal lands with acid soils, serving as green manure or forage [19]. Notably, *S. angustifolia* (NCBI:txid79067) has demonstrated significant drought tolerance in infertile acid soils [20,21]. However, the genetic and molecular basis of its drought-tolerant traits remains unreported.

With the advent of third-generation sequencing (TGS) technologies, notably Pacific Biosciences (PacBio) and Oxford Nanopore Technologies (ONT) sequencing technologies, it is now possible to assemble genomes at the telomere-to-telomere (T2T) level [22,23]. PacBio high-fidelity (HiFi) sequencing technology generates reads of 18-25 kilobases (kb) with over 99.9% accuracy [24], whereas ONT reads typically have median lengths of 50-150 kb with accuracies around 95% [25]. Reference genomes at the T2T level can be assembled using either or both types of TGS reads. Assembling plant genomes has always been challenging due to their high heterozygosity and repetitive sequences, and polyploidy [26]. Recently, T2T or near-T2T assemblies of model plants such as *Arabidopsis thaliana* [27,28], graminaceous crops like rice (*Oryza sativa*) [29,30] and maize (*Zea mays*) [31], leguminous crops such as soybean (*Glycine max*) [32,33], and economic crops like grapevine (*Vitis vinifera*) [34] have been accomplished using the latest sequencing technologies and assembly algorithms. These assemblies offer critical genetic bases for advancing various aspects of basic biology and agricultural applications, facilitating high-precision studies of plant genomes and

molecular breeding efforts. So far, only the genome of the tetraploid *S. scabra* has been deciphered using next-generation sequencing (NGS) within the genus *Stylosanthes* [35], while T2T assemblies have been completed for only a few legume species, including soybean [32,33] and the allotetraploid *Sesbania cannabina* [36]. There remains a significant need to decode more legume genomes at the T2T level to enhance our understanding and utilization of these important plant species.

Gene duplication is a pivotal mechanism through which organisms acquire new genes and generate genetic diversity, playing a crucial role in plant evolution and adaptation [37–39]. Tandem duplication (TD) significantly drives gene family expansion, often resulting in gene members with nearly identical sequences and potentially redundant functions. Recent studies increasingly show that the expansion of tandem duplicated genes (TDGs) contributes to plant environmental adaptation. For instance, we have previously reported that TDGs facilitate adaptation to low phosphorus stress in pigeonpea (*Cajanus cajan*) [40,41]. Additionally, TDGs are involved in environmental responses and adaptive evolution in grapevine [42] and silver birch (*Betula pendula*) [43]. The expansion of lineage-specific TDGs is vital for adaptive evolution in rapidly changing environments [44]. Whole-genome duplication (WGD) and TD also drive the expansion of salinity adaptation genes in the halophyte *Tamarix chinensis* [45]. Comprehensive genome-wide identification of TDGs, combined with multi-omics data analysis, can effectively elucidate the crucial roles of these genes in genome evolution and adaptation to adverse environmental conditions [41,45–47].

In this study, we sequenced and assembled a T2T genome of the leguminous pioneer plant *S. angustifolia* using ONT, NGS, and high-through chromosome conformation capture (Hi-C) sequencing. Comparative genomics analyses clarified the divergence time and evolutionary position of the *S. angustifolia* through phylogenetic analyses. Furthermore, comparative genomics, transcriptomics, and metabolomics demonstrated that TDGs play an essential role in genome evolution and adaptation to drought stress in *S. angustifolia*.

Results

Sequencing and Assembly of the Genome of *S. angustifolia*

In this study, we employed NGS and ONT technologies for the whole-genome sequencing of *S.*

angustifolia (Germplasm number: TF0003 and FigureS1). A total of 35.08 Gb (~ 53.02 × coverage) NGS data and 104.72 Gb (~ 158.29 × coverage) ONT data were generated (Table S1 and Table S2). Initially, we estimated the genome size of *S. angustifolia* to be 661.55 Mb using k-mer analysis of the NGS data. NextDenovo was adopted to correct and assemble the raw ONT data into initial contigs. Subsequently, NextPolish was employed to polish the initial contigs using both ONT and NGS data to obtain high-quality contig sequences. Furthermore, we utilized Hi-C for chromosomal-level assembly of the *S. angustifolia* genome, generating 53.89 Gb (~ 81.47 × coverage) clean data (Table S3). Further analysis with Juicer and 3dDNA software aligned the Hi-C data to high-quality contigs, facilitating chromosome grouping and ordering to achieve a chromosomal-level assembly of the *S. angustifolia* genome. Additionally, LR_Gapcloser was applied to fill the remaining gaps within the chromosomes. The final assembled genome size of *S. angustifolia* was 631.17 Mb, with a GC content of 35.53%, and a contig N50 of 64.91 Mb (Figure 1 and Table 1). There was only one gap in chromosome 7 of the assembled genome (Figure 1a). Moreover, telomeric sequences "TTTAGGG" were identified at the ends of all chromosomes except one terminus on chromosomes 7 and 10 (Figure 1a and Table S4). Assessment of the assembled genome using Benchmarking Universal Single-Copy Orthologs (BUSCO) and LTR assembly index (LAI) software revealed completeness scores of 99.26% and 19.49, respectively (Table 1 and Table S5). Additionally, k-mer completeness analysis yielded a score of 96.37% for the *S. angustifolia* genome (Table 1). Alignment of NGS and ONT reads to the assembled genome showed mapping rates of 99.15% and 99.69%, respectively, with average depths of 54.16 for NGS reads and 156.20 for ONT reads. Overall, 95.16% and 99.96% of the genomic sequences were covered by NGS and ONT reads, respectively.

Table 1. Statistics of genomic features of *S. angustifolia*

**Terms**	***S. angustifolia***
Estimated genome size (Mb)	661.55
Assembled genome size (Mb)	631.17
Contig N50 (Mb)	64.91
GC content (%)	35.53

BUSCO (%)	99.26
LTR assembly index	19.49
k-mer completeness (%)	96.37
Number of chromosomes	10
Number of identified telomere sequences	18
Repeat content (Mb)	319.98
Repeat ratio (%)	50.70
Number of protein-coding genes	36,857
Mean exon length (Bp)	242.95
Mean intron length (Bp)	465.57

Identification of candidate centromeres on the *S. angustifolia* genome

Centromeres are regions on dividing chromosomes where spindle microtubules bind, and the centromeric region has the characteristics of the high density of short tandem repeats and low gene density. Our telomere-to-telomere assembled *S. angustifolia* genome makes it possible to investigate these regions. We identified tandem repeats and long terminal repeats (LTRs) across the chromosomes and then we employed quarTeT for the identification of centromeric regions. The location and length of the centromeres differ significantly among the ten chromosomes of the *S. angustifolia* genome (Table 2). There were four chromosomes (chr2, chr5, chr8, and chr9) comprising long centromere regions over 10 Mb and two chromosomes (chr1 and chr6) comprised short centromere regions less than 1Mb (Table 2). Interestingly, the GC content in the centromere region was similar, except for lower GC content for chr1 (Table 2).

Table 2. Identification of centromeres in the assembled *S. angustifolia* genome.

**Chr**	**Start**	**End**	**Length**	**GC (%)**
chr1	75,244,451	75,385,629	141,179	25.43
chr2	28,643,039	45,565,248	16,922,210	37.80
chr3	37,114,288	40,318,197	3,203,910	37.91

chr4	34,275,759	39,959,271	5,683,513	38.07
chr5	22,763,480	48,374,971	25,611,492	37.84
chr6	6,161,638	6,404,824	243,187	35.59
chr7	38,083,163	40,541,321	2,458,159	36.77
chr8	23,443,538	38,727,823	15,284,286	37.88
chr9	19,059,531	30,598,702	11,539,172	38.08
chr10	6,130,200	8,349,710	2,219,511	38.32

Genome annotation of the *S. angustifolia* genome

Both *de novo* and homology-based approaches were adopted for repetitive sequence annotation. A total of 319.98 Mb repetitive sequences were obtained, accounting for 50.70% of the *S. angustifolia* genome (Table 1). The LTRs were the most abundant repeat families, accounting for 42.91% of the genome, followed by DNA transposons (2.49% of the genome) and long interspersed nuclear element (LINE) (1.64% of the genome) (Table 1, Table S6 and Table S7).

To assist in the prediction of protein-coding genes, we performed transcriptome sequencing on the roots, stems, leaves, flowers, and seeds of *S. angustifolia*, producing a total of 40.40 Gb of clean data (Table S8). Transcriptome assembly was performed using the reference-based and *de novo* assembly and transcripts from both reference-based and *de novo* assembly were adopted for protein-coding genes prediction. We predicted protein-coding genes in the *S. angustifolia* genome based on *ab initio* prediction, homology-based prediction, and transcriptomic evidence. As a result, we identified 36,857 protein-coding genes in the *S. angustifolia* genome, with an average exon length of 242.95 base pairs (bp) and an average intron length of 465.57 bp (Table 1). The gene set completeness was assessed using BUSCO, revealing that the predicted gene set has a completeness of 97.71% (92.50% single-copy BUSCOs and 5.20% duplicated BUSCOs) (Table S9). Furthermore, functional annotation of the gene set indicated that 98.94% of the genes were annotated, with 68.80% and 65.07% being annotated in the Kyoto Encyclopedia of Genes and Genomes (KEGG) and Gene Ontology (GO) databases, respectively (Table S10). In addition, we predicted non-coding RNAs (ncRNAs) in the *S. angustifolia* genome, identifying 97 miRNAs, 3,048 snRNAs, and 573 tRNAs (Table S11).

Comparative genomic analysis among leguminous plants and Arabidopsis

To investigate the evolutionary relationships of *S. angustifolia*, we conducted gene family and phylogenetic analyses on nine leguminous plants, as well as Arabidopsis. Gene clustering among the studied species identified 31,810 orthologous groups (OGs), with 9,460 OGs being shared across all species. Additionally, 633 OGs were single-copy, and 578 OGs were specific to *S. angustifolia* (Figure 2a).

Based on the single-copy OGs, we constructed a phylogenetic tree, revealing that *S. angustifolia*, along with the wild relatives of peanuts, *A. duranensis*, and *A. ipaensis*, form sister branches (Figure 2b). This phylogenetic tree further confirmed their evolutionary position within the subtribe *Stylosanthinae* (Benth.) of the legume family. Furthermore, to explore the expansion and contraction of gene families in *S. angustifolia*, we analyzed gene clustering and expansion/contraction patterns with six species. This analysis identified 2,089 expanded gene families in *S. angustifolia*, with 158 OGs significantly expanded ($P < 0.05$) (Figure S3). These 158 significantly expanded OGs encompassed 3,071 genes, primarily associated with KEGG pathways such as "carbohydrate metabolism", "Biosynthesis of other secondary metabolites", and "lipid metabolism" (Figure S4).

In addition, collinear gene blocks within and between *S. angustifolia*, soybean, *A. duranensis*, and *A.ipaensis* were identified. Despite some genomic rearrangements in *S. angustifolia*, *A. duranensis*, and *A.ipaensis*, such as in chr3, chr9, and chr10 in *S. angustifolia*, they still maintain relatively good genomic collinearity (Figure 2c). The Ks distribution of collinear gene pairs revealed that *S. angustifolia* shared the ancestral Papilionoideae whole-genome duplication event (PWGD) with soybean, *A. duranensis*, and *A. ipaensis* (Figure 2d), with similar Ks peaks for *S. angustifolia*, *A. duranensis*, and *A. ipaensis* (Ks peak ~ 0.8, estimated time at 49.26 million years ago (MYA)) (Figure 2d). Furthermore, interspecies divergence time was calculated based on the Ks peak of inter-species collinear gene pairs, revealing the split of *S. angustifolia* and these two Arachis species occurring at about 11 MYA, which was similar to the divergence time estimated by single-copy gene families (Figure 2b, 2d).

Transcriptome analysis and genome evolution of *S. angustifolia* in adaptation to drought stress

To identify genes in *S. angustifolia* responding to drought stress, we conducted transcriptome sequencing on roots and leaves subjected to drought and control treatments (Figure 3a). The transcriptome sequencing generated a total of 86.66 Gb clean data (average 7.22 Gb per sample) with Q30 > 93.54% (Table S12). Further analysis of gene expression levels revealed 28,751 genes expressed under control and drought conditions. Additionally, a total of 8,384 and 7,305 differentially expressed genes (DEGs) were identified in roots and leaves under drought stress, respectively. In roots, 2,876 were up-regulated and 5,508 were down-regulated, whereas in leaves, 3,691 were up-regulated and 3,614 were down-regulated in leaves (Figure 3b and Figure S5).

Intersection analysis of expanded genes in *S. angustifolia* and DEGs revealed 155 and 166 genes that were up-regulated in roots and leaves, respectively, under drought stress (Figure 3c, d). Further enrichment analysis of these expanded and up-regulated genes revealed enrichment in the "carotenoid biosynthesis pathway" (map00906, Q-value < 0.05) in both roots and leaves (Figure 3e, Table S13 and Table S14). Functional analysis revealed that genes enriched in carotenoid biosynthesis were *xanthoxin dehydrogenase* (*ABA2*, K09841), a key gene family involved in ABA biosynthesis (Table S13 and Table S14). Genome-wide identification of *ABA2* genes in *S. angustifolia*, soybean, barrel medic, and Arabidopsis showed that the number of *ABA2* genes was higher in the studied leguminous plants compared to Arabidopsis (Table S15). Notably, seven *ABA2* genes independently underwent TD on chromosome 4 of *S. angustifolia*, with divergence time analysis indicating these duplications occurred at approximately 24.12 MYA, with the most recent duplication occurring at 5.01 MYA (Figure 4b, c and Table S16). More importantly, among the seven independently tandem duplicated *ABA2* genes, six were up-regulated in roots and four were up-regulated in leaves under drought stress (Figure 4a). To investigate whether the expansion and up-regulation of *ABA2* genes under drought stress led to the accumulation of ABA, we quantitatively measured ABA content. Results showed that compared to controls, drought treatment led to a 20.43-fold increase in ABA content in the roots and a 5.05-fold increase in the leaves of *S. angustifolia* (Figure 4d, e).

Additionally, genes that were expanded and up-regulated in the leaves of *S. angustifolia* were significantly enriched in the "Isoflavonoid biosynthesis pathway" (map00943) (Figure 3e). Functional analysis of these genes revealed the inclusion of three *2-hydroxyisoflavanone*

dehydratase (*HIDH*, K13258) genes, which are involved in the biosynthesis of genistein and daidzein (Table S14). Phylogenetic and microsynteny analyses indicated a specific expansion event of *HIDH* genes in *S. angustifolia*, particularly through TD on chromosome 2 (Figure 5a, b, and Table S17). These *HIDH* genes expanded approximately 68.16 MYA in the *S. angustifolia* genome, with the most recent duplication occurring at 11.11 MYA (Table S18). Measurements of genistein and daidzein revealed that, under drought treatment, the contents of genistein and daidzein in *S. angustifolia* leaves were 5.48 times and 1.94 times higher, respectively, than those in the control leaves (Figure 5c, d).

Contribution of TDGs to drought tolerance of *S. angustifolia*

The *S. angustifolia* genome does not exhibit recent WGDs (Figure 2d), and gene families analysis revealed that TD plays an important role in gene expansion in the *S. angustifolia* genome (Figure 3, Figure 4, and Figure 5). Therefore, we performed a genome-wide identification of TDGs and investigated their response to drought stress in *S. angustifolia*. As a result, we identified 3,634 TDGs in the *S. angustifolia* genome, which is more than in *A. duranensis* (2,735) and *A. ipaensis* (3,449) but fewer than in soybean (5,022) and barrel medic (7,032). By plotting the Ks distribution of TDGs, we determined that TDGs in *S. angustifolia* underwent substantial expansion approximately 12.31 MYA (Ks peak at ~0.2) (Figure 6a). TDGs in *S. angustifolia* were significantly enriched in KEGG pathways, including "Biosynthesis of secondary metabolites", "Flavonoid biosynthesis", and "Isoflavonoid biosynthesis" (Figure 6b). To investigate the differential expression of TDGs in response to drought stress in *S. angustifolia*, we compared TDGs with DEGs. The results indicated that 537 and 464 TDGs were up-regulated and down-regulated in leaves under drought stress, respectively (Figure 6c). Further KOG classification of the 537 up-regulated TDGs in leaves revealed that 30 genes belong to "Lipid transport and metabolism" (Figure 6d).

Lipid metabolism in *S. angustifolia* leaves in response to drought stress

To evaluate the impact of drought stress on lipid metabolism in *S. angustifolia* leaves, we conducted a lipidomic analysis on leaves with or without drought stress. A total of 912 lipids across 7 classes were identified, with 134 being differentially accumulated lipids (DALs), including 52 up-regulated

and 82 down-regulated lipids (Table S19 and Figure S6). These DALs primarily belong to four classes: glycerophospholipids, sulfolipids, galactolipids, and glycerolipids (Figure 6e and Table S19).

In the *S. angustifolia* genome, we discovered that *patatin-related phospholipase A* (*pPLA*) underwent TD approximately 18.67 million years ago (Ks = 0.30) (Figure S7 and Table S20). This gene family is known to degrade membrane lipids containing C18:3 chains, releasing α-linolenic acid (ALA, C18:3) [48]. Transcriptome analysis showed significant up-regulation of four *pPLA* genes in drought-treated leaves (Figure 7 and Table S20). Correspondingly, the lipidomic analysis revealed a significant reduction in the accumulation of membrane lipids containing C18:3 chains (e.g., PE, PC, MDGD, SQDG) in *S. angustifolia* leaves under drought stress (Figure 6e). As *ALA* is a precursor for jasmonic acid (JA) biosynthesis, we identified and analyzed the expression changes of JA synthesis pathway-related genes. The results showed substantial upregulation of genes in the JA synthesis pathway in drought-treated *S. angustifolia* leaves, including two *lipoxygenases* (*LOXs*), two *allene oxide synthases* (*AOSs*), two *allene oxide cyclases* (*AOCs*), three *12-oxophytodienoate reductases* (*OPRs*), two *OPC-8: CoA ligase 1s* (*OPCL1s*), three *acyl-CoA oxidases* (*ACXs*), three *acetyl-CoA acyltransferase 1s* (*ACAA1s*), three *multifunctional protein 2s* (*MFP2s*), and two *jasmonate resistant 1s* (*JAR1s*) (Figure 7, Table S21 and Table S22). Additionally, we quantitatively measured JA and jasmonoyl-L-isoleucine (JA-Ile) levels and found that, under drought treatment, the JA and JA-Ile contents in *S. angustifolia* leaves were 1.77 times and 5.47 times higher, respectively, than those in the control leaves (Figure 7).

Interestingly, the lipidomic analysis identified 15 phosphatidylinositols (PIs) as DALs, all of which significantly decreased in accumulation after drought treatment (Table S19). Degradation of PIs can produce inositol, which can further synthesize raffinose (Figure 8a). Transcriptome results showed a substantial upregulation of genes involved in raffinose biosynthesis in drought-treated *S. angustifolia* leaves, including four phosphoinositide-specific phospholipase Cs (*PLCs*), three *inositol 3-alpha-galactosyltransferases* (*GOLSs*), and six *raffinose synthases* (*RAFSs*) (Figure 8b and Table S23). Consistently, the raffinose content in *S. angustifolia* leaves subjected to drought treatment was 3.25 times higher than that in the control leaves (Figure 8c). Notably, among the six upregulated *RAFS* genes in leaves, two were expanded by TD in the *S. angustifolia* genome at

approximately 18.02 MYA (Figure 8c).

Discussion

Exploring the potential mechanisms by which pioneer plants adapt to harsh environmental conditions can help improve stress tolerance traits in crops [49,50]. Despite the fact that the pioneer plant *S. angustifolia* exhibits exceptional drought-tolerant traits, the lack of high-quality genomic resources has significantly impeded the elucidation of the molecular mechanisms underlying its drought resistance. In this study, we present the first T2T genome of *S. angustifolia*, achieved by integrating NGS, ONT, and Hi-C sequencing technologies, with a genome size of 631.17 Mb. Various genome assessment methods demonstrated that our assembled genome exhibits high integrity and accuracy (Table 1 and Figure 1). The assembled T2T genome made it possible for us to explore the telomeric and centromeric sequences. The centromeric sequences of *S. angustifolia* are diverse in chromosomal location and length, yet relatively conserved in GC content (Table 2). The T2T genome of *S. angustifolia* will be invaluable for further evolutionary, genetic, and functional studies, as well as for the molecular breeding of *S. angustifolia* and other plants in the genus *Stylosanthes*.

Gene duplication is a crucial evolutionary mechanism that contributes to genome adaptation to various environments [37,38,51]. Particularly, TD is a significant characteristic in secondary metabolite biosynthesis pathways and biotic and abiotic stress responses [52,53]. For instance, the expansion of sugar metabolism-related genes *α-amylase* (*AMY3*) and *β-fructofuranosidase* (*CWINV1*) in *Sophora moorcroftiana* contributes to high sucrose content, promoting long root growth and enhancing drought tolerance [54]. In orchardgrass (*Dactylis glomerata*), the DgMADS-box genes have expanded via TD, contributing to longer root lengths and higher survival rates under various abiotic stresses [55]. Similarly, the RWP-RK gene family in the Pearl millet (*Pennisetum glaucum*) genome has expanded, enabling rapid responses to heat stress by regulating the expression of endoplasmic reticulum (ER)-related genes [56]. Our previous studies have also demonstrated that TDGs play an important role in the adaptation of pigeonpea (*Cajanus cajan*) to low phosphorus stress [40,41].

In this study, we found that TDGs play a crucial role in the adaptation of *S. angustifolia* to drought

stress through an integration analysis of comparative genomics, transcriptomics, and metabolomics. The phytohormone ABA is known to play a crucial role in multiple abiotic stress conditions, notably, ABA is a key water stress hormone that triggers stomatal closure and reduces transpirational water loss under drought conditions [57–59]. The *ABA2* genes, involved in ABA biosynthesis [60], have undergone expansion through TD in the *S. angustifolia* genome. This gene is notably up-regulated in the roots and leaves of *S. angustifolia* under drought stress, thereby enhancing ABA synthesis and, in turn improving the adaptation of *S. angustifolia* to drought conditions (Figure 3 and Figure 4). Additionally, research on legume pigeonpea has demonstrated that ABA enhances drought resistance through the accumulation of genistein [4,61]. In our current study, we discovered that the *HIDH* gene, which is involved in the biosynthesis of genistein and daidzein [62], is specifically expanded in the *S. angustifolia* genome by TD. This gene exhibited significant up-regulation in *S. angustifolia* leaves under drought stress conditions, thereby facilitating the increased production of genistein and daidzein (Figure 5). The expansion in the copy number of *ABA2* and *HIDH* genes in the *S. angustifolia* genome and the upregulation of these two genes under drought stress represents one of the evolutionary mechanisms of *S. angustifolia* adaptation to drought stress.

JA is another important and widely distributed plant hormone that regulates the processes of plant growth, development, and defense. Several studies have reported that JA plays a crucial role in plant responses to drought stresses [5,63,64]. Recently, research in Arabidopsis discovered that *JAR1* facilitates the conversion of JA to JA-Ile [65], which also serves as a critical plant hormone and plays a significant role in enhancing plant adaptation to drought stress [66,67]. Our study revealed that *pPLA*, responsible for the degradation of C18:3 branched membrane lipids, underwent an expansion in the *S. angustifolia* genome at approximately 18.67 MYA through TD. Moreover, ten genes involved in the biosynthesis of JA and JA-Ile, including *pPLA* and *JAR1*, were significantly up-regulated in *S. angustifolia* leaves under drought stress (Figure 7). This upregulation, along with the expansion of *pPLA*, contributes to the increased accumulation of JA and JA-Ile, facilitating the adaptation of *S. angustifolia* to drought stress (Figure 7).

Raffinose, a non-reducing trisaccharide, accumulates in leaves under drought stress and plays a crucial role in plant drought tolerance [6,68]. RAFS catalyzes the biosynthesis of raffinose using sucrose and galactinol as substrates. Overexpression of *RAFS* gene in maize (*Zea mays*) [6] and

Arabidopsis [68] results in a significant increase in raffinose content, thereby enhancing drought tolerance by reducing leaf water loss. Our findings indicated that *RAFS* genes underwent TD in the *S. angustifolia* genome at approximately 18.02 MYA (Figure 8c). Six *RAFS* genes, including two from TD, were significantly up-regulated in *S. angustifolia* leaves under drought stress (Figure 8a). Correspondingly, drought treatment led to an increased accumulation of raffinose in the leaves of *S. angustifolia* (Figure 8b). These results suggest that the expansion of *RAFS* genes through TD in the *S. angustifolia* genome, combined with the upregulation of *RAFS* gene expression under drought stress, facilitates raffinose biosynthesis, thereby aiding *S. angustifolia* in adapting to drought conditions.

In summary, this study presents the first T2T genome of *S. angustifolia* and integrates comparative genomics, transcriptomics, and metabolomics to demonstrate that TD significantly contributes to the adaptation of *S. angustifolia* to drought stress.

Methods

Plant materials

S. angustifolia (Germplasm number: TF0003 and FigureS1) was obtained at the germplasm nursery of the Chinese Academy of Tropical Agricultural Sciences (CATAS) in Hainan, China. DNA was extracted from the young leaves of *S. angustifolia* using the CTAB method. The roots, stems, leaves, flowers, and seeds of *S. angustifolia* were collected for RNA extraction and transcriptome sequencing.

Genome Sequencing and Assembly

High-quality genomic DNA was extracted for the construction of ONT and NGS libraries construction at the Genome Center of Grandomics (Wuhan, China). The ONT library was sequenced using the ONT PromethlON platform at the Genome Center of Grandomics (Wuhan, China). The NGS library was sequenced using the MGI-SEQ 2000 platform (MGI Tech, China). Hi-C technology was adopted for chromosome-level genome assembly. Hi-C library construction and sequencing were performed at the Genome Center of Grandomics (Wuhan, China) using DPN II as

the restriction enzyme. Guppy (v6.5.7) was adopted for ONT reads processing and SOAPnuke (v2.1.7) was adopted for quality control of raw sequencing reads of NGS and Hi-C with the parameters '-n 0.01 -l 20 -q 0.3 --polyX 50'. ONT reads were corrected and assembled into initial contigs using NextDenovo (v2.3.0) [69] with "read_cutoff" set to 2k. The initial contigs were then polished using NextPolish (v1.4.1) [70] based on NGS and ONT reads. Clean reads from Hi-C were aligned to the polished contigs using the Burrows-Wheeler Aligner (BWA, v0.7.17), and Hi-C maps were generated using Juicer (v1.6). The 3D de novo assembly (3D-DNA, v180922) pipeline was adopted for chromosomal grouping, sorting, and orientation. Hi-C maps visualization and manual check was performed using Juicebox (v1.11.08). Finally, gaps within the chromosomes were closed in one iteration using LR_Gapcloser (https://github.com/CAFS-bioinformatics/LR_Gapcloser) [71]. The final assembled *S. angustifolia* genome contained only one gap on chromosome 7 (59505100 bp to 59505196 bp). Genome completeness was assessed using BUSCO (v5.3.2) analysis based on embryophyta_odb10 database, and LAI analysis using LTR_retriever (v2.9.0) [72]. Merqury (v1.3) was adopted for assembly completeness evaluation using an efficient k-mer set. ONT reads were aligned to the genome using minimap2 (v2.17-r941) and NGS reads were aligned to the genome using BWA (v0.7.17). SAMtools (v1.9) and PanDepth (v2.19) were adopted for genome mapping ratio and coverage statistics.

Genome annotation

We performed both *de novo* and homologue-based approaches for repetitive element identification. Extensive *de novo* TE Annotator (EDTA, v2.0.1) [73] and LTR_retriever (v2.9.0) [72] were adopted for *de novo* identification of transposable elements (TEs). Additionally, known TEs from RepBase (v21.12) were identified by employing RepeatMasker (v4.1.4). Tandem Repeat Finder (TRF, v. 4.09.1) was employed to detect the tandem repeats. Protein-coding gene prediction was performed based on the repeat-masked genome by employing *de novo*-based prediction, RNA-seq-based prediction, and homologue-based prediction. We employed GALBA (https://github.com/Gaius-Augustus/GALBA, v1.0.8) for automated training and prediction of protein-coding genes, utilizing AUGUSTUS (https://github.com/Gaius-Augustus/Augustus, v3.5.0) and miniport (https://github.com/lh3/miniprot, v0.13) with default parameters. SNAP (v. 2013-02-16) was also

adopted for *de novo* gene prediction. Transcriptome sequencing reads from roots, stems, leaves, flowers, and seeds were aligned to the *S. angustifolia* genome using HISAT2 (v2.2.1), and transcript construction was conducted using StringTie (v2.2.1) [74]. RNA *de novo* assembly was performed using Trinity (v2.15.1). Coding regions of the predicted transcripts were identified using TransDecoder (v 5.7.0). For homology-based prediction, proteins from five species, namely, *G. max*, *Medicago truncatula*, *A. ipaensis*, *Senna tora*, and *Arabidopsis thaliana*, were aligned to the *S. angustifolia* genome using tBLASTn (v2.13.0) and gene structure prediction were performed by miniport (v0.13). EVidenceModeler (https://github.com/EVidenceModeler/EVidenceModeler, v2.1.0) was adopted for the identification of non-redundance consensus genes from all available evidence. Furthermore, PASA (v2.5.3) was adopted to refine gene structure and annotate untranslated regions (UTRs) based on transcriptome data. The predicted protein-coding genes were aligned to various known databases, including NCBI Non-Redundant Protein Sequence Database (NR), KEGG, Eukaryotic Orthologous Groups of Protein (KOG), Swiss-Prot, TrEMBL, InterPro databases, for functional annotation. BLASTp (v2.13.0) was adopted for homology search against NR, KEGG, KOG, Swiss-Prot, and TrEMBL with parameters '-outfmt 6 -evalue 1e-10'. Blast2GO (v6.0) was adopted for GO annotation based on the NR annotation, The best hit for each gene was retained for subsequent analysis. Additionally, non-coding genes (ncRNAs) were predicted by BLASTn (v2.13.0) and INFERNAL (v1.0) based on the Rfam database (v12.0).

Identification of telomeres and centromeres

We employed a Telomere Identification Toolkit (tidk, https://github.com/tolkit/telomeric-identifier) for telomere identification by searching the sequence TTTAGGG. Centromeric regions are characterized by a high density of short tandem repeats and low gene density. Tandem repeats were identified using TRF (v4.09.1) and TEs were identified using LTR_retriever (v2.9.9). Finally, candidate centromeres were identified by employing quarTeT (v1.2.1) [75].

Comparative genomic analysis

Nine legumes, including *S. angustifolia, A. ipaensis*, *Arachis duranensis*, *Aeschynomene evenia*, *G. max*, *M. truncatula*, *Phaseolus vulgaris*, *Cajanus cajan*, and *Senna tora*, along with one non-legume,

Arabidopsis thaliana, were utilized for comparative analysis. Protein sequences were compared using BLASTp (v2.2.23, e-value set to 1e-5) in an all-vs-all alignment, and OrthoFinder (v2.5.4) was utilized for orthologous groups (OGs) identification and phylogenetic tree construction with parameters '-S diamond -M msa -A mafft'. Divergence times between *G. max*, *M. truncatula*, *C. cajan*, and *P. vulgaris* were queried on timetree (http://www.timetree.org/) as known divergence times. Based on single-copy OGs, the substitution rates were estimated using the MCMCTREE program within the PAML (v4.5) software package, further calculating the divergence times between species. MCScanX (https://github.com/wyp1125/MCScanX) was employed for intra- and inter-species gene collinearity analysis. First, protein sequences were aligned using BLASTp (v2.2.23, e-value set to 1e-5), and MCScanX was employed to identify collinear regions. For intra-species analysis, we employed the duplicate_gene_classifier, a program of MCScanX, to classify paralogous genes into single-copy genes, dispersed duplicated genes, proximal duplicated genes, TDGs, and whole genome or segmental duplicated genes. Microcollinearity of TDGs in *S. angustifolia* genome compared with soybean and Medicago was performed using MCscan-(Python-version) (https://github.com/tanghaibao/jcvi). Additionally, the nonsynonymous (Ka) and synonymous (Ks) substitution rates of gene pairs within the collinear regions, as well as the Ka and Ks of TDGs were calculated using PAML (v4.9e) and PAL2NAL (v14) using the Nei-Gojobori (NG) method [76]. R platform (v4.0.2) was adopted for Ks distribution visualization and peak identification. The divergence time of gene pairs was calculated using the formula T=Ks/2r, where the neutral substitution rate r was selected as 8.12×10^{-9} in this study [77].

Drought treatment, transcriptome sequencing, and bioinformatics analysis

Seeds of *S. angustifolia* were sown on soil in pots, and the seedlings were grown for 60 days in a greenhouse with daily watering. The drought treatment group consisted of 60-day-old seedlings that were not watered for 5 days, while the control group consisted of 60-day-old seedlings that were not subjected to the drought treatment. Samples of roots and leaves were collected separately, and RNA was extracted for transcriptome sequencing. Each treatment group in this experiment consisted of three biological replicates, with each biological replicate containing 20 seedlings. Transcriptomes were sequenced on the MGI-SEQ 2000 platform (MGI Tech, China) and SOAPnuke (v2.1.7) [78]

was adopted for quality controlling of the raw sequencing reads. Clean reads were mapped onto the *S. angustifolia* genome using HISAT2 (v2.2.1). The featureCounts (v2.0.6) were employed for the calculation of gene read counts and an in-house Perl script was adopted for (transcripts per million, TPM) calculation. DEGs were identified using DESeq2 (v3.19), and the BH method was adopted for false discovery rate (FDR) calculation. Genes with $|\log_2$ fold change$| > 1$ FDR < 0.05 were considered as DEGs. KEGG pathway enrichment analysis of DEGs was performed by the phyper and *p.adjust* functions under the R platform (v4.0.2) and KEGG pathways with FDR < 0.05 were considered as significantly enriched pathways. Gene expression heatmaps were generated using the R platform (v4.0.2).

Lipidomics analysis of *S. angustifolia* under drought stress

Lipidomics analysis was conducted on *S. angustifolia* leaf samples from the drought treatment group and the control group at Biotree Biomedical Technology Co., Ltd. (Shanghai, China). Sample extraction was performed with slight modifications to our previously reported method [79]. Briefly, freeze-dried samples were extracted using the flowing extracting solution: MTBE: MeOH= 5:1 (v/v) containing an isotope-labeled internal standard. Subsequently, 100 μL of the extracted supernatant was transferred to the injection bottle for lipid metabolite detection.

The chromatographic separation of the target compounds was performed using a Phenomenex Kinetex C18 column (2.1 mm × 100 mm, 2.6 μm) on a Vanquish ultra-performance liquid chromatograph (Thermo Fisher Scientific). The mobile phase A consisted of 40% water and 60% acetonitrile with 10 mmol/L ammonium formate, while phase B comprised 10% acetonitrile and 90% isopropanol, supplemented with 50 mL of 10 mmol/L ammonium formate aqueous solution per liter. The injection volume was set at 2 μL. Mass spectrometric analysis was performed on an Orbitrap Exploris 120, allowing for both primary and secondary mass spectrometry data acquisition using Xcalibur (v4.4). The operational parameters were: Sheath gas flow rate at 30 Arb, auxiliary gas flow rate at 10 Arb, capillary temperature at 320 °C (both positive and negative modes), full MS resolution at 60,000, MS/MS resolution at 15,000, collision MS resolution at 15,000, collision energy at 15/30/45 in NCE mode, and spray voltage at 3.8 kV (positive) or -3.4 kV (negative).

The mass spectrum raw data were converted to mzXML format using ProteoWizard software.

XCMS was then used to do retention time correction, peak identification, peak extraction, peak integration, and peak alignment. The minimum fraction (minfrac) was set to 0.5 and cutoff was set to 0.3. Lipid identification was performed through a spectral match using the LipidBlast library within the XCMS software [80].

MetaboAnalyst (v6.0) [81] was adopted for lipids analyses. Lipids showing a fold change of ≥ 2 or ≤ 0.5 in relative abundance with or without drought treatment, with variable importance for projection (VIP) > 1 and an adjusted *P*-value (*P*adj) < 0.05, were identified as differentially accumulated lipids (DALs). Each treatment had three biological replicates.

Determination of ABA, JA and JA-Ile, genistein, daidzein, and raffinose

Quantitative assays of three plant hormones (ABA, JA, and JA-Ile) in the roots and leaves of *S. angustifolia* were conducted at Biotree Biomedical Technology Co., Ltd. (Shanghai, China). Weighed 100 mg of the freeze-dried samples and extracted them with 1 mL of ice-cold 50% acetonitrile (ACN) aqueous solution. Sonicate the sample at 4°C for 3 minutes, followed by extraction at 4°C for 30 minutes. Centrifuge at 12,000 rpm for 10 minutes at 4°C and collect the supernatant. Pass the sample through an RP-SPE column: Add 1 mL of 100% methanol (MeOH) and 1 mL of deionized water, then equilibrate the column with 50% ACN aqueous solution. Load the sample, wash the column with 1 mL of 30% ACN, and collect the eluent. Evaporate the sample to dryness under a nitrogen stream, dissolve it in 200 μL of 30% ACN, and transfer it to a sample vial with an insert. The data acquisition system mainly consisted of ultra-high-performance liquid chromatography (UPLC, Vanquish, Thermo, USA) and a high-resolution mass spectrometer (Q Exactive, Thermo, USA). The liquid chromatography parameters are set as follows: Chromatographic column: Waters HSS T3 (50 × 2.1 mm, 1.8 μm); mobile phase: Phase A was ultrapure water (containing 0.1% acetic acid), Phase B was acetonitrile (containing 0.1% acetic acid); flow rate: 0.3 mL/min; column temperature: 40°C; injection volume: 2 μL; elution gradient: 0 min water/acetonitrile (90:10, v/v), 1 min water/acetonitrile (90:10, v/v), 5 min water/acetonitrile (10:90, v/v), 7 min water/acetonitrile (10:90, v/v), 7.1 min water/acetonitrile (90:10, v/v), 9 min water/acetonitrile (90:10, v/v). During the entire analysis, samples were kept in an auto-sampler at 4°C. To avoid signal fluctuation impacts, samples were analyzed in random sequence. QC samples

were inserted into the sample queue to monitor and evaluate system stability and data reliability. Data acquisition was performed using the Q Exactive high-resolution mass spectrometer (Thermo Fisher Scientific, USA). Electrospray ionization (ESI) conditions were as follows: sheath gas 40 arb; auxiliary gas 10 arb; spray voltage 3000V; temperature 350°C; ion transfer tube temperature 320°C. The scan mode was single ion monitoring (SIM) in positive ion mode. The primary scan m/z range was 100-500. Mass spectrometry data were processed using TraceFinder software.

Quantitative assays of raffinose in the leaves of *S. angustifolia* were conducted by Biotech-pack-analytical Inc. (Beijing, China). Weighed 300 mg of freeze-dried samples and extracted them with 5 mL of 80% ethanol at 85°C twice, each time for 30 minutes. Following each extraction, the mixture was centrifuged at 12,000 rpm for 5 minutes. The combined supernatants were collected and evaporated to dryness using a vacuum centrifuge. The resulting dried residue was resuspended in 300 μL of distilled water and centrifuged again at 12,000 rpm for 10 minutes. The final supernatant was collected for high-performance liquid chromatograph (HPLC) analysis. The liquid chromatography analysis was performed using a Waters 2695 HPLC coupled with a Waters 2424 evaporative light-scattering detector (ELSD). The chromatographic conditions were as follows: column temperature: 40 °C; flow rate: 1.0 mL/min; injection volume: 3 μL of sample; chromatographic column: Sepax HP-Amino (4.6 x 250 mm, 5 μm, 120 Å); mobile phase: acetonitrile: water (70:30) with isocratic elution; total run time: 20 minutes.

Quantitative assays of genistein and daidzein in the leaves of *S. angustifolia* were conducted as described in our previous report [79].

Figure legends

Figure 1. Genomic features of *S. angustifolia*. (a). Features of assembled *S. angustifolia* genome. From 1 to 6: chromosomes, repeat element density, gene density, non-coding RNA density, GC content, and intraspecific collinearity between chromosomes. The contents of 2 to 5 were calculated using a non-overlapping window size of 500 kilobases (kb) **(b).** Hi-C interactions among ten chromosomes of the *S. angustifolia* genome. Dark red indicates strong interactions and yellow indicates weak interactions.

Figure 2. Comparative genomic analyses of *S. angustifolia* and other plant species. (a)

Orthologous groups (OGs) and shared OGs of studied Fabaceae species and Arabidopsis. The red circle represents *S. angustifolia* specific OGs. **(b)** Phylogenetic trees and divergence time analysis of the studied species based on single-copy OGs. **(c)** Genomic synteny comparisons between *S. angustifolia*, *A. duranensis*, and *A. ipaensis*. **(d)** Ks distribution of collinear gene pairs within and between *S. angustifolia*, *A. duranensis*, *A. ipaensis*, and *G. max*.

Figure 3. Transcriptome analysis and genome evolution of *S. angustifolia* in adaptation to drought stress. (a) Morphological characteristics of *S. angustifolia* with or without drought treatments. **(b)** The number of differentially expressed genes (DEGs) in *S. angustifolia* under drought versus control conditions. **(c)** Intersection analysis of genome-expanded genes and DEGs in the roots of *S. angustifolia* in response to drought stress. **(d)** Intersection analysis of genome-expanded genes and DEGs in the leaves of *S. angustifolia* in response to drought stress. **(e)** KEGG pathway enrichment analysis of genome-expanded genes up-regulated by drought stress in *S. angustifolia* roots and leaves.

Figure 4. **The evolution and expansion of *xanthoxin dehydrogenase* (*ABA2*) genes in the *S. angustifolia* genome and their response to drought stress. (a)** Phylogenetic tree of *ABA2* genes in *S. angustifolia*, *G. max*, *M. truncatula*, and *A. thaliana*. The heatmap of ABA2 expression in *S. angustifolia* under drought versus control conditions. Red arrows indicate genes up-regulated by drought stress, while blue arrows indicate genes down-regulated by drought stress. **(b)** Microcollinearity of *ABA2* genes in *S. angustifolia* compared with *G. max* and *M. truncatula*. Red curves represent the correspondence of *ABA2* genes across different species. **(c)** The divergence time of *ABA2* genes occurred by tandem duplication. **(d)** Quantification of ABA contents in *S. angustifolia* roots. **(e)** Quantification of ABA contents in *S. angustifolia* leaves.

Figure 5. **The evolution and expansion of *2-hydroxyisoflavanone dehydratase* (*HIDH*) genes in the *S. angustifolia* genome and their response to drought stress. (a)** Phylogenetic tree of *HIDH* genes in *S. angustifolia*, *G. max*, and *M. truncatula*. The heatmap of *HIDH* expression in *S. angustifolia* under drought versus control conditions. Red arrows indicate genes up-regulated by drought stress, while blue arrows indicate genes down-regulated by drought stress. **(b)** Microcollinearity of *HIDH* genes in *S. angustifolia* compared with *G. max* and *M. truncatula*. Red curves represent the correspondence of *HIDH* genes across different species. **(c)** Quantification of

genistein contents in *S. angustifolia* leaves. **(d)** Quantification of daidzein contents in *S. angustifolia* leaves.

Figure 6. **Analysis of tandem duplicated genes (TDGs) involved in lipid metabolism in *S. angustifolia* under drought stress. (a)** Ks distribution of TDGs in *S. angustifolia*, *A. duranensis*, *A. ipaensis*, *G. max*, and *M. truncatula*. **(b)** KEGG pathway enrichment analysis of TDGs in *S. angustifolia*. **(c)** Intersection analysis of TDGs and DEGs in the leaves of *S. angustifolia* in response to drought stress. **(e)** Differentially accumulated lipids (DALs) belonging to glycerophospholipids, galactolipids, and sulfolipids in the leaves of *S. angustifolia* under drought conditions compared to control conditions.

Figure 7. **Genes involved in the biosynthesis pathway of jasmonic acid (JA) and jasmonoyl-isoleucine (JA-Ile) in response to drought stress in *S. angustifolia* leaves.**

Figure 8. **Genes involved in the raffinose biosynthesis pathway in response to drought stress in *S. angustifolia* leaves. (a)** Expression heatmap of genes involved in the raffinose biosynthesis pathway. **(b)** Quantification of raffinose contents in *S. angustifolia* leaves. **(c)** Microcollinearity of *raffinose synthase* (*RAFS*) genes in *S. angustifolia* compared with *G. max* and *M. truncatula*. Red curves represent the correspondence of *RAFS* genes across different species.

Additional Files

Fig. S1. Morphological characteristics of *S. angustifolia*.

Fig. S2. Genomic survey analysis of *S. angustifolia*.

Fig. S3. Gene family expansion and contraction analyses of seven studied plant species.

Fig. S4. KEGG classification of *S. angustifolia* expanded OGs.

Fig. S5. Volcano plot of differentially expressed genes (DEGs) in the roots and leaves of *S. angustifolia* under drought stress conditions.

Fig. S6. Volcano plot of differentially accumulated lipids (DALs) in the leaves of *S. angustifolia* under drought stress conditions.

Fig. S7. Microcollinearity of *Patatin-related phospholipase A* (*pPLA*) genes in *S. angustifolia*, soybean, and barrel medic.

Table S1. Statistics of nanopore (ONT) sequencing data of *S. angustifolia*.

Table S2. Statistics of next-generation sequencing (NGS) data of *S. angustifolia*.

Table S3. Statistics of Hi-C sequencing data of *S. angustifolia*.

Table S4. Identification of telomeres in the assembled *S. angustifolia* genome.

Table S5. BUSCO assessment of assembled genome.

Table S6. Statistics of repetitive sequence in the assembled genome.

Table S7. Classification of repetitive sequence in the assembled genome.

Table S8. Statistics of RNA-seq data from different tissues.

Table S9. BUSCO assessment of predicted gene set.

Table S10. Functional annotation of the predicted genes.

Table S11. Statistics of non-coding RNAs in the assembled genome.

Table S12. Statistics of RNA-seq data with or without drought treatments.

Table S13. KEGG pathway enrichment analysis of the *S. angustifolia* expanded genes that were up-regulated in roots under drought stress.

Table S14. KEGG pathway enrichment analysis of the *S. angustifolia* expanded genes that were up-regulated in leaves under drought stress.

Table S15. Identification of *ABA2* (K09841) genes in *S. angustifolia*, soybean, barrel medic, and Arabidopsis based on the KEGG database.

Table S16. Calculation of divergence time of the tandem duplicated *ABA2* genes in the *S. angustifolia* genome.

Table S17. Identification of *HIDH* (K13258) genes in *S. angustifolia*, soybean, and barrel medic based on the KEGG database.

Table S18. Calculation of divergence time of the tandem duplicated *HIDH* genes in the *S. angustifolia* genome.

Table S19. Identification of lipids in *S. angustifolia* leaves with or without drought treatment.

Table S20. Identification of *patatin-related phospholipase A* (*pPLA*) genes in the *S. angustifolia* genome and its regulation under drought stress.

Table S21. Identification of genes involved in lipid degradation and its regulation under drought stress.

Table S22. Identification of *jasmonate resistant 1* (*JAR1*) genes in the *S. angustifolia* genome and its regulation under drought stress.

Table S23. Regulation of genes involved in phosphatidylinositol degradation under drought stress.

Abbreviations

T2T: telomere-to-telomere; Gb: gigabase; Mb: megabase; BUSCO: Benchmarking Universal Single-Copy Orthologs; WGD: whole-genome duplication events; TD: tandem duplication; ABA2: xanthoxin dehydrogenase; HIDH: 2-hydroxyisoflavanone dehydratase; pPLA: patatin-related phospholipase A; RAFS: raffinose synthases; ABA: abscisic acid; JA: jasmonic acid; JA-Ile: jasmonoyl-isoleucine; TGS: third-generation sequencing; PacBio: Pacific Biosciences; ONT: Oxford Nanopore Technologies; NGS next-generation sequencing; TDG: tandem duplicated gene; Hi-C: high-through chromosome conformation capture; LTR: long terminal repeat; LAI: LTR assembly index; LINE: long interspersed nuclear element; KEGG: Kyoto Encyclopedia of Genes and Genomes GO: Gene Ontology; ncRNA: non-coding RNA; MYA: million years ago; OG: orthologous group; DEG: differentially expressed gene; DAL: differentially accumulated lipids; LOX: lipoxygenase; AOS: allene oxide synthase; AOC: allene oxide cyclase; OPR: 12-oxophytodienoate reductase, OPCL1: OPC-8: CoA ligase 1; ACS: acyl-CoA oxidase;ACAA1: acetyl-CoA acyltransferase 1, MFP2: multifunctional protein 2; JAR1: jasmonate resistant 1; AMY3: α-amylase; CWINV1: β-fructofuranosidase; 3D-DNA: 3D de novo assembly; EDTA: Extensive de novo TE Annotator; TE: transposable element; UTR: untranslated region; FDR: false discovery rate; VIP: variable importance for projection.

Data Availability

The raw genomic sequencing data, including ONT, NGS, and Hi-C data, as well as transcriptome data, have been deposited in the National Genomics Data Center (NGDC, https://ngdc.cncb.ac.cn) under BioProject PRJCA027610. The raw sequence data have been deposited in the Genome Sequence Archive in the National Genomics Data Center, China National Center for Bioinformation / Beijing Institute of Genomics, Chinese Academy of Sciences (GSA: CRA017744) that are publicly accessible at https://ngdc.cncb.ac.cn/gsa. The assembly and annotation of *S. angustifolia* have been

deposited in the Genome Warehouse in the National Genomics Data Center under accession number GWHEUEA00000000.1, which is publicly accessible at https://ngdc.cncb.ac.cn/gwh.

Competing Interests

The authors declare that they have no competing interests.

Author Contributions

P.L. conceived the project and designed the experiments. C.L. performed genome assembly, annotation, transcriptome, and lipidome analyses. G.L. provided funding and *S. angustifolia* germplasm. L.L. performed supervision. J. Z., R. X., and J. L. planted and collected samples. S.Z., M.B., and Z.Q. provided technical support and suggestions on manuscript revision. C.L. performed the manuscript. P.L. revised the manuscript. All authors read and approved the final manuscript.

Funding

The research was financially supported by the Natural Science Foundation of Hainan Province (323CXTD387), the National Natural Science Foundation of China (32371769), the earmarked fund for China Agriculture Research System (CARS-34 and CARS-22), the Agricultural Research Outstanding Talents and Innovation Team of MARA (No. 13210268), the Central Public-interest Scientific Institution Basal Research Fund for CATAS (No. 1630032022023), and the Guangxi Special Project for Innovation-driven Development (Guike AA18242040).

References

1. Gupta A, Rico-Medina A, Caño-Delgado AI. The physiology of plant responses to drought. Science 2020;368:266–269. https://doi.org/10.1126/science.aaz7614.

2. Vadez V, Grondin A, Chenu K, et al. Crop traits and production under drought. Nat Rev Earth Environ 2024;5:211–225. https://doi.org/10.1038/s43017-023-00514-w.

3. Wang X, Li Q, Xie J, et al. Abscisic acid and jasmonic acid are involved in drought priming-induced tolerance to drought in wheat. Crop Journal 2021;9:120–132. https://doi.org/10.1016/j.cj.2020.06.002.

4. Yang W, Li N, Fan Y, et al. Transcriptome analysis reveals abscisic acid enhancing drought resistance by regulating genes related to flavonoid metabolism in pigeon pea. Environ Exp Bot 2021;191:104627. https://doi.org/10.1016/j.envexpbot.2021.104627.

5. Wen D, Zheng Y, Han Y, et al. Sodium selenite increases drought tolerance by promoting jasmonic acid biosynthesis in cucumber. HORTIC ADV 2023;1,6. https://doi.org/10.1007/s44281-023-00009-0.

6. Liu Y, Li T, Zhang C, et al. Raffinose positively regulates maize drought tolerance by reducing leaf transpiration. Plant J 2023;114(1):55–67. https://doi.org/10.1111/tpj.16116.

7. Hu H, Xiong L. Genetic engineering and breeding of drought-resistant crops. Annu Rev Plant Biol 2014;65:715–41. https://doi.org/10.1146/annurev-arplant-050213-040000.

8. Yang Z, Qin F. The battle of crops against drought: Genetic dissection and improvement. J Integr Plant Biol 2023;65:496–525. https://doi.org/10.1111/jipb.13451.

9. He Z, Zhang P, Jia H, et al. Regulatory mechanisms and breeding strategies for crop drought resistance. New Crops 2024;1:2949–9526. https://doi.org/10.1016/J.NCROPS.2024.100029.

10. Marques A, Moraes L, Dos Santos MA, et al. Origin and parental genome characterization of the allotetraploid *stylosanthes scabra* vogel (Papilionoideae, Leguminosae), an important legume pasture crop. Ann Bot 2018;122:1143–1159. https://doi.org/10.1093/aob/mcy113.

11. Luo J, Chen Z, Huang R, et al. Multi-omics analysis reveals the roles of purple acid phosphatases in organic phosphorus utilization by the tropical legume *Stylosanthes guianensis*. Plant J 2024 117:729–746; https://doi.org/10.1111/tpj.16526.

12. Song J, Zou X, Liu P, et al. Differential expressions and enzymatic properties of malate dehydrogenases in response to nutrient and metal stresses in *Stylosanthes guianensis*. Plant Physiol Biochem 2022;170:325–337. https://doi.org/10.1016/j.plaphy.2021.12.012.

13. Siqueira JA, Wakin T, Batista-Silva W, et al. A long and stressful day: Photoperiod shapes aluminium tolerance in plants. J Hazard Mater 2022;432:128704. https://doi.org/10.1016/j.jhazmat.2022.128704.

14. Sun L, Liang C, Chen Z, et al. Superior aluminium (Al) tolerance of stylosanthes is achieved mainly by malate synthesis through an Al-enhanced malic enzyme, SgME1. New Phytol 2014;202:209–219. https://doi.org/10.1111/nph.12629.

15. Jia Y, Li X, Liu Q, et al. Physiological and transcriptomic analyses reveal the roles of secondary metabolism in the adaptive responses of Stylosanthes to manganese toxicity. BMC Genomics 2020;21:861. https://doi.org/10.1186/s12864-020-07279-2.

16. Liu P, Huang R, Hu X, et al. Physiological responses and proteomic changes reveal insights into Stylosanthes response to manganese toxicity. BMC Plant Biol 2019;19:212. https://doi.org/10.1186/s12870-019-1822-y.

17. Ribeiro RP, Costa LC, Medina EF, et al. Ethylene coordinates seed germination behavior in response to low soil pH in *Stylosanthes humilis*. Plant Soil 2018;425:87–100. https://doi.org/10.1007/s11104-018-3572-2.

18. Habermann E, Dias de Oliveira EA, Delvecchio G, et al. How does leaf physiological acclimation impact forage production and quality of a warmed managed pasture of *Stylosanthes capitata* under different conditions of soil water availability? Sci Total Environ 2021;759:143505. https://doi.org/10.1016/j.scitotenv.2020.143505.

19. Schultze-Kraft R, Hubiao Y, Jun T, et al. *Stylosanthes guianensis* CIAT 184 – review of a tropical forage legume. Tropical Grasslands-Forrajes Tropicales 2023:11:95–120. https://doi.org/10.17138/tgft(11)95-120.

20. Ma ZY, Chandra A, Musial JM, et al. Molecular evidence that *Stylosanthes angustifolia* is the third putative diploid progenitor of the hexaploid *S. erecta* (Fabaceae). Plant Syst Evol 2004;248:171–176. https://doi.org/10.1007/s00606-004-0179-2.

21. SCHULTZE-KRAFT R, GIACOMETTI DC. 1979. Genetic resources of forage legumes for the acid, infertile savannas of Tropical America. In: Sanchez, P.A.; Tergas, L. E. (eds.). Seminar on Pasture Production in Acid Soils of the Tropics (1978, Cali, Colombia). Pasture production in acid soils of the tropics: Proceedings. Centro Internacional de Agricultura Tropical (CIAT), Beef Program, Cali, CO. p. 55-64.

22. Nurk S, Koren S, Rhie A, et al. The complete sequence of a human genome. Science 2022;376:44–53. https://doi.org/10.1126/science.abj6987.

23. He Y, Chu Y, Guo S, et al. T2T-YAO: A Telomere-to-telomere Assembled Diploid Reference Genome for Han Chinese. Genomics Proteomics Bioinformatics 2023;21:1085–1100. https://doi.org/10.1016/j.gpb.2023.08.001.

24. Wenger AM, Peluso P, Rowell WJ, et al. Accurate circular consensus long-read sequencing improves variant detection and assembly of a human genome. Nat Biotechnol. 2019;37:1155–1162. https://doi.org/10.1038/s41587-019-0217-9.

25. Jain M, Koren S, Miga KH, et al. Nanopore sequencing and assembly of a human genome with ultra-long reads. Nat Biotechnol 2018;36:338–345. https://doi.org/10.1038/nbt.4060.

26. Kong W, Wang Y, Zhang S, et al. Recent Advances in Assembly of Complex Plant Genomes. Genomics Proteomics Bioinformatics 2023;21:427–439. https://doi.org/10.1016/j.gpb.2023.04.004.

27. Hou X, Wang D, Cheng Z, et al. A near-complete assembly of an *Arabidopsis thaliana* genome. Mol Plant 2022;15:247–1250. https://doi.org/10.1016/j.molp.2022.05.014.

28. Naish M, Alonge M, Wlodzimierz P, et al. The genetic and epigenetic landscape of the *Arabidopsis* centromeres. Science 2021;374:eabi7489. https://doi.org/10.1126/science.abi7489.

29. Song JM, Xie WZ, Wang S, et al. Two gap-free reference genomes and a global view of the centromere architecture in rice. Mol Plant 2021;14:1757–1767. https://doi.org/10.1016/j.molp.2021.06.018.

30. Shang L, He W, Wang T, et al. A complete assembly of the rice Nipponbare reference genome. Mol Plant 2023;16:1232–1236. https://doi.org/10.1016/j.molp.2023.08.003.

31. Chen J, Wang Z, Tan K, et al. A complete telomere-to-telomere assembly of the maize genome. Nat Genet 2023;55:1221–1231. https://doi.org/10.1038/s41588-023-01419-6.

32. Zhang C, Xie L, Yu H, et al. The T2T genome assembly of soybean cultivar ZH13 and its epigenetic landscapes. Mol Plant 2023;16:1715–1718. https://doi.org/10.1016/j.molp.2023.10.003.

33. Wang L, Zhang M, Li M, et al. A telomere-to-telomere gap-free assembly of soybean genome. Mol Plant 2023;16:1711–1714. https://doi.org/10.1016/j.molp.2023.08.012.

34. Shi X, Cao S, Wang X, et al. The complete reference genome for grapevine (*Vitis vinifera* L.) genetics and breeding. Hortic Res 2023;10:uhad061. https://doi.org/10.1093/hr/uhad061.

35. Ferreira-Neto JRC, da Silva MD, Binneck E, et al. Bridging the Gap: Combining Genomics and Transcriptomics Approaches to Understand *Stylosanthes scabra*, an Orphan Legume from the Brazilian Caatinga. Plants. 2023;12:3246. https://doi.org/10.3390/plants12183246.

36. Luo H, Wang X, You C, et al. Telomere-to-telomere genome of the allotetraploid legume Sesbania cannabina reveals transposon-driven subgenome divergence and mechanisms of alkaline

stress tolerance. Sci China Life Sci 2024;67:149–160. https://doi.org/10.1007/s11427-023-2463-y.

37. Nei M. Gene duplication and nucleotide substitution in evolution. Nature 1969;221:40–2. https://doi.org/10.1038/221040a0.

38. Long M, Betrán E, Thornton K, et al. The origin of new genes: Glimpses from the young and old. Nat Rev Genet 2003;4:865–75. https://doi.org/10.1038/nrg1204.

39. Innan H, Kondrashov F. The evolution of gene duplications: Classifying and distinguishing between models. Nat Rev Genet 2010;11:97–108. https://doi.org/10.1038/nrg2689.

40. Liu C, Wu Y, Liu Y, et al. Genome-wide analysis of tandem duplicated genes and their contribution to stress resistance in pigeonpea (*Cajanus cajan*). Genomics 2021;113:728–735. https:doi.org/10.1016/j.ygeno.2020.10.003.

41. Liu C, Tai Y, Luo J, et al. Integrated multi-omics analysis provides insights into genome evolution and phosphorus deficiency adaptation in pigeonpea (*Cajanus cajan*). Hortic Res 2022;9:uhac107. https://doi.org/10.1093/hr/uhac107.

42. Qu J, Liu L, Guo Z, et al. The ubiquitous position effect, synergistic effect of recent generated tandem duplicated genes in grapevine, and their co-response and overactivity to biotic stress. Fruit Research 2023;3:16. https://doi.org/10.48130/FruRes-2023-0016.

43. Salojärvi J, Smolander OP, Nieminen K, et al. Genome sequencing and population genomic analyses provide insights into the adaptive landscape of silver birch. Nat Genet 2017;49:904–912. https://doi.org/10.1038/ng.3862.

44. Hanada K, Zou C, Lehti-Shiu MD, et al. Importance of lineage-specific expansion of plant tandem duplicates in the adaptive response to environmental stimuli. Plant Physiol 2008;148:993–1003. https://doi.org/10.1104/pp.108.122457.

45. Liu JN, Fang H, Liang Q, et al. Genomic analyses provide insights into the evolution and salinity adaptation of halophyte *Tamarix chinensis*. Gigascience 2022;12:giad053. https://doi.org/10.1093/gigascience/giad053.

46. Wang M, Yuan J, Qin L, et al. TaCYP81D5, one member in a wheat cytochrome P450 gene cluster, confers salinity tolerance via reactive oxygen species scavenging. Plant Biotechnol J 2020;18:791–804. https://doi.org/10.1111/pbi.13247.

47. Wang X, Gao Y, Wu X, et al. High-quality evergreen azalea genome reveals tandem duplication-

facilitated low-altitude adaptability and floral scent evolution. Plant Biotechnol J 2021;19:2544–2560. https://doi.org/10.1111/pbi.13680.

48. Rietz S, Dermendjiev G, Oppermann E, et al. Roles of Arabidopsis patatin-related phospholipases a in root development are related to auxin responses and phosphate deficiency. Mol Plant 2010;3:524–38. https://doi.org/10.1093/mp/ssp109.

49. Li X, Bai W, Yang Q, et al. The extremotolerant desert moss *Syntrichia caninervis* is a promising pioneer plant for colonizing extraterrestrial environments. The Innovation 2024;5:100657. https://doi.org/10.1016/J.XINN.2024.100657.

50. Lyu S, Mei Q, Liu H, et al. Genome assembly of the pioneer species Plantago major L. (Plantaginaceae) provides insight into its global distribution and adaptation to metal-contaminated soil. DNA Research 2023;30:dsad013. https://doi.org/10.1093/dnares/dsad013.

51. Lawton-Rauh A. Evolutionary dynamics of duplicated genes in plants. Mol Phylogenet Evol 2003;29:396–409. https://doi.org/10.1016/j.ympev.2003.07.004.

52. Xu Z, Pu X, Gao R, et al. Tandem gene duplications drive divergent evolution of caffeine and crocin biosynthetic pathways in plants. BMC Biol 2020;18:63. https://doi.org/10.1186/s12915-020-00795-3.

53. Cannon SB, Mitra A, Baumgarten A, et al. The roles of segmental and tandem gene duplication in the evolution of large gene families in *Arabidopsis thaliana*. BMC Plant Biol 2004;4:10. https://doi.org/10.1186/1471-2229-4-10.

54. Yin X, Yang D, Liu Y, et al. *Sophora moorcroftiana* genome analysis suggests association between sucrose metabolism and drought adaptation. Plant Physiol 2023;191:844–848. https://doi.org/10.1093/plphys/kiac558.

55. Yang Z, Nie G, Feng G, et al. Genome-wide identification of MADS-box gene family in orchardgrass and the positive role of DgMADS114 and DgMADS115 under different abiotic stress. Int J Biol Macromol 2022;223:129–142. https://doi.org/10.1016/j.ijbiomac.2022.11.027.

56. Yan H, Sun M, Zhang Z, et al. Pangenomic analysis identifies structural variation associated with heat tolerance in pearl millet. Nat Genet 2023;55:507–518. https://doi.org/10.1038/s41588-023-01302-4.

57. Herrera-Vásquez A, Salinas P, Holuigue L. Salicylic acid and reactive oxygen species interplay

in the transcriptional control of defense genes expression. Front Plant Sci 2015;6:171. https://doi.org/10.3389/fpls.2015.00171.

58. Shu S, Gao P, Li L, et al. Abscisic acid-induced H_2O_2 accumulation enhances antioxidant capacity in pumpkin-grafted cucumber leaves under $Ca(NO_3)_2$ stress. Front Plant Sci 2016;7:1489. https://doi.org/10.3389/fpls.2016.01489.

59. Kim TH, Böhmer M, Hu H, et al. Guard cell signal transduction network: Advances in understanding abscisic acid, CO_2, and Ca_2^+ signaling. Annu Rev Plant Biol 2010;61:561–91. https://doi.org/10.1146/annurev-arplant-042809-112226.

60. González-Guzmán M, Apostolova N, Bellés JM, et al. The short-chain alcohol dehydrogenase ABA2 catalyzes the conversion of xanthoxin to abscisic aldehyde. Plant Cell 2002;14:1833–46. https://doi.org/10.1105/tpc.002477.

61. Meng D, Dong B, Niu L, et al. The pigeon pea CcCIPK14-CcCBL1 pair positively modulates drought tolerance by enhancing flavonoid biosynthesis. Plant J 2021;106:1278–1297. https://doi.org/10.1111/tpj.15234.

62. Akashi T, Aoki T, Ayabe SI. Molecular and biochemical characterization of 2-hydroxyisoflavanone dehydratase. Involvement of carboxylesterase-like proteins in leguminous isoflavone biosynthesis. Plant Physiol 2005;137:882–91. https://doi.org/10.1104/pp.104.056747.

63. Song S, Qi T, Wasternack C, et al. Jasmonate signaling and crosstalk with gibberellin and ethylene. Curr Opin Plant Biol 2014;21:112–119. https://doi.org/10.1016/j.pbi.2014.07.005.

64. Gupta A, Bhardwaj M, Tran L-SP. JASMONATE ZIM-DOMAIN Family Proteins: Important Nodes in Jasmonic Acid-Abscisic Acid Crosstalk for Regulating Plant Response to Drought. Curr Protein Pept Sci 2021;22:759–766. https://doi.org/10.2174/1389203722666211018114443.

65. Mahmud S, Ullah C, Kortz A, et al. Constitutive expression of JASMONATE RESISTANT 1 induces molecular changes that prime the plants to better withstand drought. Plant Cell Environ 2022;45:2906–2922. https://doi.org/10.1111/pce.14402.

66. Staswick PE, Tiryaki I. The oxylipin signal jasmonic acid is activated by an enzyme that conjugate it to isoleucine in Arabidopsis W inside box sign. Plant Cell 2004;16:2117–27. https://doi.org/10.1105/tpc.104.023549.

67. Howe GA, Major IT, Koo AJ. Modularity in Jasmonate Signaling for Multistress Resilience.

Annu Rev Plant Biol 2018;69:387–415. https://doi.org/10.1146/annurev-arplant-042817-040047.

68. Li T, Zhang Y, Liu Y, et al. Raffinose synthase enhances drought tolerance through raffinose synthesis or galactinol hydrolysis in maize and Arabidopsis plants. J Biol Chem 2020;295:8064–8077. https://doi.org/10.1074/jbc.RA120.013948.

69. Hu J, Wang Z, Sun Z, et al. NextDenovo: an efficient error correction and accurate assembly tool for noisy long reads. Genome Biol 2024;25:107. https://doi.org/10.1186/s13059-024-03252-4.

70. Hu J, Fan J, Sun Z, et al. NextPolish: A fast and efficient genome polishing tool for long-read assembly. Bioinformatics 2020;36:2253–2255. https://doi.org/10.1093/bioinformatics/btz891.

71. Huang J, Liang X, Xuan Y, et al. LR_Gapcloser: a tiling path-based gap closer that uses long reads to complete genome assembly. Gigascience 2019;8:giy157. https://doi.org/10.1093/gigascience/giy157.

72. Ou S, Jiang N. LTR_retriever: A highly accurate and sensitive program for identification of long terminal repeat retrotransposons. Plant Physiol 2018;176:1410–1422. https://doi.org/10.1104/pp.17.01310.

73. Ou S, Su W, Liao Y, et al. Benchmarking transposable element annotation methods for creation of a streamlined, comprehensive pipeline. Genome Biol 2019;20:275. https://doi.org/10.1186/s13059-019-1905-y.

74. Pertea M, Kim D, Pertea GM, et al. Transcript-level expression analysis of RNA-seq experiments with HISAT, StringTie and Ballgown. Nat Protoc 2016;11:1650–67. https://doi.org/10.1038/nprot.2016.095.

75. Lin Y, Ye C, Li X, et al. QuarTeT: A telomere-To-Telomere toolkit for gap-free genome assembly and centromeric repeat identification. Hortic Res 2023;10:uhad127. https://doi.org/10.1093/hr/uhad127.

76. Nei M, Gojobori T. Simple methods for estimating the numbers of synonymous and nonsynonymous nucleotide substitutions. Mol Biol Evol 1986;3:418–26. https:doi/org/10.1093/oxfordjournals.molbev.a040410.

77. Bertioli DJ, Cannon SB, Froenicke L, et al. The genome sequences of *Arachis duranensis* and *Arachis ipaensis*, the diploid ancestors of cultivated peanut. Nat Genet 2016;48:438–46. https://doi.org/10.1038/ng.3517.

78. Chen Y, Chen Y, Shi C, et al. SOAPnuke: A MapReduce acceleration-supported software for integrated quality control and preprocessing of high-throughput sequencing data. Gigascience 2018;7:1–6. https://doi.org/10.1093/gigascience/gix120.

79. Liu C, Huang R, Zhao X, et al. Comparative analysis of lipid and flavonoid biosynthesis between Pongamia and soybean seeds: genomic, transcriptional, and metabolic perspectives. Biotechnol Biofuels Bioprod 2024;17:86. https://doi.org/10.1186/s13068-024-02538-w.

80. Smith CA, Want EJ, O'Maille G, et al. XCMS: Processing mass spectrometry data for metabolite profiling using nonlinear peak alignment, matching, and identification. Anal Chem 2006;78:779–87. https://doi.org/10.1021/ac051437y.

81. Pang Z, Lu Y, Zhou G, et al. MetaboAnalyst 6.0: towards a unified platform for metabolomics data processing, analysis and interpretation. Nucleic Acids Res 2024;52:W398–W406. https://doi.org/10.1093/nar/gkae253.

Figure 1

Click here to access/download;Figure;Figure 1.pdf

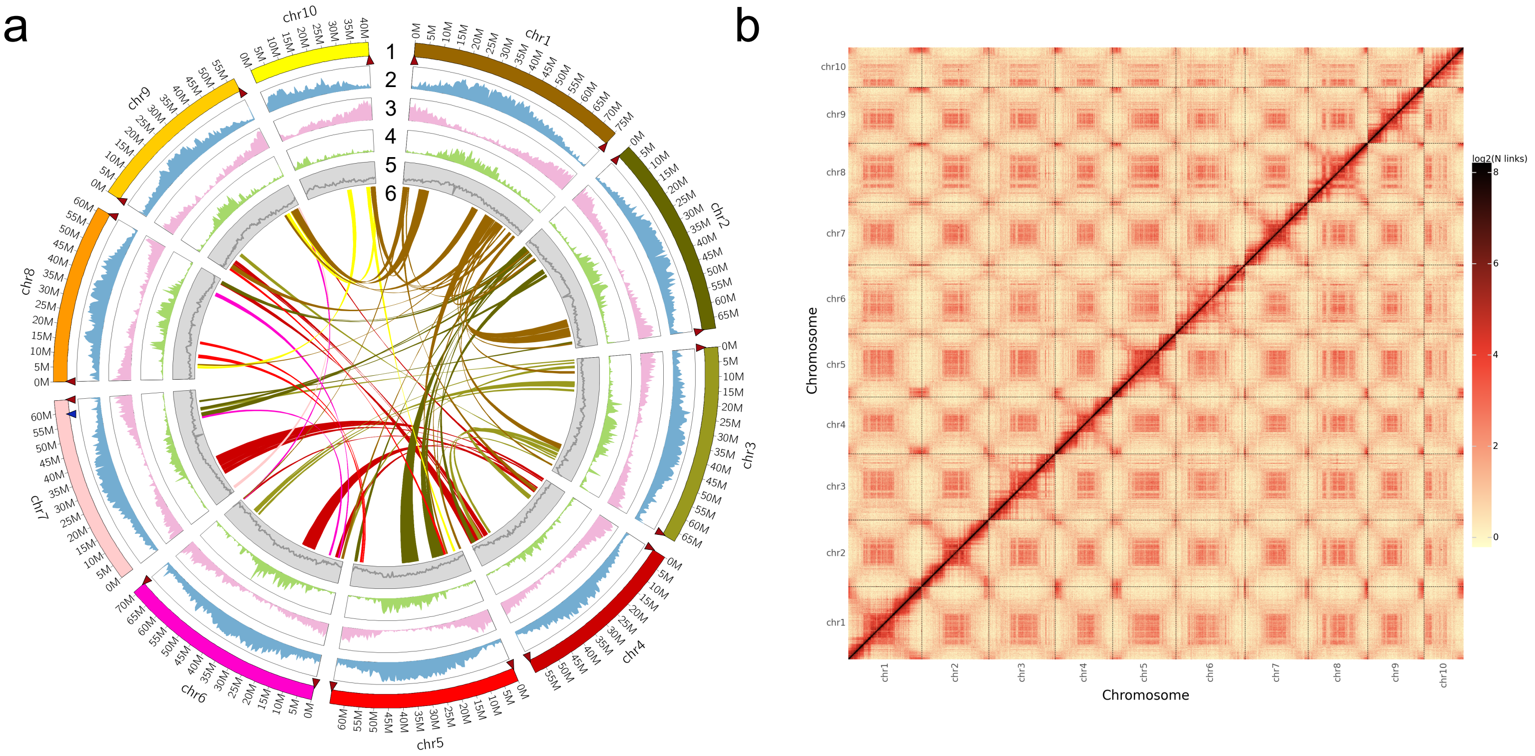

Figure 2

Click here to access/download;Figure;Figure 2.pdf

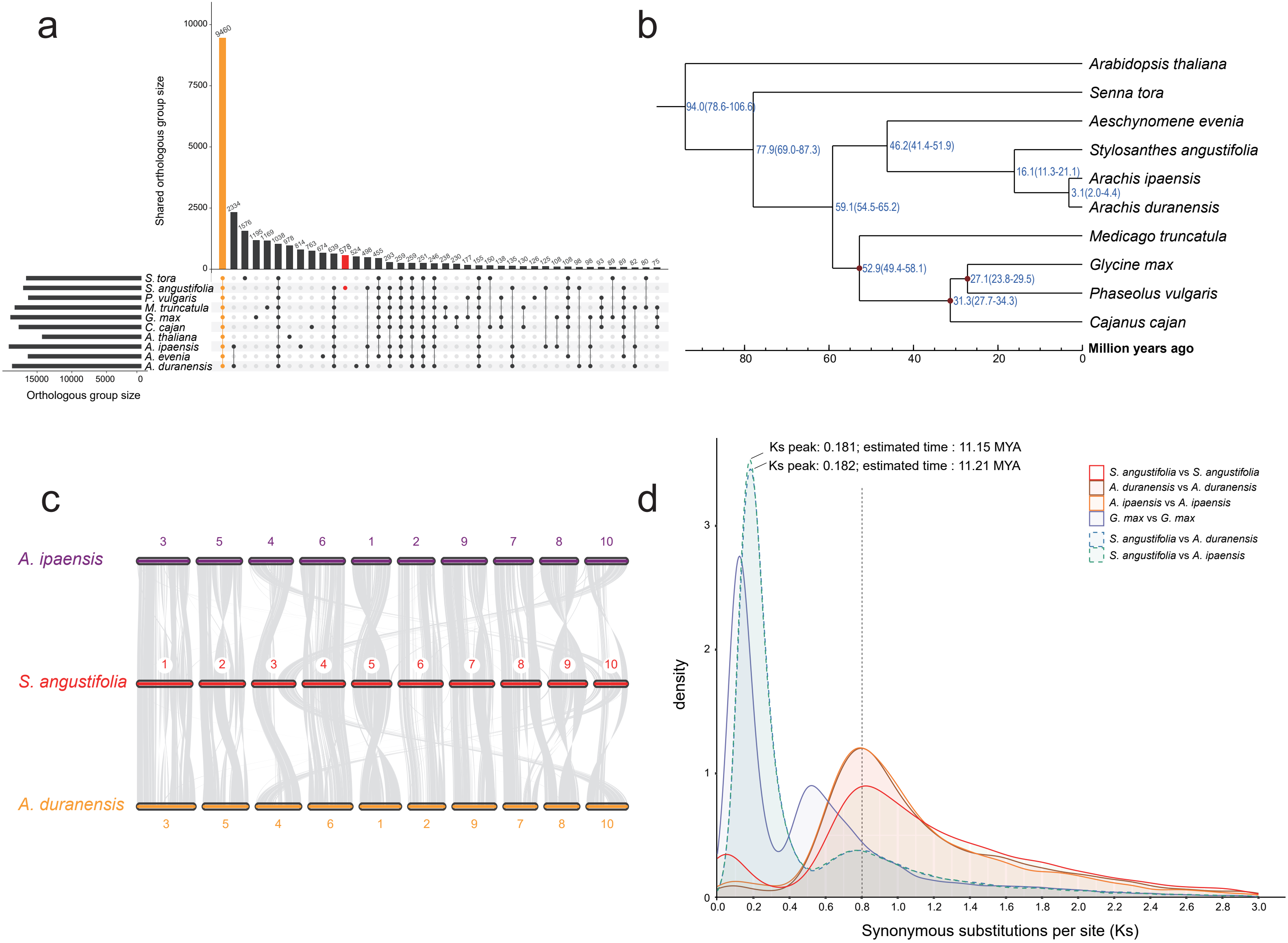

Figure 3

Click here to access/download;Figure;Figure 3.pdf

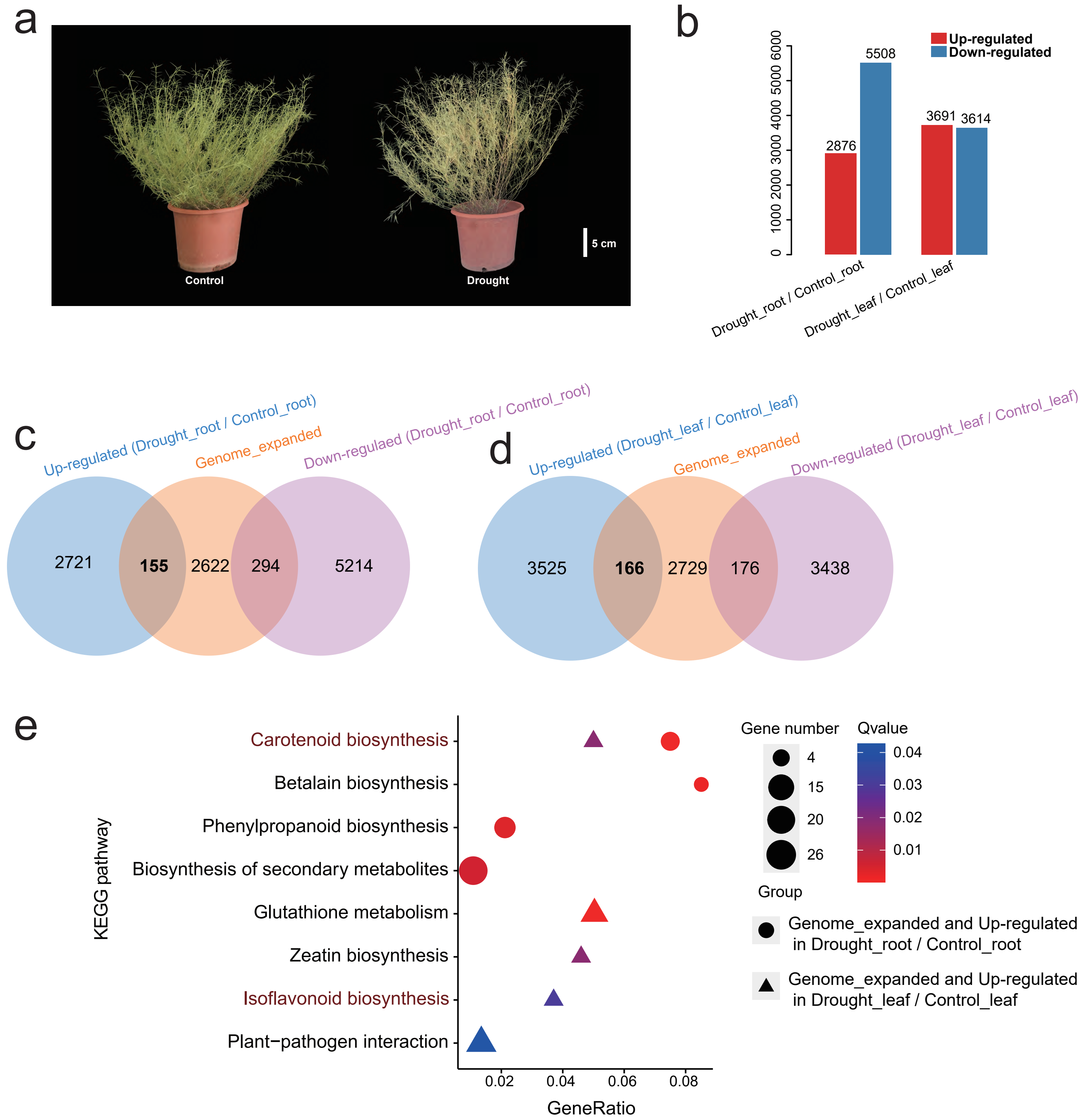

Figure 4

Click here to access/download;Figure;Figure 4.pdf

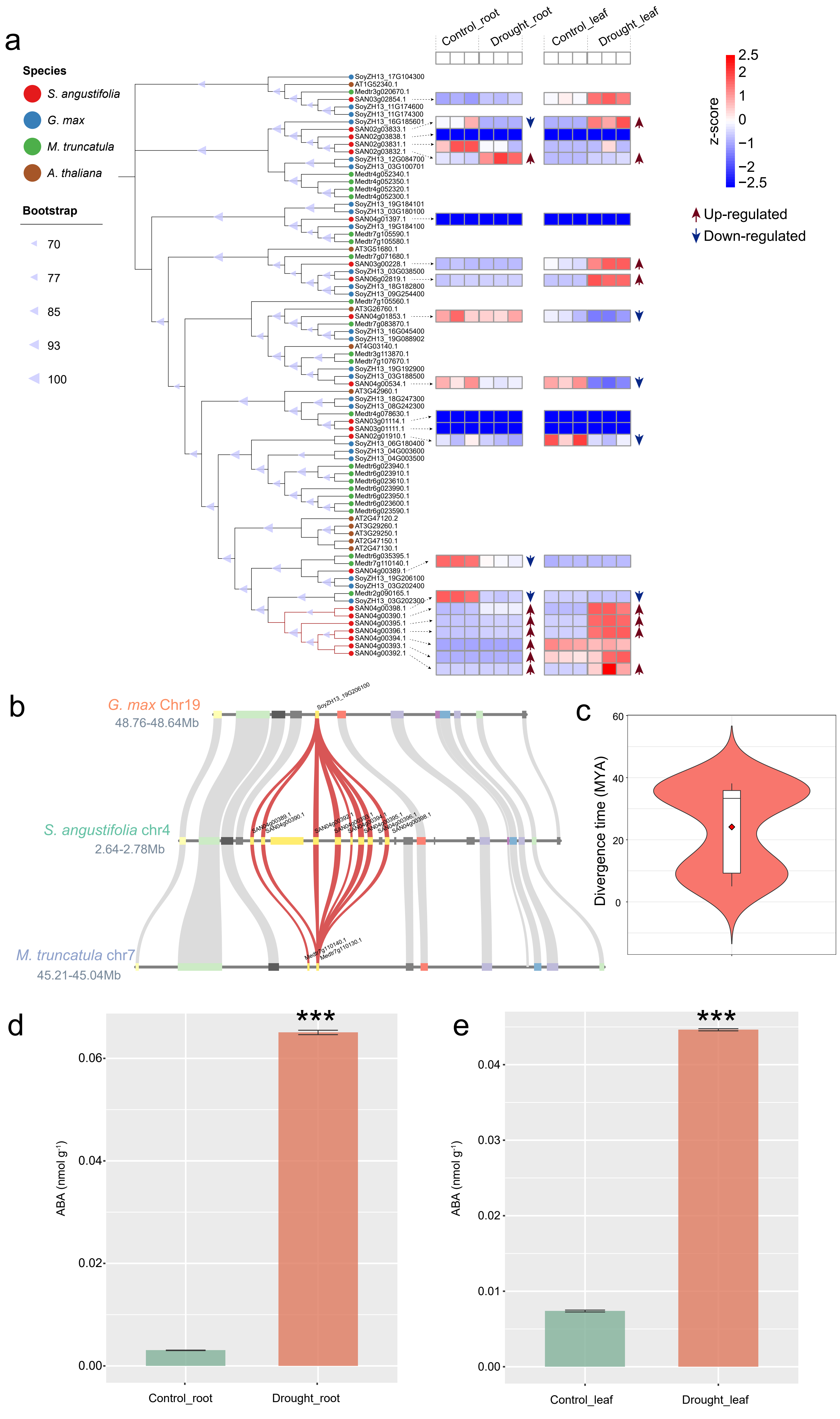

Figure 5

Click here to access/download;Figure;Figure 5.pdf

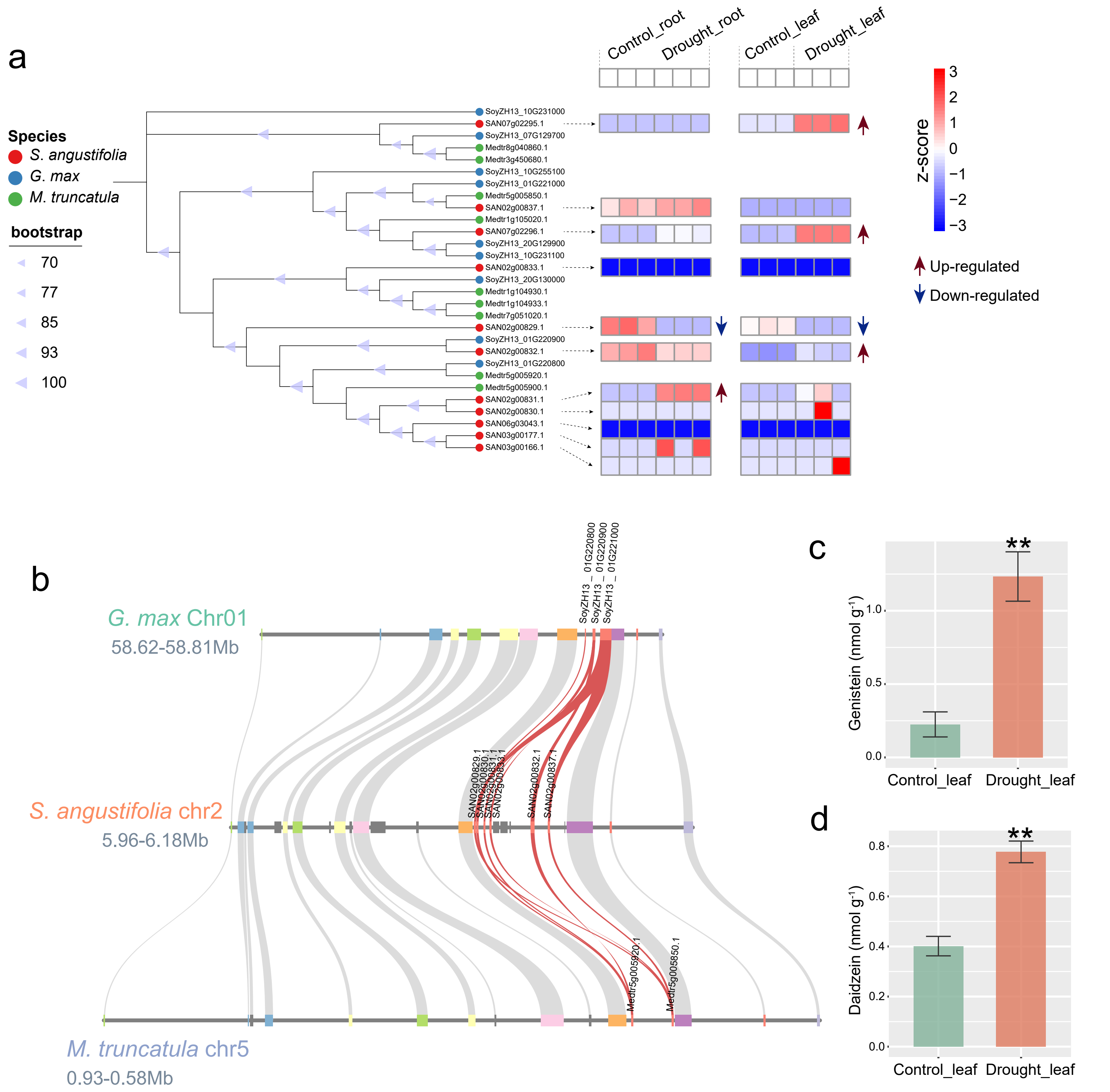

Figure 6

Click here to access/download;Figure;Figure 6.pdf

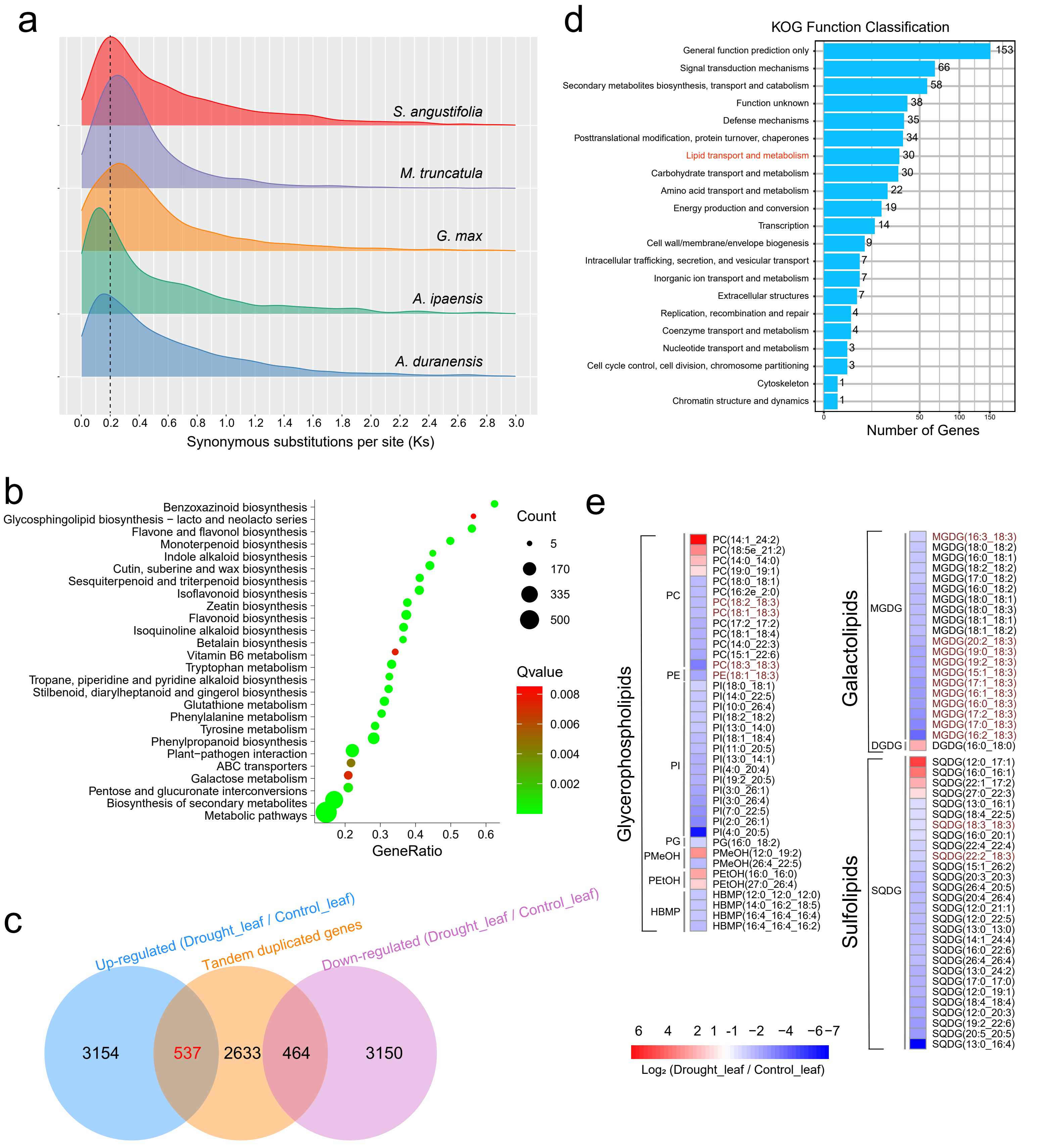

Figure 7 Click here to access/download;Figure;Figure 7.pdf

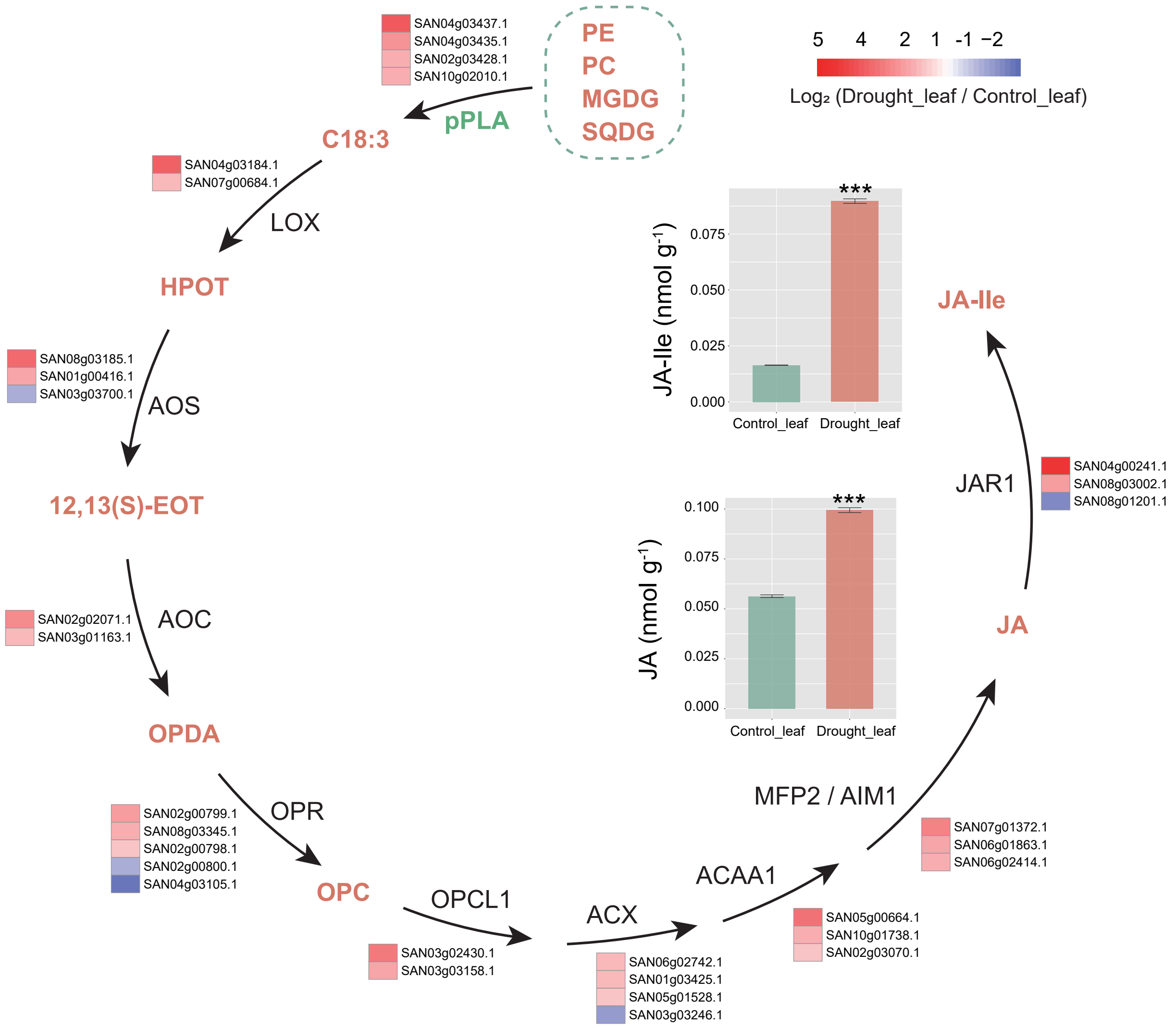

Figure 8

Click here to access/download;Figure;Figure 8.pdf

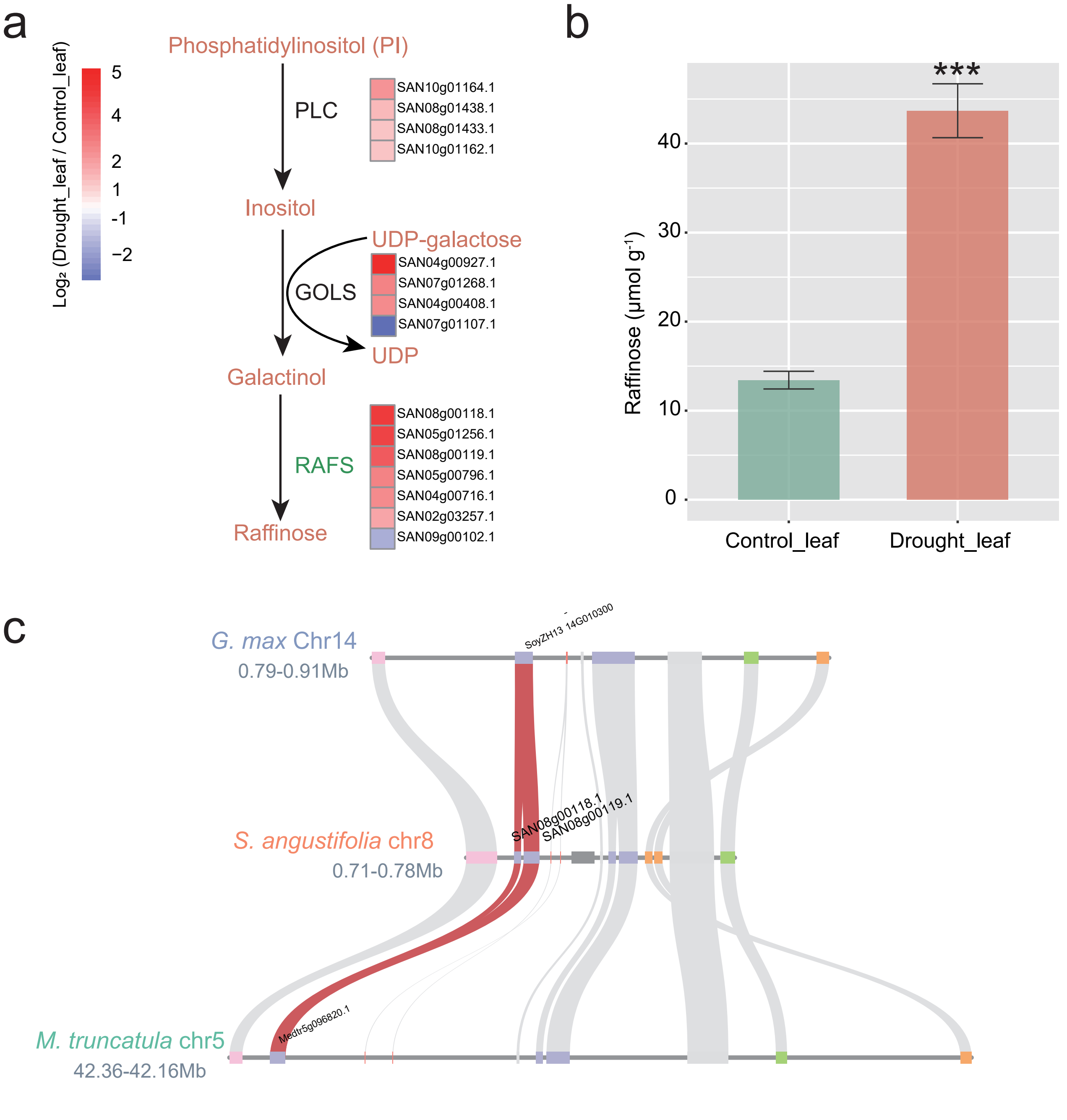

Figure S1

Click here to access/download;Figure;Figure S1.pdf

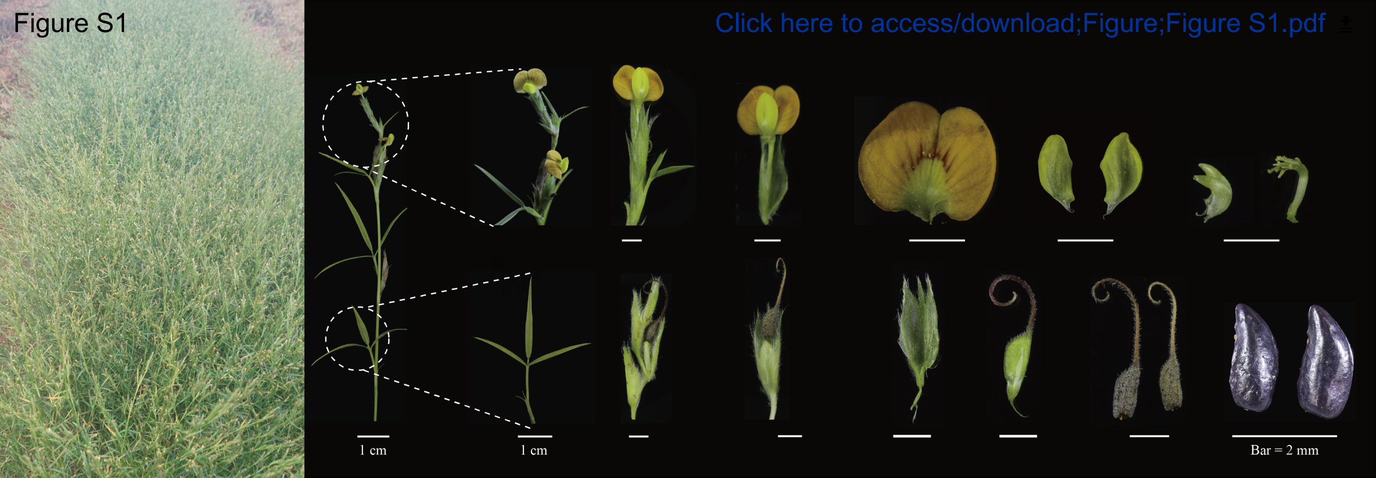

Figure S2

Click here to access/download;Figure;Figure S2.pdf

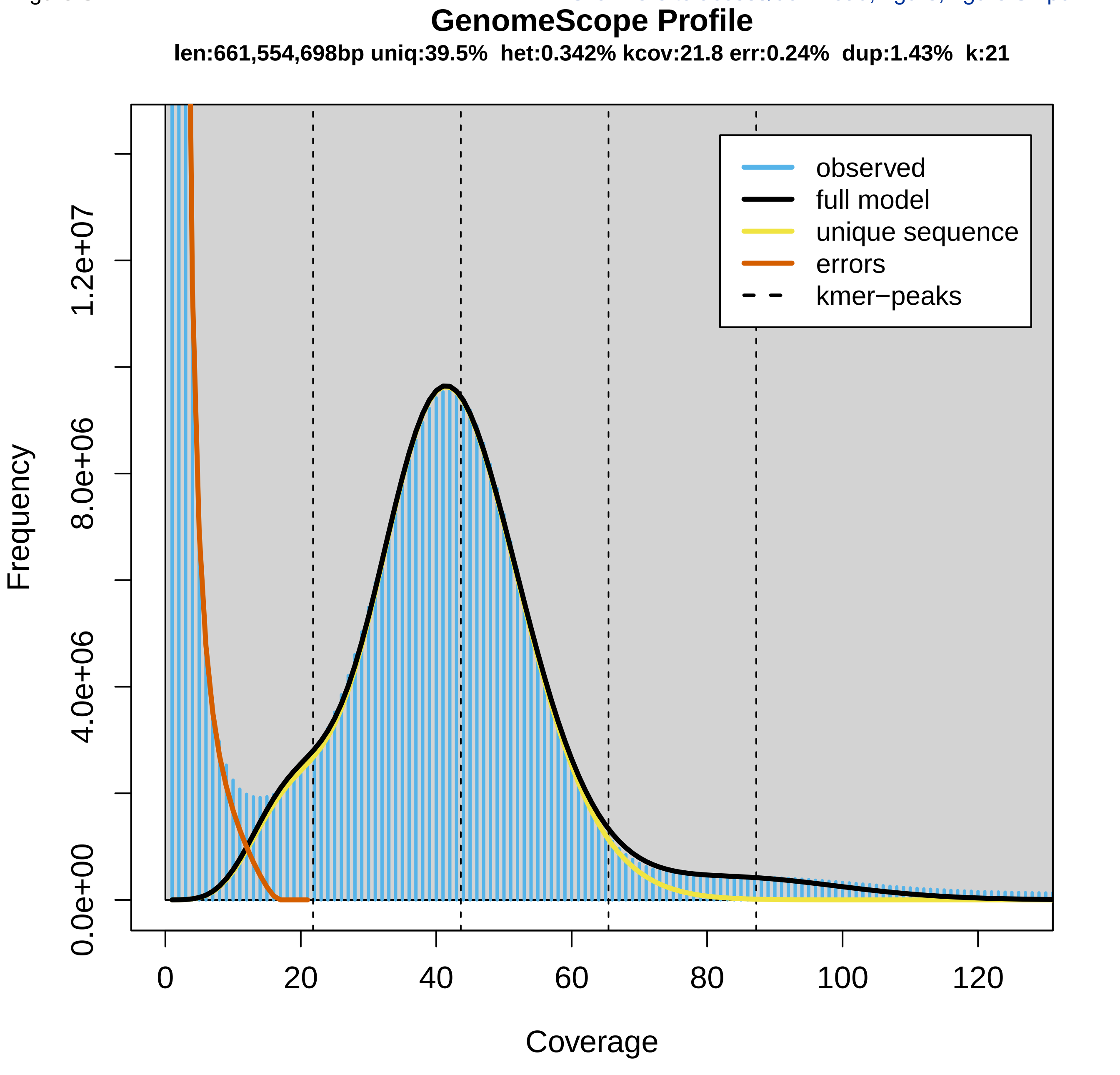

Figure S3

Click here to access/download;Figure;Figure S3.pdf

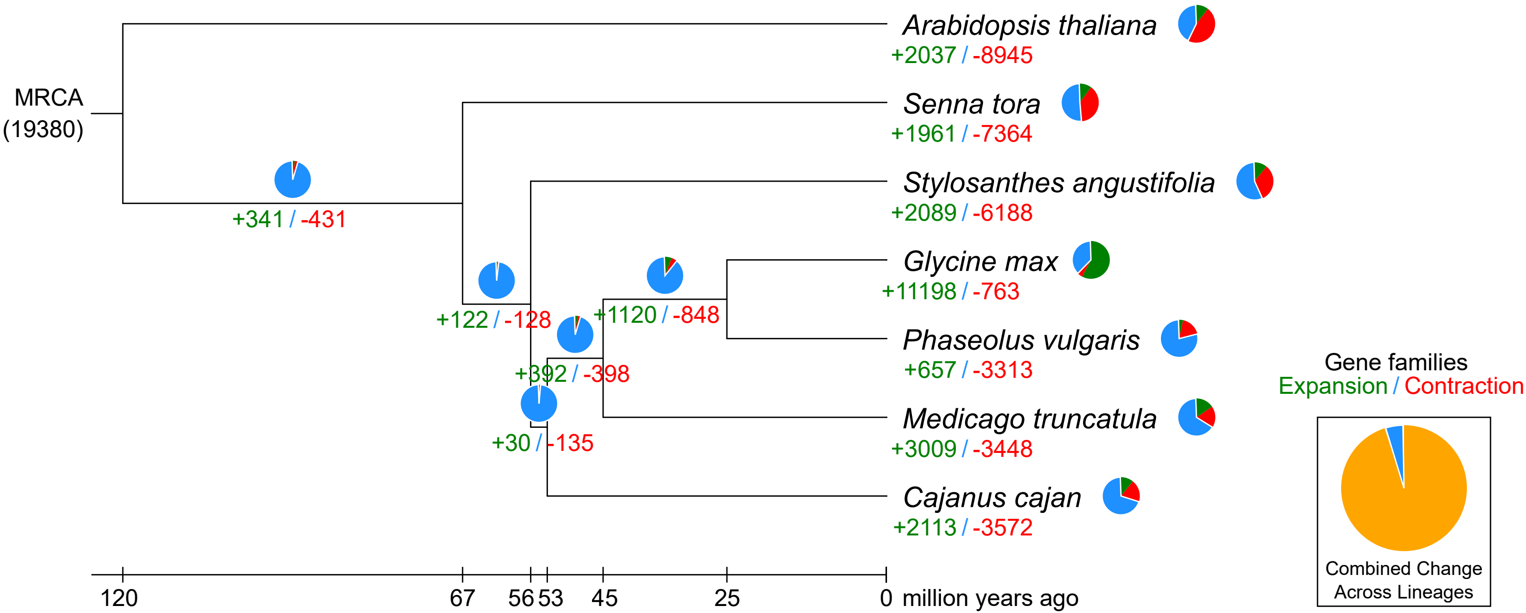

Figure S4

Click here to access/download;Figure;Figure S4.pdf

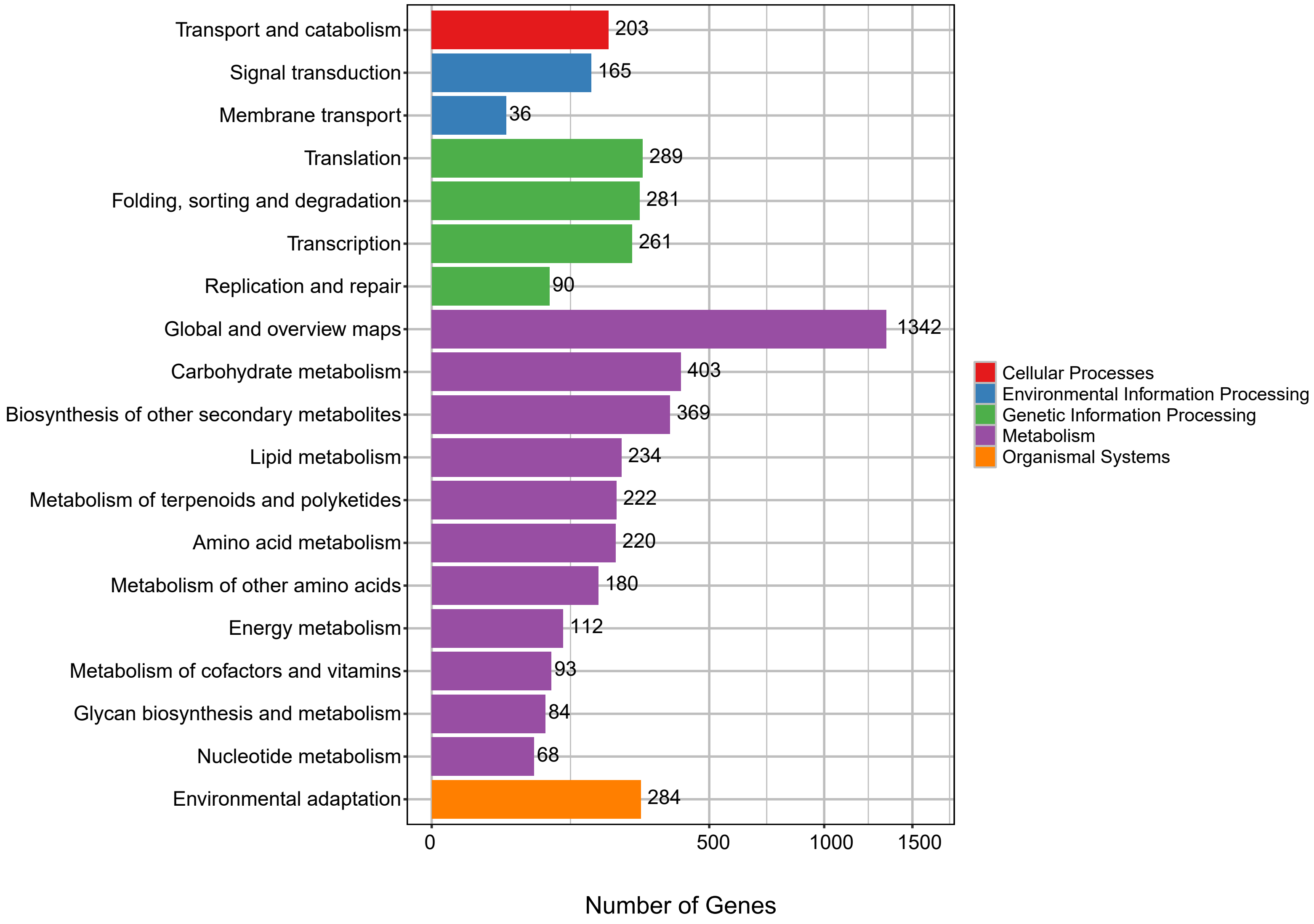

Figure S5

Click here to access/download;Figure;Figure S5.pdf

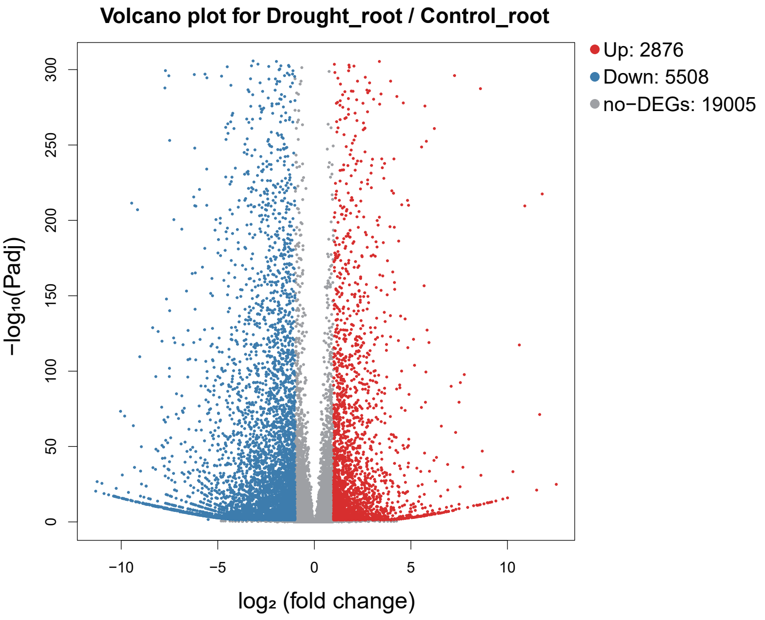

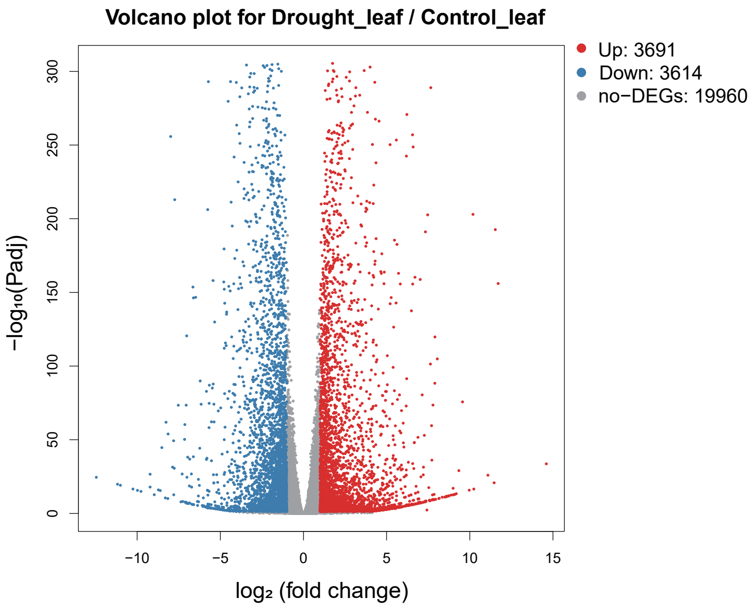

Figure S6

Click here to access/download;Figure;Figure S6.pdf

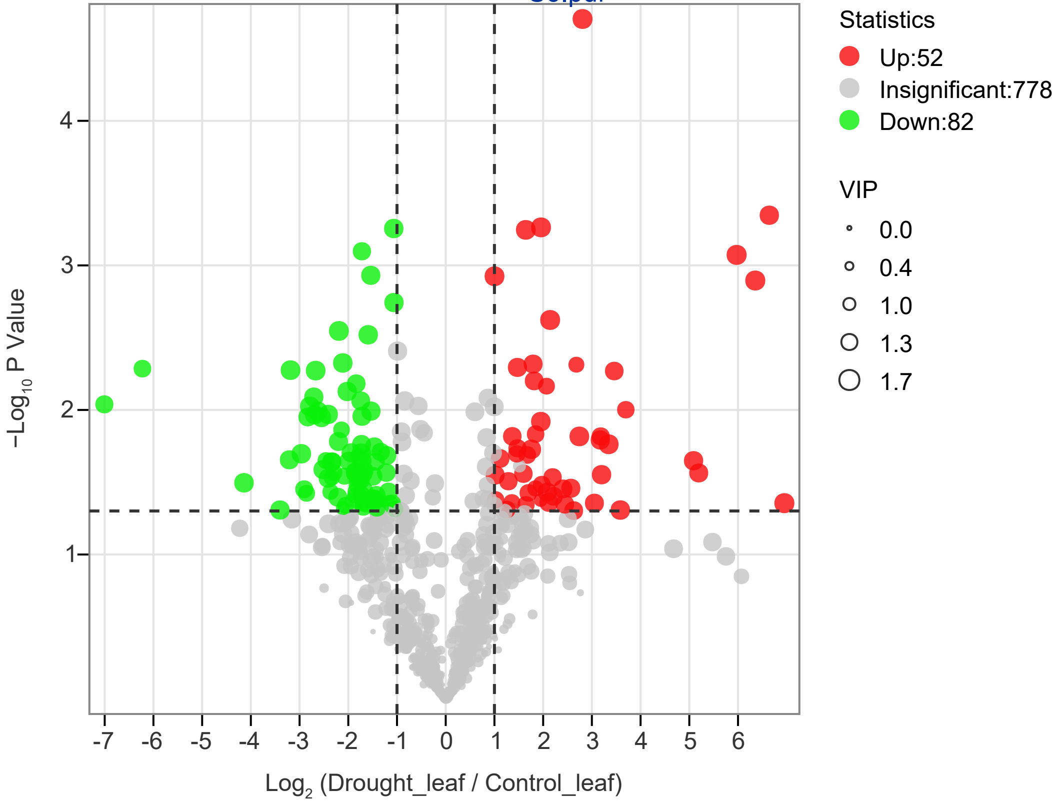

Figure S7

Click here to access/download;Figure;Figure S7.pdf

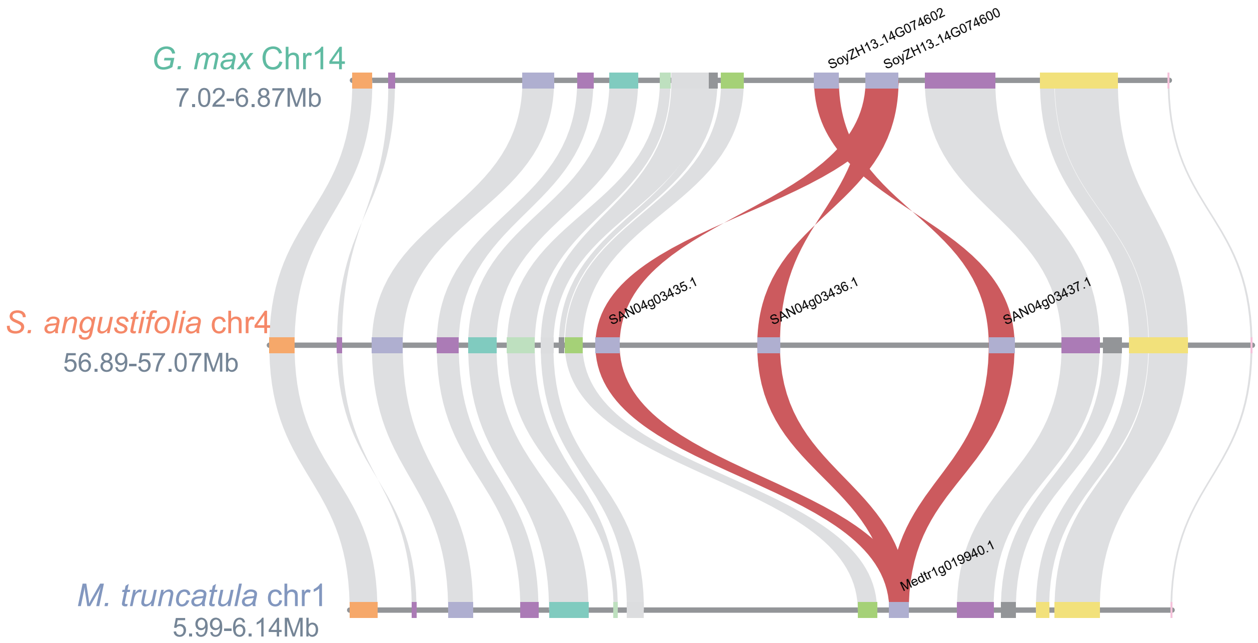

Click here to access/download
Supplementary Material
Supplementary Tables.xlsx
