# Supplementary material for: A chromosome-scale genome assembly of the pioneer plant Stylosanthes angustifolia: insights into genome evolution and drought adaptation
Source: Gigascience. 2025 Jan 24;14:giae118. doi: 10.1093/gigascience/giae118 (PMC11758145; doi:10.1093/gigascience/giae118)
Supplement: giae118_GIGA-D-24-00294_Revision_1 [file giae118_giga-d-24-00294_revision_1.pdf]

GigaScience

The telomere-to-telomere (T2T) genome of the pioneer plant Stylosanthes angustifolia: insights into genome evolution and drought adaptation

--Manuscript Draft--

**Manuscript Number:**	GIGA-D-24-00294R1
**Full Title:**	The telomere-to-telomere (T2T) genome of the pioneer plant Stylosanthes angustifolia: insights into genome evolution and drought adaptation
**Article Type:**	Research
**Funding Information:**	

Funding	Recipient
Earmarked fund for China Agriculture Research System—Forage and Grass (CARS-34)	prof. Guodao Liu
Central Public-interest Scientific Institution Basal Research Fund for CATAS (1630032022023 and 1630032024016)	Prof. Pandao Liu
Earmarked fund for China Agriculture Research System—Green Manure (CARS-22)	prof. Guodao Liu
Natural Science Foundation of Hainan Province (323CXTD387)	Prof. Pandao Liu
National Natural Science Foundation of China (32371769)	Prof. Pandao Liu
Guangxi Special Project for Innovation-driven Development (Guike AA18242040)	Dr. Zhu Qiao

Abstract:

Background: Drought is a major limiting factor for plant survival and crop productivity. Stylosanthes angustifolia, a pioneer plant, exhibits remarkable drought tolerance, yet the molecular mechanisms driving its drought resistance remain largely unexplored.
Results: We present the telomere-to-telomere (T2T) genome assembly of S. angustifolia, providing insights into its genome evolution and drought tolerance mechanisms. The assembled genome is 631.17 Mb in size, containing 319.98 Mb of repetitive sequences and 36,857 protein-coding genes. Notably, 17 telomeric sequences and 10 centromeric sequences were identified across 10 chromosomes. The high quality of this genome assembly is demonstrated by the presence of 99.26% Benchmarking Universal Single-Copy Orthologs and a 19.49 LTR assembly index. Evolutionary analyses revealed that S. angustifolia shares a whole-genome duplication (WGD) event with other legumes but lacks recent WGD. Additionally, S. angustifolia has undergone gene expansion through tandem duplication approximately 12.31 million years ago. Through integrative multi-omics analyses, we identified four gene families, namely xanthoxin dehydrogenase, 2-hydroxyisoflavanone dehydratase, patatin-related phospholipase A, and stachyose synthetase, that underwent tandem duplication and were significantly up-regulated under drought stress. These gene families contribute to the biosynthesis of abscisic acid, genistein, daidzein, jasmonic acid, and stachyose, thereby enhancing drought tolerance.
Conclusions: Our T2T genome assembly of S. angustifolia represents a significant advancement in understanding the genetic mechanisms underlying drought tolerance in this pioneer plant species. This genomic resource provides critical insights into the evolution of drought resistance and offers valuable genetic information for breeding programs aimed at improving drought resistance in crops.

**Corresponding Author:**	Pandao Liu<br>Chinese Academy of Tropical Agricultural Sciences Tropical Crops Genetic Resources Institute<br>Haikou, CHINA
**Corresponding Author Secondary Information:**	
**Corresponding Author's Institution:**	Chinese Academy of Tropical Agricultural Sciences Tropical Crops Genetic Resources Institute

Powered by Editorial Manager® and ProduXion Manager® from Aries Systems Corporation

**Corresponding Author's Secondary Institution:**	
**First Author:**	Chun Liu
**First Author Secondary Information:**	
**Order of Authors:**	Chun Liu
	Jianyu Zhang
	Ranran Xu
	Jinhui Lv
	Zhu Qiao
	Mingzhou Bai
	Shancen Zhao
	Lijuan Luo
	Guodao Liu
	Pandao Liu
**Order of Authors Secondary Information:**	
**Response to Reviewers:**	GIGA-D-24-00294<br><br>Dear Reviewers and Editors:<br><br>Thank you very much for your valuable feedback and comments. Your suggestions have been instrumental in revising and improving our manuscript. As recommended, we have carefully revised the manuscript and made corrections that we hope will meet your approval. The modified sections are highlighted in red within the manuscript. The main corrections in the paper and the responses to the reviewer's comments are as follows:<br><br>Reviewer #1: This manuscript by Liu et al reports the first telomere-to-telomere (T2T) genome assembly of Stylosanthes angustifolia. Together with previously published Legume and Arabidopsis genomes, they conducted evolutionary analyses. They also conducted transcriptome sequencing and metabolome profiling in S. angustifolia under both drought and control treatments to explore the mechanisms underlying the drought tolerance. Please see below all my comments.<br>Major concerns:<br>1.I question whether this genome is truly near-complete with only one gap, as claimed. The authors did not provide detailed information regarding how the gaps were closed. Although the 3rd generation sequencing technologies have advanced substantially, creating a gapless genome remains challenging. Typically, this process requires combining various state-of-the-art sequencing technologies (HiFi, ONT ultra-long reads and Hi-C) and/or different gap-closing strategies. The authors combined ONT (N50: 27 kb, which is not ultra-long), Hi-C and Illumina reads to assemble what they describe as a T2T genome containing only one gap. However, several questions remain: How many contigs were generated by NextDenovo? How many of these represent complete chromosomes? How many were anchored to chromosomes using HI-C data? How many gaps were there before the gap-closing step? Also, the authors need to carefully check the gaps that were closed by LR_Gapcloser, since they did not generate new data but relied solely on existing ONT reads.<br>REPLY: Thank you for the valuable comments regarding the completeness of our genome assembly and the gap-closing process. We utilized NextDenovo to correct and assemble the 158x ONT sequencing data, resulting in 167 initial contigs (Contig N50: 14.99 Mb and 13 contigs). These initial contigs were further polished using both ONT and NGS reads to generate high-quality contigs. Subsequently, Hi-C reads were used to successfully anchor 106 contigs (totaling 631.54 Mb in length) to 10 chromosomes, which initially contained 96 gaps. To further refine the assembly, we performed two rounds of gap filling using LR_Gapcloser with raw ONT reads, successfully closing 95 of these gaps.

Powered by Editorial Manager® and ProduXion Manager® from Aries Systems Corporation

The decision to use raw ONT reads for gap filling was made to avoid the potential discontinuities caused by misprocessing of complex regions during the ONT read correction step in NextDenovo. In addition, we used NGS data to detect SNPs, calculating a heterozygosity rate of 0.06% for this genome based on heterozygous SNPs. We believe that the low heterozygosity of this genome, coupled with the high ONT sequencing depth, enabled us to achieve a near-complete assembly of this genome using only ONT, NGS, and Hi-C data. Moreover, the quality assessment of the genome assembly showed a BUSCO completeness score of 99.26%, LTR assembly index of 19.49, and k-mer completeness of 96.37%, further supporting the high quality and integrity of the assembly.
Additionally, the use of the same sequencing technology for both genome assembly and gap-filling is also adopted by researchers (Nature Communications, 2024, DOI: 10.1038/s41467-024-51158-3; BMC Genomics, 2024, DOI: 10.1186/s12864-024-10711-6). (see lines 130 to 138, lines 400to 423 for details)

2.For the drought experimental design, the authors wrote: the drought treatment group consisted of 60-day-old seedlings that were not watered for 5 days, while the control group consisted of 60-day-old seedlings that were not subjected to the drought treatment. I was wondering whether this is a widely accepted method for drought experiment? The authors did not measure the soil moisture. How can the authors ensure that the plants are truly water-deficit? Also, how often did the authors water the plants for the control group?
REPLY: Thank you for the valuable comments regarding our drought experiment.
Drought Experiment Methodology: There may have been an unclear description in our original manuscript. In fact, we conducted a dynamic drought treatment by stopping watering for the 60-day-old seedlings. Seedlings were sampled by collecting leaves and roots at day 0 (D0) for the control group, and at days 3 (D3) and 5 (D5) after drought for the treatment groups. Due to the most visible phenotypic changes appearing after 5 days of drought treatment, we presented data only at D0 and D5 in the original manuscript. In the revised manuscript, we present data on physiological phenotypes and transcriptome analyses after 3 days of drought treatment (D3). Additionally, we have provided a clearer description of the drought treatment process in the methods section. (see lines 498 to 515). In pot experiments, drought treatment was initiated by ceasing watering, with samples collected on various days of water cessation. This method is commonly used to impose drought stress on different plant species. (Nature Genetics, 2016, DOI:10.1038/ng.3636; Plant and Soil, 2019, DOI: 10.1007/s11104-018-3893-1; Plant Physiology, 2022, DOI: 10.1093/plphys/kiac272; International Journal of Biological Macromolecules, 2023, DOI: 10.1016/j.ijbiomac.2023.126582).
Assessment of Drought Stress. Since the experiment was conducted using potted plants, the soil water content is a more appropriate description of the drought conditions rather than soil moisture (Plant and Soil, 2019, DOI: 10.1007/s11104-018-3893-1). In the revised manuscript, we have included measurements for soil water content, shoot water content, and chlorophyll levels (chlorophyll a and b) on D0, D3, and D5. This ensures a robust assessment of the drought conditions. Soil water content was quantified using a previously reported method (Plant and Soil, 2019, DOI: 10.1007/s11104-018-3893-1; Physiol Plantarum, 2016, DOI:10.1111/ppl.12433; Physiol Plantarum, 2016, DOI: 10.1111/ppl.12346), with soil water content significantly decreasing from 100% pot capacity on D0 to 37.84% by D3, and 18.09% by D5 (P-value < 0.05). Additionally, there was a significant reduction in both shoot water content and chlorophyll levels at D3 and D5 compared to D0 (P-value < 0.05). These measurements confirm that the plants were subjected to drought stress.
Transcriptome Sequencing and Metabolite Analysis: In the drought experiment, we collected root and leaf samples on D0, D3, and D5, and performed transcriptome sequencing on all these samples. In a previous version of the manuscript, we included only transcriptome data from D0 and D5 because the phenotypic differences were most pronounced on D5. However, in this revised version, we have analyzed and compared the transcriptome data for D0, D3, and D5 to provide a more comprehensive understanding of S. angustifolia response to drought stress. Since the phenotypic differences were most evident on D5, metabolite analysis was conducted solely on D0 and D5 to investigate changes in metabolite composition. Notably, the same samples were used for both transcriptome sequencing and metabolite analysis.
(see lines 213 to 227 and lines 498 to 530 for details)

Powered by Editorial Manager® and ProduXion Manager® from Aries Systems Corporation

	3.Abstract: too much genome assembly statistics were written in abstract.<br>REPLY: Thank you for your valuable comments. We have revised the abstract to include only the genomic assessment results from BUSCO and LAI. (see Abstract for details)<br><br>4.The authors may consider extensive revision for the introduction. They used lengthy text to describe the sequencing technologies that can generate T2T genomes. However, only little was introduced for the species S. angusifolia. My reading-through the introduction did not find whether this is a diploid or polyploid. Are there any genomic studies have been published on it before? Why S. angusifolia is important in the genus Stylosanthes and why its genome needs to be sequenced?<br>REPLY: Thank you for your valuable comments. We have substantially revised the introduction section to better emphasize the significance of Stylosanthes angustifolia and the genus Stylosanthes. We clarified that S. angustifolia is a diploid species (2n = 2x = 20). Furthermore, we have reduced the content concerning telomere-to-telomere (T2T) sequencing to maintain focus on the biological aspects of the species.<br>Stylosanthes guianensis (2n = 2x = 20), the most widely domesticated and utilized species in the genus Stylosanthes, serves as a significant forage and green manure crop. Numerous cultivars have been developed for agricultural use in different countries. The utilization of wild relatives in genomic and genetic research is particularly valuable, as these species provide unique genetic diversity and traits that are essential for crop improvement. Our research targets S. angustifolia, a wild relative of S. guianensis, which exhibits remarkable adaptability in the dry-hot valley regions of southwestern China, where seasonal drought is common.<br>Moreover, we are collaborating with international partners to investigate the genome of S. guianensis (unpublished), which makes sequencing S. angustifolia and elucidating its drought resistance mechanisms even more crucial. This study lays a foundation for the improvement of S. guianensis and offers valuable insights for breeding drought-tolerant cultivars within the genus. (see lines 79 to 105 for details)<br><br>5.Did the authors use the same samples (tissues) for both mRNA-Seq sequencing and the lipidomics, ABA, JA and JA-Ile, genistein, daidzein, and raffinose determination?<br>REPLY: Thank you for your valuable comments. Yes, in our study, the same samples were utilized for both transcriptome sequencing and metabolite analysis. (see line 513 and 515 for details)<br><br>6.Table S4: The meaning of "Chr window" is unclear? In chr4, the "TTTAGGG" was located ~8Mb. Is this sequence truly a telomere, which typically occurs at chromosome ends?<br>REPLY: Thank you for raising this important question regarding the "TTTAGGG" sequence located at approximately 8 Mb on chromosome 4. While this sequence consists of 43 repeats, it is insufficient to classify this region as a functional telomere, which is defined by its specific location at chromosome ends and its association with telomere-binding proteins. The observed sequence is more likely an interstitial telomeric sequence (ITS), a common feature in plant genomes such as Arabidopsis thaliana, often arising from historical chromosomal rearrangements or duplications. These internal repeats do not serve the protective roles of functional telomeres but are remnants of past genomic events (Pl Syst Evol, 1995, doi.org/10.1007/BF00982962; Plants, 2021, doi.org/10.3390/plants10112541). Additionally, as noted in the discussion, further validation is necessary to definitively determine whether this is a true telomere or an ITS, and comparative genomics of related species could offer additional insights. (see lines 143 to 146, lines 328 to 333 for details)<br>We changed "Chr window" to the chromosome start and end positions in this revision. (see Table S4 for details)<br><br>7.Lines 159-163: The authors conclude that the length of the centromeres differ significantly, ranging from 141 kb to 25.6 Mb. This is a main result shown in Table 2. More evidences are needed to support this conclusion, not solely based on the distribution of tandem repeats and LTRs.<br>REPLY: Thank you for your valuable comments. In the revised version, we used CentIER (Plant Communications, 2024, DOI: 10.1016/j.xplc.2024.101046), a recently published tool, for centromere identification. Unlike traditional methods that rely solely on detecting tandem repeat sequences, CentIER was designed to account for various sequence features, including tandem repeats, retrotransposons, and k-mer frequency

	distribution. We combined the software's results with the k-mer distribution plots of the chromosomes to accurately define the centromeric regions. (see lines 156 to 161 and lines 458 to 463 for details)<br><br>Minor concerns:<br>1.Please ensure all mentions of significant, significantly, and significance refer to statistically tested significance. The level of significance (p-value) and name of the statistical test should be provided, either in the text or in the appropriate table/figure/dataset. For mentions not supported by statistical analyses, please choose another word, such as substantial, substantially, importance, etc.<br>REPLY: Thank you for regarding the terminology related to significance. We have carefully revised the manuscript to ensure that all mentions of "significant," "significantly," and "significance" explicitly refer to statistically tested significance, including the relevant p-values and names of statistical tests. For instances not supported by statistical analyses, we have replaced these terms with alternatives.<br><br>2.It is not clear whether the genome of S. angusifolia is highly heterozygous or not. It seems that the authors performed genome survey analysis (shown in Figure S2). It is never mentioned in the main text.<br>REPLY: Thank you for your valuable comments. We performed a genome survey analysis using k-mer analysis based on second-generation sequencing (NGS) data, which estimated the genome size to be 661.55 Mb. To assess heterozygosity, we aligned the NGS data to the assembled genome using BWA and identified variants with GATK. Our analysis detected 379,931 heterozygous SNPs, corresponding to a heterozygosity rate of 0.06% for S. angustifolia, indicating that the genome is not highly heterozygous. (see lines 140 to 142 and Figure S2 for details)<br><br>3.Line 412: LTR_Retriever is actually implemented in the EDTA tool.<br>REPLY: Thank you for your notification. We have addressed this issue in the revised version.<br><br>4.Line 444: LTR_Retriever is the tool for LTR retrotransposons identification. TEs generally refer to all types of repeats. Please make it clear.<br>REPLY: Thank you for your valuable comments. We have addressed this issue in the revised version. (see 427 to 428 for details)<br><br>5.Line 201: Is there a particular reason why the other species were excluded for the expansion/contraction analysis?<br>REPLY: Thank you for your valuable comments. The exclusion of certain species (e.g., Aeschynomene evenia, Arachis ipaensis, and Arachis duranensis) from the expansion/contraction analysis was based on several factors. First, we aimed to focus on species that are more phylogenetically relevant to S. angustifolia, along with well-annotated and widely studied legumes (e.g., Glycine max, Phaseolus vulgaris, and Cajanus cajan) to obtain robust results. These species better represent evolutionary trends within the legume family, which is our primary focus. Additionally, Arabidopsis thaliana was included as an outgroup. We excluded other species due to considerations related to the completeness of their genome annotations and the potential complexity they could introduce into the analysis. By concentrating on a subset of legumes with higher-quality data, we aimed to ensure that the results remain interpretable and computationally feasible. We believe this focused selection allows us to more accurately reflect gene family dynamics relevant to our study objectives. (see lines 195 to 198 for details)<br><br>6.Lines 206-208: Not "some". I see extensive genomic rearrangements among these species.<br>REPLY: We have revised the description as follows: The genomes of S. angustifolia, A. duranensis, and A. ipaensis show extensive genomic rearrangements, particularly on chromosomes chr3, chr9, and chr10. However, certain regions of the genomes still maintain strong synteny, such as chromosomes chr1, chr6, and chr8. (see lines 202 to 204 for details)<br><br>7.Line 226: how many up-regulated genes overlap between roots and leaves?<br>REPLY: Thank you for your valuable comments. After 3 days of drought treatment, a total of 384 genes were upregulated in both roots and leaves. After 5 days of drought

treatment, this number increased to 1,246 genes. (see Figure S8 and lines 225 to 227 for details)

8.Lines 234-235: Not clear. Do you mean that there are seven ABA2 genes that independently
underwent tandem duplication, resulting in many tandem duplicated genes? Or are there a total of seven tandem duplicated ABA2 genes?
REPLY: Thank you for your valuable comments. There was a total of seven tandem duplicated ABA2 genes on chromosome 4 of S. angustifolia. (see Figure 4b and line 238 to 239 for details)

9.Why were lipidomics analysis, raffinose, genistein and daidzein determination performed only in leaves but not in roots?
REPLY: Thank you for your valuable comments. In the previous version of the manuscript, we presented data on these metabolites exclusively for leaf samples on D0 and D5, as we observed that the genes responsible for synthesizing these metabolites were more prominently upregulated in the leaves. In this revised version, metabolite analysis, including lipidomics analysis, as well as the quantification of raffinose, stachyose, genistein, and daidzein, was conducted on root and leaf samples collected on D0 and D5 following drought treatment. We present the accumulation of these metabolites in both root and leaf tissues to facilitate a more comprehensive integrated analysis with the transcriptome data. (see lines 258 to 314 and lines 532 to 602 for details)

Reviewer #2: In the manuscript entitled "The telomere-to-telomere (T2T) genome of the pioneer plant Stylosanthes angustifolia provides insight into its genome evolution and drought adaptation mechanisms" , the authors present a T2T genome sequence of Stylosanthes angustifolia, a significant member of the legume family. They offer detailed analyses of the genome's features, including whole genome duplication (WGD), synteny, and gene expansions resulting from duplications. Given that the sequenced species exhibits strong drought resistance, the authors explore the underlying mechanisms using transcriptomic analysis. Interestingly, they attempt to establish a causal relationship between elevated gene expressions and gene duplication. In my opinion, the reported genome and the accompanying analyses provide valuable and intriguing data resources for researchers focusing on legumes. While the manuscript has its merits, several issues need to be addressed before publication.
1. Please carefully make the writings more readable. For example, the authors used several "first" to emphasis the novelty of the genome, which are unusual expressions.
REPLY: Thank you for your valuable feedback. Upon reviewing the manuscript, we agree that the repeated use of "first" to highlight the novelty of the genome may detract from the clarity of the text. To address this, we have thoroughly revised the entire manuscript to improve readability and avoid redundancy.
2. It had better remove "mechanisms" from the title.
REPLY: Thank you for your valuable comments. In the revised manuscript, we changed the title to "The telomere-to-telomere (T2T) genome of the pioneer plant Stylosanthes angustifolia: insights into genome evolution and drought adaptation".

3. As the authors reported the estimated genome size and the assembled genome size, we see some discordance between the two, could the authors give some explanations?
REPLY: Thank you for pointing out the discrepancy between the estimated and assembled genome sizes. In the manuscript, we initially estimated the genome size of S. angustifolia to be 661.55 Mb using k-mer analysis. However, the final assembled genome size was 631.17 Mb, resulting in a difference of approximately 4.6%. This discordance can be attributed to several factors:
Repetitive Regions and Heterozygosity: The k-mer analysis used for the initial genome size estimation may overestimate genome size due to the inclusion of repetitive sequences and heterozygous regions, which can lead to inflation in the estimated size. During the assembly process, some of these repetitive regions may collapse or be filtered out, leading to a smaller assembled genome size. Residual Gaps and Unresolved Regions: Despite achieving a near-complete telomere-to-telomere (T2T) assembly, a gaps and unresolved regions remain, which could also contribute to the slight reduction in genome size compared to the estimated value. This slight

	discordance between the estimated and assembled genome sizes is a common observation in T2T plant genomes. For instance, similar discrepancies were reported in the assembly of the soybean (Glycine max) and maize (Zea mays) genomes. In the soybean genome assembly, the estimated genome size was 1.115 Gb (Williams 82) (Nature, 2010, DOI: 10.1038/nature08670), but the final T2T genome size was 1.011 Gb (Williams 82) (Molecular Plant, 2023, DOI: 10.1016/j.molp.2023.08.012), with a difference of 9.33%. Likewise, in the maize T2T assembly, the estimated genome size was 2.3 Gb, while the final assembled size was 2.178 Gb (nature genetics, 2023, DOI: 10.1038/s41588-023-01419-6), with a 5.2% difference. These examples illustrate that minor reductions in size from the estimated genome are a common occurrence in high-quality genome assemblies.<br><br>4. In the evolutionary tree, the authors used Arabidopsis as root, why not consider grape?<br>REPLY: We chose Arabidopsis thaliana as the outgroup for the phylogenetic tree based on its well-established role as a model organism, with a fully sequenced and highly annotated genome. Arabidopsis has been widely used in phylogenetic analyses across various plant families, including legumes, due to its comprehensive genomic resources and its position as a representative of the Brassicales, a well-known eudicot clade. While Vitis vinifera is also a eudicot, Arabidopsis is a more commonly accepted outgroup in legume-related studies. Its inclusion allows us to maintain consistency with previous gene family and divergence analyses, ensuring reliable comparative results. Additionally, the high level of functional annotation available for Arabidopsis provides a strong reference for identifying gene family expansions and contractions in legumes. We believe that using Arabidopsis thaliana as the outgroup provides the most appropriate framework for our study and helps ensure the accuracy and interpretability of our evolutionary analyses. (see 187 to 188 for details)
**Additional Information:**	
**Question**	**Response**
Are you submitting this manuscript to a special series or article collection?	No
**Experimental design and statistics**<br><br>Full details of the experimental design and statistical methods used should be given in the Methods section, as detailed in our Minimum Standards Reporting Checklist. Information essential to interpreting the data presented should be made available in the figure legends.<br><br>Have you included all the information requested in your manuscript?	Yes
**Resources**<br><br>A description of all resources used, including antibodies, cell lines, animals and software tools, with enough information to allow them to be uniquely identified, should be included in the Methods section. Authors are strongly	Yes

Powered by Editorial Manager® and ProduXion Manager® from Aries Systems Corporation

encouraged to cite Research Resource Identifiers (RRIDs) for antibodies, model organisms and tools, where possible.<br><br>Have you included the information requested as detailed in our Minimum Standards Reporting Checklist?	
**Availability of data and materials**<br><br>All datasets and code on which the conclusions of the paper rely must be either included in your submission or deposited in publicly available repositories (where available and ethically appropriate), referencing such data using a unique identifier in the references and in the “Availability of Data and Materials” section of your manuscript.<br><br>Have you have met the above requirement as detailed in our Minimum Standards Reporting Checklist?	Yes

Manuscript
Click here to access/download;Manuscript;manuscript-SAN-revision_R1.docx

The telomere-to-telomere (T2T) genome of the pioneer plant *Stylosanthes angustifolia*: insights into genome evolution and drought adaptation

Chun Liu[1,2,3,4], Jianyu Zhang[1,2,3,4], Ranran Xu[1,2,3,4], Jinhui Lv[1,2,3,4], Zhu Qiao[5], Mingzhou Bai[6], Shancen Zhao[7], Lijuan Luo[1], Guodao Liu[2,*], and Pandao Liu[2,3,4,*]

[1] School of Tropical Agriculture and Forestry & Sanya Institute Breeding and Multiplication, Hainan University, Haikou/Sanya 570228/572025, China.
[2] Tropical Crops Genetic Resources Institute, Chinese Academy of Tropical Agricultural Sciences (CATAS), Haikou 571101, China
[3] Key Laboratory of Crop Gene Resources and Germplasm Enhancement in Southern China, Ministry of Agriculture and Rual Affairs, Haikou 571101, China.
[4] Key Laboratory of Tropical Crops Germplasm Resources Genetic Improvement and Innovation of Hainan Province, Haikou 571101, China.
[5] Guangxi Key Laboratory of Medicinal Resources Protection and Genetic Improvement/ Guangxi Engineering Research Center of TCM Resource Intelligent Creation, Guangxi Botanical Garden of Medicinal Plants, Nanning 530023, China.
[6] Department of Biotechnology and Biomedicine, Technical University of Denmark, Kongens Lyngby 2800, Denmark.
[7] Beijing Life Science Academy, Beijing 102200, China.

E-mail addresses of all authors
Chun Liu: xiaoyaoma@live.cn
Jianyu Zhang: jianyuzhang@hainanu.edu.cn
Ranran Xu: 2512891913@qq.com
Jinhui Lv: lvjinhuixue@163.com
Zhu Qiao: qiaozhu@gxyyzwy.com
Mingzhou Bai: mingbai@dtu.dk
Shancen Zhao: zhaosc@blsa.com.cn
Lijuan Luo: luoljd@126.com
Guodao Liu: Guodao_Liu@163.com
Pandao Liu: liupandao2019@163.com

*Correspondence addresses.
Pandao Liu, CATAS, West Xueyuan Road, Haikou 571101, China. E-mail: liupandao2019@163.com, ORCID: 0000-0003-2296-9130;
Guodao Liu, CATAS, West Xueyuan Road, Haikou 571101, China. E-mail: Guodao_Liu@163.com, ORCID: 0000-0003-4189-9959.

Abstract

Background: Drought is a major limiting factor for plant survival and crop productivity. *Stylosanthes angustifolia*, a pioneer plant, exhibits remarkable drought tolerance, yet the molecular mechanisms driving its drought resistance remain largely unexplored.

Results: We present the telomere-to-telomere (T2T) genome assembly of *S. angustifolia*, providing insights into its genome evolution and drought tolerance mechanisms. The assembled genome is 631.17 Mb in size, containing 319.98 Mb of repetitive sequences and 36,857 protein-coding genes. Notably, 17 telomeric sequences and 10 centromeric sequences were identified across 10 chromosomes. The high quality of this genome assembly is demonstrated by the presence of 99.26% Benchmarking Universal Single-Copy Orthologs and a 19.49 LTR assembly index. Evolutionary analyses revealed that *S. angustifolia* shares a whole-genome duplication (WGD) event with other legumes but lacks recent WGD. Additionally, *S. angustifolia* has undergone gene expansion through tandem duplication approximately 12.31 million years ago. Through integrative multi-omics analyses, we identified four gene families, namely *xanthoxin dehydrogenase*, *2-hydroxyisoflavanone dehydratase*, *patatin-related phospholipase A*, and *stachyose synthetase,* that underwent tandem duplication and were significantly up-regulated under drought stress. These gene families contribute to the biosynthesis of abscisic acid, genistein, daidzein, jasmonic acid, and stachyose, thereby enhancing drought tolerance.

Conclusions: Our T2T genome assembly of *S. angustifolia* represents a significant advancement in understanding the genetic mechanisms underlying drought tolerance in this pioneer plant species. This genomic resource provides critical insights into the evolution of drought resistance and offers valuable genetic information for breeding programs aimed at improving drought resistance in crops.

Keywords: *Stylosanthes angustifolia*, Pioneer plant, T2T genome, Multi-omics, Drought tolerance

Introduction

Drought represents one of the most significant environmental challenges, drastically impairing plant survival and considerably reducing annual crop yields [1,2]. Plants have developed a range of physiological, biochemical, and morphological mechanisms to respond to drought stress. These

include stomatal closure to minimize transpiration, alterations in root architecture to optimize water uptake, and increased biosynthesis of compounds such as abscisic acid (ABA), osmoprotectants, flavonoids, and isoflavonoids [1,3–5]. Additionally, the accumulation of non-reducing sugars, such as raffinose, plays a crucial role in drought adaptation [6,7]. Understanding the genes and molecular pathways that underpin these adaptive responses is essential for advancing the development of drought-tolerant crop cultivars [8–10]. Therefore, comprehensive research on the genetic mechanisms that enable plants to withstand drought stress is urgently needed.

The genus *Stylosanthes* (family Leguminosae) comprises approximately 50 species, including diploids (2n = 2x = 20), tetraploids (2n = 4x = 40), and hexaploids (2n = 6x = 60) species, distributed across tropical and subtropical regions [11,12]. This genus is a pioneer plant in acid soils[13], exhibiting superior adaptability to frequent abiotic stresses such as low phosphorus availability [14,15], aluminum toxicity [16,17], manganese toxicity [18,19], and low pH [20]. *Stylosanthes guianensis* (2n = 2x = 20) is the most widely domesticated and utilized species in the genus *Stylosanthes*, serving as both forage and green manure [21]. Numerous cultivars of *S. guianensis* have been developed across different countries: the cultivars “Bandeirante”, “Mineirão”, and “IRI 1022” in Brazil; the cultivars “Schofield”, “Endeavour”, and “Cook” in Australia; and the cultivars “Reyan No. 2”, “Reyan No. 5”, and “Stylo 907” in China [13,22,23]. The significance of utilizing wild relatives in genomic and genetic research is immense, as they offer invaluable genetic diversity and traits that are crucial for enhancing cultivars [24–26]. *S. angustifolia*, a diploid species (2n = 2x = 20), is a wild relative of *S. guianensis* [27]. We observed that *S. angustifolia* exhibits good adaptability in the dry-hot valley regions of southwestern China, which frequently experience seasonal drought. However, the potential mechanisms underlying its drought tolerance remain unclear. This study aims to elucidate the molecular basis of drought-tolerant traits in *S. angustifolia* through genomic sequencing, thereby providing genetic resources for breeding drought-adapted *Stylosanthes* cultivars.

Advances in third-generation sequencing (TGS) technologies, such as Pacific Biosciences (PacBio) and Oxford Nanopore Technologies (ONT), have enabled the assembly of genomes at the telomere-to-telomere (T2T) level, even in plant species characterized by high heterozygosity and complex repetitive sequences [28,29]. To date, only the tetraploid *S. scabra* genome has been

sequenced within the *Stylosanthes* genus, employing next-generation sequencing (NGS) [30]. In leguminous species, T2T genome assemblies have been completed for only a limited number of species, including soybean (*Glycine max*) [31,32] and *Sesbania cannabina* [33]. Given the importance of legumes for agriculture and the environment, additional T2T legume genomes are necessary to deepen our understanding of these vital species.

Gene duplication plays a pivotal role in generating genetic diversity and driving plant evolution and adaptation [34–36]. Tandem duplication is a significant driver of gene family expansion, often leading to gene members with nearly identical sequences and potentially redundant functions. Recent researches have highlighted the role of tandem duplicated genes (TDGs) in environmental adaptation, as demonstrated in species like pigeonpea (*Cajanus cajan*) [37,38]. grapevine (*Vitis vinifera*) [39], and silver birch (*Betula pendula*) [40]. Lineage-specific TDGs are particularly important for the adaptive evolution of plants in rapidly changing environments [41]. Whole-genome duplication (WGD) and tandem duplication have also been linked to the expansion of salinity adaptation genes in the halophyte *Tamarix chinensis* [42]. Genome-wide identification of TDGs, combined with multi-omics analyses, is essential for understanding how these genes contribute to plant evolution and environmental adaptation [38,42–44].

In this study, we present the T2T genome assembly of the leguminous pioneer plant *S. angustifolia*, utilizing ONT, NGS, and high-through chromosome conformation capture (Hi-C) technologies. Comparative genomics reveals the evolutionary position and divergence of *S. angustifolia*. Integrating comparative genomics, transcriptomics, and metabolomics, we demonstrate the critical role of TDGs in *S. angustifolia* genome evolution and its adaptation to drought stress.

Results

Sequencing and Assembly of the *S. angustifolia* Genome

In this study, we employed NGS and ONT technologies for the whole-genome sequencing of *S. angustifolia* (Germplasm number: TF0003 and Figure S1). A total of 35.08 Gb (~ 53.02 × coverage) NGS data and 104.72 Gb (~ 158.29 × coverage) ONT data were generated (Table S1 and Table S2). The estimated genome size of *S. angustifolia* was calculated as 661.55 Mb using k-mer analysis of

the NGS data (Figure S2). We used NextDenovo to correct and assemble the raw ONT data into an initial 167 contigs, with a total length of 645.87 Mb and an N50 of 14.98 Mb. Subsequently, we employed NextPolish to polish the initial contigs using both ONT and NGS data to obtain high-quality contigs. Hi-C technology was further applied for chromosomal-level assembly, generating 53.89 Gb (~ 81.47 × coverage) of clean data (Table S3). By analyzing the Hi-C data with Juicer and 3dDNA software, we aligned and grouped the high-quality contigs into chromosomes, achieving a chromosomal-level assembly of the *S. angustifolia* genome. As a result, a total of 106 contigs, spanning 631.54 Mb, were anchored into 10 chromosomes, with 96 gaps. To address these gaps, we used LR_Gapcloser, which successfully filled 95 of them using the raw ONT reads. The final T2T assembly of the *S. angustifolia* genome was 631.17 Mb in size, with a GC content of 35.53% and a contig N50 of 64.91 Mb (Figure 1 and Table 1). Notably, only one gap remained on chromosome 7 (Figure 1a). SNP analysis using genome analysis toolkit (GATK) revealed that the genome had 379,931 heterozygous SNPs, corresponding to a heterozygosity rate of 0.06%. Moreover, telomeric sequences (“TTTAGGG”) were identified at the ends of all chromosomes except one terminus on chromosomes 4, 7, and 10 (Figure 1a and Table S4). It is worth mentioning that a telomeric sequence was observed around 8 Mb on chromosome 4, repeated 43 times. This is likely an interstitial telomeric sequence (ITS) that resulting of historical chromosomal structural variations. The completeness of the assembled genome was evaluated using Benchmarking Universal Single-Copy Orthologs (BUSCO) and LTR assembly index (LAI), resulting in scores of 99.26% and 19.49, respectively (Table 1 and Table S5). Additionally, k-mer completeness analysis yielded a score of 96.37% for the *S. angustifolia* genome (Table 1). The mapping rates for NGS and ONT reads were 99.15% and 99.69%, respectively, with average depths of 54.16× for NGS reads and 156.20× for ONT reads. In total, 95.16% and 99.96% of the genome was covered by NGS and ONT reads, respectively.

Table 1. Statistics of genomic features of *S. angustifolia*

**Terms**	***S. angustifolia***
Estimated genome size (Mb)	661.55
Assembled genome size (Mb)	631.17

Contig N50 (Mb)	64.91
GC content (%)	35.53
BUSCO (%)	99.26
LTR assembly index	19.49
K-mer completeness (%)	96.37
Number of chromosomes	10
Repeat content (Mb)	319.98
Repeat ratio (%)	50.70
Number of protein-coding genes	36,857
Mean exon length (bp)	242.95
Mean intron length (bp)	465.57

Identification of Candidate Centromeres on the *S. angustifolia* Genome

Centromeres, defined as regions with high densities of short tandem repeats and low gene density, were identified using CentIER software. Except for chromosome 10, the centromeric sequences were located approximately in the middle of each chromosome (Table 2 and Figure S3). The lengths of the centromeric regions ranged from 2.00 Mb to 5.17 Mb, with GC contents slightly higher than that of average GC content of the genome (35.53%) (Table 2).

Table 2. Identification of centromeres in the assembled *S. angustifolia* genome.

Chr	Start	End	Length (Mb)	GC (%)
chr1	35,100,001	40,100,000	5.00	37.84
chr2	34,700,001	38,700,000	4.00	37.31
chr3	33,113,333	39,446,666	6.33	38.21
chr4	33,920,000	37,919,999	4.00	38.02
chr5	38,933,334	42,766,667	3.83	37.93
chr6	37,350,000	42,349,999	5.00	38.24
chr7	36,400,001	38,400,000	2.00	36.45
chr8	31,816,666	36,983,333	5.17	38.65
chr9	24,030,000	29,029,999	5.00	38.46
chr10	4,433,334	8,266,667	3.83	37.88

Genome Annotation of the *S. angustifolia* Genome

We utilized both *de novo* and homology-based approaches to annotate the repetitive sequence in the *S. angustifolia* genome. A total of 319.98 Mb of repetitive sequences were identified, accounting for 50.70% of the *S. angustifolia* genome (Table 1). Among these, long terminal repeats (LTRs) were the most abundant, accounting for 42.91% of the genome, followed by DNA transposons (2.49% of the genome) and long interspersed nuclear element (LINE) (1.64% of the genome) (Table 1, Table S6, Table S7).

To assist in the prediction of protein-coding genes, transcriptome sequencing was performed on the roots, stems, leaves, flowers, and seeds of *S. angustifolia*, generating 40.40 Gb of clean data (Table S8). Transcriptome assembly was performed using the reference-based and *de novo* approaches, with transcripts from both methods incorporated into the prediction of protein-coding genes. Based on *ab initio* prediction, homology, and transcriptomic evidence, 36,857 protein-coding genes were identified in the *S. angustifolia* genome, with an average exon length of 242.95 base pairs (bp) and an average intron length of 465.57 bp (Table 1). The completeness of the gene set was assessed using BUSCO, revealing a completeness score of 97.71% (92.50% single-copy BUSCOs and 5.20% duplicated BUSCOs) (Table S9). Furthermore, functional annotation indicated that 98.94% of the predicted genes were annotated, with 68.80% and 65.07% being annotated in the Kyoto Encyclopedia of Genes and Genomes (KEGG) and Gene Ontology (GO) databases, respectively (Table S10). Additionally, we identified non-coding RNAs (ncRNAs) within the *S. angustifolia* genome, including 97 miRNAs, 3,048 snRNAs, and 573 tRNAs (Table S11).

Comparative Genomic Analysis Among Leguminous Plants and Arabidopsis

To investigate the evolutionary relationships of *S. angustifolia*, we conducted gene family and phylogenetic analyses on nine leguminous species and the model plant Arabidopsis. Gene clustering analysis identified 31,810 orthologous groups (OGs) across the studied species, with 9,460 OGs shared by all species. Additionally, 633 OGs were identified as single-copy, while 578 OGs were found to be specific to *S. angustifolia* (Figure 2a).

A phylogenetic tree based on the single-copy OGs, revealed that *S. angustifolia* is closely related to the wild relatives of peanuts, *A. duranensis* and *A. ipaensis*, forming sister branches (Figure 2b). This relationship further supports the evolutionary position of *S. angustifolia* within the

subtribe *Stylosanthinae* (Benth.) of the legume family. Furthermore, to explore the dynamics of gene families, we performed gene families expansion and contraction analyses on six well-annotated and extensively studied legumes. This analysis identified 2,089 expanded gene families in *S. angustifolia*, with 158 OGs showing significant expansion (*P*-value < 0.05) (Figure S4). These 158 OGs contained 3,071 genes primarily involved in KEGG pathways related to "carbohydrate metabolism", "Biosynthesis of other secondary metabolites", and "lipid metabolism" (Figure S5).

We also identified collinear gene blocks within and between *S. angustifolia*, soybean, *A. duranensis*, and *A.ipaensis*. The genomes of *S. angustifolia*, *A. duranensis*, and *A.ipaensis* exhibit extensive genomic rearrangements, especially on chromosomes chr3, chr9, and chr10. However, some chromosomal regions maintained strong synteny, such as chromosomes chr1, chr6, and chr8 (Figure 2c). The synonymous substitution rate (Ks) distribution of collinear gene pairs revealed that *S. angustifolia* shared the ancestral Papilionoideae whole-genome duplication event (PWGD) with soybean, *A. duranensis*, and *A. ipaensis* (Figure 2d), with similar Ks peaks for *S. angustifolia*, *A. duranensis*, and *A. ipaensis* (Ks peak ~ 0.8, estimated time at 49.26 million years ago, MYA) (Figure 2d). Furthermore, the divergence time between *S. angustifolia* and the two *Arachis* species estimated to be 11 MYA, consistent with divergence time derived from single-copy gene family analysis (Figure 2b, 2d).

Transcriptome Analysis of *S. angustifolia* in Response to Drought Stress

To assess the response of *S. angustifolia* to drought stress, 60-day-old seedlings were subjected to drought treatments for 0 days (D0, control), 3 days (D3), and 5 days (D5) under pot conditions. As the duration of drought treatment increased, the leaves of *S. angustifolia* progressively turned yellow (Figure 3a, b), accompanied by a gradual decrease in chlorophyll a and b content (Figure S6a, b), as well as in shoot water content (Figure S6c). In addition, soil water content decreased by 62.16% at D3 and by 81.91% at D5 relative to D0 (Figure S6d). To further understand the gene expression dynamics under drought stress, transcriptome sequencing was conducted on the D0, D3, and D5 samples from the roots and leaves (Figure 3a). This yielded a total of 130.05 Gb clean data (average 7.22 Gb per sample) with Q30 greater than 93.54% (Table S12). Gene expression analysis revealed that 29,215 genes were expressed during the drought experiment. Additionally, differential gene

expression analysis showed that more genes were differentially expressed in roots and leaves after 5 days of drought treatment compared to 3 days (Figure S7). Among these, 384 genes were up-regulated in both roots and leaves after 3 days of drought treatment, while 1,246 genes were up-regulated in roots and leaves after 5 days of drought treatment (Figure S8).

An intersection analysis between the expanded gene families and the differentially expressed genes (DEGs) revealed 98 up-regulated genes in the root and 84 in the leaves after 3 days of drought treatment (Figure 3c, d). After 5 days of drought treatment, 155 genes in the roots and 166 genes in the leaves were up-regulated (Figure 3e, f). Enrichment analysis of these expanded and up-regulated genes revealed significant enrichment in the "carotenoid biosynthesis pathway" (map00906, *Q*-value < 0.05) in both roots and leaves (Figure 3g, Table S13 - S16). Further analysis highlighted the role of *xanthoxin dehydrogenase* (*ABA2*, K09841), a key gene family involved in ABA biosynthesis, with significant up-regulated in both roots and leaves under drought stress (Table S14 - S16). Genome-wide identification of *ABA2* genes in *S. angustifolia*, soybean, barrel medic, and Arabidopsis revealed a higher number of *ABA2* genes in the studied leguminous plants compared to Arabidopsis (Table S17). Notably, *ABA2* gene underwent tandem duplication, resulting in the expansion of seven *ABA2* genes on chromosome 4 of *S. angustifolia* (Figure 4a, b). Divergence time analysis indicated these duplications occurred approximately 24.13 MYA, with the most recent duplication around 5.01 MYA (Table S18). Among the seven tandem duplicated *ABA2* genes, six were up-regulated in the roots and four in the leaves after 5 days of drought treatment (Figure 4a). Quantification of ABA content revealed a 20.43-fold increase in the roots and a 5.05-fold increase in the leaves at D5 compared to D0 (Figure 4d, e), suggesting that the expansion and up-regulation of *ABA2* genes contributed to ABA biosynthesis under drought stress.

In addition, genes expanded and up-regulated in the leaves after 5 days of drought treatment were significantly enriched in the "Isoflavonoid biosynthesis pathway" (map00943) (*Q*-value < 0.05) (Figure 3g). Further analysis identified three *2-hydroxyisoflavanone dehydratase* (*HIDH*, K13258) genes involved in genistein and daidzein biosynthesis (Table S16). Phylogenetic and microsynteny analyses indicated a specific expansion event of *HIDH* genes on chromosome 2 through tandem duplication (Figure 5a, b, Table S19), with the most recent duplication occurring at 11.11 MYA (Table S20). Three *HIDH* genes were up-regulated in the leaves after 5 days of drought treatment

(Figure 5a). Consistent with the transcriptome results, compared to D0, the contents of genistein and daidzein in the leaves increased by 448% and 94% at D5, respectively (Figure 5d, f). However, the contents of genistein and daidzein in the roots showed no significant differences between D0 and D5 (Figure 5c, e).

Contribution of TDGs to Drought Tolerance of *S. angustifolia*

The *S. angustifolia* genome shows no evidence of recent WGDs (Figure 2d), and gene family analysis revealed that tandem duplication plays an important role in gene expansion within the *S. angustifolia* genome (Figure 3, Figure 4, Figure 5). Therefore, we performed a genome-wide identification of TDGs and investigated their response to drought stress in *S. angustifolia*. As a result, we identified 3,634 TDGs in the *S. angustifolia* genome, which is higher than in *A. duranensis* (2,735) and *A. ipaensis* (3,449), but lower than in soybean (5,022) and barrel medic (7,032). By analyzing the Ks distribution of TDGs, we found substantial expansion of TDGs in *S. angustifolia* approximately 12.31 MYA, with a Ks peak at about 0.2 (Figure 6a).

TDGs in *S. angustifolia* were significantly enriched in KEGG pathways including "Biosynthesis of secondary metabolites", "Flavonoid biosynthesis", "Isoflavonoid biosynthesis", and "Galactose metabolism", among others (*Q*-value <0.05). (Figure 6b). Transcriptomic analysis revealed that three gene families involved in the biosynthesis of raffinose and stachyose in the galactose metabolism pathway—inositol 3-alpha-galactosyltransferases (*GOLSs*), raffinose synthases (*RAFSs*), and stachyose synthetases (*STSs*)—exhibited significantly increased expression following 3 and 5 days of drought treatment, with the most pronounced up-regulation occurring after 5 days (Figure 6c, Table S21). Notably, the two *STS* genes, which were significantly up-regulated in the roots after 5 days of drought treatment, were expanded through tandem duplication (Figure 6c, d).

Consistent with gene expression patterns, the raffinose content increased by 129% in the roots and by 225% in the leaves at D5 compared to D0 (Figure 6e, f). Similarly, the stachyose content increased by 142% in the roots and by 99% in the leaves at D5 compared to D0 (Figure 6g, h).

Lipid Metabolism in *S. angustifolia* in Response to Drought Stress

Comparative genomic analysis revealed that significantly expanded gene families in *S. angustifolia* are involved in the lipid metabolism pathway (Figure S5). To evaluate the effects of drought stress on lipid metabolism in *S. angustifolia*, we conducted a lipidomic analysis on the D0 and D5 samples from the roots and leaves. A total of 874 lipids in the roots and 904 lipids in the leaves, belonging to six major lipid classes, were identified (Figure 7a, b, Table S22, Table S23). Of these, 52 lipids in the roots and 134 lipids in the leaves were identified as differentially accumulated lipids (DALs) at D5 compared to D0 (Figure 7a, b). Among the DALs, 22 triacylglycerols (TAGs) were detected in the roots and 29 TAGs in the leaves, with 81.81% of the TAGs in the roots and 89.66% of the TAGs in the leaves being up-regulated after 5 days of drought treatment (Figure 7c, d). In plants, TAGs associate with membrane protein families, including oleosins, caleosins, and steroleosins, to form subcellular organelles known as oil bodies, which play a crucial role in regulating lipid metabolism and maintaining lipid homeostasis [45]. With the accumulation of TAGs at D5, two of the seven oleosin family genes were up-regulated in the roots of *S. angustifolia*, while four were up-regulated in the leaves (Figure 7e, Table S24). Similarly, after 5 days of drought treatment, all four oleosin family genes in *S. angustifolia* were up-regulated in the roots, while three of the four were up-regulated in the leaves (Figure 7e, Table S24). Notably, two of the up-regulated oleosin genes resulted from tandem duplications (Figure 7f).

In plants, the degradation of membrane lipids containing C18:3 chains produce α-linolenic acid, which serves as a precursor for the synthesis of jasmonic acid (JA) [46]. Lipidomic analyses revealed a significant reduction in four phospholipids, two sulfolipids, twelve galactolipids, and one glucolipid containing C18:3 chains in the leaves after 5 days of drought stress (Figure 8a). In contrast, the roots exhibited a significant decrease in only one phospholipid and one galactolipid containing C18:3 chains after 5 days of drought stress (Figure 8b). Consistent with this finding, drought treatment for 5 days led to the up-regulation of several gene families involved in the biosynthesis of JA and jasmonoyl-L-isoleucine (JA-Ile) in the leaves (Figure 9a). Specifically, four members of *patatin-related phospholipase A* (*pPLA*), one member of *phospholipase A1* (*DAD1*), three members of *acyl-CoA oxidase* (*ACX*), three members of *multifunctional protein* (*MFP*), two members of *ketoacyl-CoA thiolase* (*KAT*), and one member of *jasmonate-amido synthetase* (*JAR*) were up-regulated (Figure 9a). Interestingly, *pPLA* underwent tandem duplication approximately

18.67 MYA (Ks = 0.30) (Figure 9b). Quantitative measurements of JA and JA-Ile showed that their contents significantly increased in the leaves, with JA levels increasing by 77% and JA-Ile levels increasing by 447% at D5 compared to D0 (Figure 9d, f). In contrast, JA and JA-Ile levels were significantly reduced in the roots at D5 compared to D0 (Figure 9c, e).

Discussion

Exploring the mechanisms by which pioneer plants adapt to harsh environmental conditions offers valuable insights for enhancing stress tolerance traits in crops [47,48]. Despite the pioneer plant *S. angustifolia* being recognized for its exceptional drought tolerance, the absence of high-quality genomic resources has considerably impeded a comprehensive understanding of its molecular mechanisms for drought resistance. In this study, we report the T2T genome assembly of *S. angustifolia*, achieved by integrating NGS, ONT, and Hi-C sequencing technologies, yielding a genome size of 631.17 Mb. The genome assembly demonstrated high integrity and accuracy, as confirmed by multiple quality assessment metrics (Table 1 and Figure 1). Notably, the *S. angustifolia* genome is relatively simple, exhibits low heterozygosity (0.06%), which facilitated the successful assembly of a T2T genome using only ONT, NGS, and Hi-C data (Figure S2, Table 1).

The T2T genome facilitated the exploration of telomeric and centromeric regions in *S. angustifolia*. We identified 17 of the 20 telomeric sequence were identified, while an ITS was detected on chromosome 4 (Table S4 and Figure S3). ITSs are commonly found in plant genomes and are typically the result of historical chromosomal rearrangements, duplications, or other structural variations. These internal repeats do not function as telomeres and are instead remnants of past genomic events [49,50]. Further validation of this ITS is necessary, and comparative genomics of related species could offer additional insights. Moreover, we identified and characterized centromeric regions in the *S. angustifolia* genome, which exhibited a slightly higher GC content than the average GC content of the genome (Table 2). The T2T genome provides a valuable resource for evolutionary, genetic, and functional studies, particularly in molecular breeding programs targeting drought tolerance.

Gene duplication plays a crucial role in plant evolution and environmental adaptation [34,35]. Particularly, tandem duplication is a significant characteristic in secondary metabolite biosynthesis

pathways and in response to biotic and abiotic stresses [51,52]. For instance, the expansion of sugar metabolism-related genes *α-amylase* (*AMY3*) and *β-fructofuranosidase* (*CWINV1*) in *Sophora moorcroftiana* contributes to high sucrose content, promoting long root growth and enhancing drought tolerance [53]. In orchardgrass (*Dactylis glomerata*), the DgMADS-box genes have expanded via tandem duplication, contributing to longer root lengths and higher survival rates under various abiotic stresses [54]. Similarly, the RWP-RK gene family in the Pearl millet (*Pennisetum glaucum*) genome has expanded, enabling rapid responses to heat stress by regulating the expression of endoplasmic reticulum (ER)-related genes [55]. These examples highlight the significance of TDGs in environmental adaptation.

In *S. angustifolia*, TDGs played a crucial role in its drought adaptation, as demonstrated by an integrative comparative genomics, transcriptomics, and metabolomics analysis. ABA, a key phytohormone involved in the drought stress response [56–58], exhibited significantly increased accumulation in the leaves and roots of *S. angustifolia* following 5 days of drought treatment (Figure 4d, e). The expansion of *ABA2* genes through tandem duplication in the genome of *S. angustifolia* may contribute to increased ABA biosynthesis under drought stress (Figure 4a, b). Additionally, JA and its active conjugate, JA-Ile, are important plant hormones that are widely distributed in plants and involved in resistance to biotic and abiotic stresses, including drought [5,59–63]. Our study found that the coding genes for pPLAs, which degrade C18:3-branched membrane lipids, have expanded through tandem duplication and may enhance the biosynthesis of JA and JA-Ile in the leaves of *S. angustifolia* under drought conditions (Figure 9).

Raffinose family oligosaccharides (RFOs), which are *α*-galactosyl derivatives of sucrose, including raffinose, stachyose, and verbascose, are prevalent in plants and play important roles in regulating plant responses to abiotic stress [64]. In maize, drought treatment increases raffinose accumulation. Overexpression of *ZmRAFS*, responsible for raffinose biosynthesis, enhances drought tolerance in transgenic plants [6,7]. Similar results were observed in *S. angustifolia*, where *RAFS* family genes were up-regulated in both leaves and roots under drought conditions, accompanied by an increase in raffinose accumulation (Figure 6c, e, f). Raffinose can be further synthesized into stachyose. We found that stachyose accumulation increased in the leaves and roots of *S. angustifolia* under drought conditions (Figure 6g, h). In addition, the *STS* genes responsible for stachyose

biosynthesis in *S. angustifolia* underwent tandem duplicative expansion and were upregulated under drought conditions (Figure 6c, d), which may contribute to the drought tolerance mechanism characteristic of *S. angustifolia*. Apart from RFOs, isoflavonoids, such as genistein, have been shown to contribute to enhancing plant drought resistance [4, 66]. The *HIDH* genes, responsible for the biosynthesis of genistein and daidzein, have undergone expansion through tandem duplication in *S. angustifolia* (Figure 5b). After 5 days of drought treatment, the accumulation of genistein and daidzein in the leaves of *S. angustifolia* increased (Figure 5d, f), likely due to the up-regulation of the *HIDH* genes (Figure 5a), suggesting their active role in drought tolerance.

In conclusion, the T2T genome assembly of *S. angustifolia* provides new insights into the genetic basis of drought tolerance. Our study highlights the significant role of tandem duplication in the expansion of key gene families involved in phytohormone biosynthesis, secondary metabolism, and osmoprotection, which together enhance the drought adaptation of *S. angustifolia*. These findings advance our understanding of the molecular mechanisms underlying drought tolerance and offer valuable genomic resources for breeding drought-tolerant crops.

Methods

Plant Materials

S. angustifolia (Germplasm number: TF0003, Figure S1) was provided by the National Tropical Plants Germplasm Resource Center (Hainan, China). Young leaves were harvested for DNA extraction using the CTAB method for both NGS and ONT sequencing. Roots, stems, leaves, flowers, and seeds were collected for RNA extraction and transcriptome sequencing.

Genome Sequencing and Assembly

High-quality genomic DNA was extracted for the construction of ONT and NGS libraries. Sequencing was performed at the Genome Center of Grandomics (Wuhan, China). The ONT library was sequenced using the ONT PromethlON platform, while the NGS library was sequenced using the MGI-SEQ 2000 platform (MGI Tech, China). Hi-C technology was adopted for chromosome-level genome assembly. Hi-C library construction and sequencing were performed at the Genome

Center of Grandomics (Wuhan, China) using DPN II as the restriction enzyme. Guppy (v6.5.7) was adopted for ONT reads processing and SOAPnuke (v2.1.7) was adopted for quality control of raw sequencing reads of NGS and Hi-C with the parameters '-n 0.01 -l 20 -q 0.3 --polyX 50'. ONT reads were corrected and assembled into initial contigs using NextDenovo (v2.3.0) [67], with "read_cutoff" set to 2k to ensure the high-quality assembly results. The initial contigs were further polished using NextPolish (v1.4.1) [68], incorporating both NGS and ONT reads. Clean Hi-C reads were aligned to the polished contigs using the Burrows-Wheeler Aligner (BWA, v0.7.17), and Hi-C contact maps were generated using Juicer (v1.6). The 3D de novo assembly (3D-DNA, v180922) pipeline was adopted for chromosomal grouping, sorting, and orientation. Visualization and manual curation of Hi-C maps were conducted using Juicebox (v1.11.08) to ensure the accuracy of chromosome assembly. During this process, 61 shorter contigs (14.34 Mb in length) exhibited no discernible Hi-C crosslinking signals. Given that these contigs could not be reliably anchored to specific chromosomal locations, and are likely to represent low-complexity sequences, repetitive elements, potential contaminants, or assembly artifacts, they were cautiously removed. This step was taken to improve the overall accuracy and reliability of the assembly, minimizing the risk of potential misassemblies affecting downstream analyses. Subsequently, LR_Gapcloser (https://github.com/CAFS-bioinformatics/LR_Gapcloser) [69] was employed to filled 95 gaps across the chromosomes based on the raw ONT reads, leaving only one gap in chromosome 7. The completeness of the genome assembly was evaluated using BUSCO (v5.3.2) based on embryophyta_odb10 database, and LAI analysis using LTR_retriever (v2.9.0) [70]. Merqury (v1.3) was adopted for assembly completeness evaluation using an efficient k-mer set. Reads were aligned to the genome using minimap2 (v2.17-r941) for ONT and BWA (v0.7.17) for NGS. SAMtools (v1.9) and PanDepth (v2.19) were adopted for genome mapping ratios and coverage statistics. Based on the NGS mapping result, GATK (v4.1.2.0) were employed to detected SNPs. The heterozygosity rate of *S. angustifolia* was calculated as the number of heterozygous SNPs divided by the number of effective genome bases multiplied by 100.

Genome Annotation

We performed both *de novo* and homologue-based approaches for repetitive element identification.

Extensive *de novo* TE Annotator (EDTA, v2.0.1) [71] was adopted for *de novo* identification of transposable elements (TEs). Additionally, known repetitive elements from RepBase (v21.12) were identified by employing RepeatMasker (v4.1.4). Tandem Repeat Finder (TRF, v. 4.09.1) was employed to detect the tandem repeats.

Protein-coding gene prediction was performed based on the repeat-masked genome by employing *de novo*-based prediction, RNA-seq-based prediction, and homologue-based prediction. We employed GALBA (https://github.com/Gaius-Augustus/GALBA, v1.0.8) for automated training and prediction of protein-coding genes, utilizing AUGUSTUS (https://github.com/Gaius-Augustus/Augustus, v3.5.0) and miniport (https://github.com/lh3/miniprot, v0.13) with default parameters. SNAP (v. 2013-02-16) was also adopted for *de novo* gene prediction. Transcriptome sequencing reads from roots, stems, leaves, flowers, and seeds were aligned to the *S. angustifolia* genome using HISAT2 (v2.2.1), and transcript construction was conducted using StringTie (v2.2.1) [72]. RNA *de novo* assembly was performed using Trinity (v2.15.1). Coding regions of the predicted transcripts were identified using TransDecoder (v 5.7.0). For homology-based prediction, proteins from five species, namely, *G. max*, *Medicago truncatula*, *A. ipaensis*, *Senna tora*, and *Arabidopsis thaliana*, were aligned to the *S. angustifolia* genome using tBLASTn (v2.13.0) and gene structure prediction were performed by miniport (v0.13). EVidenceModeler (v2.1.0, https://github.com/EVidenceModeler/EVidenceModeler) was adopted for the identification of non-redundance consensus genes from all available evidence. Furthermore, PASA (v2.5.3) was adopted to refine gene structure and annotate untranslated regions (UTRs) based on transcriptome data. The predicted protein-coding genes were aligned to various known databases, including NCBI Non-Redundant Protein Sequence Database (NR), KEGG, Eukaryotic Orthologous Groups of Protein (KOG), Swiss-Prot, TrEMBL, InterPro databases, for functional annotation. BLASTp (v2.13.0) was adopted for homology search against NR, KEGG, KOG, Swiss-Prot, and TrEMBL with parameters '-outfmt 6 -evalue 1e-10'. Blast2GO (v6.0) was adopted for GO annotation based on the NR annotation, The best hit for each gene was retained for subsequent analysis. Additionally, non-coding genes (ncRNAs) were predicted by BLASTn (v2.13.0) and INFERNAL (v1.0) based on the Rfam database (v12.0).

Identification of Telomeres and Centromeres

We employed a Telomere Identification Toolkit (tidk, https://github.com/tolkit/telomeric-identifier) for telomere identification by searching the sequence "TTTAGGG" repeat motif. Centromeric regions, characterized by a high density of short tandem repeats and low gene density, were identified using CentIER software (v3.0) [73] by analyzing sequence features such as tandem repeat sequences, retrotransposons, and k-mer frequency distribution. Additionally, we generated k-mer distribution maps for each chromosome to assist in the identification and verification of centromeric regions.

Comparative Genomic Analysis

Nine legumes, including *S. angustifolia, A. ipaensis*, *Arachis duranensis*, *Aeschynomene evenia*, *G. max*, *M. truncatula*, *Phaseolus vulgaris*, *Cajanus cajan*, and *Senna tora*, along with model species, *Arabidopsis thaliana*, were utilized for comparative analysis. Protein sequences from these species were compared through all-vs-all alignments using BLASTp (v2.2.23, e-value set to 1e-5). OrthoFinder (v2.5.4) was utilized for orthologous groups (OGs) identification and phylogenetic tree construction with parameters '-S diamond -M msa -A mafft'. Divergence times between *G. max*, *M. truncatula*, *C. cajan*, and *P. vulgaris* were queried on TimeTree (http://www.timetree.org/) as known divergence times. Based on single-copy OGs, the substitution rates were estimated using the MCMCTREE program within the PAML (v4.5) software package, further calculating the divergence times between species. MCScanX (https://github.com/wyp1125/MCScanX) was employed for intra- and inter-species gene collinearity analysis. First, protein sequences were aligned using BLASTp (v2.2.23, e-value set to 1e-5), and MCScanX was employed to identify collinear regions. For intra-species analysis, we employed the duplicate_gene_classifier from MCScanX, to classify paralogous genes into categories, including single-copy genes, dispersed duplicated genes, proximal duplicated genes, TDGs, and whole genome or segmental duplicated genes. Microcollinearity of TDGs in *S. angustifolia* genome compared with soybean and Medicago was performed using MCscan-(Python-version) (https://github.com/tanghaibao/jcvi). Additionally, the non-synonymous (Ka) and synonymous (Ks) substitution rates of gene pairs within the collinear regions, as well as the Ka and Ks of TDGs were calculated using PAML (v4.9e) and PAL2NAL

(v14) using the Nei-Gojobori (NG) method [74]. The R platform (v4.0.2) was adopted for Ks distribution visualization and peak identification. The divergence time of gene pairs was calculated using the formula T=Ks/2r, where the neutral substitution rate r was selected as 8.12×10^{-9} in this study [75].

Gene Identification and Phylogenetic Analysis

Protein sequences from *S. angustifolia*, soybean, and barrel medic were aligned against The Arabidopsis Information Resource (TAIR) database. Genes with an identity greater than 35% and coverage exceeding 50% were retained for further analysis of metabolic pathways. For the phylogenetic analysis, protein sequences from studied gene families were aligned using MUSCLE (v5.2) and phylogenetic tree was conducted using FastTree version (v2.1.11) with the approximately-maximum-likelihood. Visualization of the resulting phylogenetic trees was performed using iTOL (v6, https://itol.embl.de/).

Drought Treatment on *S. angustifolia*

After germination, seeds of *S. angustifolia* were planted in pots (30 cm in height, 25 cm in diameter) filled with sandy soil. Drought treatment was imposed on the 60-day-old seedlings by stopping watering. Seedlings were sampled by collecting leaves and roots at day 0 (D0) for the control group, and at days 3 (D3) and 5 (D5) after the drought treatment.

Soil water content was determined using previously reported methods [76–78]. Briefly, after harvesting the plants, the total soil in each pot was recorded to obtain the wet weight (Ww). The soil was then dried at 65°C for 72 hours to obtain the dry weight (Wd). The soil water content was calculated using the formula: (Ww – Wd) / Ww ×100%. The soil water content at D0 was normalized to 100% of pot capacity (PC), while the corresponding soil water contents at D3 and D5 were expressed as percentages of this normalized value. Shoot water content was measured according to the methods previously reported [79]. In brief, the shoot was harvested and weighed to obtain the fresh weight (FW). After drying at 65°C for 48 hours to a constant weight, the tissues were weighed again to determine the dry weight (DW). The water content, expressed as a percentage of fresh weight (% FW), was calculated using the formula: (FW – DW) / FW ×100%. Chlorophyll content was measured according to previously published methods [80]. Three biological replicates

of root and leaf samples were collected for RNA sequencing, metabolite quantification, and lipidomics analysis.

Transcriptome Sequencing and Bioinformatics Analysis

RNA was extracted from root and leaf samples collected at D0, D3, and D5 during the drought treatment for transcriptome sequencing. Each treatment group in this experiment consisted of three biological replicates, with each biological replicate containing 20 seedlings. Transcriptomes were sequenced on the MGI-SEQ 2000 platform (MGI Tech, China) and SOAPnuke (v2.1.7) [81] was adopted for quality controlling of the raw sequencing reads. Clean reads were mapped onto the *S. angustifolia* genome using HISAT2 (v2.2.1). The featureCounts (v2.0.6) were employed for the calculation of gene read counts and an in-house Perl script was adopted for (transcripts per million, TPM) calculation. DEGs were identified using DESeq2 (v3.19), and the BH method was adopted for false discovery rate (FDR) calculation. Genes with $|\log_2$ fold change$| > 1$ and $Padj < 0.05$ were considered as DEGs. KEGG pathway enrichment analysis of DEGs was performed by the phyper and *p.adjust* functions under the R platform (v4.0.2) and KEGG pathways with a Q-value < 0.05 were considered as significantly enriched pathways. Gene expression heatmaps were generated under the R platform (v4.0.2).

Lipidomics Analysis of *S. angustifolia* under Drought Stress

Lipidomics analysis was conducted on root and leaf samples of *S. angustifolia* by Biotree Biomedical Technology Co., Ltd. (Shanghai, China). Sample extraction was performed with slight modifications to our previously reported method [82]. Briefly, freeze-dried samples were extracted using the flowing extracting solution: MTBE: MeOH= 5:1 (v/v) containing an isotope-labeled internal standard. Subsequently, 100 μL of the extracted supernatant was transferred to the injection bottle for lipid metabolite detection.

The chromatographic separation of the target compounds was performed using a Phenomenex Kinetex C18 column (2.1 mm × 100 mm, 2.6 μm) on a Vanquish ultra-performance liquid chromatograph (Thermo Fisher Scientific). The mobile phase A consisted of 40% water and 60% acetonitrile with 10 mmol/L ammonium formate, while phase B comprised 10% acetonitrile and 90%

isopropanol, supplemented with 50 mL of 10 mmol/L ammonium formate aqueous solution per liter. The injection volume was set at 2 μL. Mass spectrometric analysis was performed on an Orbitrap Exploris 120, allowing for both primary and secondary mass spectrometry data acquisition using Xcalibur (v4.4). The operational parameters were: Sheath gas flow rate at 30 Arb, auxiliary gas flow rate at 10 Arb, capillary temperature at 320 °C (both positive and negative modes), full MS resolution at 60,000, MS/MS resolution at 15,000, collision MS resolution at 15,000, collision energy at 15/30/45 in NCE mode, and spray voltage at 3.8 kV (positive) or -3.4 kV (negative).

The raw mass spectrum data were converted to mzXML format using ProteoWizard software. XCMS was then used for retention time correction, peak identification, extraction, integration, and alignment. The minimum fraction (minfrac) was set to 0.5 and cutoff was set to 0.3. Lipid identification was performed through a spectral match using the LipidBlast library within the XCMS software [83].

MetaboAnalyst (v6.0) [84] was adopted for lipids analysis. Lipids with a fold change of ≥ 2 or ≤ 0.5 in relative abundance between D5 and D0, along with variable importance for projection (VIP) score > 1 and an adjusted *P*-value (*Padj*) < 0.05, were identified as differentially accumulated lipids (DALs). Each experimental group had three biological replicates.

Determination of ABA, JA and JA-Ile, Genistein, Daidzein, Raffinose, and Stachyose

Quantitative assays of plant hormones (ABA, JA, and JA-Ile) in the roots and leaves of *S. angustifolia* were conducted by Biotree Biomedical Technology Co., Ltd. (Shanghai, China). A total of 100 mg of the freeze-dried samples was weighted and extracted using 1 mL of ice-cold 50% acetonitrile (ACN) aqueous solution. The sample was sonicated at 4°C for 3 minutes, followed by extraction at 4°C for an additional 30 minutes. The mixture was centrifuged at 12,000 rpm for 10 minutes at 4°C, and the supernatant was collected. The sample was then passed through an RP-SPE column: 1 mL of 100% methanol (MeOH) and 1 mL of deionized water were added, then equilibrate the column with 50% ACN aqueous solution. The sample was loaded onto the column, which was washed with 1 mL of 30% ACN, and the eluent was collected. The sample was evaporated to dryness under a nitrogen stream, dissolved in 200 μL of 30% ACN, and transferred to a sample vial with an insert.

The data acquisition system primarily consisted of ultra-high-performance liquid chromatography (UPLC, Vanquish, Thermo, USA) coupled with a high-resolution mass spectrometer (Q Exactive, Thermo, USA). The liquid chromatography parameters were set as follows: chromatographic column: Waters HSS T3 (50 × 2.1 mm, 1.8 μm); mobile phase: phase A was ultrapure water (containing 0.1% acetic acid), and phase B was acetonitrile (containing 0.1% acetic acid); flow rate: 0.3 mL/min; column temperature: 40°C; injection volume: 2 μL; elution gradient: 0 min water/acetonitrile (90:10, v/v), 1 min water/acetonitrile (90:10, v/v), 5 min water/acetonitrile (10:90, v/v), 7 min water/acetonitrile (10:90, v/v), 7.1 min water/acetonitrile (90:10, v/v), 9 min water/acetonitrile (90:10, v/v). During the entire analysis, samples were kept in an auto-sampler at 4°C. To avoid signal fluctuation impacts, samples were analyzed in random sequence. QC samples were inserted into the sample queue to monitor and evaluate system stability and data reliability. Data acquisition was performed using the Q Exactive high-resolution mass spectrometer (Thermo Fisher Scientific, USA). The electrospray ionization (ESI) conditions were as follows: sheath gas 40 arb; auxiliary gas 10 arb; spray voltage 3000V; temperature 350°C; ion transfer tube temperature 320°C. The scan mode was set to single ion monitoring (SIM) in positive ion mode. with a primary scan m/z range was 100-500. Mass spectrometry data were processed using TraceFinder software.

Quantitative assays of raffinose and stachyose in the roots and leaves of *S. angustifolia* were conducted by Biotech-Pack-Analytical Inc. (Beijing, China). A total of 300 mg of freeze-dried samples was weighted and extracted twice with 5 mL of 80% ethanol at 85°C for 30 minutes each time. Following each extraction, the mixture was centrifuged at 12,000 rpm for 5 minutes. The combined supernatants were collected and evaporated to dryness using a vacuum centrifuge. The resulting dried residue was resuspended in 300 μL of distilled water and centrifuged again at 12,000 rpm for 10 minutes. The final supernatant was collected for high-performance liquid chromatograph (HPLC) analysis. The liquid chromatography analysis was performed using a Waters 2695 HPLC coupled with a Waters 2424 evaporative light-scattering detector (ELSD). The chromatographic conditions were as follows: column temperature: 40 °C; flow rate: 1.0 mL/min; injection volume: 3 μL of sample; chromatographic column: Sepax HP-Amino (4.6 x 250 mm, 5 μm, 120 Å); mobile phase: acetonitrile: water (70:30) with isocratic elution; total run time: 20 minutes.

Quantitative assays of genistein and daidzein in the leaves and roots of *S. angustifolia* were conducted as described in our previous report [82].

Abbreviations

Gb: gigabase; Mb: megabase; Bp: base pair; T2T: telomere-to-telomere; ABA: abscisic acid; TGS: third-generation sequencing; ONT: Oxford Nanopore Technologies; NGS: next-generation sequencing; TDG: tandem duplicated gene; WGD: whole-genome duplication; Hi-C: high-through chromosome conformation capture; GATK: genome analysis toolkit; ITS: interstitial telomeric sequence; BUSCO: Benchmarking Universal Single-Copy Orthologs; LAI: LTR assembly index; LTR: long terminal repeat; KEGG: Kyoto Encyclopedia of Genes and Genomes; GO: Gene Ontology;OG: orthologous group; MYA: million years ago; DEG: differentially expressed gene; HIDH: 2-hydroxyisoflavanone dehydratase; ABA2: xanthoxin dehydrogenase; GOLS: inositol 3-alpha-galactosyltransferases; RAFS: raffinose synthases; STS: stachyose synthetases; DALs: differentially accumulated lipids; TAG: triacylglycerol; JA: jasmonic acid; JA-Ile: jasmonoyl-L-isoleucine; pPLA: patatin-related phospholipase A; DAD1: phospholipase A1; ACX: acyl-CoA oxidase; MFP: multifunctional protein; KAT: ketoacyl-CoA thiolase; JAR: jasmonate-amido synthetase; AMY3: α-amylase; CWINV1: β-fructofuranosidase; EDTA: Extensive de novo TE Annotator; UTR: untranslated region; NR: NCBI Non-Redundant Protein Sequence Database; KOG: Eukaryotic Orthologous Groups of Protein; Ka: non-synonymous; Ks: synonymous.

Data Availability

The raw genomic sequencing data, including ONT, NGS, and Hi-C data, as well as transcriptome data, have been deposited in the National Genomics Data Center (NGDC, https://ngdc.cncb.ac.cn) under BioProject PRJCA027610. The raw sequence data have been deposited in the Genome Sequence Archive in the National Genomics Data Center, China National Center for Bioinformation / Beijing Institute of Genomics, Chinese Academy of Sciences (GSA: CRA017744) that are publicly accessible at https://ngdc.cncb.ac.cn/gsa. The assembly and annotation of *S. angustifolia* have been deposited in the Genome Warehouse in the National Genomics Data Center under accession number GWHEUEA00000000.1, which is publicly accessible at https://ngdc.cncb.ac.cn/gwh. The raw

sequencing data can also be accessed through NCBI under the accession number PRJNA1140667.

Competing Interests

The authors declare that they have no competing interests.

Author Contributions

P.L. conceived the project and designed the experiments. C.L. performed genome assembly, annotation, transcriptome, and lipidome analyses. G.L. provided funding and *S. angustifolia* germplasm. L.L. performed supervision. J. Z., R. X., and J. L. planted and collected samples. S.Z., M.B., and Z.Q. provided technical support and suggestions on manuscript revision. C.L. performed the manuscript. P.L. revised the manuscript. All authors read and approved the final manuscript.

Funding

The research was financially supported by the Natural Science Foundation of Hainan Province (323CXTD387), the National Natural Science Foundation of China (32371769), the Earmarked fund for China Agriculture Research System—Forage and Grass (CARS-34), the Earmarked fund for China Agriculture Research System—Green Manure (CARS-22), the Central Public-interest Scientific Institution Basal Research Fund for CATAS (1630032022023 and 1630032024016), and the Guangxi Special Project for Innovation-driven Development (Guike AA18242040).

References

1. Gupta A, Rico-Medina A, Caño-Delgado AI. The physiology of plant responses to drought. Science 2020;368:266–269. https://doi.org/10.1126/science.aaz7614.

2. Vadez V, Grondin A, Chenu K, et al. Crop traits and production under drought. Nat Rev Earth Environ 2024;5:211–225. https://doi.org/10.1038/s43017-023-00514-w.

3. Wang X, Li Q, Xie J, et al. Abscisic acid and jasmonic acid are involved in drought priming-induced tolerance to drought in wheat. Crop Journal 2021;9:120–132. https://doi.org/10.1016/j.cj.2020.06.002.

4. Yang W, Li N, Fan Y, et al. Transcriptome analysis reveals abscisic acid enhancing drought

resistance by regulating genes related to flavonoid metabolism in pigeon pea. Environ Exp Bot 2021;191:104627. https://doi.org/10.1016/j.envexpbot.2021.104627.

5. Wen D, Zheng Y, Han Y, et al. Sodium selenite increases drought tolerance by promoting jasmonic acid biosynthesis in cucumber. HORTIC ADV 2023;1:6. https://doi.org/10.1007/s44281-023-00009-0.

6. Li T, Zhang Y, Liu Y, et al. Raffinose synthase enhances drought tolerance through raffinose synthesis or galactinol hydrolysis in maize and Arabidopsis plants. J Biol Chem 2020;295:8064–8077. https://doi.org/10.1074/jbc.RA120.013948.

7. Liu Y, Li T, Zhang C, et al. Raffinose positively regulates maize drought tolerance by reducing leaf transpiration. Plant J 2023;114:55–67. https://doi.org/10.1111/tpj.16116.

8. Hu H, Xiong L. Genetic engineering and breeding of drought-resistant crops. Annu Rev Plant Biol 2014;65:715–41. https://doi.org/10.1146/annurev-arplant-050213-040000.

9. Yang Z, Qin F. The battle of crops against drought: Genetic dissection and improvement. J Integr Plant Biol 2023;65:496–525. https://doi.org/10.1111/jipb.13451.

10. He Z, Zhang P, Jia H, et al. Regulatory mechanisms and breeding strategies for crop drought resistance. New Crops 2024;1:2949–9526. https://doi.org/10.1016/J.NCROPS.2024.100029.

11. Marques A, Moraes L, Dos Santos MA, et al. Origin and parental genome characterization of the allotetraploid *stylosanthes scabra* vogel (Papilionoideae, Leguminosae), an important legume pasture crop. Ann Bot 2018;122:1143–1159. https://doi.org/10.1093/aob/mcy113.

12. Cameron D. Chromosome number and morphology of some introduced *Strylosanthes* species. Crop & Pasture Science 1967;18:375–379.

13. Miller CP, Rains JP, Shaw KA, et al. Commercial development of *Stylosanthes pastures* in northern Australia. II. *Stylosanthes* in the northern Australian Beef Industry. Tropical Grasslands 1997; 31:509–514.

14. Luo J, Chen Z, Huang R, et al. Multi-omics analysis reveals the roles of purple acid phosphatases in organic phosphorus utilization by the tropical legume *Stylosanthes guianensis*. Plant J 2024 117:729–746. https://doi.org/10.1111/tpj.16526.

15. Song J, Zou X, Liu P, et al. Differential expressions and enzymatic properties of malate dehydrogenases in response to nutrient and metal stresses in *Stylosanthes guianensis*. Plant Physiol

Biochem 2022;170:325–337. https://doi.org/10.1016/j.plaphy.2021.12.012.
16. Siqueira JA, Wakin T, Batista-Silva W, et al. A long and stressful day: Photoperiod shapes aluminium tolerance in plants. J Hazard Mater 2022;432:128704. https://doi.org/10.1016/j.jhazmat.2022.128704.
17. Sun L, Liang C, Chen Z, et al. Superior aluminium (Al) tolerance of stylosanthes is achieved mainly by malate synthesis through an Al-enhanced malic enzyme, SgME1. New Phytol 2014;202:209–219. https://doi.org/10.1111/nph.12629.
18. Jia Y, Li X, Liu Q, et al. Physiological and transcriptomic analyses reveal the roles of secondary metabolism in the adaptive responses of *Stylosanthes* to manganese toxicity. BMC Genomics 2020;21:861. https://doi.org/10.1186/s12864-020-07279-2.
19. Liu P, Huang R, Hu X, et al. Physiological responses and proteomic changes reveal insights into *Stylosanthes* response to manganese toxicity. BMC Plant Biol 2019;19:212. https://doi.org/10.1186/s12870-019-1822-y.
20. Ribeiro RP, Costa LC, Medina EF, et al. Ethylene coordinates seed germination behavior in response to low soil pH in *Stylosanthes humilis*. Plant Soil 2018;425:87–100. https://doi.org/10.1007/s11104-018-3572-2.
21. Wu Y, Zhao C, Zhao X, et al. Multi-omics-based identification of purple acid phosphatases and metabolites involved in phosphorus recycling in stylo root exudates. Int J Biol Macromol 2023;241:124569. https://doi.org/10.1016/j.ijbiomac.2023.124569.
22. Braga GJ, Ramos AKB, Carvalho MA, et al. Liveweight gain of beef cattle in *Brachiaria brizantha* pastures and mixtures with *Stylosanthes guianensis* in the *Brazilian savannah*. Grass Forage Sci 2020;75:206–215. https://doi.org/10.1111/gfs.12473.
23. Schultze-Kraft R, Hubiao Y, Jun T, et al. Stylosanthes guianensis CIAT 184 – review of a tropical forage legume. Tropical Grasslands-Forrajes Tropicales 2023:11:95–120. https://doi.org/10.17138/tgft(11)95-120.
24. Huang Y, Wang H, Zhu Y, et al. THP9 enhances seed protein content and nitrogen-use efficiency in maize. Nature 2022;612:292–300. https://doi.org/10.1038/s41586-022-05441-2.
25. Qi X, Li MW, Xie M, et al. Identification of a novel salt tolerance gene in wild soybean by whole-genome sequencing. Nat Commun 2014;5:4340. https://doi.org/10.1038/ncomms5340

26. Zhang T, Peng W, Xiao H, et al. Population genomics highlights structural variations in local adaptation to saline coastal environments in woolly grape. J Integr Plant Biol 2024;66:1408–1426. https://doi.org/10.1111/jipb.13653.

27. Maass BL, Sawkins MC. History, relationships and diversity among *Stylosanthes* species of commercial significance. In: Chakraborty S (ed) High-yielding anthracnose-resistant *Stylosanthes* for agricultural systems. Australian Centre for International Agricultural Research, Canberra 2004:9–26.

28. Nurk S, Koren S, Rhie A, et al. The complete sequence of a human genome. Science 2022;376:44–53. https://doi.org/10.1126/science.abj6987.

29. He Y, Chu Y, Guo S, et al. T2T-YAO: A Telomere-to-telomere Assembled Diploid Reference Genome for Han Chinese. Genomics Proteomics Bioinformatics 2023;21:1085–1100. https://doi.org/10.1016/j.gpb.2023.08.001.

30. Ferreira-Neto JRC, da Silva MD, Binneck E, et al. Bridging the Gap: Combining Genomics and Transcriptomics Approaches to Understand *Stylosanthes scabra*, an Orphan Legume from the Brazilian Caatinga. Plants 2023;12:3246. https://doi.org/10.3390/plants12183246.

31. Zhang C, Xie L, Yu H, et al. The T2T genome assembly of soybean cultivar ZH13 and its epigenetic landscapes. Mol Plant 2023;16:1715–1718. https://doi.org/10.1016/j.molp.2023.10.003.

32. Wang L, Zhang M, Li M, et al. A telomere-to-telomere gap-free assembly of soybean genome. Mol Plant 2023;16:1711–1714. https://doi.org/10.1016/j.molp.2023.08.012.

33. Luo H, Wang X, You C, et al. Telomere-to-telomere genome of the allotetraploid legume *Sesbania cannabina* reveals transposon-driven subgenome divergence and mechanisms of alkaline stress tolerance. Sci China Life Sci 2024;67:149–160. https://doi.org/10.1007/s11427-023-2463-y.

34. Nei M. Gene duplication and nucleotide substitution in evolution. Nature 1969;221:40–2. https://doi.org/10.1038/221040a0.

35. Long M, Betrán E, Thornton K, et al. The origin of new genes: Glimpses from the young and old. Nat Rev Genet 2003;4:865–75. https://doi.org/10.1038/nrg1204.

36. Innan H, Kondrashov F. The evolution of gene duplications: Classifying and distinguishing between models. Nat Rev Genet 2010;11:97–108. https://doi.org/10.1038/nrg2689.

37. Liu C, Wu Y, Liu Y, et al. Genome-wide analysis of tandem duplicated genes and their

contribution to stress resistance in pigeonpea (*Cajanus cajan*). Genomics 2021;113:728–735. https:doi.org/10.1016/j.ygeno.2020.10.003.

38. Liu C, Tai Y, Luo J, et al. Integrated multi-omics analysis provides insights into genome evolution and phosphorus deficiency adaptation in pigeonpea (*Cajanus cajan*). Hortic Res 2022;9:uhac107. https://doi.org/10.1093/hr/uhac107.

39. Qu J, Liu L, Guo Z, et al. The ubiquitous position effect, synergistic effect of recent generated tandem duplicated genes in grapevine, and their co-response and overactivity to biotic stress. Fruit Research 2023;3:16. https://doi.org/10.48130/FruRes-2023-0016.

40. Salojärvi J, Smolander OP, Nieminen K, et al. Genome sequencing and population genomic analyses provide insights into the adaptive landscape of silver birch. Nat Genet 2017;49:904–912. https://doi.org/10.1038/ng.3862.

41. Hanada K, Zou C, Lehti-Shiu MD, et al. Importance of lineage-specific expansion of plant tandem duplicates in the adaptive response to environmental stimuli. Plant Physiol 2008;148:993–1003. https://doi.org/10.1104/pp.108.122457.

42. Liu JN, Fang H, Liang Q, et al. Genomic analyses provide insights into the evolution and salinity adaptation of halophyte *Tamarix chinensis*. Gigascience 2022;12:giad053. https://doi.org/10.1093/gigascience/giad053.

43. Wang M, Yuan J, Qin L, et al. TaCYP81D5, one member in a wheat cytochrome P450 gene cluster, confers salinity tolerance via reactive oxygen species scavenging. Plant Biotechnol J 2020;18:791–804. https://doi.org/10.1111/pbi.13247.

44. Wang X, Gao Y, Wu X, et al. High-quality evergreen azalea genome reveals tandem duplication-facilitated low-altitude adaptability and floral scent evolution. Plant Biotechnol J 2021;19:2544–2560. https://doi.org/10.1111/pbi.13680.

45. Shimada TL, Hayashi M, Hara-Nishimura I. Membrane Dynamics and Multiple Functions of Oil Bodies in Seeds and Leaves. Plant Physiol 2018;176:199-207. https://doi.org/10.1104/pp.17.01522.

46. Wan S, Xin XF. Regulation and integration of plant jasmonate signaling: a comparative view of monocot and dicot. J Genet Genomics 2022;49:704–714. https://doi.org/10.1016/j.jgg.2022.04.002.

47. Li X, Bai W, Yang Q, et al. The extremotolerant desert moss *Syntrichia caninervis* is a promising

pioneer plant for colonizing extraterrestrial environments. The Innovation 2024;5:100657. https://doi.org/10.1016/J.XINN.2024.100657.

48. Lyu S, Mei Q, Liu H, et al. Genome assembly of the pioneer species *Plantago major* L. (Plantaginaceae) provides insight into its global distribution and adaptation to metal-contaminated soil. DNA Research 2023;30:dsad013. https://doi.org/10.1093/dnares/dsad013.

49. Fuchs J, Brandes A, Schubert I. Telomere sequence localization and karyotype evolution in higher plants. Pl Syst Evol 1995;196:227–241. https://doi.org/10.1007/BF00982962.

50. Maravilla AJ, Rosato M, Rosselló JA. Interstitial Telomeric-like Repeats (ITR) in Seed Plants as Assessed by Molecular Cytogenetic Techniques: A Review. Plants (Basel) 2021;10:2541. https://doi.org/10.3390/plants10112541.

51. Xu Z, Pu X, Gao R, et al. Tandem gene duplications drive divergent evolution of caffeine and crocin biosynthetic pathways in plants. BMC Biol 2020;18:63. https://doi.org/10.1186/s12915-020-00795-3.

52. Cannon SB, Mitra A, Baumgarten A, et al. The roles of segmental and tandem gene duplication in the evolution of large gene families in *Arabidopsis thaliana*. BMC Plant Biol 2004;4:10. https://doi.org/10.1186/1471-2229-4-10.

53. Yin X, Yang D, Liu Y, et al. *Sophora moorcroftiana* genome analysis suggests association between sucrose metabolism and drought adaptation. Plant Physiol 2023;191:844–848. https://doi.org/10.1093/plphys/kiac558.

54. Yang Z, Nie G, Feng G, et al. Genome-wide identification of MADS-box gene family in orchardgrass and the positive role of DgMADS114 and DgMADS115 under different abiotic stress. Int J Biol Macromol 2022;223:129–142. https://doi.org/10.1016/j.ijbiomac.2022.11.027.

55. Yan H, Sun M, Zhang Z, et al. Pangenomic analysis identifies structural variation associated with heat tolerance in pearl millet. Nat Genet 2023;55:507–518. https://doi.org/10.1038/s41588-023-01302-4.

56. Herrera-Vásquez A, Salinas P, Holuigue L. Salicylic acid and reactive oxygen species interplay in the transcriptional control of defense genes expression. Front Plant Sci 2015;6:171. https://doi.org/10.3389/fpls.2015.00171.

57. Shu S, Gao P, Li L, et al. Abscisic acid-induced H_2O_2 accumulation enhances antioxidant

capacity in pumpkin-grafted cucumber leaves under $Ca(NO_3)_2$ stress. Front Plant Sci 2016;7:1489. https://doi.org/10.3389/fpls.2016.01489.

58. Kim TH, Böhmer M, Hu H, et al. Guard cell signal transduction network: Advances in understanding abscisic acid, CO_2, and Ca_2^+ signaling. Annu Rev Plant Biol 2010;61:561–91. https://doi.org/10.1146/annurev-arplant-042809-112226.

59. Song S, Qi T, Wasternack C, et al. Jasmonate signaling and crosstalk with gibberellin and ethylene. Curr Opin Plant Biol 2014;21:112–119. https://doi.org/10.1016/j.pbi.2014.07.005.

60. Gupta A, Bhardwaj M, Tran L-SP. JASMONATE ZIM-DOMAIN Family Proteins: Important Nodes in Jasmonic Acid-Abscisic Acid Crosstalk for Regulating Plant Response to Drought. Curr Protein Pept Sci 2021;22:759–766. https://doi.org/10.2174/1389203722666211018114443.

61. Mahmud S, Ullah C, Kortz A, et al. Constitutive expression of JASMONATE RESISTANT 1 induces molecular changes that prime the plants to better withstand drought. Plant Cell Environ 2022;45:2906–2922. https://doi.org/10.1111/pce.14402.

62. Staswick PE, Tiryaki I. The oxylipin signal jasmonic acid is activated by an enzyme that conjugates it to isoleucine in Arabidopsis. Plant Cell 2004;16:2117–27. https://doi.org/10.1105/tpc.104.023549.

63. Howe GA, Major IT, Koo AJ. Modularity in Jasmonate Signaling for Multistress Resistance. Annu Rev Plant Biol 2018;69:387–415. https://doi.org/10.1146/annurev-arplant-042817-040047.

64. Yan S, Liu Q, Li W, et al. Raffinose Family Oligosaccharides: Crucial Regulators of Plant Development and Stress Responses. Critical Reviews in Plant Sciences 2022;41:286–303. https://doi.org/10.1080/07352689.2022.2111756.

65. Lahuta LB, Górecki RJ, Szablińska-Piernik J, et al. Changes in the Carbohydrate Profile in Common Buckwheat (*Fagopyrum esculentum* Moench) Seedlings Induced by Cold Stress and Dehydration. Metabolites 2023;13:672. https://doi.org/10.3390/metabo13050672.

66. Meng D, Dong B, Niu L, et al. The pigeon pea CcCIPK14-CcCBL1 pair positively modulates drought tolerance by enhancing flavonoid biosynthesis. Plant J 2021;106:1278–1297. https://doi.org/10.1111/tpj.15234.

67. Hu J, Wang Z, Sun Z, et al. NextDenovo: an efficient error correction and accurate assembly tool for noisy long reads. Genome Biol 2024;25:107. https://doi.org/10.1186/s13059-024-03252-4.

68. Hu J, Fan J, Sun Z, et al. NextPolish: A fast and efficient genome polishing tool for long-read assembly. Bioinformatics 2020;36:2253–2255. https://doi.org/10.1093/bioinformatics/btz891.

69. Huang J, Liang X, Xuan Y, et al. LR_Gapcloser: a tiling path-based gap closer that uses long reads to complete genome assembly. Gigascience 2019;8:giy157. https://doi.org/10.1093/gigascience/giy157.

70. Ou S, Jiang N. LTR_retriever: A highly accurate and sensitive program for identification of long terminal repeat retrotransposons. Plant Physiol 2018;176:1410–1422. https://doi.org/10.1104/pp.17.01310.

71. Ou S, Su W, Liao Y, et al. Benchmarking transposable element annotation methods for creation of a streamlined, comprehensive pipeline. Genome Biol 2019;20:275. https://doi.org/10.1186/s13059-019-1905-y.

72. Pertea M, Kim D, Pertea GM, et al. Transcript-level expression analysis of RNA-seq experiments with HISAT, StringTie and Ballgown. Nat Protoc 2016;11:1650–67. https://doi.org/10.1038/nprot.2016.095.

73. Xu D, Yang J, Wen H, et al. CentIER: Accurate centromere identification for plant genomes. Plant Commun 2024:101046. https://doi.org/10.1016/j.xplc.2024.101046.

74. Nei M, Gojobori T. Simple methods for estimating the numbers of synonymous and nonsynonymous nucleotide substitutions. Mol Biol Evol 1986;3:418–26. https:doi/org/10.1093/oxfordjournals.molbev.a040410.

75. Bertioli DJ, Cannon SB, Froenicke L, et al. The genome sequences of *Arachis duranensis* and *Arachis ipaensis*, the diploid ancestors of cultivated peanut. Nat Genet 2016;48:438–46. https://doi.org/10.1038/ng.3517.

76. Boyle RK, McAinsh M, Dodd IC. Stomatal closure of Pelargonium × hortorum in response to soil water deficit is associated with decreased leaf water potential only under rapid soil drying. Physiol Plant 2016;156:84–96. https://doi.org/10.1111/ppl.12346.

77. Boyle RK, McAinsh M, Dodd IC. Daily irrigation attenuates xylem abscisic acid concentration and increases leaf water potential of Pelargonium × hortorum compared with infrequent irrigation. Physiol Plant 2016;158:23–33. https://doi.org/10.1111/ppl.12433.

78. Turner, N.C. Imposing and maintaining soil water deficits in drought studies in pots. Plant Soil

2019;439:45–55. https://doi.org/10.1007/s11104-018-3893-1.

79. Liang X, Liu S, Wang T, et al. Metabolomics-driven gene mining and genetic improvement of tolerance to salt-induced osmotic stress in maize. New Phytol 2021;230:2355–2370. https://doi.org/10.1111/nph.17323.

80. Lichtenthaler H K, Wellburn AR. Determinations of total carotenoids and chlorophylls a and b of leaf extracts in different solvents. Biochemical Society Transactions 1983;11:591–592.

81. Chen Y, Chen Y, Shi C, et al. SOAPnuke: A MapReduce acceleration-supported software for integrated quality control and preprocessing of high-throughput sequencing data. Gigascience 2018;7:1–6. https://doi.org/10.1093/gigascience/gix120.

82. Liu C, Huang R, Zhao X, et al. Comparative analysis of lipid and flavonoid biosynthesis between Pongamia and soybean seeds: genomic, transcriptional, and metabolic perspectives. Biotechnol Biofuels Bioprod 2024;17:86. https://doi.org/10.1186/s13068-024-02538-w.

83. Smith CA, Want EJ, O'Maille G, et al. XCMS: Processing mass spectrometry data for metabolite profiling using nonlinear peak alignment, matching, and identification. Anal Chem 2006;78:779–87. https://doi.org/10.1021/ac051437y.

84. Pang Z, Lu Y, Zhou G, et al. MetaboAnalyst 6.0: towards a unified platform for metabolomics data processing, analysis and interpretation. Nucleic Acids Res 2024;52:W398–W406. https://doi.org/10.1093/nar/gkae253.

Figure legends

Figure 1. Genomic features of *S. angustifolia*. (a) Features of assembled *S. angustifolia* genome. From 1 to 6: chromosomes, repeat element density, gene density, non-coding RNA density, GC content, and intraspecific collinearity between chromosomes. The contents of 2 to 5 were calculated using a non-overlapping window size of 500 Kb. **(b)** Hi-C interactions among ten chromosomes of the *S. angustifolia* genome. Dark red indicates strong interactions and yellow indicates weak interactions.

Figure 2. Comparative genomic analyses of *S. angustifolia* and other plant species. (a) Orthologous groups (OGs) and shared OGs of studied Fabaceae species and Arabidopsis. The red circle represents *S. angustifolia* specific OGs. **(b)** Phylogenetic trees and divergence time analysis

of the studied species based on single-copy OGs. **(c)** Genomic synteny comparisons between *S. angustifolia*, *A. duranensis*, and *A. ipaensis*. **(d)** Ks distribution of collinear gene pairs within and between *S. angustifolia*, *A. duranensis*, *A. ipaensis*, and *G. max*.

Figure 3. Transcriptome analysis and genome evolution of *S. angustifolia* in adaptation to drought stress. Plant phenotypes **(a)** and leaf characteristics **(b)** of *S. angustifolia* after 0 days (D0), 3 days (D3), and 5 days (D5) of drought treatment. Intersection analysis of genome-expanded genes and differentially expressed genes (DEGs) in the roots **(c)** and leaves **(d)** of *S. angustifolia* at D3 compared to D0. Intersection analysis of genome-expanded genes and DEGs in the roots **(e)** and leaves **(f)** of *S. angustifolia* at D5 compared to D0. **(g)** KEGG pathway enrichment analysis of genome-expanded genes that are up-regulated by drought stress in the roots and leaves of *S. angustifolia* (*Q*-value < 0.05).

Figure 4. Evolution and expansion of *xanthoxin dehydrogenase* (*ABA2*) genes in the *S. angustifolia* genome and their response to drought stress. (a) Phylogenetic tree of *ABA2* genes in *S. angustifolia*, *G. max*, *M. truncatula*, and *A. thaliana*. The heatmap illustrates the changes in the expression of the *ABA2* genes after drought treatment for 3 days (D3) or 5 days (D5), compared to the control (0 days, D0). Differentially expressed genes (DEGs) are defined as those with a $|\log_2$ fold change$| > 1$ and *Padj* < 0.05. **(b)** Microcollinearity of *ABA2* genes in *S. angustifolia* compared with *G. max* and *M. truncatula*. Red curves represent the correspondence of *ABA2* genes across different species. **(c)** The divergence time of *ABA2* genes occurred by tandem duplication. The quantification of ABA contents in the roots **(d)** and leaves **(e)** of *S. angustifolia*. Asterisks indicate significant differences between D5 and D0, as determined by Student's *t*-test: ****P* < 0.001.

Figure 5. Evolution and expansion of *2-hydroxyisoflavanone dehydratase* (*HIDH*) genes in the *S. angustifolia* genome and their response to drought stress. (a) Phylogenetic tree of *HIDH* genes in *S. angustifolia*, *G. max*, and *M. truncatula*. The heatmap illustrates the changes in the expression of the *HIDH* genes after drought treatment for 3 days (D3) or 5 days (D5), compared to the control (0 days, D0). Differentially expressed genes (DEGs) are defined as those with a $|\log_2$ fold change$| > 1$ and *Padj* < 0.05. **(b)** Microcollinearity of *HIDH* genes in *S. angustifolia* compared with *G. max* and *M. truncatula*. Red curves represent the correspondence of *HIDH* genes across different species. The quantification of genistein contents in the roots **(c)** and leaves **(d)** of *S. angustifolia*. The

quantification of daidzein contents in the roots **(e)** and leaves **(f)** of *S. angustifolia*. Asterisks indicate significant differences between D5 and D0, as determined by Student's *t*-test: * $0.01 \leq P < 0.05$, ** $P < 0.01$. NS, not significant.

Figure 6. Analysis of tandem duplicated genes (TDGs) and the biosynthesis pathway of raffinose and stachyose in *S. angustifolia* under drought stress. (a) Ks distribution of TDGs in *S. angustifolia*, *A. duranensis*, *A. ipaensis*, *G. max*, and *M. truncatula*. **(b)** KEGG pathway enrichment analysis of TDGs in *S. angustifolia*. The KEGG pathways depicted in the figure are associated with a *Q*-value < 0.05. **(c)** Gene expression changes in the raffinose and stachyose biosynthesis pathway after 3 days (D3) and 5 days (D5) of drought treatment compared to the control (0 days, D0). Differentially expressed genes (DEGs) are defined as those with a $|\log_2 \text{fold change}| > 1$ and $Padj < 0.05$. **(d)** Microcollinearity of *STS* genes in *S. angustifolia* compared with *G. max* and *M. truncatula*. Red curves represent the correspondence of *STS* genes across different species. The quantification of raffinose contents in the roots **(e)** and leaves **(f)** of *S. angustifolia*. The quantification of stachyose contents in the roots **(g)** and leaves **(h)** of *S. angustifolia*. Asterisks indicate significant differences between D5 and D0, as determined by Student's *t*-test: * $0.01 \leq P < 0.05$, ** $P < 0.01$.

Figure 7. Lipidomics analysis of roots and leaves of *S. angustifolia* in response to drought stress. The volcano plots show lipid profiles in the roots **(a)** and leaves **(b)** after 5 days (D5) of drought treatment compared to the control (0 days, D0). The heatmap illustrates the differentially accumulated triacylglycerols (TAGs) in the roots **(c)** and leaves **(d)** at D5 compared to D0. **(e)** Changes in gene expression of the *oleosin* and *caleosin* families at D3 and D5 compared to D0. Differentially expressed genes (DEGs) are defined as those with a $|\log_2 \text{fold change}| > 1$ and $Padj < 0.05$. **(f)** Phylogenetic tree of caleosins in *S. angustifolia, G. max, M. truncatula*, and *A. thaliana*.

Figure 8. Differential accumulation of phospholipids, sulfolipids, galactolipids, and glucolipids in the leaves (a) and roots (b) of *S. angustifolia* after 5 days (D5) of drought treatment compared to the control (0 days, D0).

Figure 9. Biosynthesis of jasmonic acid (JA) and jasmonoyl-L-isoleucine (JA-Ile) in *S. angustifolia* in response to drought stress. (**a**) Gene expression changes in the JA biosynthesis pathway after 3 days (D3) and 5 days (D5) of drought treatment compared to the control (0 days,

D0). Differentially expressed genes (DEGs) are defined as those with a |$\log_2$ fold change| > 1 and *Padj* < 0.05. (**b**) Microcollinearity of *pPLA* genes in *S. angustifolia* compared with *G. max* and *M. truncatula*. Red curves represent the correspondence of *pPLA* genes across different species. The quantification of JA contents in the roots **(c)** and leaves **(d)** of *S. angustifolia*. The quantification of JA-Ile contents in the roots **(e)** and leaves **(f)** of *S. angustifolia*. Asterisks indicate significant differences between D5 and D0, as determined by Student's *t*-test: ****P* < 0.001.

Additional Files

Fig. S1. The field phenotype of *S. angustifolia*.

Fig. S2. Genomic survey analysis of *S. angustifolia*.

Fig. S3. K-mer frequency distribution of 10 chromosomes.

Fig. S4. Gene family expansion and contraction analyses of seven studied plant species.

Fig. S5. KEGG classification of *S. angustifolia* expanded OGs.

Fig. S6. Leaf chlorophyll a (**a**), leaf chlorophyll b (**b**), soil water content (**c**), and shoot water content (**d**) of *S. angustifolia* after drought treatment for 0 days (D0), 3 days (D3), and 5 days (D5) under pot conditions.

Fig. S7. Volcano plot of differentially expressed genes (DEGs) in the roots and leaves of *S. angustifolia* under drought stress.

Fig. S8. (**a**) Intersection analysis of DEGs between roots and leaves at D3 compared to D0. (**b**) Intersection analysis of DEGs between roots and leaves at D5 compared to D0.

Table S1. Statistics of Oxford Nanopore (ONT) sequencing data of *S. angustifolia*.

Table S2. Statistics of Next-Generation Sequencing (NGS) data of *S. angustifolia*.

Table S3. Statistics of high-through chromosome conformation capture (Hi-C) sequencing data of *S. angustifolia*.

Table S4. Identification of telomere regions in the assembled *S. angustifolia* genome.

Table S5. BUSCO assessment of assembled genome.

Table S6. Statistics of repetitive sequence in the assembled genome.

Table S7. Classification of repetitive sequence in the assembled genome.

Table S8. Statistics of RNA-seq data from different tissues.

Table S9. BUSCO assessment of predicted gene set.

Table S10. Functional annotation of the predicted genes.

Table S11. Statistics of non-coding RNAs in the assembled genome.

Table S12. Statistics of RNA-seq data of *S. angustifolia* after drought treatment for 0 days (D0), 3 days (D3), and 5 days (D5).

Table S13. KEGG pathway enrichment analysis of the *S. angustifolia* expanded genes that were up-regulated in the roots at D3 compared to D0.

Table S14. KEGG pathway enrichment analysis of the *S. angustifolia* expanded genes that were up-regulated in the leaves at D3 compared to D0.

Table S15. KEGG pathway enrichment analysis of the *S. angustifolia* expanded genes that were up-regulated in the roots at D5 compared to D0.

Table S16. KEGG pathway enrichment analysis of the *S. angustifolia* expanded genes that were up-regulated in the leaves at D5 compared to D0.

Table S17. Identification of *ABA2* (K09841) genes in *S. angustifolia*, soybean, barrel medic, and Arabidopsis based on the KEGG database.

Table S18. Calculation of divergence time of the tandem duplicated *ABA2* genes in the *S. angustifolia* genome.

Table S19. Identification of *HIDH* (K13258) genes in *S. angustifolia*, soybean, and barrel medic based on the KEGG database.

Table S20. Calculation of divergence time of the tandem duplicated *HIDH* genes in the *S. angustifolia* genome.

Table S21. The expression changes of genes involved in the biosynthesis of raffinose and stachyose in *S. angustifolia* at D3 or D5 compared to D0.

Table S22. Lipid profiles in the roots of *S. angustifolia* at D5 and D0.

Table S23. Lipid profiles in the leaves of *S. angustifolia* at D5 and D0.

Table S24. Identification of oleosin and caleosin encoding genes in *S. angustifolia*, soybean, and barrel medic based on Arabidopsis database.

Table S25. The expression changes of genes involved in the biosynthesis of JA and JA-Ile in *S. angustifolia* at D3 or D5 compared to D0.

Figure 1

Click here to access/download;Figure;Figure 1.pdf

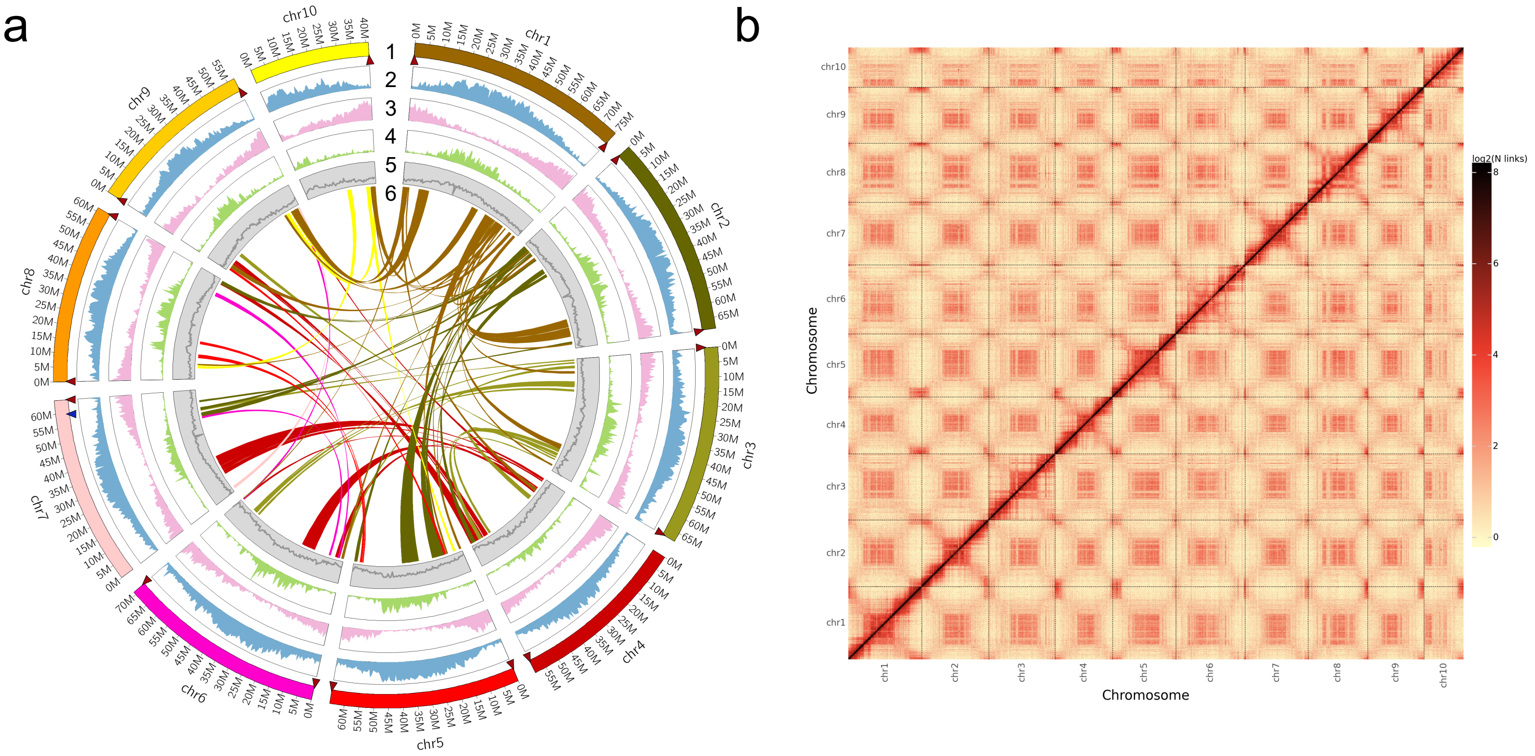

Figure 3

Click here to access/download;Figure;Figure 3.pdf

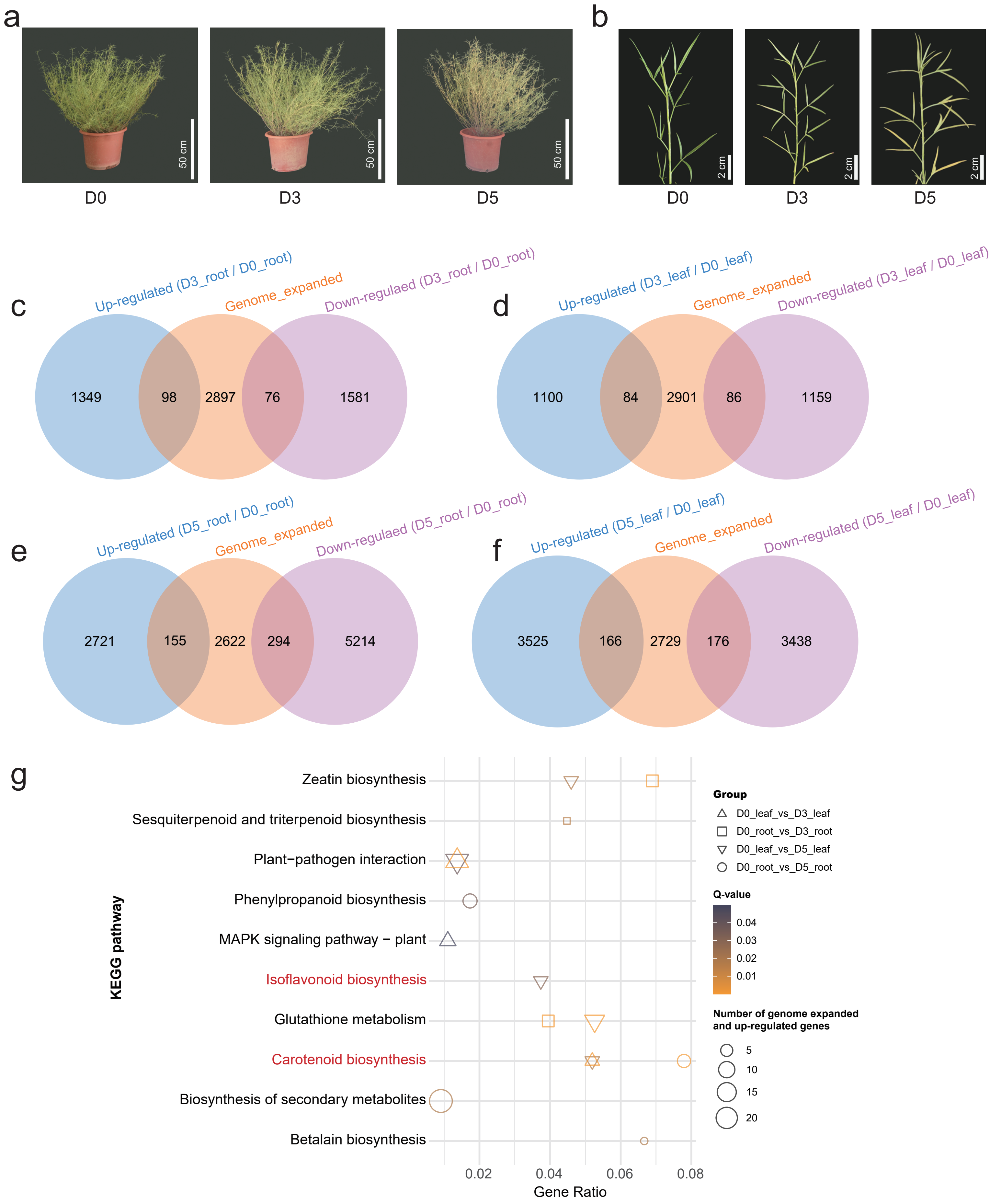

Figure 4 Click here to access/download;Figure;Figure 4.pdf

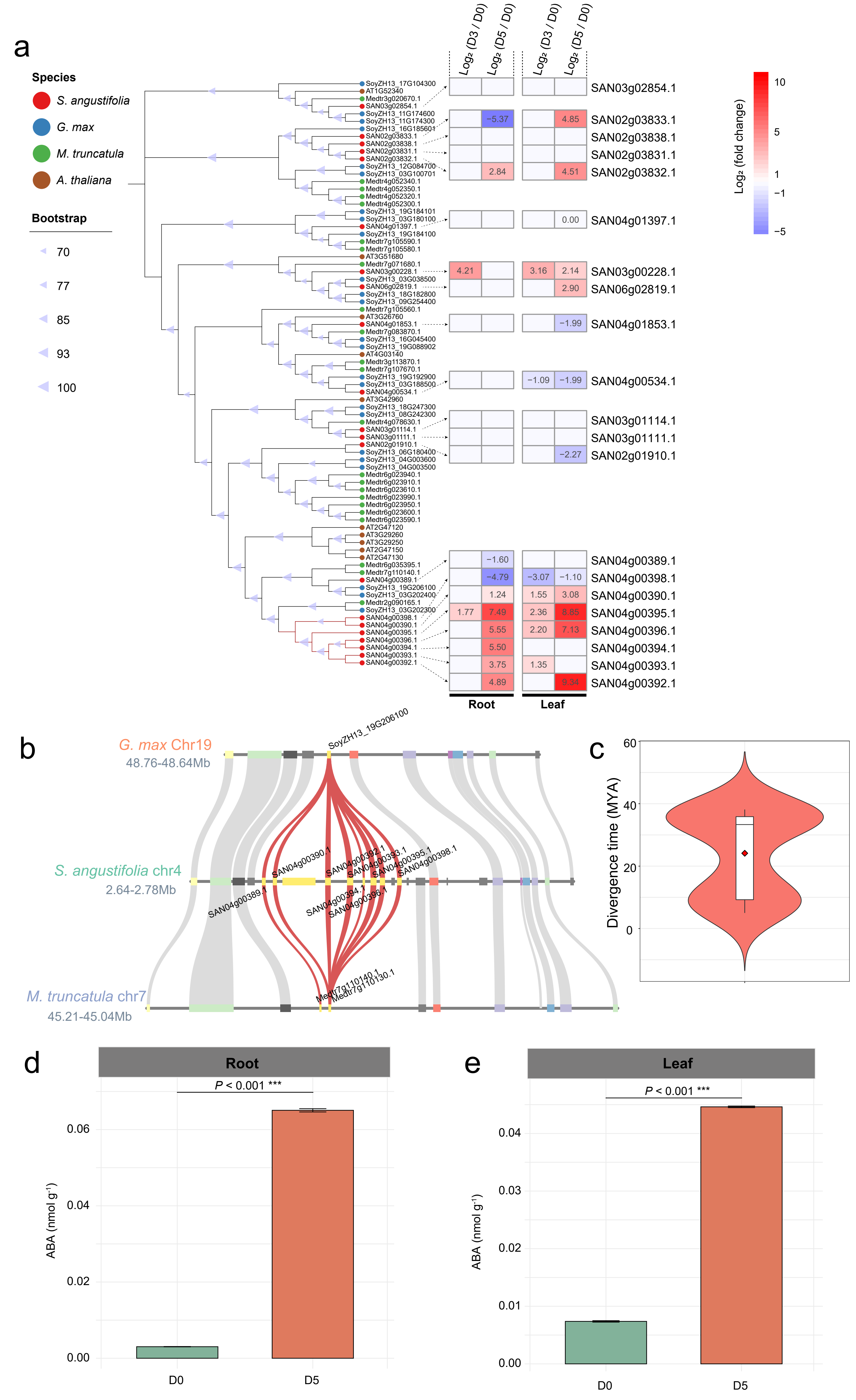

Figure 5

Click here to access/download;Figure;Figure 5.pdf

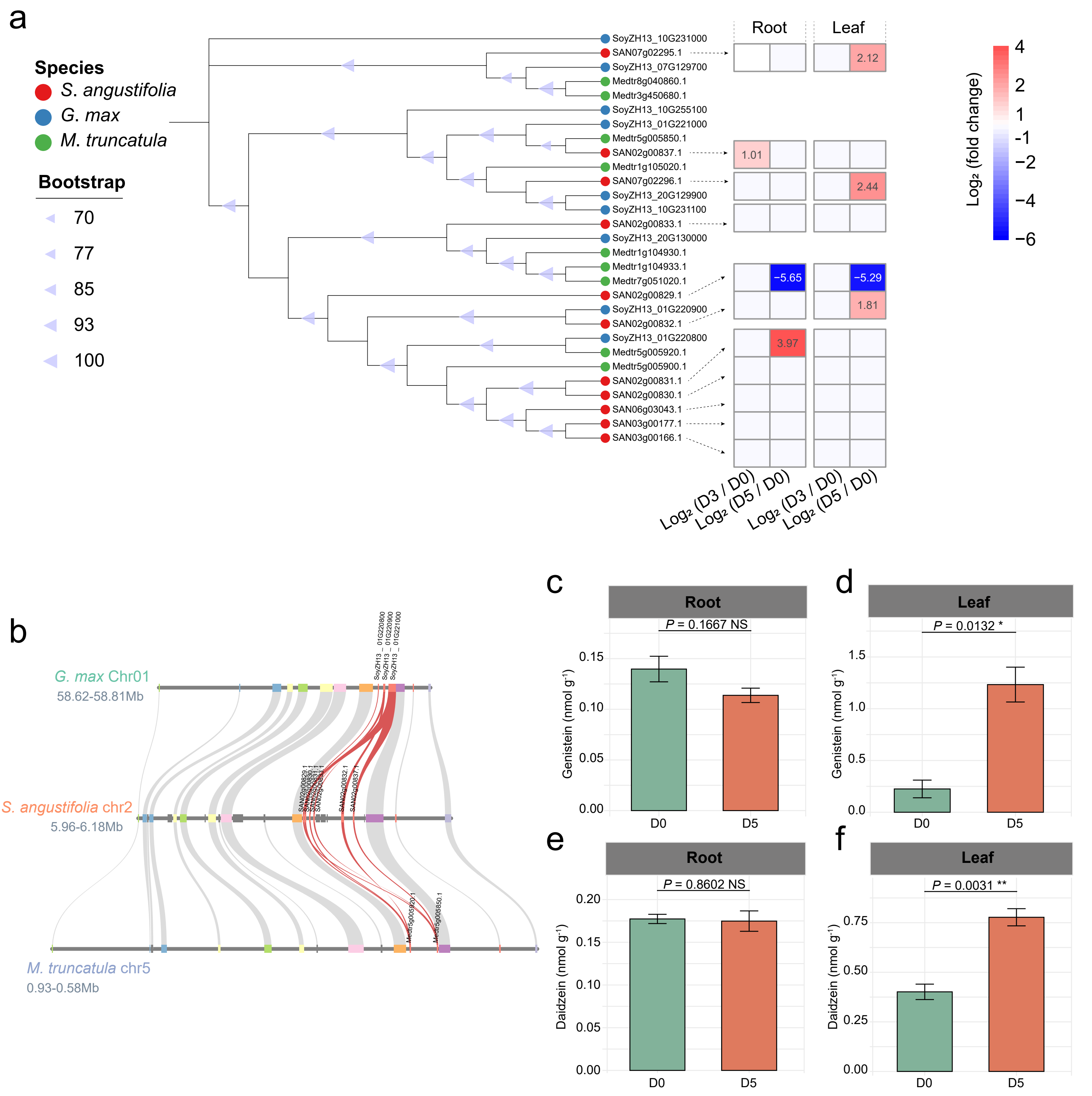

Figure 6

Click here to access/download;Figure;Figure 6.pdf

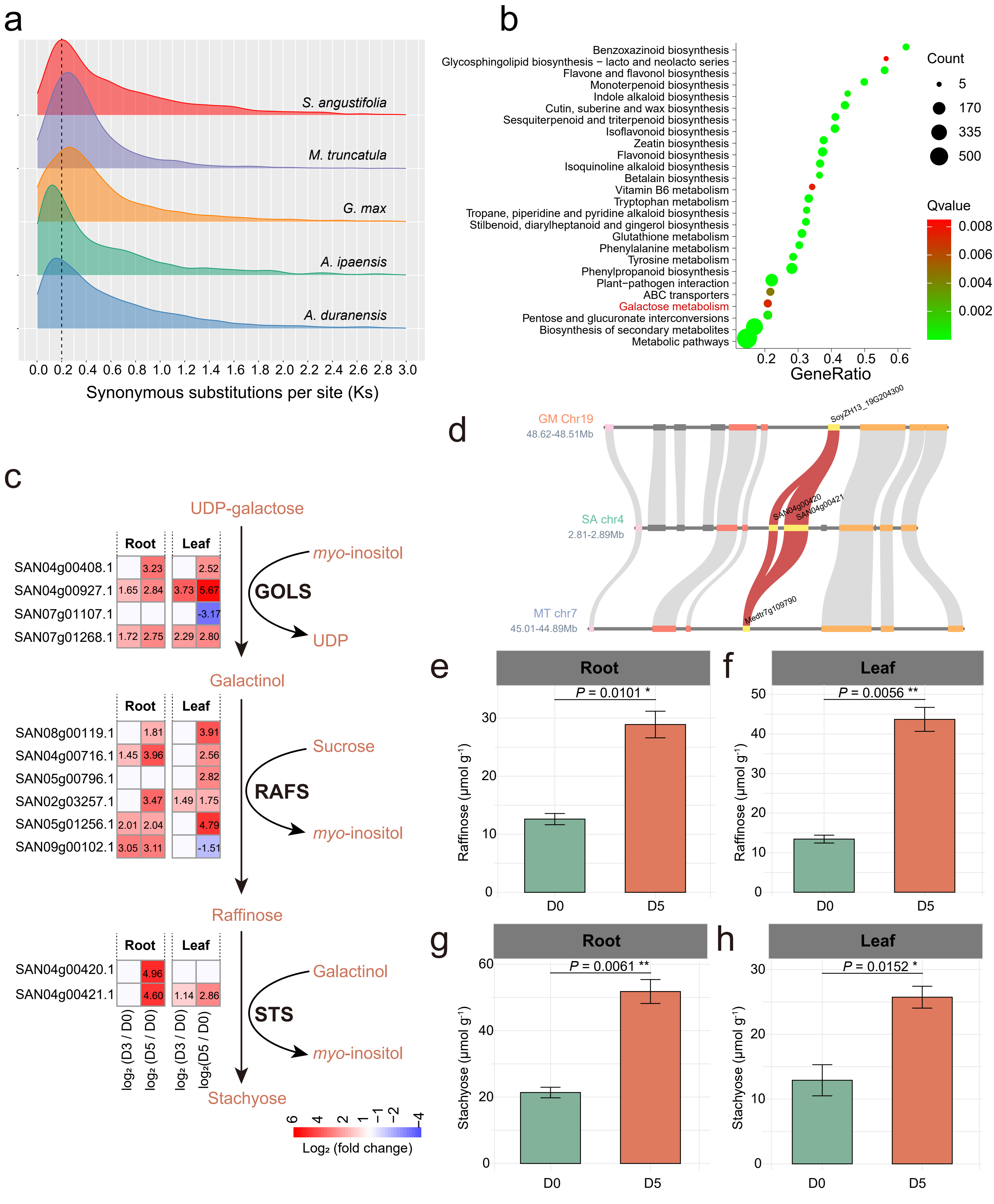

Figure 7

Click here to access/download;Figure;Figure 7.pdf

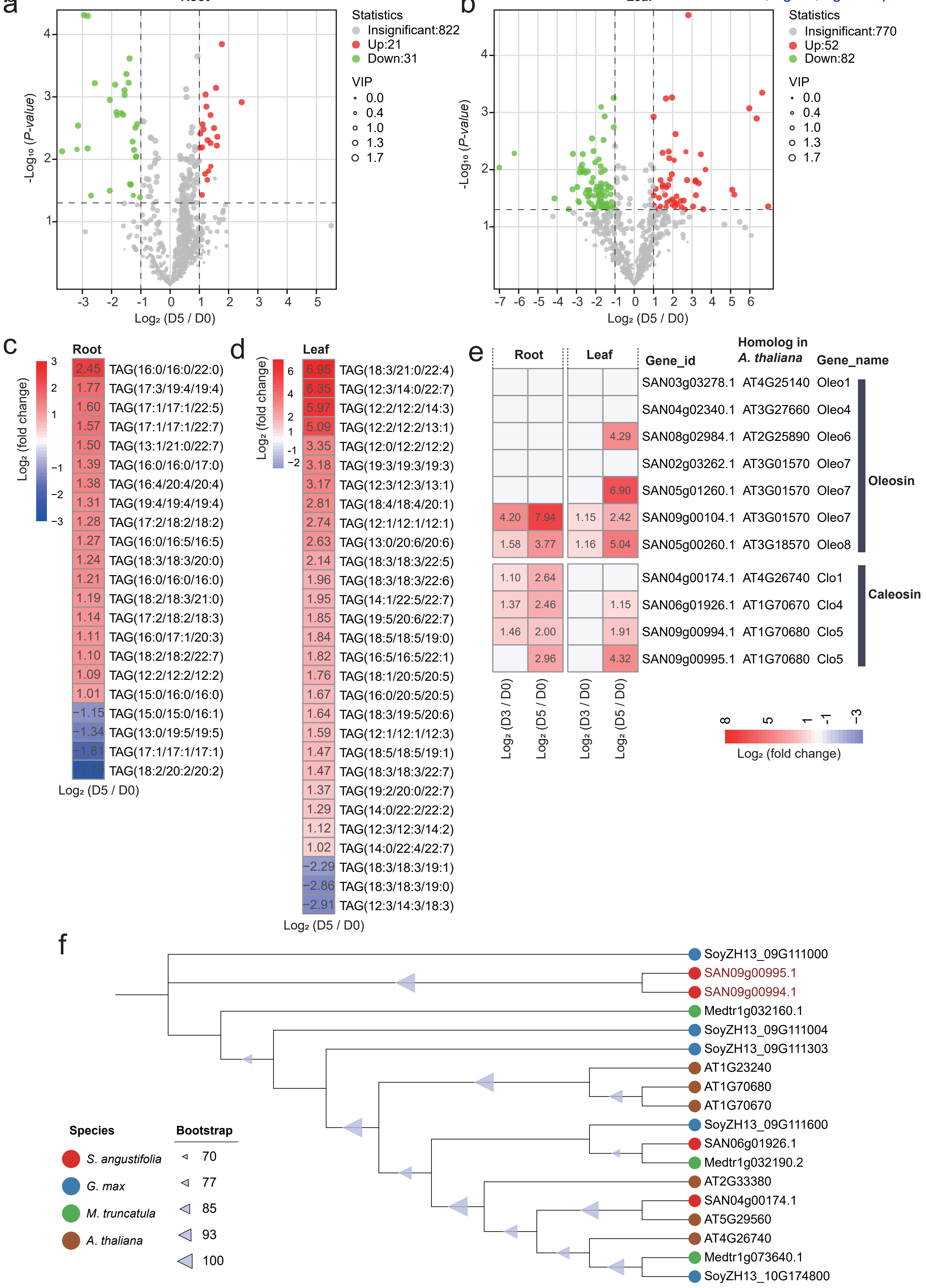

Figure 8

Click here to access/download;Figure;Figure 8.pdf

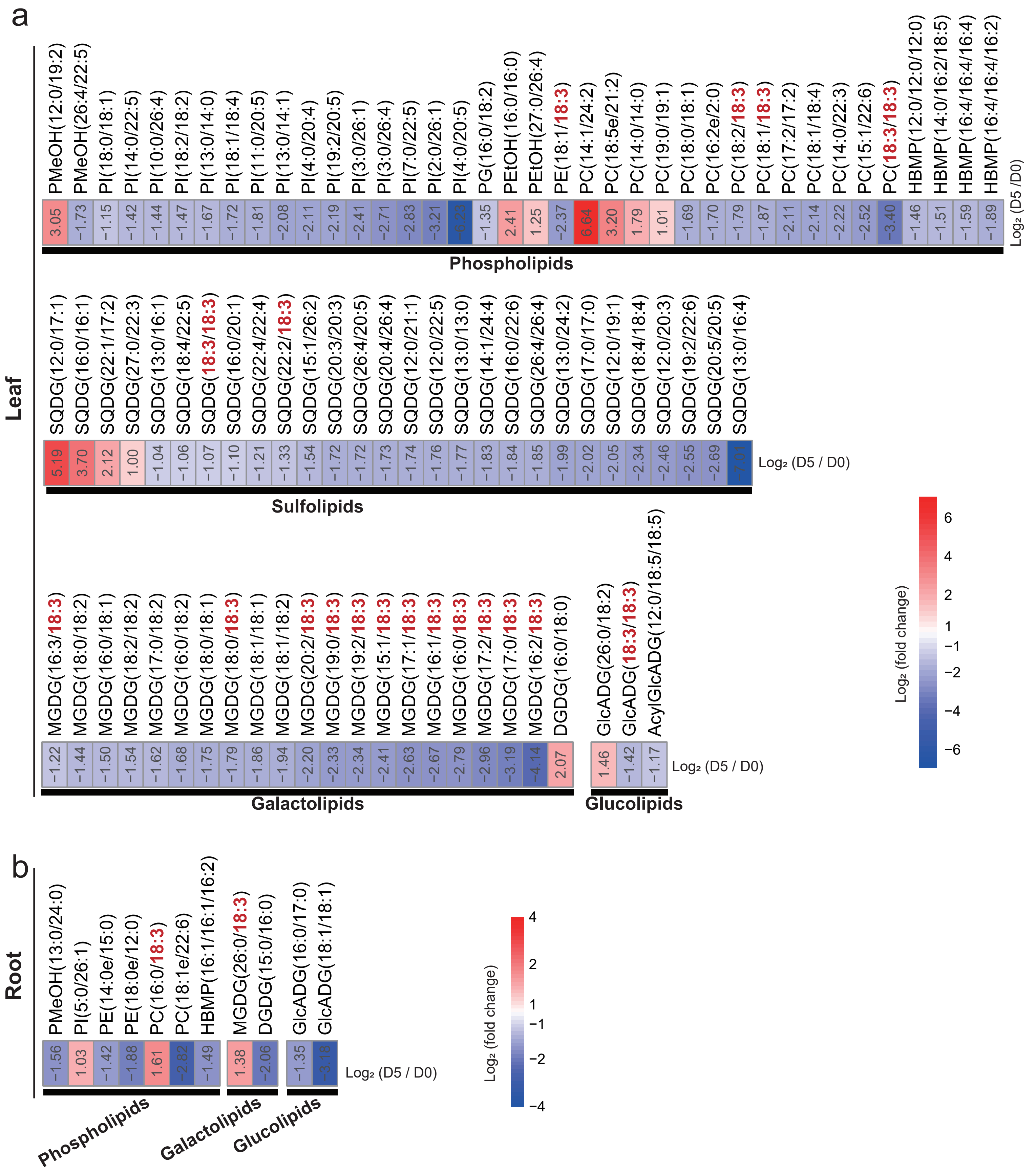

Figure 9

Click here to access/download;Figure;Figure 9.pdf

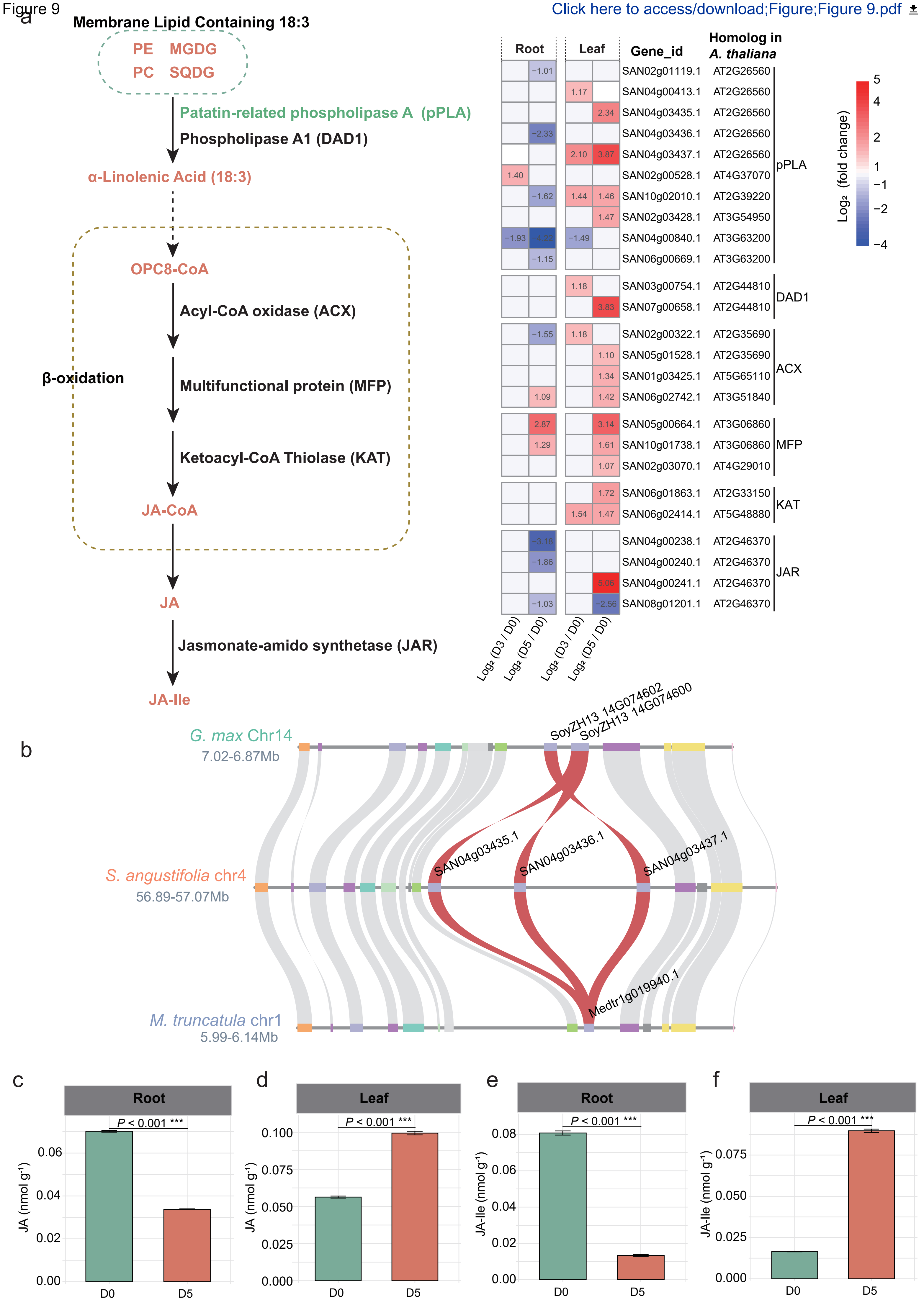

Figure S3

Click here to access/download;Figure;Figure S3.pdf

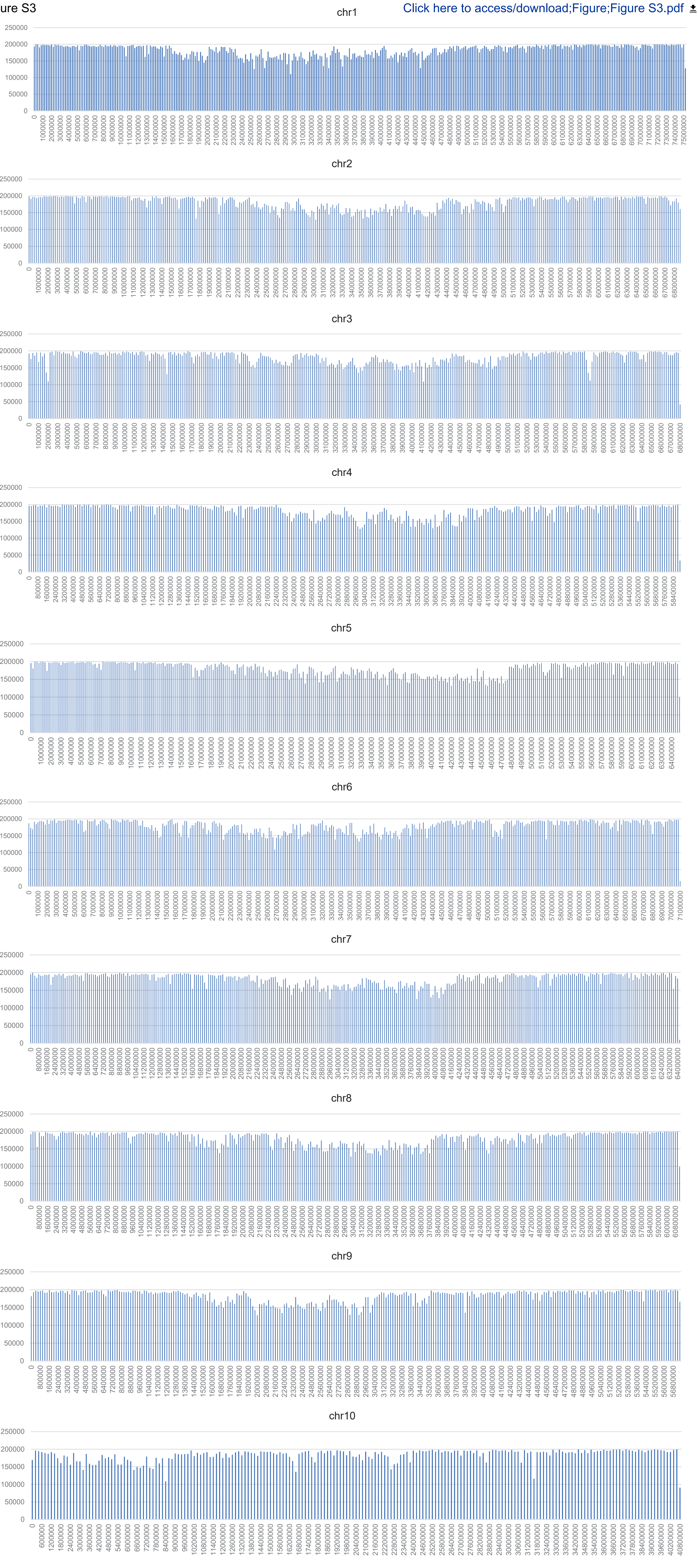

Figure S4

Click here to access/download;Figure;Figure S4.pdf

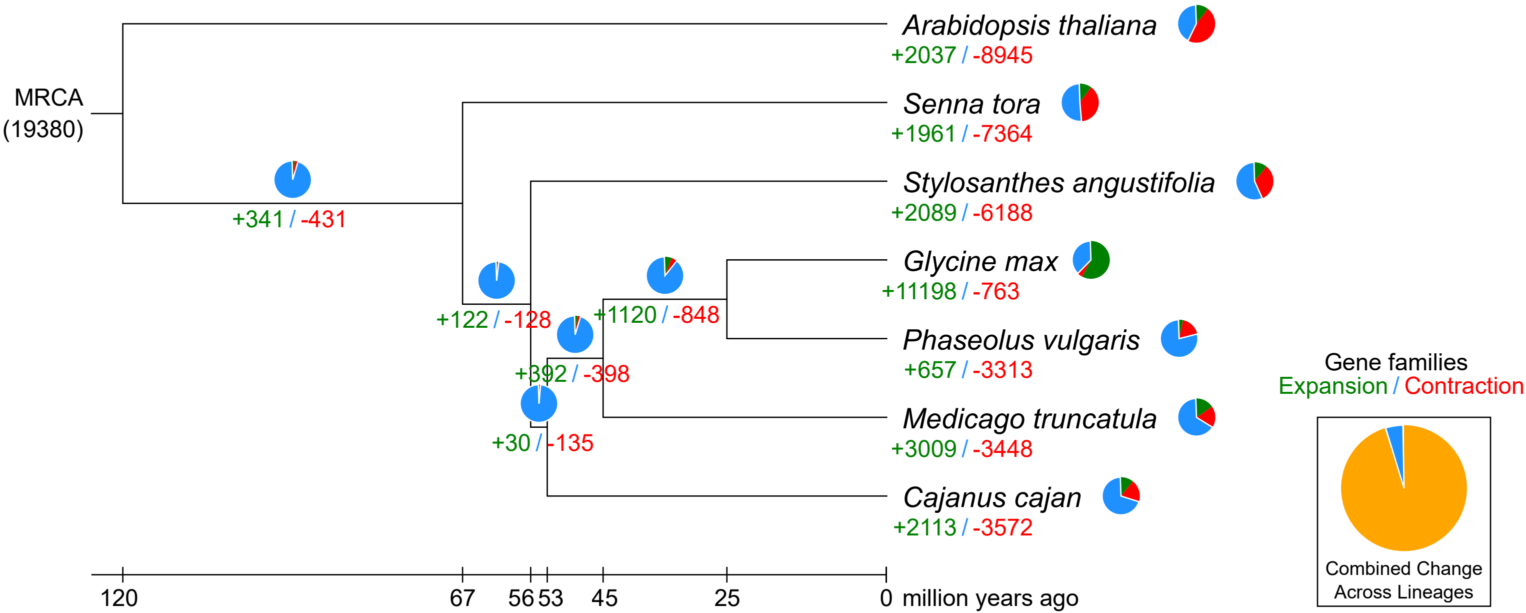

Figure S5

Click here to access/download;Figure;Figure S5.pdf

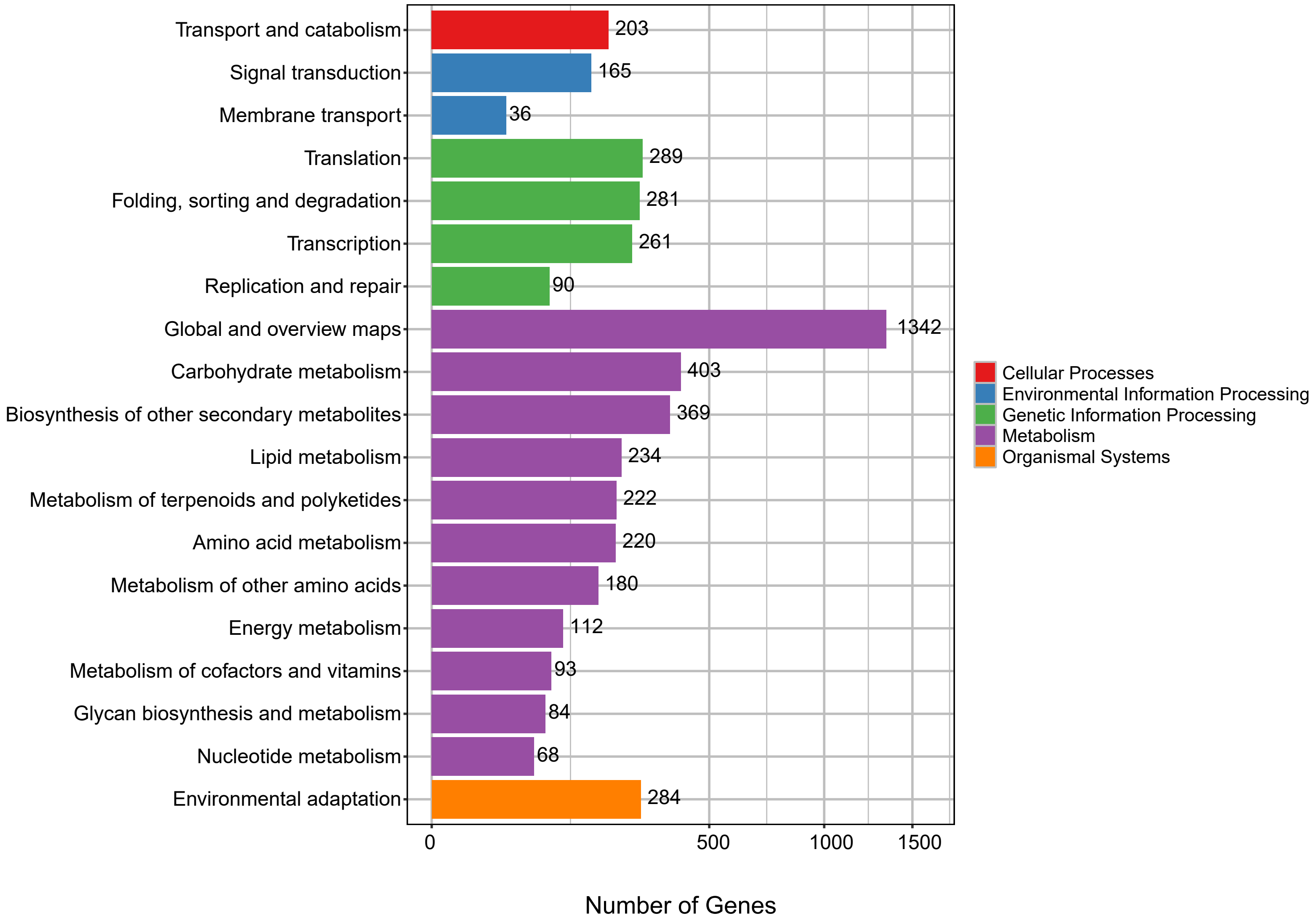

Figure S6

Click here to access/download;Figure;Figure S6.pdf

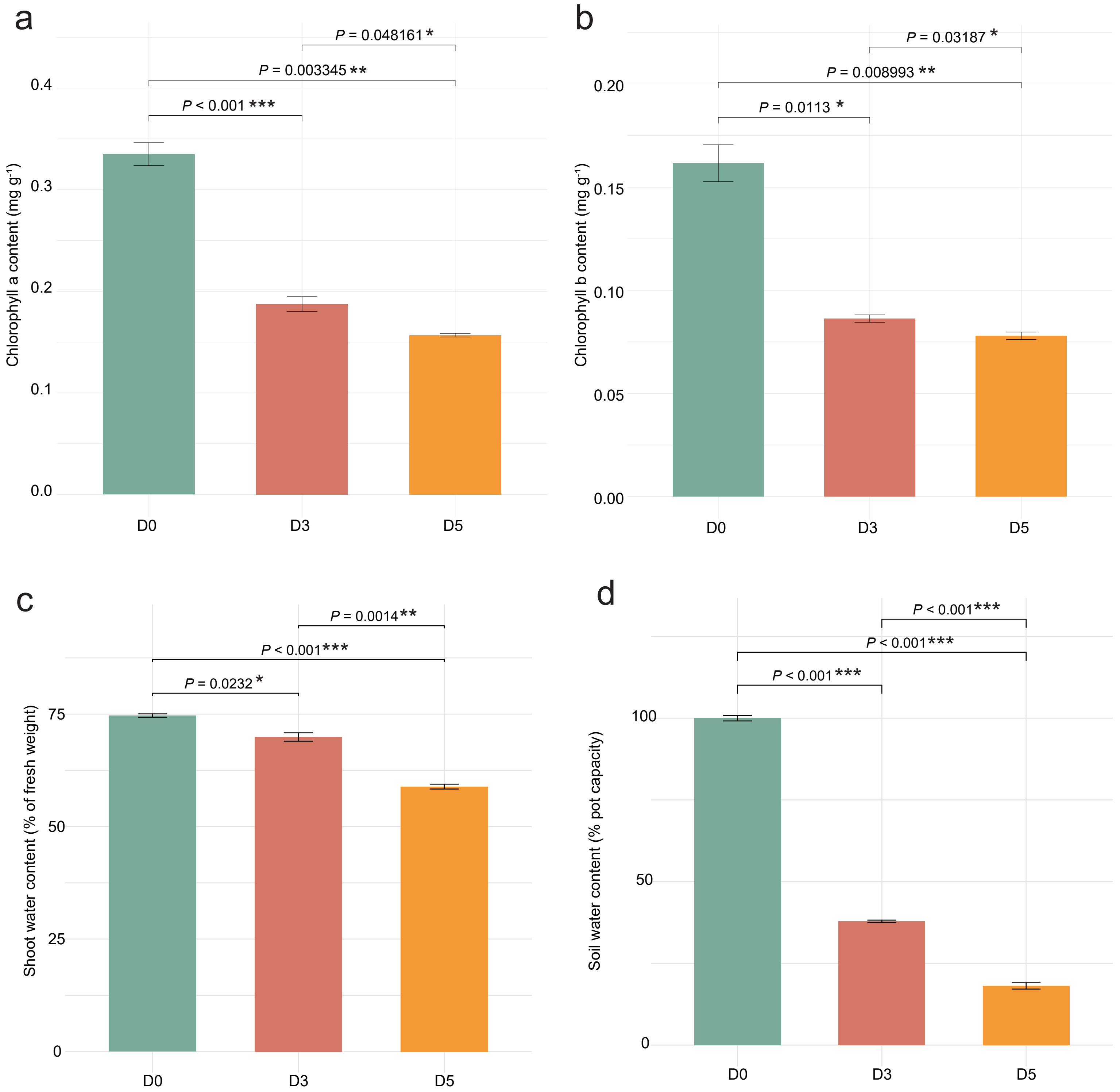

Figure S7

Click here to access/download;Figure;Figure S7.pdf

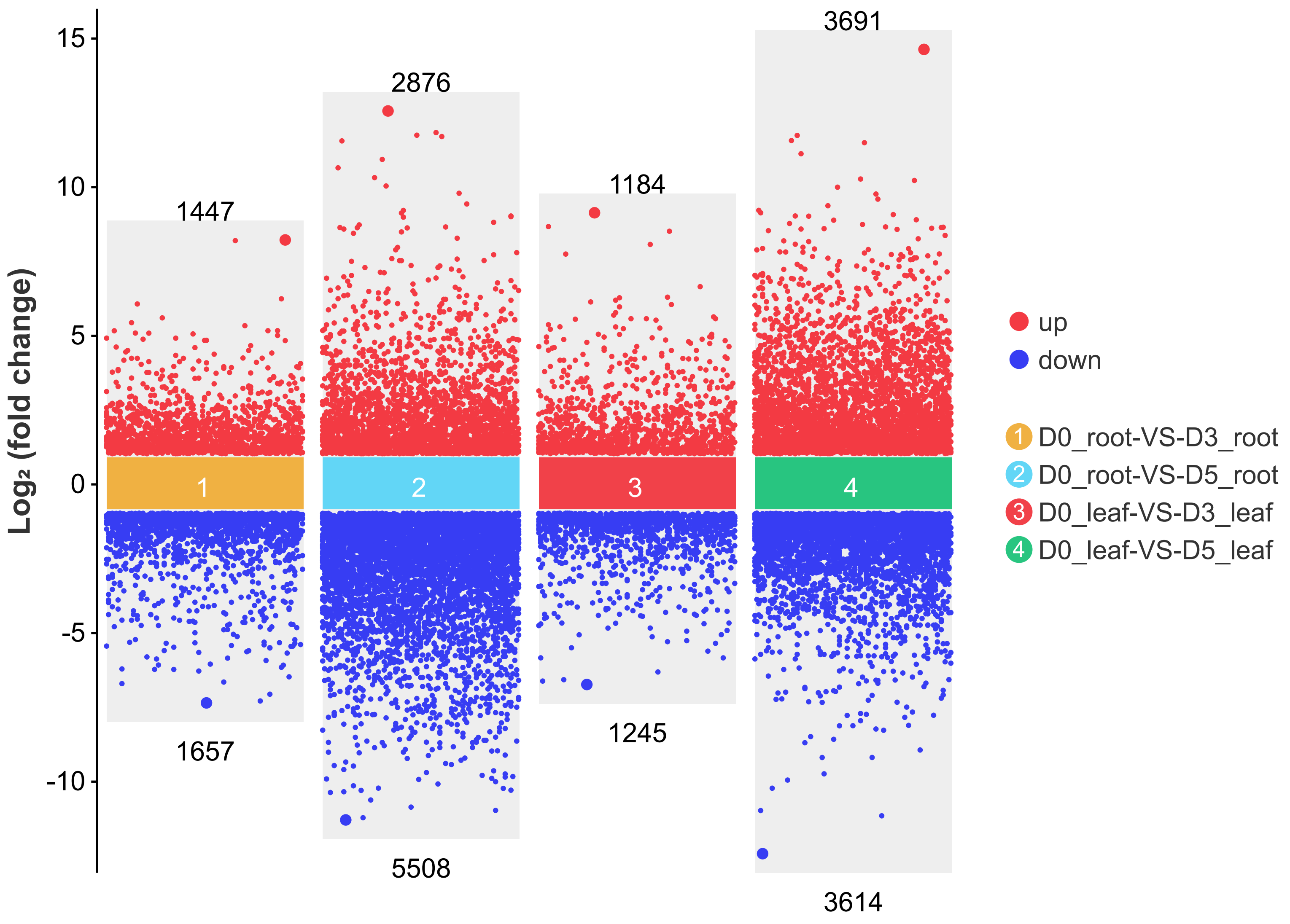

Figure S8

Click here to access/download;Figure;Figure S8.pdf

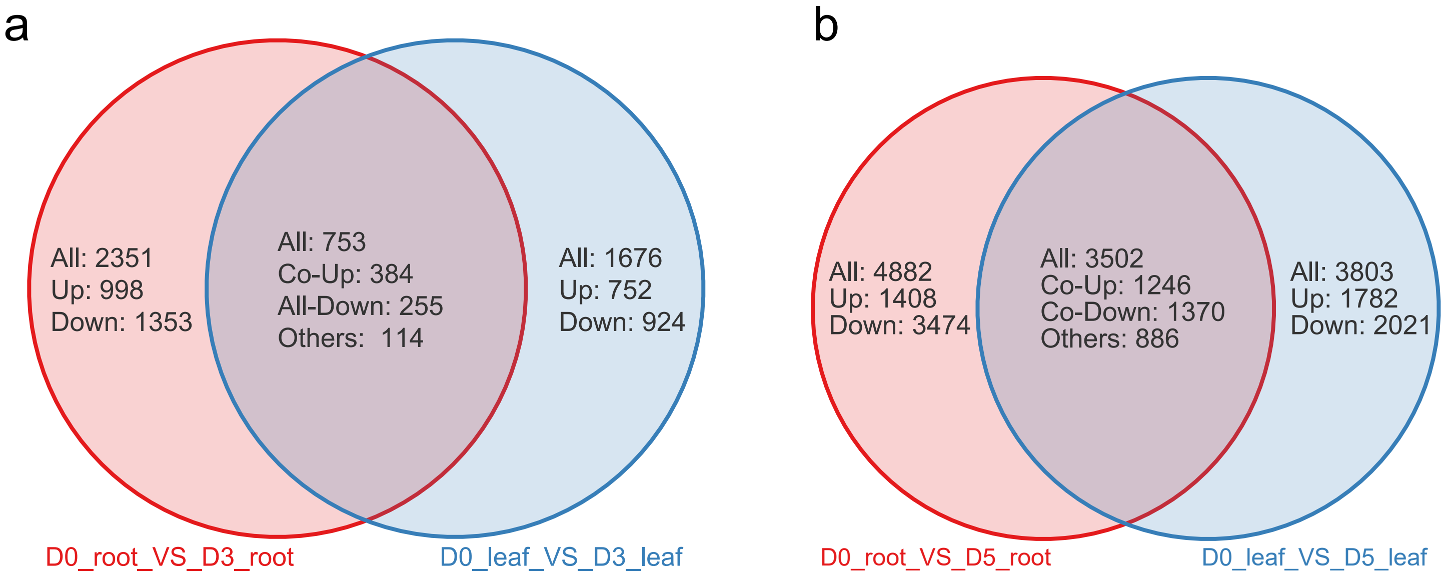

Supplementary Tables

Click here to access/download
Supplementary Material
Supplementary Tables.xlsx
