# Supplementary material for: A chromosome-scale genome assembly of the pioneer plant Stylosanthes angustifolia: insights into genome evolution and drought adaptation
Source: Gigascience. 2025 Jan 24;14:giae118. doi: 10.1093/gigascience/giae118 (PMC11758145; doi:10.1093/gigascience/giae118)
Supplement: giae118_GIGA-D-24-00294_Revision_2 [file giae118_giga-d-24-00294_revision_2.pdf]

GigaScience

A chromosome-scale genome assembly of the pioneer plant Stylosanthes angustifolia: insights into genome evolution and drought adaptation

--Manuscript Draft--

**Manuscript Number:**	GIGA-D-24-00294R2
**Full Title:**	A chromosome-scale genome assembly of the pioneer plant Stylosanthes angustifolia: insights into genome evolution and drought adaptation
**Article Type:**	Research
**Funding Information:**	Natural Science Foundation of Hainan Province (323CXTD387) — Prof. Pandao Liu<br>National Natural Science Foundation of China (32371769) — Prof. Pandao Liu<br>Earmarked fund for China Agriculture Research System—Forage and Grass (CARS-34) — prof. Guodao Liu<br>Central Public-interest Scientific Institution Basal Research Fund for CATAS (1630032022023 and 1630032024016) — Prof. Pandao Liu<br>Guangxi Special Project for Innovation-driven Development (Guike AA18242040) — Dr. Zhu Qiao<br>Earmarked fund for China Agriculture Research System—Green Manure (CARS-22) — prof. Guodao Liu
**Abstract:**	Background: Drought is a major limiting factor for plant survival and crop productivity. Stylosanthes angustifolia, a pioneer plant, exhibits remarkable drought tolerance, yet the molecular mechanisms driving its drought resistance remain largely unexplored.<br>Results: We present a chromosome-scale reference genome of S. angustifolia, which provides insights into its genome evolution and drought tolerance mechanisms. The assembled genome is 645.88 Mb in size, containing 319.98 Mb of repetitive sequences and 36,857 protein-coding genes. The high quality of this genome assembly is demonstrated by the presence of 99.26% Benchmarking Universal Single-Copy Orthologs and a 19.49 LTR assembly index. Evolutionary analyses revealed that S. angustifolia shares a whole-genome duplication (WGD) event with other legumes but lacks recent WGD. Additionally, S. angustifolia has undergone gene expansion through tandem duplication approximately 12.31 million years ago. Through integrative multi-omics analyses, we identified four gene families, namely xanthoxin dehydrogenase, 2-hydroxyisoflavanone dehydratase, patatin-related phospholipase A, and stachyose synthetase, that underwent tandem duplication and were significantly up-regulated under drought stress. These gene families contribute to the biosynthesis of abscisic acid, genistein, daidzein, jasmonic acid, and stachyose, thereby enhancing drought tolerance.<br>Conclusions: The genome assembly of S. angustifolia represents a significant advancement in understanding the genetic mechanisms underlying drought tolerance in this pioneer plant species. This genomic resource provides critical insights into the evolution of drought resistance and offers valuable genetic information for breeding programs aimed at improving drought resistance in crops.
**Corresponding Author:**	Pandao Liu<br>Chinese Academy of Tropical Agricultural Sciences Tropical Crops Genetic Resources Institute<br>Haikou, CHINA
**Corresponding Author Secondary Information:**	
**Corresponding Author's Institution:**	Chinese Academy of Tropical Agricultural Sciences Tropical Crops Genetic Resources Institute
**Corresponding Author's Secondary**	

Powered by Editorial Manager® and ProduXion Manager® from Aries Systems Corporation

Institution:	
First Author:	Chun Liu
First Author Secondary Information:	
Order of Authors:	Chun Liu
	Jianyu Zhang
	Ranran Xu
	Jinhui Lv
	Zhu Qiao
	Mingzhou Bai
	Shancen Zhao
	Lijuan Luo
	Guodao Liu
	Pandao Liu
Order of Authors Secondary Information:	
Response to Reviewers:	GIGA-D-24-00294R2<br><br>Dear Reviewers and Editors:<br><br>Thank you very much for your valuable feedback and comments. Your suggestions have been instrumental in revising and improving our manuscript. As recommended, we have carefully revised the manuscript and made corrections that we hope will meet your approval. The modified sections are highlighted in red within the manuscript. The main corrections in the paper and the responses to the reviewer's comments are as follows:<br><br>Reviewer reports:<br>Reviewer #1: The authors have addressed some of my concerns. I still have a few comments/questions/suggestions.<br>1.I acknowledge that the genome assembly present in this study can be high quality given the utilization of advanced sequencing technologies. However, I'm still not convinced that it qualifies as T2T or near-complete. Although the authours have provided some details regarding how the gaps were closed, they did not yet carefully check the gaps that were closed by LR_Gapcloser. My concern is that the authors did not generate new data, relying instead on the same ONT reads (N50 27kb, not ultra-long) used in the initial genome assembly to close these gaps. Almost 98.96% (95/96) gaps can be closed by this tool with the ONT reads. If these reads can bridge the gaps, it raises the question of why they were not incorporated into single contigs during the assembly process? Furthermore, telomeric sequences remain unassembled at one end of chromosomes 4, 7, and 10.<br>REPLY: Thank you for your valuable feedback on our manuscript. After careful consideration of your comments, we have decided to follow your suggestion and no longer describe the genome as a T2T assembly. We acknowledge your concerns regarding the use of LR_Gapcloser and the reliance on the same ONT reads (N50 27kb) for gap closure, which may not meet the rigorous standards required for a true T2T genome. Consequently, we have removed the gap-closing steps performed with LR_Gapcloser, retaining the 96 gaps in the genome, and reclassified the genome as a high-quality, chromosome-level assembly. Additionally, we have removed the sections related to telomeres and centromeres, which were previously associated with the T2T assembly. Furthermore, we have thoroughly reviewed the genomic data and corrected any inconsistencies. The manuscript, including the title, has also been revised to accurately reflect this updated description of the genome.<br><br>2.The authors have presented centromere identification as a standalone subsection in the Results. IMO, this result does not hold significant importance in this manuscript. This is partly because the centromeres are not studied in detail and also due to the

	instability of the identified centromeric regions between the two manuscript versions (ranging from 2.00 to 6.33 Mb in the revised version, compared to 141 kb to 25.6 Mb in the original manuscript).<br>REPLY: Thank you for your constructive feedback on our manuscript. We have carefully considered your comments regarding the centromere identification section. After revisiting the overall focus of the manuscript, we have decided to remove the centromere analysis from the revised version. As we no longer describe the genome as a T2T assembly, the detailed study of centromeres, which was not a primary focus of the manuscript, has been excluded.<br><br>3.Overall, the manuscript's language should be further improved for clarity and readability.<br>REPLY: Thank you for your suggestion to improve the clarity and readability of our manuscript. We fully understand the importance of precise language in effectively conveying our research findings. To address this, we have made comprehensive revisions throughout the manuscript and engaged a native English-speaking expert to carefully review and refine the language.<br>We have worked diligently to enhance the clarity and readability of the text, ensuring that the manuscript meets high standards of linguistic quality. We sincerely appreciate your feedback, as it has been invaluable in helping us improve the overall presentation of our work.
**Additional Information:**	
**Question**	**Response**
Are you submitting this manuscript to a special series or article collection?	No
**Experimental design and statistics**<br><br>Full details of the experimental design and statistical methods used should be given in the Methods section, as detailed in our Minimum Standards Reporting Checklist. Information essential to interpreting the data presented should be made available in the figure legends.<br><br>Have you included all the information requested in your manuscript?	Yes
**Resources**<br><br>A description of all resources used, including antibodies, cell lines, animals and software tools, with enough information to allow them to be uniquely identified, should be included in the Methods section. Authors are strongly encouraged to cite Research Resource Identifiers (RRIDs) for antibodies, model organisms and tools, where possible.	Yes

Powered by Editorial Manager® and ProduXion Manager® from Aries Systems Corporation

Have you included the information requested as detailed in our Minimum Standards Reporting Checklist?	
**Availability of data and materials**<br><br>All datasets and code on which the conclusions of the paper rely must be either included in your submission or deposited in publicly available repositories (where available and ethically appropriate), referencing such data using a unique identifier in the references and in the "Availability of Data and Materials" section of your manuscript.<br><br>Have you have met the above requirement as detailed in our Minimum Standards Reporting Checklist?	Yes

Powered by Editorial Manager® and ProduXion Manager® from Aries Systems Corporation

Manuscript
Click here to access/download;Manuscript;manuscript-SAN-revision_R2-revision-2.docx

A chromosome-scale genome assembly of the pioneer plant *Stylosanthes angustifolia*: insights into genome evolution and drought adaptation

Chun Liu[1,2,3,4], Jianyu Zhang[1,2,3,4], Ranran Xu[1,2,3,4], Jinhui Lv[1,2,3,4], Zhu Qiao[5], Mingzhou Bai[6], Shancen Zhao[7], Lijuan Luo[1], Guodao Liu[2,*], and Pandao Liu[2,3,4,*]

[1] School of Tropical Agriculture and Forestry & Sanya Institute Breeding and Multiplication, Hainan University, Haikou/Sanya 570228/572025, China.

[2] Tropical Crops Genetic Resources Institute, Chinese Academy of Tropical Agricultural Sciences (CATAS), Haikou 571101, China

[3] Key Laboratory of Crop Gene Resources and Germplasm Enhancement in Southern China, Ministry of Agriculture and Rual Affairs, Haikou 571101, China.

[4] Key Laboratory of Tropical Crops Germplasm Resources Genetic Improvement and Innovation of Hainan Province, Haikou 571101, China.

[5] Guangxi Key Laboratory of Medicinal Resources Protection and Genetic Improvement/ Guangxi Engineering Research Center of TCM Resource Intelligent Creation, Guangxi Botanical Garden of Medicinal Plants, Nanning 530023, China.

[6] Department of Biotechnology and Biomedicine, Technical University of Denmark, Kongens Lyngby 2800, Denmark.

[7] Beijing Life Science Academy, Beijing 102200, China.

E-mail addresses of all authors

Chun Liu: xiaoyaoma@live.cn

Jianyu Zhang: jianyuzhang@hainanu.edu.cn

Ranran Xu: 2512891913@qq.com

Jinhui Lv: lvjinhuixue@163.com

Zhu Qiao: qiaozhu@gxyyzwy.com

Mingzhou Bai: mingbai@dtu.dk

Shancen Zhao: zhaosc@blsa.com.cn

Lijuan Luo: luoljd@126.com
Guodao Liu: Guodao_Liu@163.com
Pandao Liu: liupandao2019@163.com

*Correspondence addresses.
Pandao Liu, CATAS, West Xueyuan Road, Haikou 571101, China. E-mail: liupandao2019@163.com, ORCID: 0000-0003-2296-9130;
Guodao Liu, CATAS, West Xueyuan Road, Haikou 571101, China. E-mail: Guodao_Liu@163.com, ORCID: 0000-0003-4189-9959.

ORCID iDs:
Chun Liu [0000-0003-1404-340X]; Jianyu Zhang [0000-0002-6991-3938]; Ranran Xu; Jinhui Lv; Zhu Qiao [0000-0002-8044-1230]; Mingzhou Bai; Shancen Zhao; Lijuan Luo; Guodao Liu [0000-0003-4189-9959]; Pandao Liu [0000-0003-2296-9130];

Abstract

Background: Drought is a major limiting factor for plant survival and crop productivity. *Stylosanthes angustifolia*, a pioneer plant, exhibits remarkable drought tolerance, yet the molecular mechanisms driving its drought resistance remain largely unexplored.

Results: We present a chromosome-scale reference genome of *S. angustifolia*, which provides insights into its genome evolution and drought tolerance mechanisms. The assembled genome is 645.88 Mb in size, containing 319.98 Mb of repetitive sequences and 36,857 protein-coding genes. The high quality of this genome assembly is demonstrated by the presence of 99.26% Benchmarking Universal Single-Copy Orthologs and a 19.49 LTR assembly index. Evolutionary analyses revealed that *S. angustifolia* shares a whole-genome duplication (WGD) event with other legumes but lacks recent WGD. Additionally, *S. angustifolia* has undergone gene expansion through tandem duplication approximately 12.31 million years ago. Through integrative multi-omics analyses, we identified four gene families, namely *xanthoxin dehydrogenase*, *2-hydroxyisoflavanone dehydratase*,

patatin-related phospholipase A, and *stachyose synthetase,* that underwent tandem duplication and were significantly up-regulated under drought stress. These gene families contribute to the biosynthesis of abscisic acid, genistein, daidzein, jasmonic acid, and stachyose, thereby enhancing drought tolerance.

Conclusions: The genome assembly of *S. angustifolia* represents a significant advancement in understanding the genetic mechanisms underlying drought tolerance in this pioneer plant species. This genomic resource provides critical insights into the evolution of drought resistance and offers valuable genetic information for breeding programs aimed at improving drought resistance in crops.

Keywords: *Stylosanthes angustifolia*, Pioneer plant, *De novo* assembly, Multi-omics, Drought tolerance

Introduction

Drought represents one of the most significant environmental challenges, drastically impairing plant survival and considerably reducing annual crop yields [1,2]. Plants have developed a range of physiological, biochemical, and morphological mechanisms to respond to drought stress. These include stomatal closure to minimize transpiration, alterations in root architecture to optimize water uptake, and increased biosynthesis of compounds such as abscisic acid (ABA), osmoprotectants, flavonoids, and isoflavonoids [1,3–6]. Additionally, the accumulation of non-reducing sugars, such as raffinose, plays a crucial role in drought adaptation [7,8]. Understanding the genes and molecular pathways that underpin these adaptive responses is essential for advancing the development of drought-tolerant crop cultivars [9–11]. Therefore, comprehensive research on the genetic mechanisms that enable plants to withstand drought stress is urgently needed.

The genus *Stylosanthes* (family Leguminosae) comprises approximately 50 species. These include diploids (2n = 2x = 20), tetraploids (2n = 4x = 40), and hexaploids (2n = 6x = 60) species, distributed across tropical and subtropical regions [12,13]. This genus is a pioneer plant in acid soils [14], exhibiting superior adaptability to frequent abiotic stresses such as low phosphorus availability [15,16], aluminum toxicity [17,18], manganese toxicity [19,20], and low pH [21]. Among the species of the *Stylosanthes* genus, *S. guianensis* (2n = 2x = 20) stands out as the most widely

domesticated and utilized species, serving as both forage and green manure [22]. Numerous cultivars of *S. guianensis* have been developed across different countries: the cultivars "Bandeirante", "Mineirão", and "IRI 1022" in Brazil; the cultivars "Schofield", "Endeavour", and "Cook" in Australia; and the cultivars "Reyan No. 2", "Reyan No. 5", and "Stylo 907" in China [14,23,24]. The significance of utilizing wild relatives in genomic and genetic research is immense, as they offer invaluable genetic diversity and traits that are crucial for enhancing cultivars [25–27]. *S. angustifolia*, a diploid species (2n = 2x = 20), is a wild relative of *S. guianensis* [28]. We observed that *S. angustifolia* exhibits good adaptability in the dry-hot valley regions of southwestern China, which frequently experience seasonal drought. However, the potential mechanisms underlying its drought tolerance remain unclear. This study aims to elucidate the molecular basis of drought-tolerant traits in *S. angustifolia* through genomic sequencing, thereby providing genetic resources for breeding drought-adapted *Stylosanthes* cultivars.

Gene duplication, including tandem duplication, plays a pivotal role in generating genetic diversity and driving plant evolution and adaptation [29–31]. Recent researches have highlighted the role of tandem duplicated genes (TDGs) in environmental adaptation, as demonstrated in species like pigeonpea (*Cajanus cajan*) [32,33]. grapevine (*Vitis vinifera*) [34], and silver birch (*Betula pendula*) [35]. Lineage-specific TDGs are particularly important for the adaptive evolution of plants in rapidly changing environments [36]. Whole-genome duplication (WGD) and tandem duplication have also been linked to the expansion of salinity adaptation genes in the halophyte *Tamarix chinensis* [37]. Genome-wide identification of TDGs, combined with multi-omics analyses, is essential for understanding how these genes contribute to plant evolution and environmental adaptation [33,37–39].

In this study, we present a high-quality, *de novo* assembly of the leguminous pioneer plant *S. angustifolia* (NCBI:txid79067), utilizing ONT, NGS, and high-through chromosome conformation capture (Hi-C) technologies. Comparative genomics reveals the evolutionary position and divergence of *S. angustifolia*. Integrating comparative genomics, transcriptomics, and metabolomics, we demonstrate the critical role of TDGs in *S. angustifolia* genome evolution and its adaptation to drought stress.

Results

Sequencing and Assembly of the *S. angustifolia* Genome

In this study, we employed NGS and ONT technologies for the whole-genome sequencing of *S. angustifolia* (Germplasm number: TF0003 and Figure S1). We generated a total of 35.08 Gb of NGS data (~ 53.02 × coverage) and 104.72 Gb of ONT data (~ 158.29 × coverage) (Table S1 and S2). Based on k-mer analysis of the NGS data, we estimated the genome size of *S. angustifolia* to be 661.55 Mb (Figure S2). We used the software NextDenovo to correct and assemble the raw ONT data, resulting in an initial assembly of 167 contigs with a total length of 645.87 Mb and an N50 of 14.98 Mb. Subsequently, we employed NextPolish to polish the initial contigs using both ONT and NGS data to produce high-quality contigs. To further achieve chromosomal-level assembly, we used Hi-C technology, generating 53.89 Gb of clean data (~ 81.47 × coverage) (Table S3). By analyzing the Hi-C data with Juicer and 3D de novo assembly (3D-DNA) software, we were able to align and organize the high-quality contigs into chromosomes. This achieving a chromosomal-level assembly of the *S. angustifolia* genome, spanning 631.54 Mb (97.78% of the assembled genome) across 10 chromosomes and containing 96 gaps. The final assembled genome had a total length of 645.88 Mb, a GC content of 35.52 %, and a contig N50 of 14.99 Mb (Figure 1 and Table 1). Single nucleotide polymorphism (SNP) analysis using genome analysis toolkit (GATK) identified 379,931 heterozygous SNPs, resulting in a heterozygosity rate of 0.06%. We evaluated the completeness of the assembled genome using the Benchmarking Universal Single-Copy Orthologs (BUSCO), which yielded a completeness score of 99.26%, and the Long Terminal Repeat (LTR) Assembly Index (LAI), which provided a score of 19.49 (Table 1 and Table S4). Additionally, k-mer completeness analysis showed a score of 96.37% for the *S. angustifolia* genome (Table 1). The mapping rates of the NGS and ONT reads to the assembled genome were 99.15% and 99.69%, respectively, with average depths of 54.16× for NGS reads and 156.20× for ONT reads. In total, 95.16% of the genome was covered by NGS and 99.96% by ONT reads.

Genome Annotation of the *S. angustifolia* Genome

We applied both *de novo* and homology-based approaches to annotate the repetitive sequence in the *S. angustifolia* genome. This analysis identified a total of 319.98 Mb of repetitive sequences, which

constitutes 50.70% of the *S. angustifolia* genome (Table 1). Among these sequences, long terminal repeats (LTRs) were the most abundant, comprising 42.91% of the genome, followed by DNA transposons (accounting for 2.49% of the genome) and long interspersed nuclear elements (LINEs) (representing 1.64% of the genome) (Table 1, Table S5, and S6).

To facilitate the prediction of protein-coding genes, transcriptome sequencing was performed on the *S. angustifolia* roots, stems, leaves, flowers, and seeds, generating 40.40 Gb of clean data (Table S7). Transcriptome assembly was performed using both reference-based and *de novo* approaches, and the transcripts from both methods were incorporated into the prediction of protein-coding genes. Using a combination of *ab initio* prediction, homology, and transcriptomic evidence, we identified 36,857 protein-coding genes in the *S. angustifolia* genome, with an average exon length of 242.95 base pairs (bp) and an average intron length of 465.57 bp (Table 1). The completeness of the gene set was assessed using BUSCO, revealing a completeness score of 97.71% (92.50% single-copy BUSCOs and 5.20% duplicated BUSCOs) (Table S8). Furthermore, functional annotation indicated that 98.94% of the predicted genes were annotated, with 68.80% and 65.07% being annotated in the Kyoto Encyclopedia of Genes and Genomes (KEGG) and Gene Ontology (GO) databases, respectively (Table S9). Additionally, we identified non-coding RNAs (ncRNAs) within the *S. angustifolia* genome, including 97 miRNAs, 3,048 snRNAs, and 573 tRNAs (Table S10).

Comparative Genomic Analysis Among Leguminous Plants and Arabidopsis

To investigate the evolutionary relationships of *S. angustifolia*, we conducted gene family and phylogenetic analyses across nine leguminous species and the model plant Arabidopsis. Gene clustering analysis identified a total of 31,810 orthologous groups (OGs) across the studied species. Of these, 9,460 OGs were found to be shared by all species. Additionally, 633 OGs were identified as single-copy across all species, while 578 OGs were found to be specific to *S. angustifolia* (Figure 2a).

The phylogenetic tree based on the single-copy OGs showed that *S. angustifolia* is closely related to the wild peanut relatives, *Arachis duranensis* and *A. ipaensis*, with these species forming sister clades (Figure 2b). This relationship further supports the evolutionary position of *S.*

angustifolia within the subtribe *Stylosanthinae* (Benth.) of the legume family. To further explore the dynamics of gene families, we performed gene family expansion and contraction analyses on six well-annotated and extensively studied legume species. The analysis identified 2,089 gene families have expanded in *S. angustifolia*, of which 158 OGs showed statistically significant expansions (*P*-value < 0.05) (Figure S3). These 158 OGs comprise 3,071 genes, primarily associated with KEGG pathways involved in "carbohydrate metabolism", "Biosynthesis of other secondary metabolites", and "lipid metabolism" (Figure S4).

We also identified collinear gene blocks within and between *S. angustifolia*, soybean, *A. duranensis*, and *A.ipaensis*. The genomes of *S. angustifolia*, *A. duranensis*, and *A.ipaensis* exhibit extensive genomic rearrangements, particularly on chromosomes 3, 9, and 10. However, some chromosomal regions maintained strong synteny, such as chromosomes 1, 6, and 8 (Figure 2c). Analysis of the synonymous substitution rate (Ks) distribution among collinear gene pairs revealed that *S. angustifolia* shared the ancestral Papilionoideae whole-genome duplication event (PWGD) with soybean, *A. duranensis*, and *A. ipaensis* (Figure 2d). Similar Ks peaks observed in *S. angustifolia*, *A. duranensis*, and *A. ipaensis* suggest that this duplication event occurred approximately 49.26 million years ago (MYA) (Figure 2d). Furthermore, the divergence time between *S. angustifolia* and the two *Arachis* species were estimated to be 11 MYA, consistent with divergence time derived from single-copy gene family analyses (Figure 2b, d).

Transcriptome Analysis of *S. angustifolia* in Response to Drought Stress

To assess the response of *S. angustifolia* to drought stress, 60-day-old seedlings were subjected to drought treatments for 0 days (D0, control), 3 days (D3), and 5 days (D5) under pot conditions. As the duration of drought treatment increased, the leaves of *S. angustifolia* progressively turned yellow (Figure 3a, b), accompanied by a gradual decrease in chlorophyll a and b content (Figure S5a, b), as well as in shoot water content (Figure S5c). Additionally, soil water content decreased by 62.16% at D3 and by 81.91% at D5 relative to D0 (Figure S5d). To further understand the gene expression dynamics under drought stress, transcriptome sequencing was conducted on root and leaf samples collected at D0, D3, and D5 (Figure 3a). This sequencing yielded a total of 130.05 Gb clean data (average 7.22 Gb per sample) with Q30 greater than 93.54% (Table S11). Gene expression analysis

revealed that 29,215 genes were expressed throughout the drought treatment period. Furthermore, differential gene expression analysis showed that a greater number of genes were differentially expressed in roots and leaves at D5 than at D3 (Figure S6). Among these differentially expressed genes (DEGs), 384 genes were up-regulated in both roots and leaves at D3, while 1,246 genes were up-regulated in roots and leaves at D5 (Figure S7).

An intersection analysis of the expanded gene families and the DEGs revealed 98 up-regulated genes in root and 84 in leaves at D3 (Figure 3c, d). At D5, 155 genes were up-regulated in roots and 166 in leaves (Figure 3e, f). Enrichment analysis of these up-regulated and expanded genes revealed significant enrichment in the "carotenoid biosynthesis pathway" (map00906, Q-value < 0.05) in both roots and leaves (Figure 3g, Table S12 - S15). Further investigation highlighted the critical role of *xanthoxin dehydrogenase* (*ABA2*, K09841), a key gene family involved in ABA biosynthesis. This gene family exhibited significant up-regulated in both roots and leaves under drought stress (Table S13 - S15). Genome-wide identification of *ABA2* genes in *S. angustifolia*, soybean, barrel medic, and Arabidopsis indicated a higher number of *ABA2* genes in the studied leguminous plants compared to Arabidopsis (Table S16). Notably, *ABA2* gene underwent tandem duplication, resulting in the expansion of seven *ABA2* genes on chromosome 4 in *S. angustifolia* (Figure 4a, b). Divergence time analysis indicated these duplications occurred approximately 24.13 MYA, with the most recent duplication around 5.01 MYA (Table S17). Among the seven tandem-duplicated *ABA2* genes, six were up-regulated in roots and four in leaves at D5 (Figure 4a). Quantification of ABA content revealed a 20.43-fold increase in roots and a 5.05-fold increase in leaves at D5 compared to D0, suggesting that *ABA2* genes expansion and up-regulation contributed to ABA biosynthesis under drought stress (Figure 4d, e).

In addition, genes that were expanded and up-regulated in leaves at D5 showed significant enrichment in the "Isoflavonoid biosynthesis pathway" (map00943) (Q-value < 0.05) (Figure 3g). Further analysis identified three *2-hydroxyisoflavanone dehydratase* (*HIDH*, K13258) genes involved in genistein and daidzein biosynthesis (Table S15). Phylogenetic and microsynteny analyses indicated a specific expansion of *HIDH* genes on chromosome 2 through tandem duplication (Figure 5a, b, Table S18), with the most recent duplication occurring at 11.11 MYA (Table S19). Three *HIDH* genes were up-regulated in leaves at D5 (Figure 5a). Consistent with the

transcriptome findings, genistein and daidzein contents in leaves at D5 increased by 448% and 94% at D5, respectively, compared to D0 (Figure 5d, f). However, genistein and daidzein contents in roots showed no significant differences between D0 and D5 (Figure 5c, e).

Contribution of TDGs to Drought Tolerance of *S. angustifolia*

The *S. angustifolia* genome shows no evidence of recent WGDs (Figure 2d), and gene family analysis indicated that tandem duplication plays an important role in gene expansion within the *S. angustifolia* genome (Figure 3, Figure 4, Figure 5). Therefore, we performed a genome-wide identification of TDGs and investigated their response to drought stress in *S. angustifolia*. We identified 3,634 TDGs in the *S. angustifolia* genome, which is more than in *A. duranensis* (2,735) and *A. ipaensis* (3,449), but fewer than in soybean (5,022) and barrel medic (7,032). Analysis of the Ks distribution of TDGs revealed a substantial expansion in *S. angustifolia* approximately 12.31 MYA, with a Ks peak at about 0.2 (Figure 6a).

TDGs in *S. angustifolia* were significantly enriched in KEGG pathways such as "Biosynthesis of secondary metabolites", "Flavonoid biosynthesis", "Isoflavonoid biosynthesis", and "Galactose metabolism" (*Q*-value < 0.05). (Figure 6b). Transcriptomic analysis revealed that three gene families involved in raffinose and stachyose biosynthesis in the galactose metabolism pathway—inositol 3-alpha-galactosyltransferases (*GOLSs*), raffinose synthases (*RAFSs*), and stachyose synthetases (*STSs*)—exhibited significantly increased expression after 3 and 5 days of drought treatment. The most pronounced up-regulation was observed at D5 (Figure 6c and Table S20). Notably, the two *STS* genes, significantly up-regulated in roots at D5, were expanded through tandem duplication (Figure 6c, d).

Consistent with gene expression patterns, raffinose content increased by 129% in roots and 225% in leaves at D5 compared to D0 (Figure 6e, f). Similarly, stachyose content increased by 142% in roots and 99% in leaves at D5 compared to D0 (Figure 6g, h).

Lipid Metabolism in *S. angustifolia* in Response to Drought Stress

Comparative genomic analysis revealed that significantly expanded gene families in *S. angustifolia* are involved in the lipid metabolism pathway (Figure S4). To assess the effects of drought stress on

lipid metabolism in *S. angustifolia*, we conducted a lipidomic analysis on samples from both roots and leaves collected on D0 and D5. A total of 874 lipids were identified in roots and 904 lipids in leaves, spanning six major lipid classes (Figure 7a, b, Table S21, and S22). Among these, 52 lipids in roots and 134 lipids in leaves were identified as differentially accumulated lipids (DALs) at D5 compared to D0 (Figure 7a, b). Notably, 22 triacylglycerols (TAGs) were detected in roots and 29 TAGs in leaves, with 81.81% of TAGs in roots and 89.66% in leaves showing up-regulated at D5 (Figure 7c, d). In plants, TAGs associate with membrane protein families, including oleosins, caleosins, and steroleosins, to form subcellular organelles known as oil bodies, which play a crucial role in regulating lipid metabolism and maintaining lipid homeostasis [40]. Following TAG accumulation at D5, two of the seven oleosin family genes were up-regulated in roots of *S. angustifolia*, while four were up-regulated in leaves (Figure 7e and Table S23). Similarly, after 5 days of drought treatment, all four oleosin family genes in *S. angustifolia* were up-regulated in roots, while three of the four were up-regulated in leaves (Figure 7e). Notably, two of these up-regulated oleosin genes originated from tandem duplications (Figure 7f).

In plants, the degradation of membrane lipids containing C18:3 chains lead to the production of α-linolenic acid, a precursor in the biosynthesis of jasmonic acid (JA) [41]. Lipidomic analyses revealed a significant reduction in four phospholipids, two sulfolipids, twelve galactolipids, and one glucolipid containing C18:3 chains in leaves after 5 days of drought stress (Figure 8a). In contrast, the roots exhibited a significant decrease in only one phospholipid and one galactolipid containing C18:3 chains after 5 days of drought stress (Figure 8b). Consistent with these lipidomic changes, drought treatment for 5 days led to the up-regulation of several gene families involved in the biosynthesis of JA and jasmonoyl-L-isoleucine (JA-Ile) in leaves (Figure 9a, Table S24). Specifically, four members of *patatin-related phospholipase A* (*pPLA*), one member of *phospholipase A1* (*DAD1*), three members of *acyl-CoA oxidase* (*ACX*), three members of *multifunctional protein* (*MFP*), two members of *ketoacyl-CoA thiolase* (*KAT*), and one member of *jasmonate-amido synthetase* (*JAR*) were up-regulated (Figure 9a). Interestingly, the *pPLA* gene underwent tandem duplication approximately 18.67 MYA (Ks = 0.30) (Figure 9b). Quantitative measurements of JA and JA-Ile levels showed substantial increases in leaves, with JA levels increasing by 77% and JA-Ile levels increasing by 447% at D5 compared to D0 (Figure 9d, f). In

contrast, JA and JA-Ile levels were significantly reduced in roots at D5 compared to D0 (Figure 9c, e).

Discussion

Exploring the mechanisms by which pioneer plants adapt to harsh environmental conditions provides valuable insights for enhancing stress tolerance traits in crops [42,43]. Despite the pioneer plant *S. angustifolia* being recognized for its exceptional drought tolerance, the absence of high-quality genomic resources has considerably impeded a comprehensive understanding of its molecular mechanisms underlying drought resistance. In this study, we assembled a high-quality genome of *S. angustifolia* by integrating NGS, ONT, and Hi-C sequencing technologies, yielding a genome size of 645.88 Mb. Our assembly demonstrates high integrity and accuracy, as confirmed by multiple quality assessment metrics (Table 1 and Figure 1). Notably, the *S. angustifolia* genome shows low heterozygosity (0.06%). This high-quality genome resource will facilitate evolutionary, genetic, and functional studies, especially those aimed at advancing molecular breeding programs for drought tolerance.

Gene duplication is essential in plant evolution and adaptation to environmental challenges [29,30]. In particular, tandem duplication is an important feature in the pathways of secondary metabolite biosynthesis and responses to biotic and abiotic stresses [44,45]. For instance, the expansion of sugar metabolism-related genes, including *α-amylase* (*AMY3*) and *β-fructofuranosidase* (*CWINV1*), in *Sophora moorcroftiana* contributes to high sucrose content, promoting long root growth and enhancing drought tolerance [46]. In orchardgrass (*Dactylis glomerata*), tandem duplication of DgMADS-box genes has promoted longer root lengths and higher survival rates under various abiotic stresses [47]. Similarly, in Pearl millet (*Pennisetum glaucum*), expansion of the RWP-RK gene family enables rapid responses to heat stress by regulating the expression of endoplasmic reticulum (ER)-related gene expression [48]. These examples highlight the significance of TDGs in environmental adaptation.

In *S. angustifolia*, TDGs played a crucial role in its drought adaptation, as demonstrated by an integrative comparative genomics, transcriptomics, and metabolomics analysis. ABA, a key phytohormone involved in the drought stress response [49–51], exhibited significantly increased

accumulation in leaves and roots of *S. angustifolia* after 5 days of drought treatment (Figure 4d, e). The expansion of *ABA2* genes through tandem duplication in the *S. angustifolia* genome may enhance ABA biosynthesis under drought stress (Figure 4a, b). Additionally, JA and its active conjugate, JA-Ile, are widely distributed plant hormones involved in resistance to biotic and abiotic stresses, including drought [6,52–56]. Our findings indicate that genes encoding *pPLAs*, which degrade C18:3-branched membrane lipids, have expanded through tandem duplication, likely promoting JA and JA-Ile biosynthesis in *S. angustifolia* leaves under drought conditions (Figure 9).

Raffinose family oligosaccharides (RFOs), including raffinose, stachyose, and verbascose, are α-galactosyl derivatives of sucrose that are common in plants and play important roles in regulating plant responses to abiotic stress [57]. In maize, drought conditions increase raffinose accumulation, and overexpression of *ZmRAFS*, a gene responsible for raffinose biosynthesis, enhances drought tolerance in transgenic plants [7,8]. Similar results were observed in *S. angustifolia*, where *RAFS* family genes were up-regulated in both leaves and roots under drought stress, leading to increased raffinose accumulation (Figure 6c, e, f). Raffinose can be further synthesized into stachyose. We found that stachyose accumulation increased in *S. angustifolia* leaves and roots under drought conditions (Figure 6g, h). Additionally, the *STS* genes responsible for stachyose biosynthesis expanded via tandem duplication and were up-regulated under drought conditions (Figure 6c, d), which may contribute to the drought tolerance mechanism characteristic of *S. angustifolia*. Apart from RFOs, isoflavonoids, such as genistein, have been shown to contribute to enhancing plant drought resistance [4, 5]. In *S. angustifolia*, *HIDH* genes, responsible for the biosynthesis of genistein and daidzein, have underwent expansion through tandem duplication (Figure 5b). After 5 days of drought treatment, genistein and daidzein levels in *S. angustifolia* leaves increased (Figure 5d, f), likely due to the up-regulation of the *HIDH* genes (Figure 5a), suggesting their active role in drought tolerance.

In conclusion, the genome assembly of *S. angustifolia* provides new insights into the genetic basis of drought tolerance. Our study highlights the significant role of tandem duplication in the expansion of key gene families involved in phytohormone biosynthesis, secondary metabolism, and osmoprotection, which collectively enhance the drought adaptation of *S. angustifolia*. These findings advance our understanding of the molecular mechanisms underlying drought tolerance and

offer valuable genomic resources for breeding drought-tolerant crops.

Methods

Plant Materials

S. angustifolia (Germplasm number: TF0003, Figure S1) was provided by the National Tropical Plants Germplasm Resource Center (Hainan, China). Young leaves were harvested for DNA extraction using the CTAB method for both NGS and ONT sequencing. Root, stem, leaf, flower, and seed samples were collected for RNA extraction and transcriptome sequencing.

Genome Sequencing and Assembly

High-quality genomic DNA was extracted for the construction of ONT and NGS libraries. Sequencing was performed at the Genome Center of Grandomics (Wuhan, China). The ONT library was processed using the ONT PromethlON platform, while the NGS library was sequenced using the MGI-SEQ 2000 platform (MGI Tech, China). Hi-C technology was adopted for chromosome-level genome assembly. To achieve chromosome-level assembly, Hi-C technology was employed. Hi-C library construction and sequencing were also performed at the Genome Center of Grandomics (Wuhan, China) with DPN II as the restriction enzyme. The ONT reads were processed with guppy (v6.5.7). The quality control of raw NGS and Hi-C reads was performed using SOAPnuke (v2.1.7; RRID:SCR_015025) using parameters '-n 0.01 -l 20 -q 0.3 --polyX 50'. ONT reads were corrected and assembled into initial contigs using NextDenovo (v2.3.0; RRID:SCR_025033) [58], with "read_cutoff" set to 2k to ensure the high-quality assembly results. Additionally, both NGS and ONT reads were incorporated into further polishing of initial contigs using NextPolish (v1.4.1; RRID:SCR_025232) [59]. The clean Hi-C reads were aligned to the polished contigs using the Burrows-Wheeler Aligner (BWA, v0.7.17; RRID:SCR_010910), and then Hi-C contact maps were generated using Juicer (v1.6). The 3D-DNA (v180922; RRID:SCR_017227) pipeline was adopted for chromosomal grouping, sorting, and orientation. Visualization and manual curation of Hi-C maps were conducted using Juicebox (v1.11.08; RRID:SCR_021172) to ensure the accuracy of chromosome assembly. The completeness of the genome assembly was evaluated using BUSCO

(v5.3.2; RRID:SCR_015008) based on embryophyta_odb10 database, and LAI analysis using LTR_retriever (v2.9.0; RRID:SCR_017623) [60]. Additionally, merqury (v1.3; RRID:SCR_022964) was also adopted for assembly completeness evaluation using an efficient k-mer set. Reads were aligned to the genome using minimap2 (v2.17-r941; RRID:SCR_018550) for ONT and BWA (v0.7.17; RRID:SCR_010910) for NGS. SAMtools (v1.9; RRID:SCR_002105) and PanDepth (v2.19) were adopted for genome mapping ratios and coverage statistics. Based on the NGS mapping results, SNPs were detected using GATK (v4.1.2.0; RRID:SCR_001876), and the heterozygosity rate of *S. angustifolia* was calculated by dividing the number of heterozygous SNPs by the total effective genome bases, then multiplying by 100 to obtain a percentage.

Genome Annotation

We employed both *de novo* and homology-based approaches for the identification of repetitive elements. Extensive de novo TE Annotator (EDTA, v2.0.1; RRID:SCR_022063) [61] was adopted for *de novo* identification of transposable elements (TEs). Additionally, known repetitive elements from RepBase (v21.12; RRID:SCR_021169) were identified using RepeatMasker (v4.1.4; RRID:SCR_012954). Tandem Repeat Finder (TRF, v. 4.09.1; RRID:SCR_022193) was utilized to detect tandem repeats.

Protein-coding gene prediction was performed based on the repeat-masked genome by employing *de novo*-based prediction, RNA-seq-based prediction, and homologue-based prediction. We employed GALBA (v1.0.8) [62] for automated training and prediction of protein-coding genes, utilizing AUGUSTUS (v3.5.0; RRID:SCR_008417) [63] and miniport (v0.13) [64] with default parameters. SNAP (v. 2013-02-16; RRID:SCR_007936) was also employed for *de novo* gene prediction. Transcriptome sequencing reads from roots, stems, leaves, flowers, and seeds were aligned to the *S. angustifolia* genome using HISAT2 (v2.2.1; RRID:SCR_015530), and transcript construction was conducted using StringTie (v2.2.1; RRID:SCR_016323) [65]. RNA *de novo* assembly was performed using Trinity (v2.15.1; RRID:SCR_013048). Coding regions of the predicted transcripts were identified using TransDecoder (v 5.7.0; RRID:SCR_017647). For homology-based prediction, proteins from five species, namely, *G. max*, *Medicago truncatula*, *A. ipaensis*, *Senna tora*, and *Arabidopsis thaliana*, were aligned to the *S. angustifolia* genome using

tBLASTn (v2.13.0; RRID:SCR_011822) and gene structure prediction were performed by miniport (v0.13). EVidenceModeler (v2.1.0; RRID:SCR_014659) [66] was adopted for the identification of non-redundance consensus genes from all available evidence. Furthermore, PASA (v2.5.3; RRID:SCR_014656) was adopted to refine gene structure and annotate untranslated regions (UTRs) based on transcriptome data. The predicted protein-coding genes were aligned to various known databases, including NCBI Non-Redundant Protein Sequence Database (NR), KEGG, Eukaryotic Orthologous Groups of Protein (KOG), Swiss-Prot, TrEMBL, InterPro databases, for functional annotation. BLASTp (v2.13.0; RRID:SCR_001010) was employed for homology searches against NR, KEGG, KOG, Swiss-Prot, and TrEMBL using the parameters '-outfmt 6 -evalue 1e-10'. Blast2GO (v6.0; RRID:SCR_005828) was adopted for GO annotation based on the NR annotation, The best hit for each gene was retained for subsequent analysis. Additionally, non-coding genes (ncRNAs) were predicted by BLASTn (v2.13.0; RRID:SCR_001598) and INFERNAL (v1.0; RRID:SCR_011809) based on the Rfam database (v12.0; RRID:SCR_007891).

Comparative Genomic Analysis

Nine legumes, including *S. angustifolia, A. ipaensis*, *Arachis duranensis*, *Aeschynomene evenia*, *G. max*, *M. truncatula*, *Phaseolus vulgaris*, *Cajanus cajan*, and *Senna tora*, along with model species *Arabidopsis thaliana*, were utilized for comparative analysis. Protein sequences from these species were compared through all- versus-all alignments using BLASTp (v2.2.23, e-value set to 1e-5; RRID:SCR_001010). OrthoFinder (v2.5.4; RRID:SCR_017118) was utilized to identify orthologous groups (OGs) and construct phylogenetic tree using parameters '-S diamond -M msa -A mafft'. Divergence times between *G. max*, *M. truncatula*, *C. cajan*, and *P. vulgaris* were queried on TimeTree (http://www.timetree.org/) as known divergence times. Based on single-copy OGs, the substitution rates were estimated using the MCMCTREE program within the PAML (v4.5; RRID:SCR_014932) software package, which was further used to calculate the divergence times between species. MCScanX (RRID:SCR_022067) [67] was employed for intra- and inter-species gene collinearity analysis. First, protein sequences were aligned using BLASTp (v2.2.23, e-value set to 1e-5; RRID:SCR_001010), and MCScanX (RRID:SCR_022067) [67] was employed to identify collinear regions. For intra-species analysis, we employed the 'duplicate_gene_classifier'

from MCScanX to classify paralogous genes into categories such as single-copy genes, dispersed duplicated genes, proximal duplicated genes, TDGs, and whole genome or segmental duplicated genes. Microcollinearity of TDGs in the *S. angustifolia* genome was compared with that of soybean and barrel medic using the MCscan-(Python-version) [68]. Additionally, the non-synonymous (Ka) and synonymous (Ks) substitution rates of gene pairs within the collinear regions, as well as the Ka and Ks of TDGs were calculated using PAML (v4.9e; RRID:SCR_014932) and PAL2NAL (v14) using the Nei-Gojobori (NG) method [69]. The R platform (v4.0.2; RRID:SCR_001905) was adopted for Ks distribution visualization and peak identification. The divergence time of gene pairs was calculated using the formula T=Ks/2r, where the neutral substitution rate r was selected as 8.12 $\times 10^{-9}$ in this study [70].

Gene Identification and Phylogenetic Analysis

Protein sequences from *S. angustifolia*, soybean, and barrel medic were aligned against The Arabidopsis Information Resource (TAIR) database. Genes with an identity greater than 35% and coverage exceeding 50% were retained for further analysis of metabolic pathways. For the phylogenetic analysis, protein sequences from studied gene families were aligned using MUSCLE (v5.2; RRID:SCR_011812), and a phylogenetic tree was conducted using FastTree version (v2.1.11; RRID:SCR_015501) with the approximately-maximum-likelihood method. The resulting phylogenetic trees were visualized using iTOL (v6; RRID:SCR_018174) [71].

Drought Treatment on *S. angustifolia*

After seed germination, *S. angustifolia* seeds were planted in pots (30 cm in height, 25 cm in diameter) filled with sandy soil. The Drought treatment was initiated when the seedlings reached 60 days old by withholding watering. Leaf and root samples were collected at day 0 (D0) for the control group, and at days 3 (D3) and 5 (D5) after the initiation of drought treatment.

Soil water content was determined using previously reported methods [72–74]. Briefly, the total soil in each pot was recorded to obtain the wet weight (Ww) after plant harvesting. The soil was then dried at 65°C for 72 hours to obtain the dry weight (Wd). The soil water content was calculated using the formula: (Ww – Wd) / Ww ×100%. The soil water content at D0 was

normalized to 100% of pot capacity (PC), while the corresponding soil water contents at D3 and D5 were expressed as percentages of this normalized value. Shoot water content was measured following previously described methods [75]. In brief, the shoot was harvested and weighed to obtain the fresh weight (FW). After drying at 65°C until a constant weight was reached, the tissues were weighed again to determine the dry weight (DW). The water content, expressed as a percentage of fresh weight (% FW), was calculated using the formula: (FW – DW) / FW ×100%. Chlorophyll content was measured according to previously published methods [76]. For RNA sequencing, metabolite quantification, and lipidomics analysis, three biological replicates of root and leaf samples were collected.

Transcriptome Sequencing and Bioinformatics Analysis

RNA was extracted from root and leaf samples collected at D0, D3, and D5 during the drought treatment for transcriptome sequencing. Each treatment group in this experiment consisted of three biological replicates, with each biological replicate containing 20 seedlings. Transcriptomes were sequenced on the MGI-SEQ 2000 platform (MGI Tech, China) and SOAPnuke (v2.1.7; RRID:SCR_015025) [77] was adopted for quality control of the raw sequencing reads. Clean reads were mapped onto the *S. angustifolia* genome using HISAT2 (v2.2.1; RRID:SCR_015530). The featureCounts (v2.0.6; RRID:SCR_012919) were employed for the calculation of gene read counts and an in-house Perl script was adopted for the calculation of transcripts per million (TPM). DEGs were identified using DESeq2 (v3.19; RRID:SCR_015687), and the Benjamini-Hochberg (BH) method was adopted for false discovery rate (FDR) calculation. Genes with a $|\log_2$ fold change$| > 1$ and an adjusted P-value (*Padj*) < 0.05 were considered as DEGs. KEGG pathway enrichment analysis of DEGs was performed by the phyper and *p.adjust* functions under the R platform (v4.0.2; RRID:SCR_001905) and KEGG pathways with a *Q*-value < 0.05 were considered as significantly enriched pathways. Gene expression heatmaps were generated under the R platform (v4.0.2; RRID:SCR_001905).

Lipidomics Analysis of *S. angustifolia* under Drought Stress

Lipidomics analysis was conducted on root and leaf samples of *S. angustifolia* by Biotree

Biomedical Technology Co., Ltd. (Shanghai, China). Sample extraction was performed with slight modifications to a previously reported method [78]. Briefly, freeze-dried samples were extracted using the flowing extracting solution: MTBE: MeOH= 5:1 (v/v) containing an isotope-labeled internal standard. Subsequently, 100 μL of the extracted supernatant was transferred to the injection bottle for lipid metabolite detection.

The chromatographic separation of the target compounds was performed using a Phenomenex Kinetex C18 column (2.1 mm × 100 mm, 2.6 μm) on a Vanquish ultra-performance liquid chromatograph (Thermo Fisher Scientific). The mobile phase A consisted of 40% water and 60% acetonitrile with 10 mmol/L ammonium formate, while phase B comprised 10% acetonitrile and 90% isopropanol, supplemented with 50 mL of 10 mmol/L ammonium formate aqueous solution per liter. The injection volume was set at 2 μL. Mass spectrometric analysis was performed on an Orbitrap Exploris 120, allowing for both primary and secondary mass spectrometry data acquisition using Xcalibur (v4.4). The operational parameters were as follows: sheath gas flow rate at 30 Arb, auxiliary gas flow rate at 10 Arb, capillary temperature at 320 °C (both positive and negative modes), full MS resolution at 60,000, MS/MS resolution at 15,000, collision MS resolution at 15,000, collision energy at 15/30/45 in NCE mode, and spray voltage at 3.8 kV (positive) or -3.4 kV (negative).

The raw mass spectrum data were converted to mzXML format using ProteoWizard (RRID:SCR_012056) software. XCMS was then used for retention time correction, peak identification, extraction, integration, and alignment. The minimum fraction (minfrac) was set to 0.5 and the cutoff was set to 0.3. Lipid identification was performed through a spectral match using the LipidBlast library within the XCMS (RRID:SCR_015538) software [79].

MetaboAnalyst (v6.0; RRID:SCR_015539) [80] was adopted for lipids analysis. Lipids with a fold change of ≥ 2 or ≤ 0.5 in relative abundance between D5 and D0, along with variable importance for projection (VIP) score > 1 and an adjusted *P*-value (*Padj*) < 0.05, were identified as differentially accumulated lipids (DALs). Each experimental group had three biological replicates.

Determination of ABA, JA and JA-Ile, Genistein, Daidzein, Raffinose, and Stachyose

Quantitative assays of plant hormones (ABA, JA, and JA-Ile) in roots and leaves of *S. angustifolia*

were conducted by Biotree Biomedical Technology Co., Ltd. (Shanghai, China). A total of 100 mg of the freeze-dried samples was weighed and extracted using 1 mL of ice-cold 50% acetonitrile (ACN) aqueous solution. The sample was sonicated at 4°C for 3 minutes, followed by extraction at 4°C for an additional 30 minutes. The mixture was centrifuged at 12,000 rpm for 10 minutes at 4°C, and the supernatant was collected. The sample was then passed through an RP-SPE column: 1 mL of 100% methanol (MeOH) and 1 mL of deionized water were added, then the column was equilibrated with 50% ACN aqueous solution. The sample was loaded onto the column, which was washed with 1 mL of 30% ACN, and the eluent was collected. The sample was evaporated to dryness under a nitrogen stream, dissolved in 200 μL of 30% ACN, and transferred to a sample vial with an insert.

The data acquisition system primarily consisted of ultra-high-performance liquid chromatography (UPLC, Vanquish, Thermo, USA) coupled with a high-resolution mass spectrometer (Q Exactive, Thermo, USA). The liquid chromatography parameters were set as follows: chromatographic column: Waters HSS T3 (50 × 2.1 mm, 1.8 μm); mobile phase: phase A was ultrapure water (containing 0.1% acetic acid), and phase B was acetonitrile (containing 0.1% acetic acid); flow rate: 0.3 mL/min; column temperature: 40°C; injection volume: 2 μL; elution gradient: 0 min water/acetonitrile (90:10, v/v), 1 min water/acetonitrile (90:10, v/v), 5 min water/acetonitrile (10:90, v/v), 7 min water/acetonitrile (10:90, v/v), 7.1 min water/acetonitrile (90:10, v/v), 9 min water/acetonitrile (90:10, v/v). During the entire analysis, samples were kept in an autosampler at 4°C. To avoid signal fluctuation impacts, samples were analyzed in random sequence. QC samples were inserted into the sample queue to monitor and evaluate system stability and data reliability. Data acquisition was performed using the Q Exactive high-resolution mass spectrometer (Thermo Fisher Scientific, USA). The electrospray ionization (ESI) conditions were as follows: sheath gas 40 arb; auxiliary gas 10 arb; spray voltage 3000V; temperature 350°C; ion transfer tube temperature 320°C. The scan mode was set to single ion monitoring (SIM) in positive ion mode. with a primary scan m/z range was 100-500. Mass spectrometry data were processed using TraceFinder software.

Quantitative assays of raffinose and stachyose in roots and leaves of *S. angustifolia* were conducted by Biotech-Pack-Analytical Inc. (Beijing, China). A total of 300 mg of freeze-dried

samples was weighed and extracted twice with 5 mL of 80% ethanol at 85°C for 30 minutes each time. Following each extraction, the mixture was centrifuged at 12,000 rpm for 5 minutes. The combined supernatants were collected and evaporated to dryness using a vacuum centrifuge. The resulting dried residue was resuspended in 300 μL of distilled water and centrifuged again at 12,000 rpm for 10 minutes. The final supernatant was collected for high-performance liquid chromatography (HPLC) analysis. The liquid chromatography analysis was performed using a Waters 2695 HPLC coupled with a Waters 2424 evaporative light-scattering detector (ELSD). The chromatographic conditions were as follows: column temperature: 40 °C; flow rate: 1.0 mL/min; injection volume: 3 μL of sample; chromatographic column: Sepax HP-Amino (4.6 x 250 mm, 5 μm, 120 Å); mobile phase: acetonitrile: water (70:30) with isocratic elution; total run time: 20 minutes.

Quantitative assays of genistein and daidzein in leaves and roots of *S. angustifolia* were conducted as described in a previous report [78].

Abbreviations

Gb: gigabase; Mb: megabase; Bp: base pair; ABA: abscisic acid; ONT: Oxford Nanopore Technologies; NGS: next-generation sequencing; TDG: tandem duplicated gene; WGD: whole-genome duplication; Hi-C: high-through chromosome conformation capture; GATK: genome analysis toolkit; BUSCO: Benchmarking Universal Single-Copy Orthologs; LTR: long terminal repeat; LAI: LTR assembly index; KEGG: Kyoto Encyclopedia of Genes and Genomes; GO: Gene Ontology; OG: orthologous group; MYA: million years ago; DEG: differentially expressed gene; HIDH: 2-hydroxyisoflavanone dehydratase; ABA2: xanthoxin dehydrogenase; GOLS: inositol 3-alpha-galactosyltransferases; RAFS: raffinose synthases; STS: stachyose synthetases; DALs: differentially accumulated lipids; TAG: triacylglycerol; JA: jasmonic acid; JA-Ile: jasmonoyl-L-isoleucine; pPLA: patatin-related phospholipase A; DAD1: phospholipase A1; ACX: acyl-CoA oxidase; MFP: multifunctional protein; KAT: ketoacyl-CoA thiolase; JAR: jasmonate-amido synthetase; AMY3: α-amylase; CWINV1: β-fructofuranosidase; EDTA: Extensive de novo TE Annotator; UTR: untranslated region; NR: NCBI Non-Redundant Protein Sequence Database; KOG: Eukaryotic Orthologous Groups of Protein; Ka: non-synonymous; Ks: synonymous.

Data Availability

The raw genomic sequencing data, including ONT, NGS, and Hi-C data, as well as transcriptome data, have been deposited in the National Genomics Data Center (NGDC) [81] under BioProject PRJCA027610. The raw sequence data have been deposited in the Genome Sequence Archive in the National Genomics Data Center, China National Center for Bioinformation / Beijing Institute of Genomics, Chinese Academy of Sciences (GSA: CRA017744) [82]. The assembly and annotation of *S. angustifolia* have been deposited in the Genome Warehouse in the National Genomics Data Center under accession number GWHFHGI00000000.1 [83]. The raw sequencing data can also be accessed through NCBI under the accession number PRJNA1140667. All supporting data and materials are available in the *GigaScience* GigaDB database [84].

Competing Interests

The authors declare that they have no competing interests.

Author Contributions

P.L. conceived the project and designed the experiments. C.L. performed genome assembly, annotation, transcriptome, and lipidome analyses. G.L. provided funding and *S. angustifolia* germplasm. L.L. performed supervision. J. Z., R. X., and J. L. planted and collected samples. S.Z., M.B., and Z.Q. provided technical support and suggestions on manuscript revision. C.L. performed the manuscript. P.L. revised the manuscript. All authors read and approved the final manuscript.

Funding

The research was financially supported by the Natural Science Foundation of Hainan Province (323CXTD387), the National Natural Science Foundation of China (32371769), the Earmarked fund for China Agriculture Research System—Forage and Grass (CARS-34), the Earmarked fund for China Agriculture Research System—Green Manure (CARS-22), the Central Public-interest Scientific Institution Basal Research Fund for CATAS (1630032022023, 1630032024016), and the Guangxi Special Project for Innovation-driven Development (Guike AA18242040).

References

1. Gupta A, Rico-Medina A, Caño-Delgado AI. The physiology of plant responses to drought. Science 2020;368:266–269. https://doi.org/10.1126/science.aaz7614.

2. Vadez V, Grondin A, Chenu K, et al. Crop traits and production under drought. Nat Rev Earth Environ 2024;5:211–225. https://doi.org/10.1038/s43017-023-00514-w.

3. Wang X, Li Q, Xie J, et al. Abscisic acid and jasmonic acid are involved in drought priming-induced tolerance to drought in wheat. Crop Journal 2021;9:120–132. https://doi.org/10.1016/j.cj.2020.06.002.

4. Yang W, Li N, Fan Y, et al. Transcriptome analysis reveals abscisic acid enhancing drought resistance by regulating genes related to flavonoid metabolism in pigeon pea. Environ Exp Bot 2021;191:104627. https://doi.org/10.1016/j.envexpbot.2021.104627.

5. Meng D, Dong B, Niu L, et al. The pigeon pea CcCIPK14-CcCBL1 pair positively modulates drought tolerance by enhancing flavonoid biosynthesis. Plant J 2021;106:1278–1297. https://doi.org/10.1111/tpj.15234.

6. Wen D, Zheng Y, Han Y, et al. Sodium selenite increases drought tolerance by promoting jasmonic acid biosynthesis in cucumber. HORTIC ADV 2023;1:6. https://doi.org/10.1007/s44281-023-00009-0.

7. Li T, Zhang Y, Liu Y, et al. Raffinose synthase enhances drought tolerance through raffinose synthesis or galactinol hydrolysis in maize and Arabidopsis plants. J Biol Chem 2020;295:8064–8077. https://doi.org/10.1074/jbc.RA120.013948.

8. Liu Y, Li T, Zhang C, et al. Raffinose positively regulates maize drought tolerance by reducing leaf transpiration. Plant J 2023;114:55–67. https://doi.org/10.1111/tpj.16116.

9. Hu H, Xiong L. Genetic engineering and breeding of drought-resistant crops. Annu Rev Plant Biol 2014;65:715–41. https://doi.org/10.1146/annurev-arplant-050213-040000.

10. Yang Z, Qin F. The battle of crops against drought: Genetic dissection and improvement. J Integr Plant Biol 2023;65:496–525. https://doi.org/10.1111/jipb.13451.

11. He Z, Zhang P, Jia H, et al. Regulatory mechanisms and breeding strategies for crop drought resistance. New Crops 2024;1:2949–9526. https://doi.org/10.1016/J.NCROPS.2024.100029.

12. Marques A, Moraes L, Dos Santos MA, et al. Origin and parental genome characterization of the allotetraploid *stylosanthes scabra* vogel (Papilionoideae, Leguminosae), an important legume pasture crop. Ann Bot 2018;122:1143–1159. https://doi.org/10.1093/aob/mcy113.

13. Cameron D. Chromosome number and morphology of some introduced *Strylosanthes* species. Crop & Pasture Science 1967;18:375–379. https://doi.org/10.1071/AR9670375.

14. Miller CP, Rains JP, Shaw KA, et al. Commercial development of *Stylosanthes pastures* in northern Australia. II. *Stylosanthes* in the northern Australian Beef Industry. Tropical Grasslands 1997; 31:509–514. https://era.dpi.qld.gov.au/id/eprint/12536/.

15. Luo J, Chen Z, Huang R, et al. Multi-omics analysis reveals the roles of purple acid phosphatases in organic phosphorus utilization by the tropical legume *Stylosanthes guianensis*. Plant J 2024; 117:729–746. https://doi.org/10.1111/tpj.16526.

16. Song J, Zou X, Liu P, et al. Differential expressions and enzymatic properties of malate dehydrogenases in response to nutrient and metal stresses in *Stylosanthes guianensis*. Plant Physiol Biochem 2022;170:325–337. https://doi.org/10.1016/j.plaphy.2021.12.012.

17. Lin Y, Liu G, Liu P, et al. Border-like cell formation mediated by SgPG1 confers aluminum resistance in *Stylosanthes guianensis*. Plant J. 2024. https://doi.org/10.1111/tpj.17073.

18. Sun L, Liang C, Chen Z, et al. Superior aluminium (Al) tolerance of *Stylosanthes* is achieved mainly by malate synthesis through an Al-enhanced malic enzyme, SgME1. New Phytol 2014;202:209–219. https://doi.org/10.1111/nph.12629.

19. Jia Y, Li X, Liu Q, et al. Physiological and transcriptomic analyses reveal the roles of secondary metabolism in the adaptive responses of *Stylosanthes* to manganese toxicity. BMC Genomics 2020;21:861. https://doi.org/10.1186/s12864-020-07279-2.

20. Liu P, Huang R, Hu X, et al. Physiological responses and proteomic changes reveal insights into *Stylosanthes* response to manganese toxicity. BMC Plant Biol 2019;19:212. https://doi.org/10.1186/s12870-019-1822-y.

21. Ribeiro RP, Costa LC, Medina EF, et al. Ethylene coordinates seed germination behavior in response to low soil pH in *Stylosanthes humilis*. Plant Soil 2018;425:87–100. https://doi.org/10.1007/s11104-018-3572-2.

22. Wu Y, Zhao C, Zhao X, et al. Multi-omics-based identification of purple acid phosphatases and

metabolites involved in phosphorus recycling in stylo root exudates. Int J Biol Macromol 2023;241:124569. https://doi.org/10.1016/j.ijbiomac.2023.124569.

23. Braga GJ, Ramos AKB, Carvalho MA, et al. Liveweight gain of beef cattle in *Brachiaria brizantha* pastures and mixtures with *Stylosanthes guianensis* in the Brazilian savannah. Grass Forage Sci 2020;75:206–215. https://doi.org/10.1111/gfs.12473.

24. Schultze-Kraft R, Hubiao Y, Jun T, et al. *Stylosanthes guianensis* CIAT 184 – review of a tropical forage legume. Tropical Grasslands-Forrajes Tropicales 2023:11:95–120. https://doi.org/10.17138/tgft(11)95-120.

25. Huang Y, Wang H, Zhu Y, et al. THP9 enhances seed protein content and nitrogen-use efficiency in maize. Nature 2022;612:292–300. https://doi.org/10.1038/s41586-022-05441-2.

26. Qi X, Li MW, Xie M, et al. Identification of a novel salt tolerance gene in wild soybean by whole-genome sequencing. Nat Commun 2014;5:4340. https://doi.org/10.1038/ncomms5340

27. Zhang T, Peng W, Xiao H, et al. Population genomics highlights structural variations in local adaptation to saline coastal environments in woolly grape. J Integr Plant Biol 2024;66:1408–1426. https://doi.org/10.1111/jipb.13653.

28. Maass BL, Sawkins MC. History, relationships and diversity among *Stylosanthes* species of commercial significance. In: Chakraborty S (ed) High-yielding anthracnose-resistant *Stylosanthes* for agricultural systems. Australian Centre for International Agricultural Research, Canberra 2004:9–26. https://www.cabidigitallibrary.org/doi/full/10.5555/20043151632.

29. Nei M. Gene duplication and nucleotide substitution in evolution. Nature 1969;221:40–2. https://doi.org/10.1038/221040a0.

30. Long M, Betrán E, Thornton K, et al. The origin of new genes: Glimpses from the young and old. Nat Rev Genet 2003;4:865–75. https://doi.org/10.1038/nrg1204.

31. Innan H, Kondrashov F. The evolution of gene duplications: Classifying and distinguishing between models. Nat Rev Genet 2010;11:97–108. https://doi.org/10.1038/nrg2689.

32. Liu C, Wu Y, Liu Y, et al. Genome-wide analysis of tandem duplicated genes and their contribution to stress resistance in pigeonpea (*Cajanus cajan*). Genomics 2021;113:728–735. https:doi.org/10.1016/j.ygeno.2020.10.003.

33. Liu C, Tai Y, Luo J, et al. Integrated multi-omics analysis provides insights into genome

evolution and phosphorus deficiency adaptation in pigeonpea (*Cajanus cajan*). Hortic Res 2022;9:uhac107. https://doi.org/10.1093/hr/uhac107.

34. Qu J, Liu L, Guo Z, et al. The ubiquitous position effect, synergistic effect of recent generated tandem duplicated genes in grapevine, and their co-response and overactivity to biotic stress. Fruit Research 2023;3:16. https://doi.org/10.48130/FruRes-2023-0016.

35. Salojärvi J, Smolander OP, Nieminen K, et al. Genome sequencing and population genomic analyses provide insights into the adaptive landscape of silver birch. Nat Genet 2017;49:904–912. https://doi.org/10.1038/ng.3862.

36. Hanada K, Zou C, Lehti-Shiu MD, et al. Importance of lineage-specific expansion of plant tandem duplicates in the adaptive response to environmental stimuli. Plant Physiol 2008;148:993–1003. https://doi.org/10.1104/pp.108.122457.

37. Liu JN, Fang H, Liang Q, et al. Genomic analyses provide insights into the evolution and salinity adaptation of halophyte *Tamarix chinensis*. Gigascience 2022;12:giad053. https://doi.org/10.1093/gigascience/giad053.

38. Wang M, Yuan J, Qin L, et al. TaCYP81D5, one member in a wheat cytochrome P450 gene cluster, confers salinity tolerance via reactive oxygen species scavenging. Plant Biotechnol J 2020;18:791–804. https://doi.org/10.1111/pbi.13247.

39. Wang X, Gao Y, Wu X, et al. High-quality evergreen azalea genome reveals tandem duplication-facilitated low-altitude adaptability and floral scent evolution. Plant Biotechnol J 2021;19:2544–2560. https://doi.org/10.1111/pbi.13680.

40. Shimada TL, Hayashi M, Hara-Nishimura I. Membrane Dynamics and Multiple Functions of Oil Bodies in Seeds and Leaves. Plant Physiol 2018;176:199-207. https://doi.org/10.1104/pp.17.01522.

41. Wan S, Xin XF. Regulation and integration of plant jasmonate signaling: a comparative view of monocot and dicot. J Genet Genomics 2022;49:704–714. https://doi.org/10.1016/j.jgg.2022.04.002.

42. Li X, Bai W, Yang Q, et al. The extremotolerant desert moss *Syntrichia caninervis* is a promising pioneer plant for colonizing extraterrestrial environments. The Innovation 2024;5:100657. https://doi.org/10.1016/J.XINN.2024.100657.

43. Lyu S, Mei Q, Liu H, et al. Genome assembly of the pioneer species *Plantago major* L.

(Plantaginaceae) provides insight into its global distribution and adaptation to metal-contaminated soil. DNA Research 2023;30:dsad013. https://doi.org/10.1093/dnares/dsad013.

44. Xu Z, Pu X, Gao R, et al. Tandem gene duplications drive divergent evolution of caffeine and crocin biosynthetic pathways in plants. BMC Biol 2020;18:63. https://doi.org/10.1186/s12915-020-00795-3.

45. Cannon SB, Mitra A, Baumgarten A, et al. The roles of segmental and tandem gene duplication in the evolution of large gene families in *Arabidopsis thaliana*. BMC Plant Biol 2004;4:10. https://doi.org/10.1186/1471-2229-4-10.

46. Yin X, Yang D, Liu Y, et al. *Sophora moorcroftiana* genome analysis suggests association between sucrose metabolism and drought adaptation. Plant Physiol 2023;191:844–848. https://doi.org/10.1093/plphys/kiac558.

47. Yang Z, Nie G, Feng G, et al. Genome-wide identification of MADS-box gene family in orchardgrass and the positive role of DgMADS114 and DgMADS115 under different abiotic stress. Int J Biol Macromol 2022;223:129–142. https://doi.org/10.1016/j.ijbiomac.2022.11.027.

48. Yan H, Sun M, Zhang Z, et al. Pangenomic analysis identifies structural variation associated with heat tolerance in pearl millet. Nat Genet 2023;55:507–518. https://doi.org/10.1038/s41588-023-01302-4.

49. Herrera-Vásquez A, Salinas P, Holuigue L. Salicylic acid and reactive oxygen species interplay in the transcriptional control of defense genes expression. Front Plant Sci 2015;6:171. https://doi.org/10.3389/fpls.2015.00171.

50. Shu S, Gao P, Li L, et al. Abscisic acid-induced H_2O_2 accumulation enhances antioxidant capacity in pumpkin-grafted cucumber leaves under $Ca(NO_3)_2$ stress. Front Plant Sci 2016;7:1489. https://doi.org/10.3389/fpls.2016.01489.

51. Kim TH, Böhmer M, Hu H, et al. Guard cell signal transduction network: Advances in understanding abscisic acid, CO_2, and Ca_2^+ signaling. Annu Rev Plant Biol 2010;61:561–91. https://doi.org/10.1146/annurev-arplant-042809-112226.

52. Song S, Qi T, Wasternack C, et al. Jasmonate signaling and crosstalk with gibberellin and ethylene. Curr Opin Plant Biol 2014;21:112–119. https://doi.org/10.1016/j.pbi.2014.07.005.

53. Gupta A, Bhardwaj M, Tran L-SP. JASMONATE ZIM-DOMAIN Family Proteins: Important

Nodes in Jasmonic Acid-Abscisic Acid Crosstalk for Regulating Plant Response to Drought. Curr Protein Pept Sci 2021;22:759–766. https://doi.org/10.2174/1389203722666211018114443.

54. Mahmud S, Ullah C, Kortz A, et al. Constitutive expression of JASMONATE RESISTANT 1 induces molecular changes that prime the plants to better withstand drought. Plant Cell Environ 2022;45:2906–2922. https://doi.org/10.1111/pce.14402.

55. Staswick PE, Tiryaki I. The oxylipin signal jasmonic acid is activated by an enzyme that conjugates it to isoleucine in Arabidopsis. Plant Cell 2004;16:2117–27. https://doi.org/10.1105/tpc.104.023549.

56. Howe GA, Major IT, Koo AJ. Modularity in Jasmonate Signaling for Multistress Resistance. Annu Rev Plant Biol 2018;69:387–415. https://doi.org/10.1146/annurev-arplant-042817-040047.

57. Yan S, Liu Q, Li W, et al. Raffinose Family Oligosaccharides: Crucial Regulators of Plant Development and Stress Responses. Critical Reviews in Plant Sciences 2022;41:286–303. https://doi.org/10.1080/07352689.2022.2111756.

58. Hu J, Wang Z, Sun Z, et al. NextDenovo: an efficient error correction and accurate assembly tool for noisy long reads. Genome Biol 2024;25:107. https://doi.org/10.1186/s13059-024-03252-4.

59. Hu J, Fan J, Sun Z, et al. NextPolish: A fast and efficient genome polishing tool for long-read assembly. Bioinformatics 2020;36:2253–2255. https://doi.org/10.1093/bioinformatics/btz891.

60. Ou S, Jiang N. LTR_retriever: A highly accurate and sensitive program for identification of long terminal repeat retrotransposons. Plant Physiol 2018;176:1410–1422. https://doi.org/10.1104/pp.17.01310.

61. Ou S, Su W, Liao Y, et al. Benchmarking transposable element annotation methods for creation of a streamlined, comprehensive pipeline. Genome Biol 2019;20:275. https://doi.org/10.1186/s13059-019-1905-y.

62. Brůna T, Li H, Guhlin J, et al. Galba: genome annotation with miniprot and AUGUSTUS. BMC Bioinformatics 2023;24:327. https://doi.org/10.1186/s12859-023-05449-z.

63. Hoff KJ, Stanke M. Predicting Genes in Single Genomes with AUGUSTUS. Curr Protoc Bioinformatics 2019;65:e57. https://doi.org/10.1002/cpbi.57.

64. Li H. Protein-to-genome alignment with miniprot. Bioinformatics 2023;39:btad014. https://doi.org/10.1093/bioinformatics/btad014.

65. Pertea M, Kim D, Pertea GM, et al. Transcript-level expression analysis of RNA-seq experiments with HISAT, StringTie and Ballgown. Nat Protoc 2016;11:1650–67. https://doi.org/10.1038/nprot.2016.095.

66. Haas BJ, Salzberg SL, Zhu W, et al. Automated eukaryotic gene structure annotation using EVidenceModeler and the Program to Assemble Spliced Alignments. Genome Biol 2008;9:R7. https://doi.org/10.1186/gb-2008-9-1-r7.

67. MCScanX (2024). MCScanX https://github.com/wyp1125/MCScanX.

68. Tang H, Krishnakumar V, Zeng X, et al. JCVI: A versatile toolkit for comparative genomics analysis. Imeta 2024;3:e211. https://doi.org/10.1002/imt2.211.

69. Nei M, Gojobori T. Simple methods for estimating the numbers of synonymous and nonsynonymous nucleotide substitutions. Mol Biol Evol 1986;3:418–26. https:doi/org/10.1093/oxfordjournals.molbev.a040410.

70. Bertioli DJ, Cannon SB, Froenicke L, et al. The genome sequences of *Arachis duranensis* and *Arachis ipaensis*, the diploid ancestors of cultivated peanut. Nat Genet 2016;48:438–46. https://doi.org/10.1038/ng.3517.

71. Letunic I, Bork P. Interactive Tree of Life (iTOL) v6: recent updates to the phylogenetic tree display and annotation tool. Nucleic Acids Res 2024;52:W78-W82. https://doi.org/10.1093/nar/gkae268.

72. Boyle RK, McAinsh M, Dodd IC. Stomatal closure of *Pelargonium* × *hortorum* in response to soil water deficit is associated with decreased leaf water potential only under rapid soil drying. Physiol Plant 2016;156:84–96. https://doi.org/10.1111/ppl.12346.

73. Boyle RK, McAinsh M, Dodd IC. Daily irrigation attenuates xylem abscisic acid concentration and increases leaf water potential of *Pelargonium* × *hortorum* compared with infrequent irrigation. Physiol Plant 2016;158:23–33. https://doi.org/10.1111/ppl.12433.

74. Turner, N.C. Imposing and maintaining soil water deficits in drought studies in pots. Plant Soil 2019;439:45–55. https://doi.org/10.1007/s11104-018-3893-1.

75. Liang X, Liu S, Wang T, et al. Metabolomics-driven gene mining and genetic improvement of tolerance to salt-induced osmotic stress in maize. New Phytol 2021;230:2355–2370. https://doi.org/10.1111/nph.17323.

76. Lichtenthaler H K, Wellburn AR. Determinations of total carotenoids and chlorophylls a and b of leaf extracts in different solvents. Biochemical Society Transactions 1983;11:591–592. https://doi.org/10.1042/BST0110591.
77. Chen Y, Chen Y, Shi C, et al. SOAPnuke: A MapReduce acceleration-supported software for integrated quality control and preprocessing of high-throughput sequencing data. Gigascience 2018;7:1–6. https://doi.org/10.1093/gigascience/gix120.
78. Liu C, Huang R, Zhao X, et al. Comparative analysis of lipid and flavonoid biosynthesis between Pongamia and soybean seeds: genomic, transcriptional, and metabolic perspectives. Biotechnol Biofuels Bioprod 2024;17:86. https://doi.org/10.1186/s13068-024-02538-w.
79. Smith CA, Want EJ, O'Maille G, et al. XCMS: Processing mass spectrometry data for metabolite profiling using nonlinear peak alignment, matching, and identification. Anal Chem 2006;78:779–87. https://doi.org/10.1021/ac051437y.
80. Pang Z, Lu Y, Zhou G, et al. MetaboAnalyst 6.0: towards a unified platform for metabolomics data processing, analysis and interpretation. Nucleic Acids Res 2024;52:W398–W406. https://doi.org/10.1093/nar/gkae253.
81. CNCB-NGDC Members and Partners. Database Resources of the National Genomics Data Center, China National Center for Bioinformation in 2025. Nucleic Acids Res 2024;gkae978. https://doi.org/10.1093/nar/gkae978.
82. Chen T, Chen X, Zhang S, et al. The Genome Sequence Archive Family: Toward Explosive Data Growth and Diverse Data Types. Genomics Proteomics Bioinformatics 2021;19:578-583. https://doi.org/10.1016/j.gpb.2021.08.001.
83. Chen M, Ma Y, Wu S, et al. Genome Warehouse: A Public Repository Housing Genome-scale Data. Genomics Proteomics Bioinformatics 2021;19:584-589. https://doi.org/10.1016/j.gpb.2021.04.001.
84. Liu C; Zhang J; Xu R; Lv J; Qiao Z; Bai M; Zhao S; Luo L; Liu G; Liu P. Supporting data for "A chromosome-scale genome assembly of the pioneer plant *Stylosanthes angustifolia*: insights into genome evolution and drought adaptation" GigaScience Database 2024. https://doi.org/10.5524/102626.

Table 1. Statistics of genomic features of *S. angustifolia*

**Terms**	***S. angustifolia***
Estimated genome size (Mb)	661.55
Assembled genome size (Mb)	645.88
Contig N50 (Mb)	14.99
GC content (%)	35.52
BUSCO (%)	99.26
LTR assembly index	19.49
K-mer completeness (%)	96.37
Number of chromosomes	10
Repeat content (Mb)	319.98
Repeat ratio (%)	49.54
Number of protein-coding genes	36,857
Mean exon length (bp)	242.95
Mean intron length (bp)	465.57

Figure legends

Figure 1. Genomic features of *S. angustifolia*. (a) Features of assembled *S. angustifolia* genome. From 1 to 6: chromosomes, repeat element density, gene density, non-coding RNA density, GC content, and intraspecific collinearity between chromosomes. The contents of 2 to 5 were calculated using a non-overlapping window size of 500 Kb. **(b)** Hi-C interactions among ten chromosomes of the *S. angustifolia* genome. Dark red indicates strong interactions and yellow indicates weak interactions.

Figure 2. Comparative genomic analyses of *S. angustifolia* and other plant species. (a) Orthologous groups (OGs) and shared OGs of studied Fabaceae species and Arabidopsis. The red circle represents *S. angustifolia*-specific OGs. **(b)** Phylogenetic trees and divergence time analysis of the studied species based on single-copy OGs. **(c)** Genomic synteny comparisons between *S. angustifolia*, *A. duranensis*, and *A. ipaensis*. **(d)** Ks distribution of collinear gene pairs within and between *S. angustifolia*, *A. duranensis*, *A. ipaensis*, and *G. max*.

Figure 3. Transcriptome analysis and genome evolution of *S. angustifolia* in adaptation to drought stress. Plant phenotypes **(a)** and leaf characteristics **(b)** of *S. angustifolia* after 0 days (D0), 3 days (D3), and 5 days (D5) of drought treatment. Intersection analysis of genome-expanded genes and differentially expressed genes (DEGs) in roots **(c)** and leaves **(d)** of *S. angustifolia* at D3 compared to D0. Intersection analysis of genome-expanded genes and DEGs in roots **(e)** and leaves **(f)** of *S. angustifolia* at D5 compared to D0. **(g)** KEGG pathway enrichment analysis of genome-expanded genes that are up-regulated by drought stress in roots and leaves of *S. angustifolia* (*Q*-value < 0.05).

Figure 4. **Evolution and expansion of *xanthoxin dehydrogenase* (*ABA2*) genes in the *S. angustifolia* genome and their response to drought stress. (a)** Phylogenetic tree of *ABA2* genes in *S. angustifolia, G. max, M. truncatula,* and *A. thaliana*. The heatmap illustrates the changes in the expression of the *ABA2* genes after drought treatment for 3 days (D3) or 5 days (D5), compared to the control (0 days, D0). Differentially expressed genes (DEGs) are defined as those with a $|\log_2$ fold change$| > 1$ and *Padj* < 0.05. **(b)** Microcollinearity of *ABA2* genes in *S. angustifolia* compared with *G. max* and *M. truncatula*. Red curves represent the correspondence of *ABA2* genes across different species. **(c)** The divergence time of *ABA2* genes occurred by tandem duplication. The quantification of ABA contents in roots **(d)** and leaves (**e**) of *S. angustifolia*. Asterisks indicate significant differences between D5 and D0, as determined by Student's *t*-test: ***$P < 0.001$.

Figure 5. **Evolution and expansion of *2-hydroxyisoflavanone dehydratase* (*HIDH*) genes in the *S. angustifolia* genome and their response to drought stress. (a)** Phylogenetic tree of *HIDH* genes in *S. angustifolia, G. max,* and *M. truncatula*. The heatmap illustrates the changes in the expression of the *HIDH* genes after drought treatment for 3 days (D3) or 5 days (D5), compared to the control (0 days, D0). Differentially expressed genes (DEGs) are defined as those with a $|\log_2$ fold change$| > 1$ and *Padj* < 0.05. **(b)** Microcollinearity of *HIDH* genes in *S. angustifolia* compared with *G. max* and *M. truncatula*. Red curves represent the correspondence of *HIDH* genes across different species. The quantification of genistein contents in roots **(c)** and leaves **(d)** of *S. angustifolia*. The quantification of daidzein contents in roots **(e)** and leaves **(f)** of *S. angustifolia*. Asterisks indicate significant differences between D5 and D0, as determined by Student's *t*-test: * $0.01 \leq P < 0.05$, **$P < 0.01$. NS, not significant.

Figure 6. **Analysis of tandem duplicated genes (TDGs) and the biosynthesis pathway of raffinose and stachyose in *S. angustifolia* under drought stress. (a)** Ks distribution of TDGs in *S. angustifolia*, *A. duranensis*, *A. ipaensis*, *G. max*, and *M. truncatula*. **(b)** KEGG pathway enrichment analysis of TDGs in *S. angustifolia*. The KEGG pathways depicted in the figure are associated with a *Q*-value < 0.05. **(c)** Gene expression changes in the raffinose and stachyose biosynthesis pathway after 3 days (D3) and 5 days (D5) of drought treatment compared to the control (0 days, D0). Differentially expressed genes (DEGs) are defined as those with a $|\log_2$ fold change$| > 1$ and *Padj* < 0.05. **(d)** Microcollinearity of *STS* genes in *S. angustifolia* compared with *G. max* and *M. truncatula*. Red curves represent the correspondence of *STS* genes across different species. The quantification of raffinose contents in roots **(e)** and leaves **(f)** of *S. angustifolia*. The quantification of stachyose contents in roots **(g)** and leaves **(h)** of *S. angustifolia*. Asterisks indicate significant differences between D5 and D0, as determined by Student's *t*-test: * $0.01 \leq P < 0.05$, **$P < 0.01$.

Figure 7. **Lipidomics analysis of roots and leaves of *S. angustifolia* in response to drought stress.** The volcano plots show lipid profiles in roots **(a)** and leaves **(b)** after 5 days (D5) of drought treatment compared to the control (0 days, D0). The heatmap illustrates the differentially accumulated triacylglycerols (TAGs) in roots **(c)** and leaves **(d)** at D5 compared to D0. (**e**) Changes in gene expression of the *oleosin* and *caleosin* families at D3 and D5 compared to D0. Differentially expressed genes (DEGs) are defined as those with a $|\log_2$ fold change$| > 1$ and *Padj* < 0.05. (**f**) Phylogenetic tree of caleosins in *S. angustifolia, G. max, M. truncatula,* and *A. thaliana*.

Figure 8. Differential accumulation of phospholipids, sulfolipids, galactolipids, and glucolipids in leaves (a) and roots (b) of *S. angustifolia* after 5 days (D5) of drought treatment compared to the control (0 days, D0).

Figure 9. Biosynthesis of jasmonic acid (JA) and jasmonoyl-L-isoleucine (JA-Ile) in *S. angustifolia* in response to drought stress. (**a**) Gene expression changes in the JA biosynthesis pathway after 3 days (D3) and 5 days (D5) of drought treatment compared to the control (0 days, D0). Differentially expressed genes (DEGs) are defined as those with a $|\log_2$ fold change$| > 1$ and *Padj* < 0.05. (**b**) Microcollinearity of *pPLA* genes in *S. angustifolia* compared with *G. max* and *M. truncatula*. Red curves represent the correspondence of *pPLA* genes across different species. The quantification of JA contents in roots **(c)** and leaves **(d)** of *S. angustifolia*. The quantification of JA-

Ile contents in roots **(e)** and leaves **(f)** of *S. angustifolia*. Asterisks indicate significant differences between D5 and D0, as determined by Student's *t*-test: ***$P < 0.001$.

Additional Files

Fig. S1. The field phenotype of *S. angustifolia*.

Fig. S2. Genomic survey analysis of *S. angustifolia*.

Fig. S3. Gene family expansion and contraction analyses of seven studied plant species.

Fig. S4. KEGG classification of *S. angustifolia* expanded orthologous groups (OGs).

Fig. S5. Leaf chlorophyll a (**a**), leaf chlorophyll b (**b**), soil water content (**c**), and shoot water content (**d**) of *S. angustifolia* after drought treatment for 0 days (D0), 3 days (D3), and 5 days (D5) under pot conditions.

Fig. S6. Volcano plot of differentially expressed genes (DEGs) in roots and leaves of *S. angustifolia* under drought stress.

Fig. S7. (**a**) Intersection analysis of DEGs between roots and leaves at D3 compared to D0. (**b**) Intersection analysis of DEGs between roots and leaves at D5 compared to D0.

Table S1. Statistics of Oxford Nanopore (ONT) sequencing data of *S. angustifolia*.

Table S2. Statistics of Next-Generation Sequencing (NGS) data of *S. angustifolia*.

Table S3. Statistics of high-through chromosome conformation capture (Hi-C) sequencing data of *S. angustifolia*.

Table S4. BUSCO assessment of assembled genome.

Table S5. Statistics of repetitive sequence in the assembled genome.

Table S6. Classification of repetitive sequence in the assembled genome.

Table S7. Statistics of RNA-seq data from different tissues.

Table S8. BUSCO assessment of predicted gene set.

Table S9. Functional annotation of the predicted genes.

Table S10. Statistics of non-coding RNAs in the assembled genome.

Table S11. Statistics of RNA-seq data of *S. angustifolia* after drought treatment for 0 days (D0), 3 days (D3), and 5 days (D5).

Table S12. KEGG pathway enrichment analysis of the *S. angustifolia* expanded genes that were up-

regulated in roots at D3 compared to D0.

Table S13. KEGG pathway enrichment analysis of the *S. angustifolia* expanded genes that were up-regulated in leaves at D3 compared to D0.

Table S14. KEGG pathway enrichment analysis of the *S. angustifolia* expanded genes that were up-regulated in roots at D5 compared to D0.

Table S15. KEGG pathway enrichment analysis of the *S. angustifolia* expanded genes that were up-regulated in leaves at D5 compared to D0.

Table S16. Identification of *ABA2* (K09841) genes in *S. angustifolia*, soybean, barrel medic, and Arabidopsis based on the KEGG database.

Table S17. Calculation of divergence time of the tandem duplicated *ABA2* genes in the *S. angustifolia* genome.

Table S18. Identification of *HIDH* (K13258) genes in *S. angustifolia*, soybean, and barrel medic based on the KEGG database.

Table S19. Calculation of divergence time of the tandem duplicated *HIDH* genes in the *S. angustifolia* genome.

Table S20. The expression changes of genes involved in the biosynthesis of raffinose and stachyose in *S. angustifolia* at D3 or D5 compared to D0.

Table S21. Lipid profiles in roots of *S. angustifolia* at D5 and D0.

Table S22. Lipid profiles in leaves of *S. angustifolia* at D5 and D0.

Table S23. Identification of oleosin and caleosin encoding genes in *S. angustifolia*, soybean, and barrel medic based on Arabidopsis database.

Table S24. The expression changes of genes involved in the biosynthesis of JA and JA-Ile in *S. angustifolia* at D3 or D5 compared to D0.

Figure 1

Click here to access/download;Figure;Figure 1.pdf

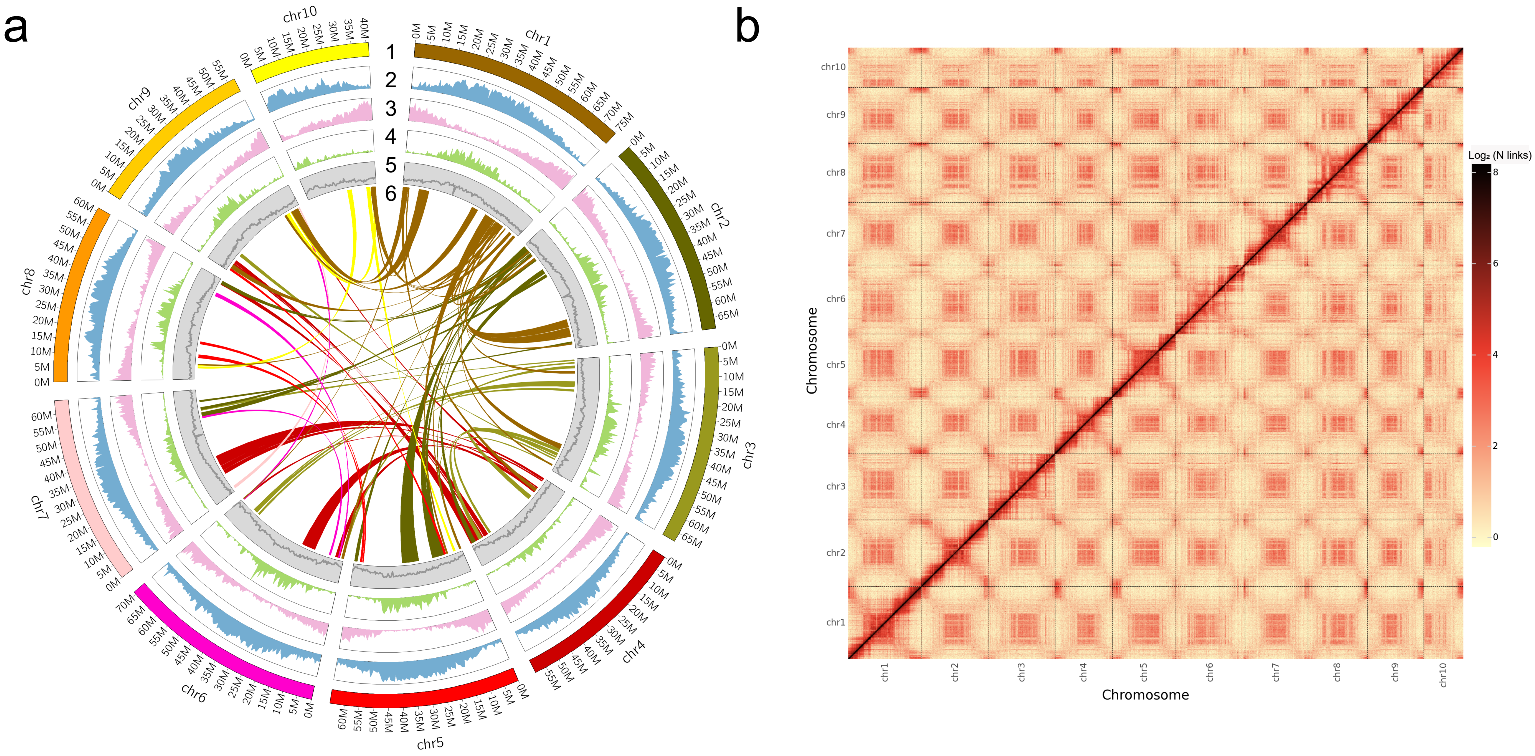

Figure 2

Click here to access/download;Figure;Figure 2.pdf

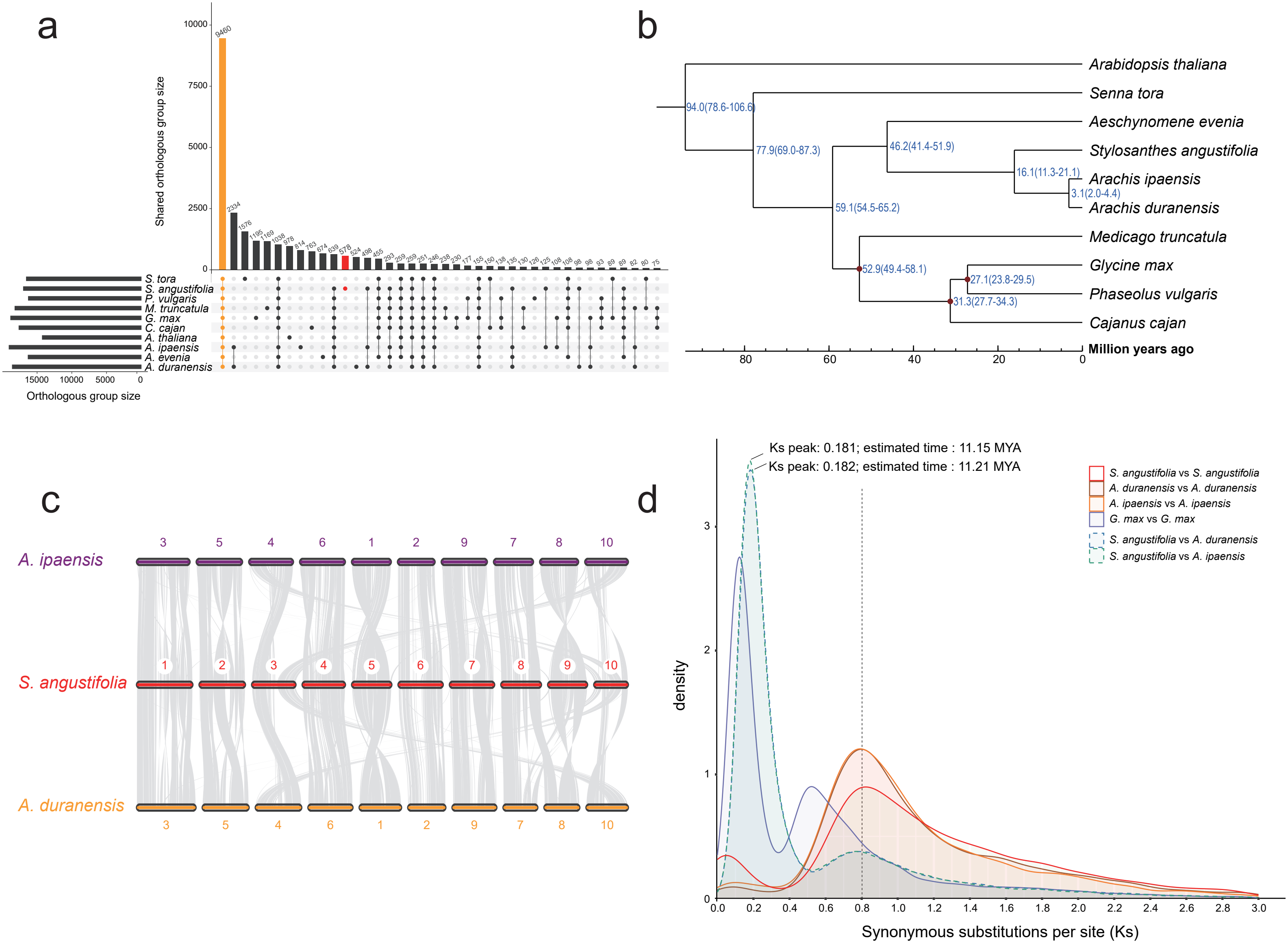

Figure 3

Click here to access/download;Figure;Figure 3.pdf

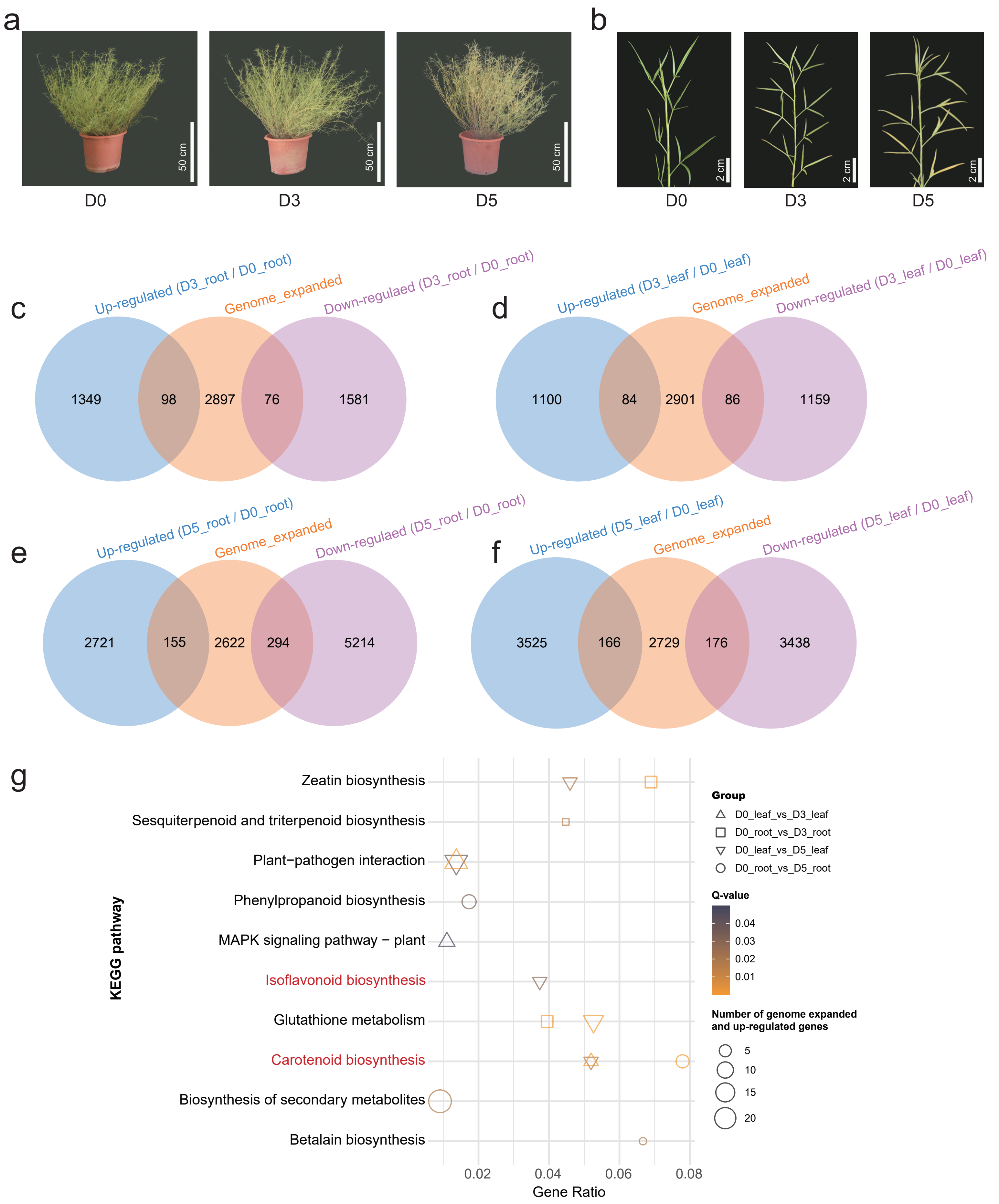

Figure 4 Click here to access/download;Figure;Figure 4.pdf

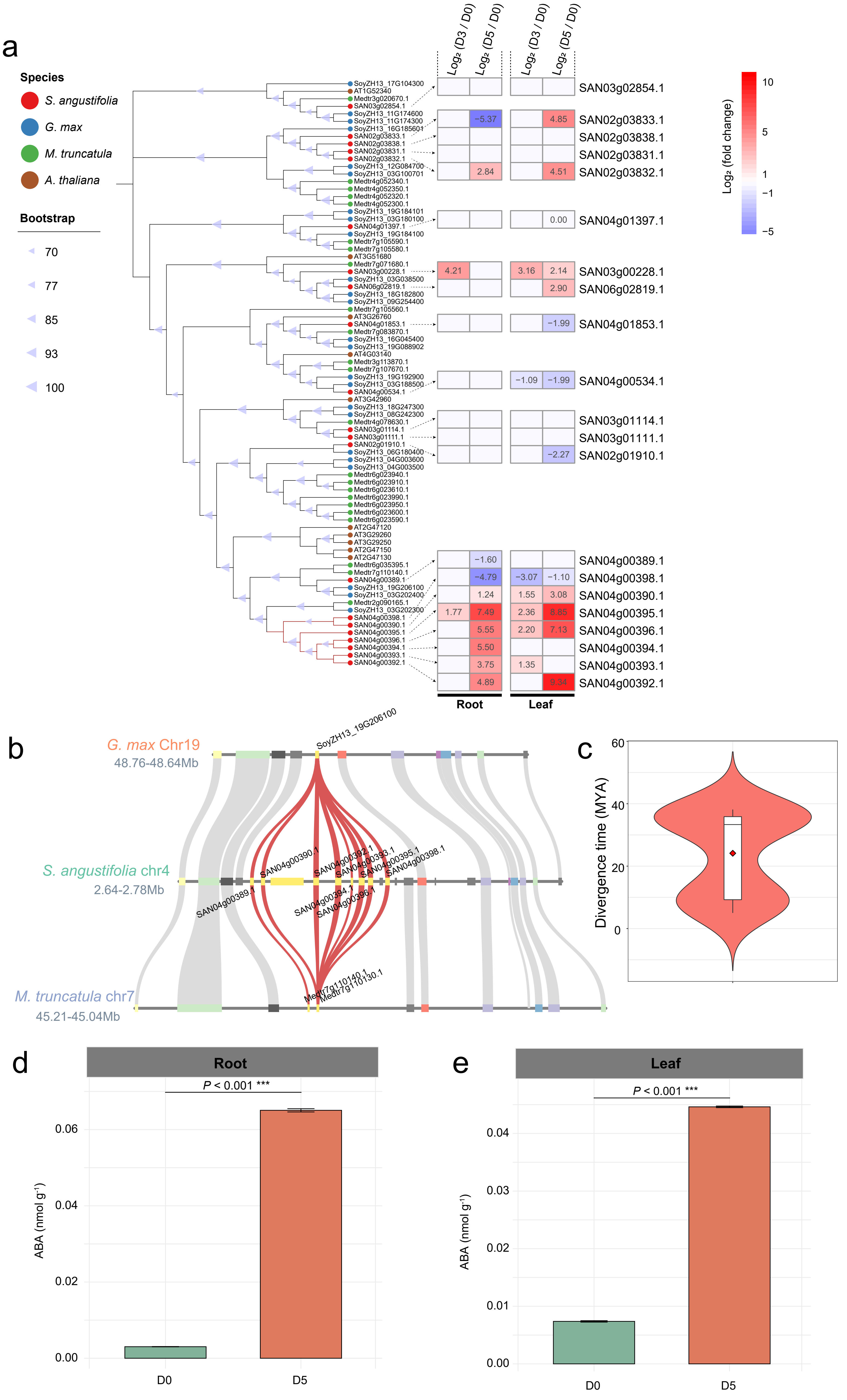

Figure 5

Click here to access/download;Figure;Figure 5.pdf

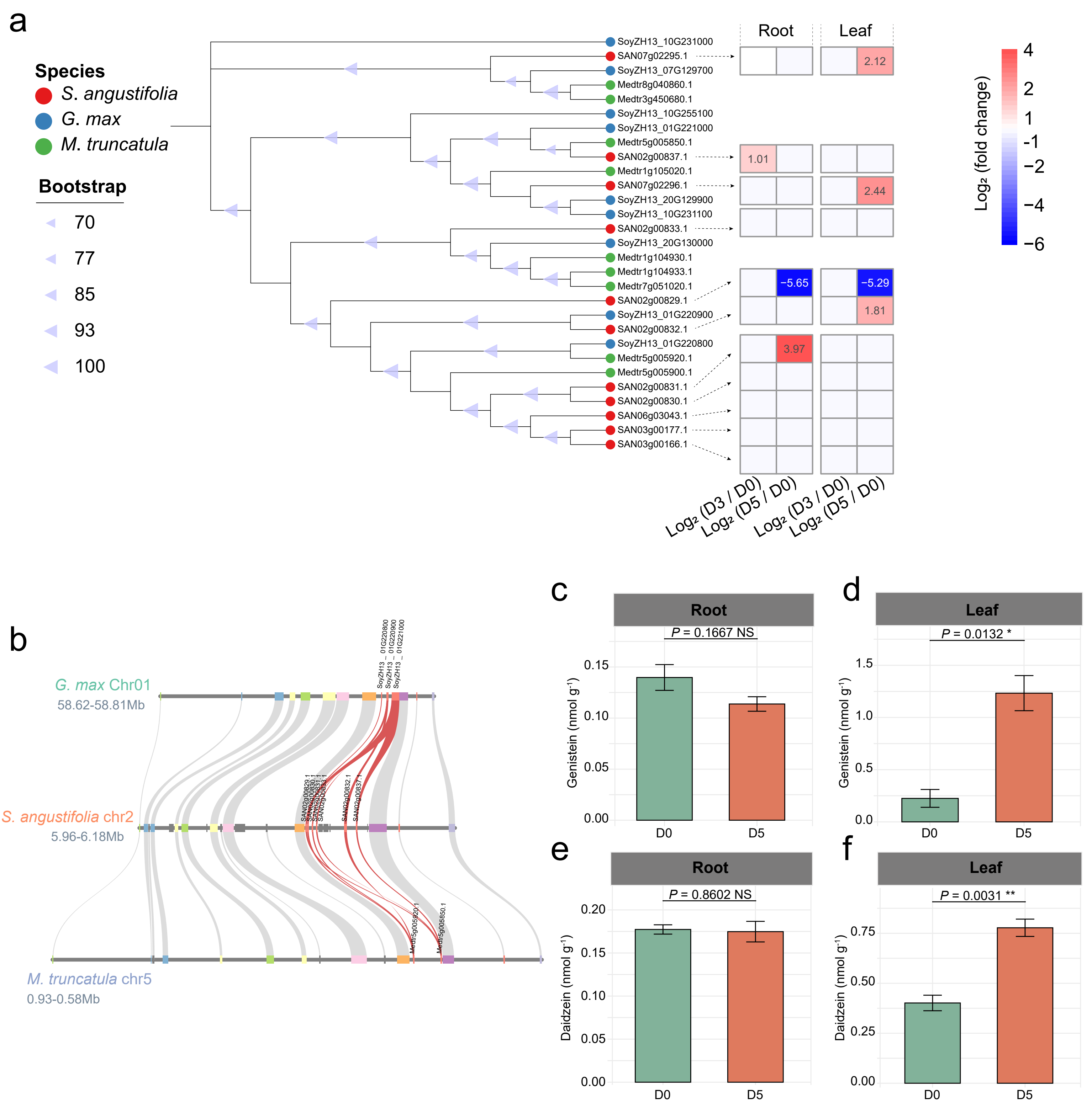

Figure 6 Click here to access/download;Figure;Figure 6.pdf

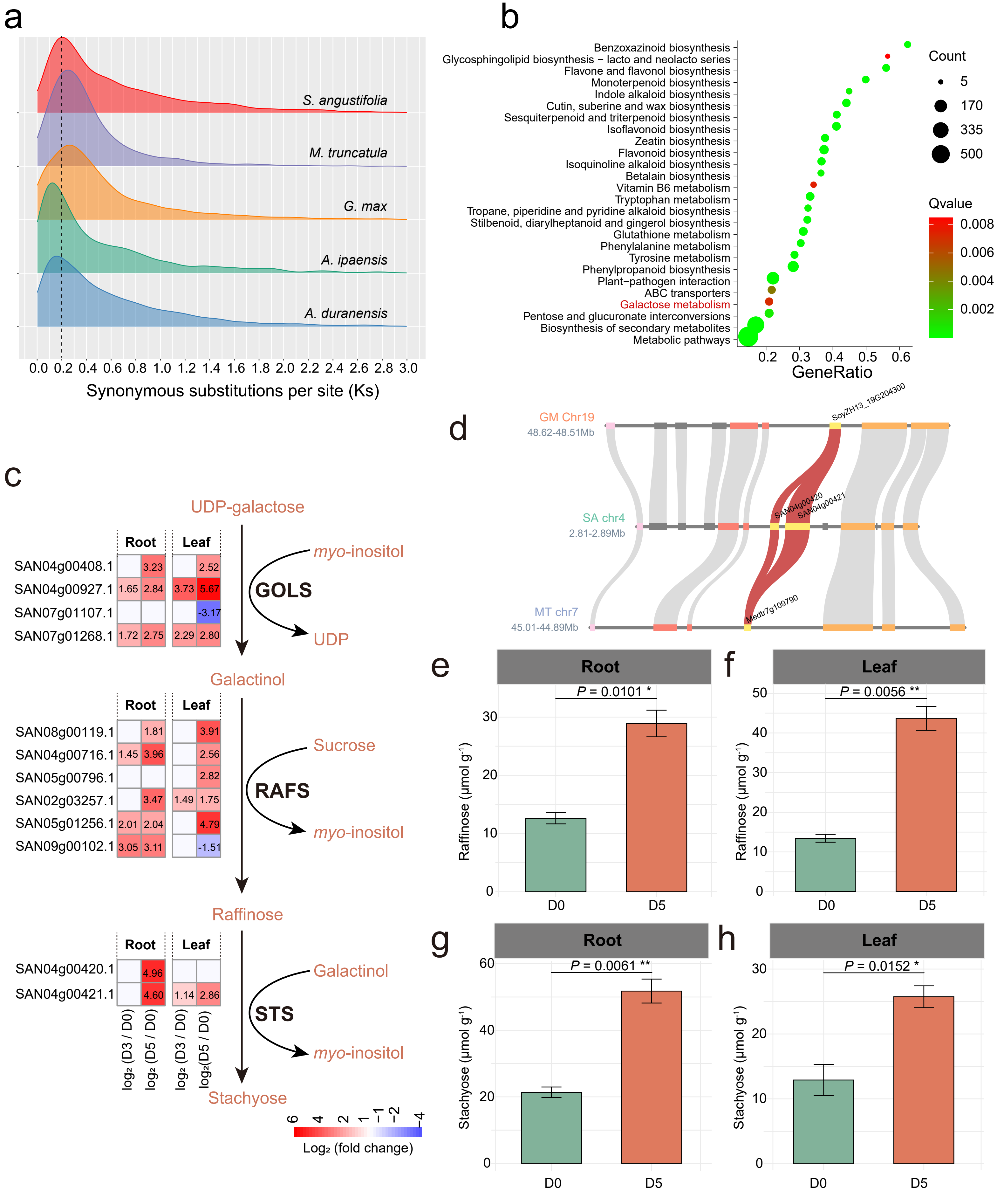

Figure 7

Click here to access/download;Figure;Figure 7.pdf

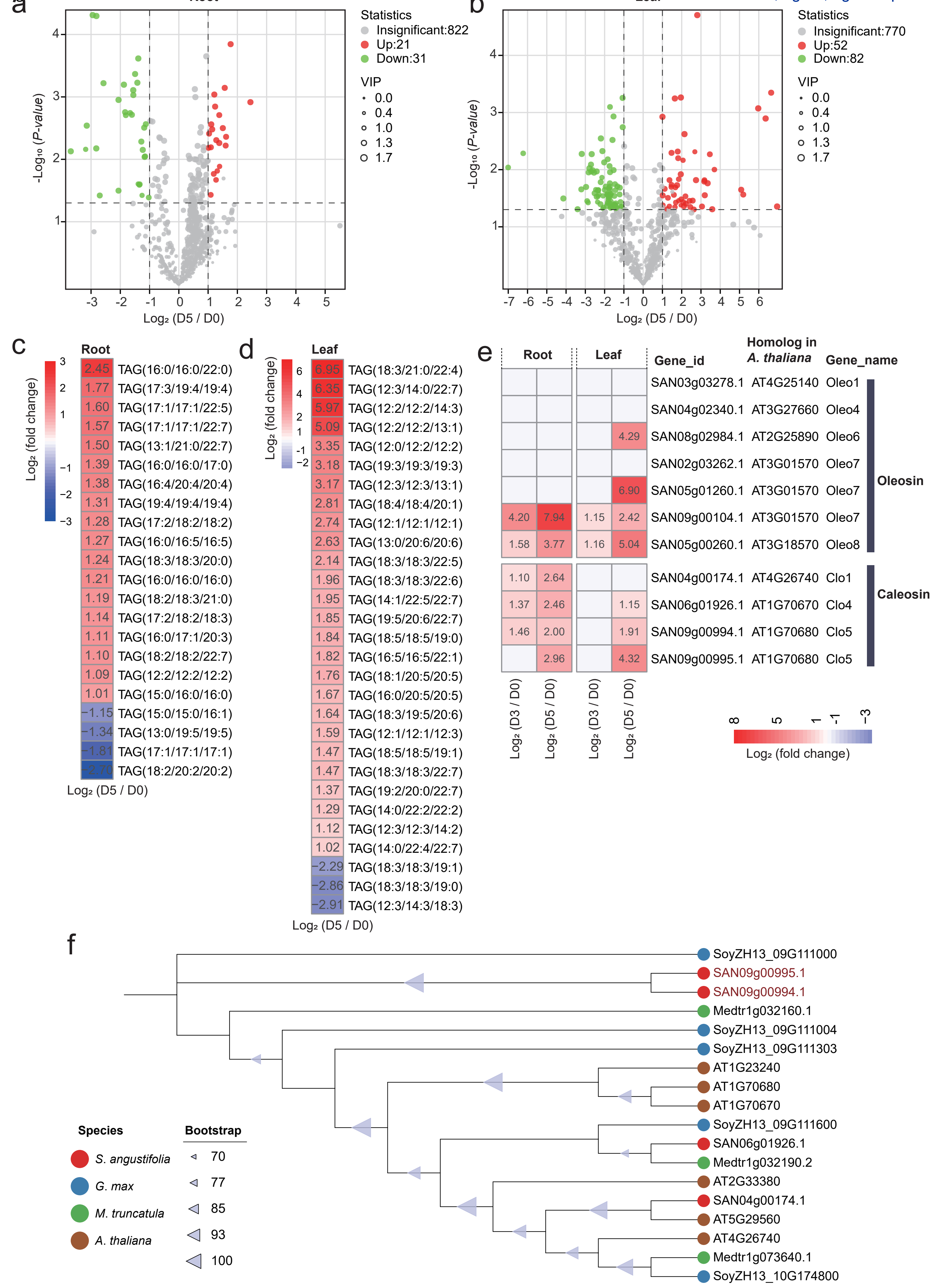

Figure 8

Click here to access/download;Figure;Figure 8.pdf

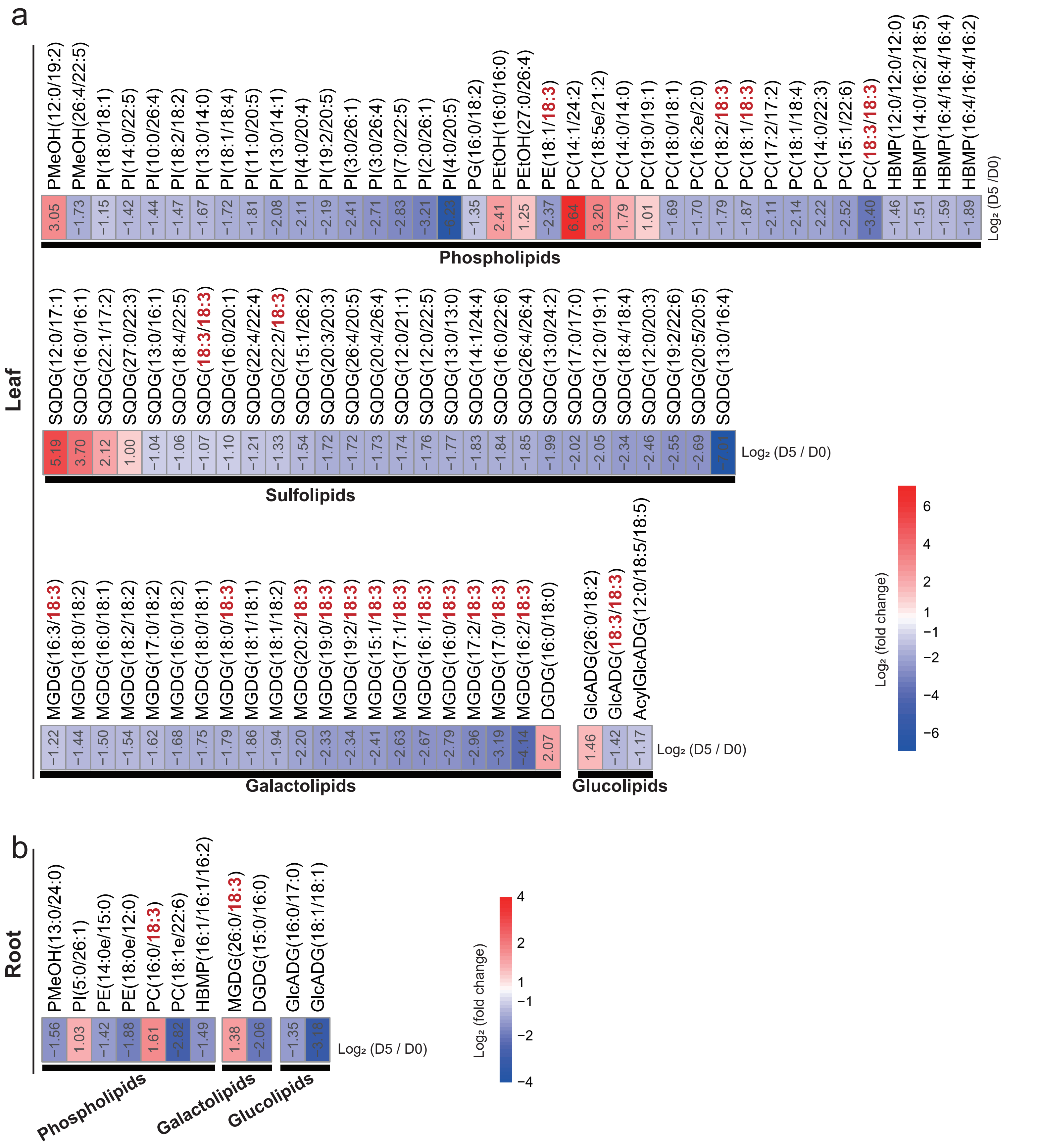

Figure 9

Click here to access/download;Figure;Figure 9.pdf

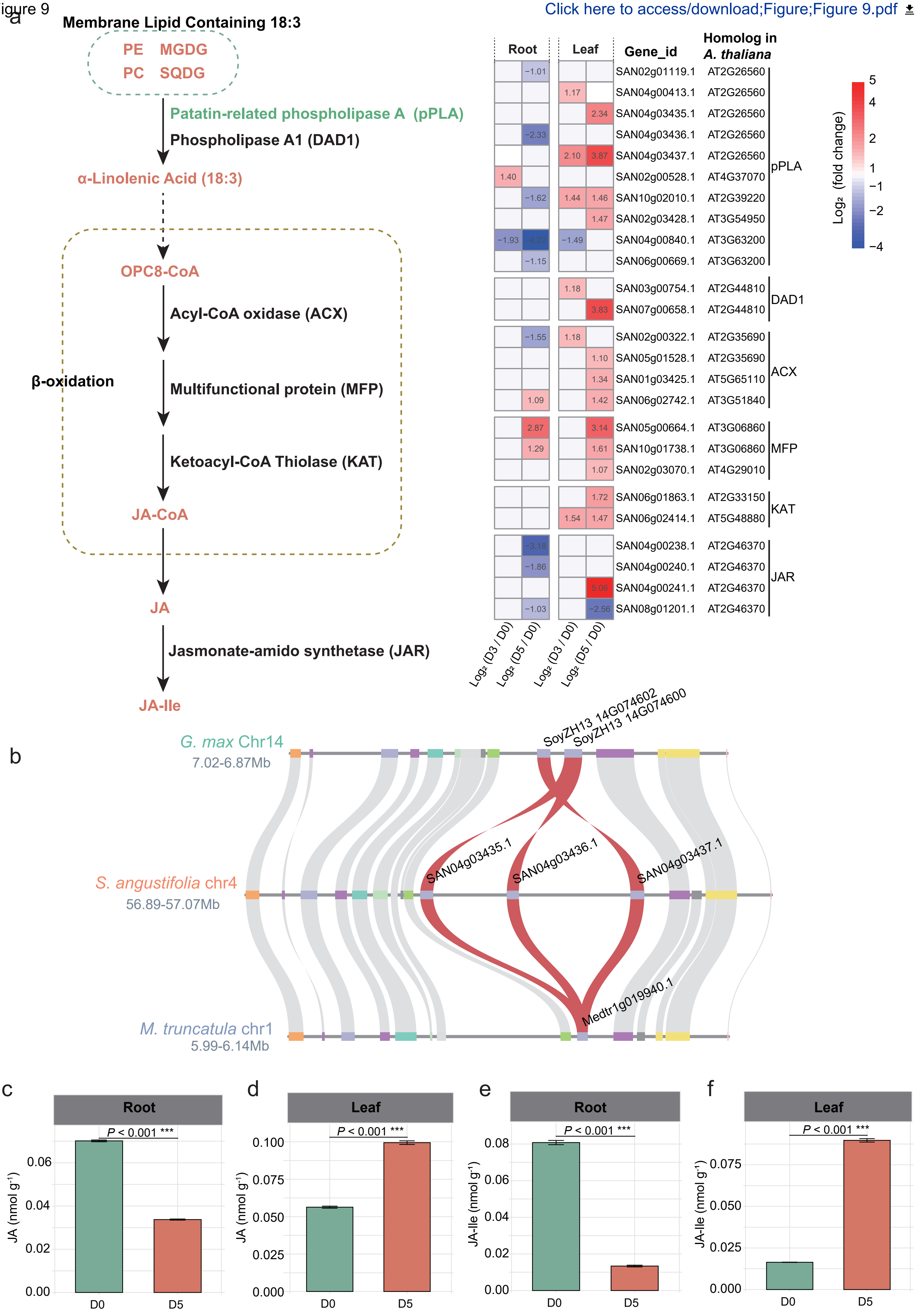

Root D3/D0	Root D5/D0	Leaf D3/D0	Leaf D5/D0	Gene_id	Homolog in A. thaliana	
	−1.01			SAN02g01119.1	AT2G26560	pPLA
		1.17		SAN04g00413.1	AT2G26560	pPLA
			2.34	SAN04g03435.1	AT2G26560	pPLA
	−2.33			SAN04g03436.1	AT2G26560	pPLA
		2.10	3.87	SAN04g03437.1	AT2G26560	pPLA
1.40				SAN02g00528.1	AT4G37070	pPLA
	−1.62	1.44	1.46	SAN10g02010.1	AT2G39220	pPLA
			1.47	SAN02g03428.1	AT3G54950	pPLA
−1.93	−4.22	−1.49		SAN04g00840.1	AT3G63200	pPLA
	−1.15			SAN06g00669.1	AT3G63200	pPLA
		1.18		SAN03g00754.1	AT2G44810	DAD1
			3.83	SAN07g00658.1	AT2G44810	DAD1
	−1.55	1.18		SAN02g00322.1	AT2G35690	ACX
			1.10	SAN05g01528.1	AT2G35690	ACX
			1.34	SAN01g03425.1	AT5G65110	ACX
	1.09		1.42	SAN06g02742.1	AT3G51840	ACX
	2.87		3.14	SAN05g00664.1	AT3G06860	MFP
	1.29		1.61	SAN10g01738.1	AT3G06860	MFP
			1.07	SAN02g03070.1	AT4G29010	MFP
			1.72	SAN06g01863.1	AT2G33150	KAT
		1.54	1.47	SAN06g02414.1	AT5G48880	KAT
	−3.18			SAN04g00238.1	AT2G46370	JAR
	−1.86			SAN04g00240.1	AT2G46370	JAR
			5.06	SAN04g00241.1	AT2G46370	JAR
	−1.03		−2.56	SAN08g01201.1	AT2G46370	JAR

Figure S1

Click here to access/download;Figure;Figure S1.pdf

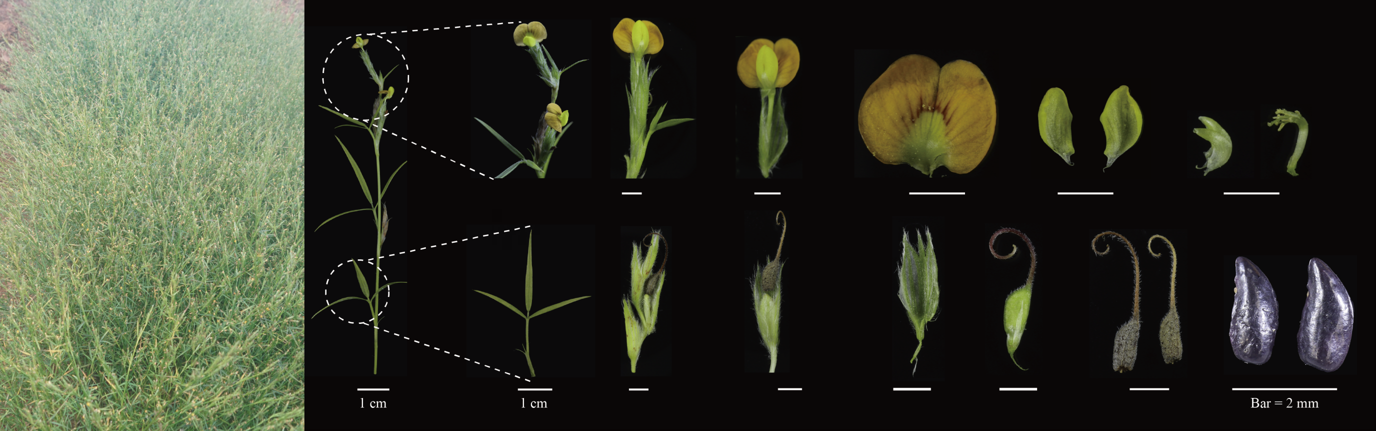

Figure S2

Click here to access/download;Figure;Figure S2.pdf

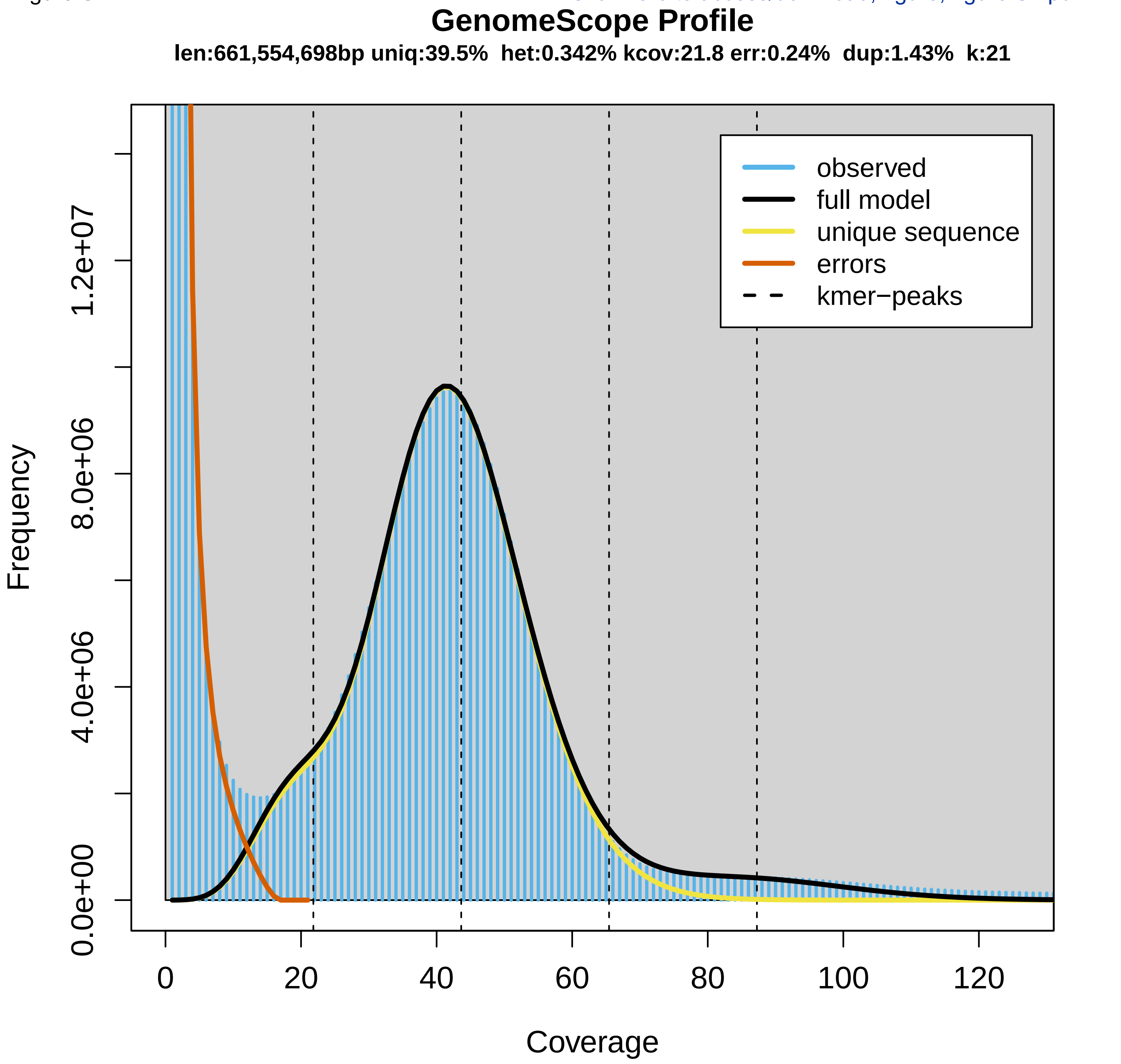

Figure S3

Click here to access/download;Figure;Figure S3.pdf

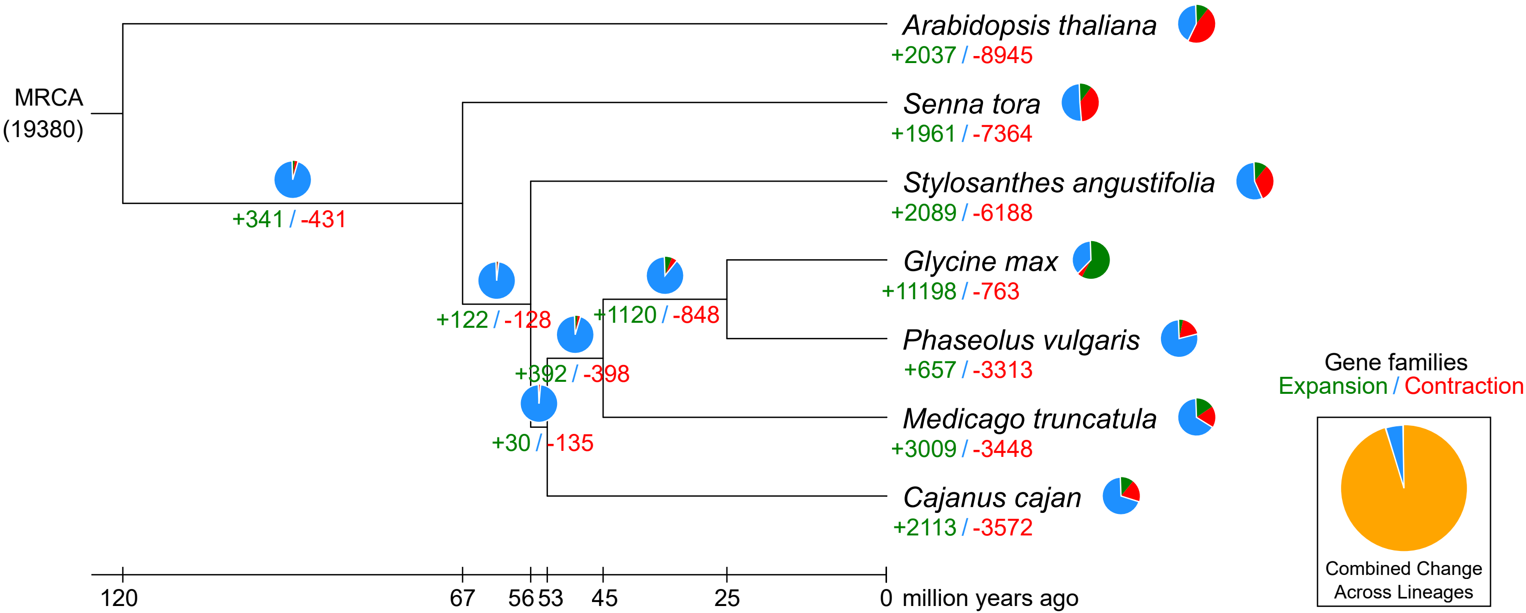

Figure S4

Click here to access/download;Figure;Figure S4.pdf

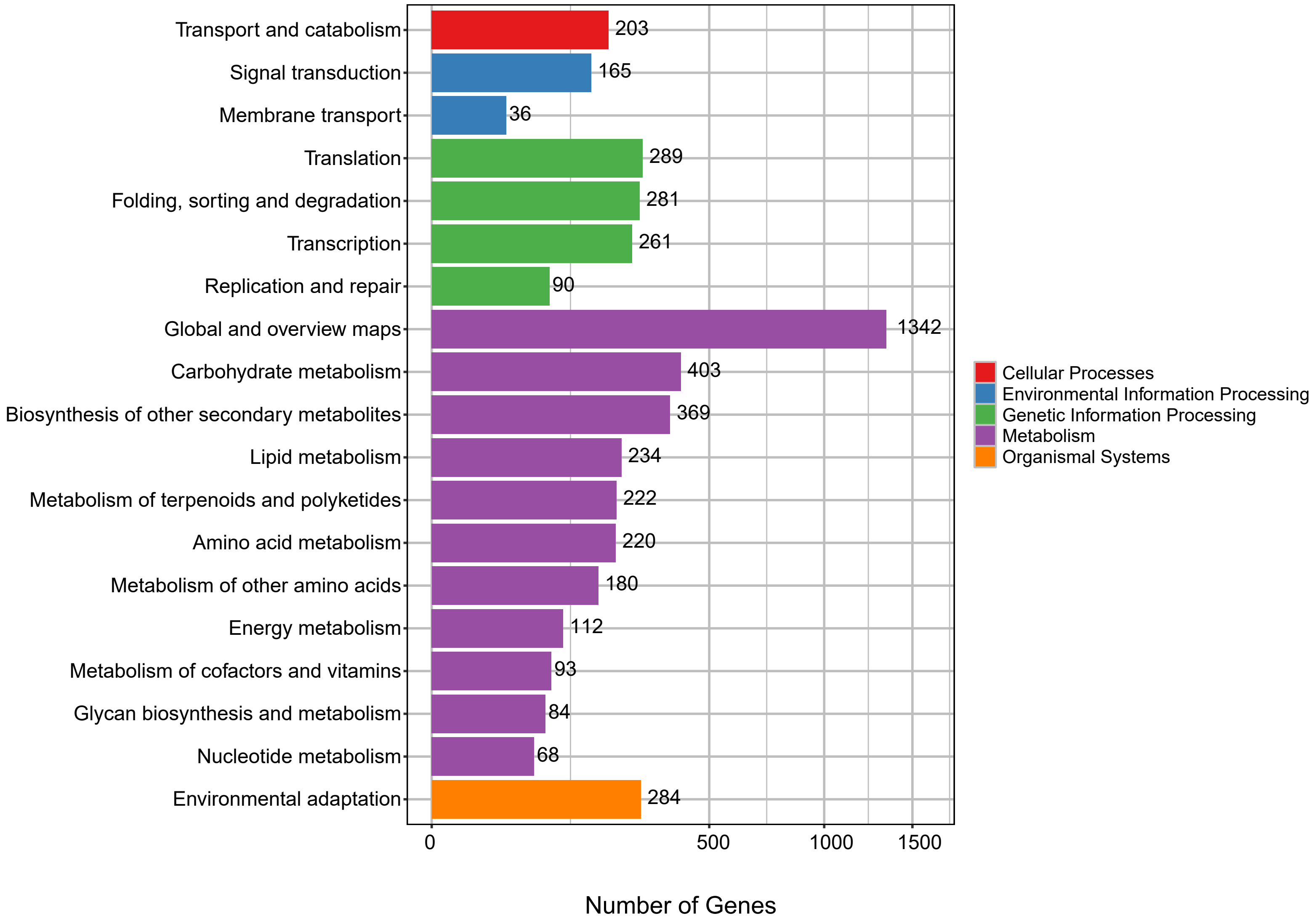

Figure S5

Click here to access/download;Figure;Figure S5.pdf

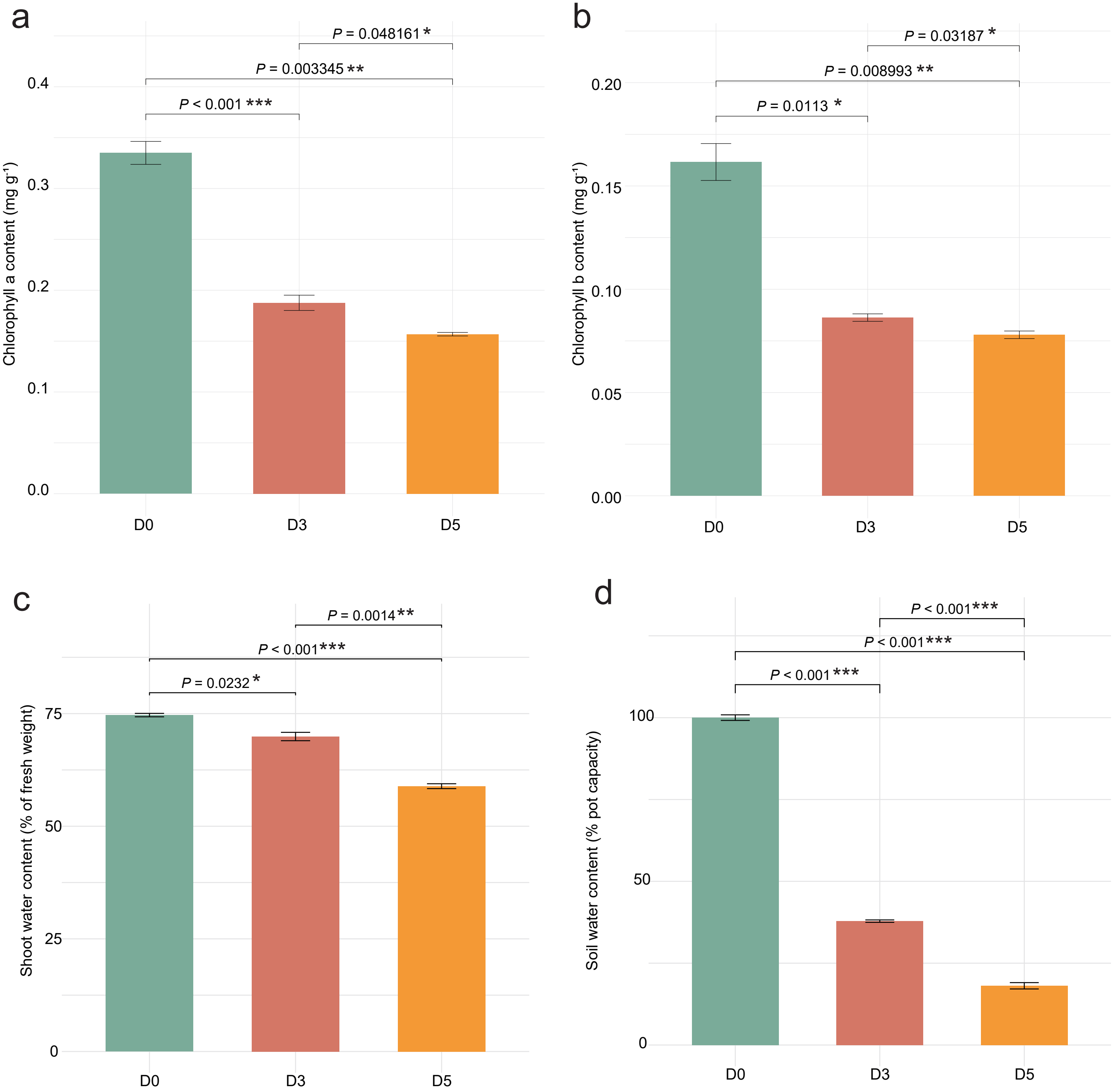

Figure S6

Click here to access/download;Figure;Figure S6.pdf

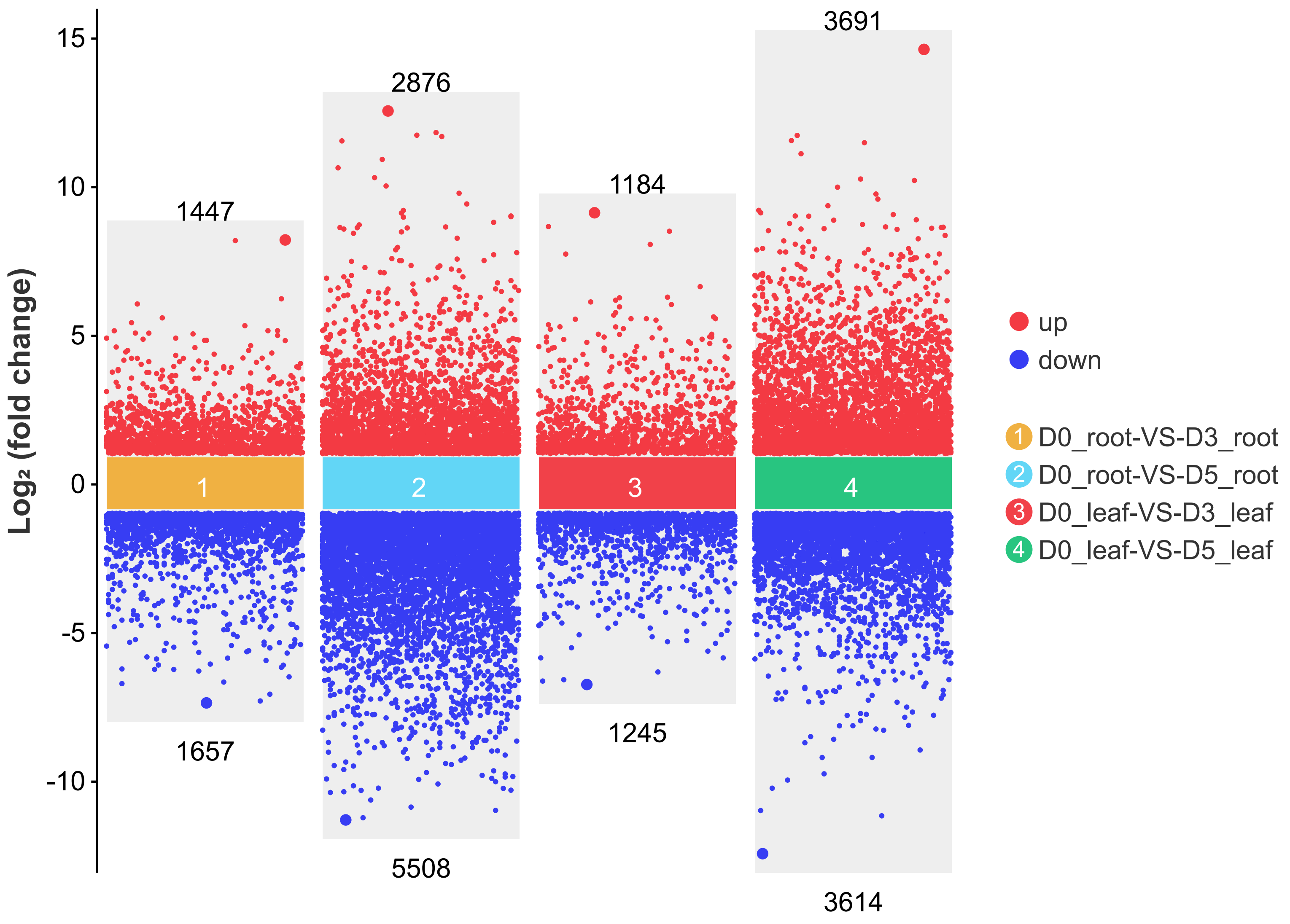

Figure S7

Click here to access/download;Figure;Figure S7.pdf

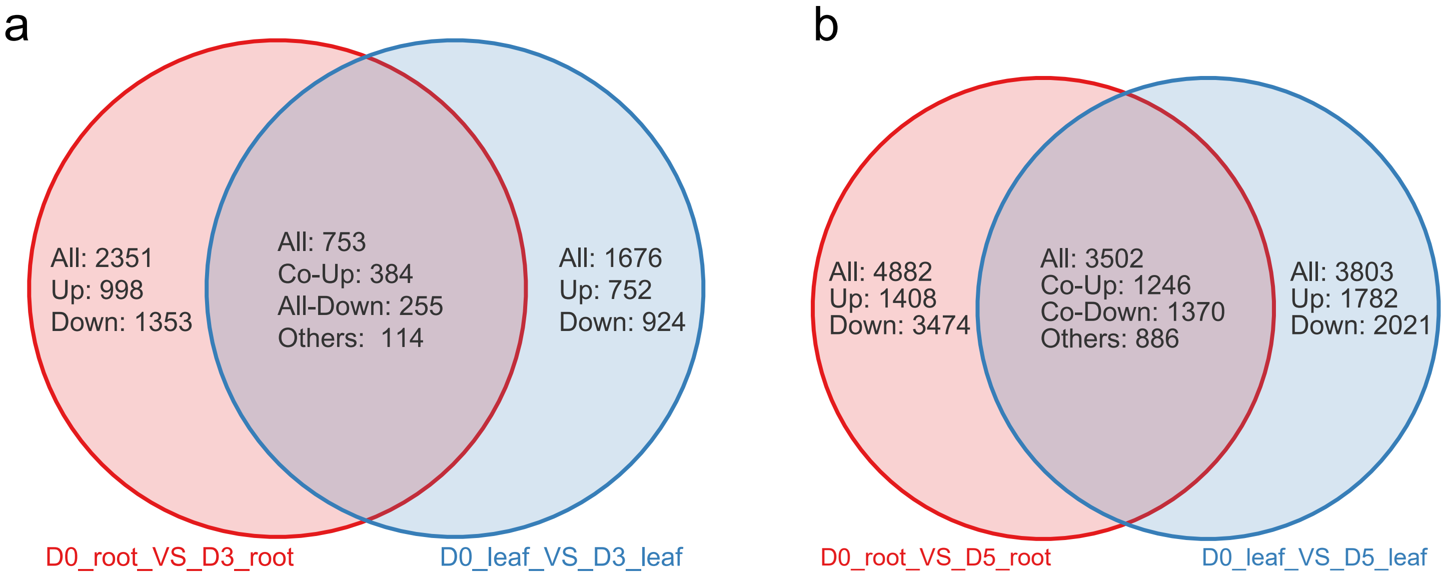

Click here to access/download
Supplementary Material
Supplementary Tables.xlsx
