# Supplementary material for: A chromosome-scale genome assembly of the pioneer plant Stylosanthes angustifolia: insights into genome evolution and drought adaptation
Source: Gigascience. 2025 Jan 24;14:giae118. doi: 10.1093/gigascience/giae118 (PMC11758145; doi:10.1093/gigascience/giae118)
Supplement: giae118_Response_to_Reviewer_Comments_Original_Submission [file giae118_response_to_reviewer_comments_original_submission.pdf]

Author's Response To Reviewer Comments

GIGA-D-24-00294

Dear Reviewers and Editors:

Thank you very much for your valuable feedback and comments. Your suggestions have been instrumental in revising and improving our manuscript. As recommended, we have carefully revised the manuscript and made corrections that we hope will meet your approval. The modified sections are highlighted in red within the manuscript. The main corrections in the paper and the responses to the reviewer’s comments are as follows:

Reviewer #1: This manuscript by Liu et al reports the first telomere-to-telomere (T2T) genome assembly of Stylosanthes angustifolia. Together with previously published Legume and Arabidopsis genomes, they conducted evolutionary analyses. They also conducted transcriptome sequencing and metabolome profiling in S. angustifolia under both drought and control treatments to explore the mechanisms underlying the drought tolerance. Please see below all my comments.
Major concerns:
1. I question whether this genome is truly near-complete with only one gap, as claimed. The authors did not provide detailed information regarding how the gaps were closed. Although the 3rd generation sequencing technologies have advanced substantially, creating a gapless genome remains challenging. Typically, this process requires combining various state-of-the-art sequencing technologies (HiFi, ONT ultra-long reads and Hi-C) and/or different gap-closing strategies. The authors combined ONT (N50: 27 kb, which is not ultra-long), Hi-C and Illumina reads to assemble what they describe as a T2T genome containing only one gap. However, several questions remain: How many contigs were generated by NextDenovo? How many of these represent complete chromosomes? How many were anchored to chromosomes using HI-C data? How many gaps were there before the gap-closing step? Also, the authors need to carefully check the gaps that were closed by LR_Gapcloser, since they did not generate new data but relied solely on existing ONT reads.
REPLY: Thank you for the valuable comments regarding the completeness of our genome assembly and the gap-closing process. We utilized NextDenovo to correct and assemble the 158x ONT sequencing data, resulting in 167 initial contigs (Contig N50: 14.99 Mb and 13 contigs). These initial contigs were further polished using both ONT and NGS reads to generate high-quality contigs. Subsequently, Hi-C reads were used to successfully anchor 106 contigs (totaling 631.54 Mb in length) to 10 chromosomes, which initially contained 96 gaps. To further refine the assembly, we performed two rounds of gap filling using LR_Gapcloser with raw ONT reads, successfully closing 95 of these gaps.
The decision to use raw ONT reads for gap filling was made to avoid the potential discontinuities caused by misprocessing of complex regions during the ONT read correction step in NextDenovo. In addition, we used NGS data to detect SNPs, calculating a heterozygosity rate of 0.06% for this genome based on heterozygous SNPs. We believe that the low heterozygosity of this genome, coupled with the high ONT sequencing depth, enabled us to achieve a near-complete assembly of this genome using only ONT, NGS, and Hi-C data. Moreover, the quality assessment of the genome assembly showed a BUSCO completeness score of 99.26%, LTR assembly index of 19.49, and k-mer completeness of 96.37%, further supporting the high quality and integrity of the assembly.
Additionally, the use of the same sequencing technology for both genome assembly and gap-filling is also adopted by researchers (Nature Communications, 2024, DOI: 10.1038/s41467-024-51158-3; BMC Genomics, 2024, DOI: 10.1186/s12864-024-10711-6). (see lines 130 to 138, lines 400to 423 for details)

2. For the drought experimental design, the authors wrote: the drought treatment group consisted of 60-day-old seedlings that were not watered for 5 days, while the control group consisted of 60-day-old seedlings that were not subjected to the drought treatment. I was wondering whether this is a widely accepted method for drought experiment? The authors did not measure the soil moisture. How can the authors ensure that the plants are truly water-deficit? Also, how often did the authors water the plants for the control group?
REPLY: Thank you for the valuable comments regarding our drought experiment.

Drought Experiment Methodology: There may have been an unclear description in our original manuscript. In fact, we conducted a dynamic drought treatment by stopping watering for the 60-day-old seedlings. Seedlings were sampled by collecting leaves and roots at day 0 (D0) for the control group, and at days 3 (D3) and 5 (D5) after drought for the treatment groups. Due to the most visible phenotypic changes appearing after 5 days of drought treatment, we presented data only at D0 and D5 in the original manuscript. In the revised manuscript, we present data on physiological phenotypes and transcriptome analyses after 3 days of drought treatment (D3). Additionally, we have provided a clearer description of the drought treatment process in the methods section. (see lines 498 to 515). In pot experiments, drought treatment was initiated by ceasing watering, with samples collected on various days of water cessation. This method is commonly used to impose drought stress on different plant species. (Nature Genetics, 2016, DOI:10.1038/ng.3636; Plant and Soil, 2019, DOI: 10.1007/s11104-018-3893-1; Plant Physiology, 2022, DOI: 10.1093/plphys/kiac272; International Journal of Biological Macromolecules, 2023, DOI: 10.1016/j.ijbiomac.2023.126582).

Assessment of Drought Stress. Since the experiment was conducted using potted plants, the soil water content is a more appropriate description of the drought conditions rather than soil moisture (Plant and Soil, 2019, DOI: 10.1007/s11104-018-3893-1). In the revised manuscript, we have included measurements for soil water content, shoot water content, and chlorophyll levels (chlorophyll a and b) on D0, D3, and D5. This ensures a robust assessment of the drought conditions. Soil water content was quantified using a previously reported method (Plant and Soil, 2019, DOI: 10.1007/s11104-018-3893-1; Physiol Plantarum, 2016, DOI:10.1111/ppl.12433; Physiol Plantarum, 2016, DOI: 10.1111/ppl.12346), with soil water content significantly decreasing from 100% pot capacity on D0 to 37.84% by D3, and 18.09% by D5 (P-value < 0.05). Additionally, there was a significant reduction in both shoot water content and chlorophyll levels at D3 and D5 compared to D0 (P-value < 0.05). These measurements confirm that the plants were subjected to drought stress.

Transcriptome Sequencing and Metabolite Analysis: In the drought experiment, we collected root and leaf samples on D0, D3, and D5, and performed transcriptome sequencing on all these samples. In a previous version of the manuscript, we included only transcriptome data from D0 and D5 because the phenotypic differences were most pronounced on D5. However, in this revised version, we have analyzed and compared the transcriptome data for D0, D3, and D5 to provide a more comprehensive understanding of S. angustifolia response to drought stress. Since the phenotypic differences were most evident on D5, metabolite analysis was conducted solely on D0 and D5 to investigate changes in metabolite composition. Notably, the same samples were used for both transcriptome sequencing and metabolite analysis.
(see lines 213 to 227 and lines 498 to 530 for details)

3. Abstract: too much genome assembly statistics were written in abstract.
REPLY: Thank you for your valuable comments. We have revised the abstract to include only the genomic assessment results from BUSCO and LAI. (see Abstract for details)

4. The authors may consider extensive revision for the introduction. They used lengthy text to describe the sequencing technologies that can generate T2T genomes. However, only little was introduced for the species S. angusifolia. My reading-through the introduction did not find whether this is a diploid or polyploid. Are there any genomic studies have been published on it before? Why S. angusifolia is important in the genus Stylosanthes and why its genome needs to be sequenced?
REPLY: Thank you for your valuable comments. We have substantially revised the introduction section to better emphasize the significance of Stylosanthes angustifolia and the genus Stylosanthes. We clarified that S. angustifolia is a diploid species (2n = 2x = 20). Furthermore, we have reduced the content concerning telomere-to-telomere (T2T) sequencing to maintain focus on the biological aspects of the species.
Stylosanthes guianensis (2n = 2x = 20), the most widely domesticated and utilized species in the genus Stylosanthes, serves as a significant forage and green manure crop. Numerous cultivars have been developed for agricultural use in different countries. The utilization of wild relatives in genomic and genetic research is particularly valuable, as these species provide unique genetic diversity and traits that are essential for crop improvement. Our research targets S. angustifolia, a wild relative of S. guianensis, which exhibits remarkable adaptability in the dry-hot valley regions of southwestern China, where seasonal drought is common.
Moreover, we are collaborating with international partners to investigate the genome of S. guianensis (unpublished), which makes sequencing S. angustifolia and elucidating its drought resistance mechanisms even more crucial. This study lays a foundation for the improvement of S. guianensis and offers valuable

insights for breeding drought-tolerant cultivars within the genus. (see lines 79 to 105 for details)

5. Did the authors use the same samples (tissues) for both mRNA-Seq sequencing and the lipidomics, ABA, JA and JA-Ile, genistein, daidzein, and raffinose determination?
REPLY: Thank you for your valuable comments. Yes, in our study, the same samples were utilized for both transcriptome sequencing and metabolite analysis. (see line 513 and 515 for details)

6. Table S4: The meaning of "Chr window" is unclear? In chr4, the "TTTAGGG" was located ~8Mb. Is this sequence truly a telomere, which typically occurs at chromosome ends?
REPLY: Thank you for raising this important question regarding the "TTTAGGG" sequence located at approximately 8 Mb on chromosome 4. While this sequence consists of 43 repeats, it is insufficient to classify this region as a functional telomere, which is defined by its specific location at chromosome ends and its association with telomere-binding proteins. The observed sequence is more likely an interstitial telomeric sequence (ITS), a common feature in plant genomes such as Arabidopsis thaliana, often arising from historical chromosomal rearrangements or duplications. These internal repeats do not serve the protective roles of functional telomeres but are remnants of past genomic events (Pl Syst Evol, 1995, doi.org/10.1007/BF00982962; Plants, 2021, doi.org/10.3390/plants10112541). Additionally, as noted in the discussion, further validation is necessary to definitively determine whether this is a true telomere or an ITS, and comparative genomics of related species could offer additional insights. (see lines 143 to 146, lines 328 to 333 for details)
We changed "Chr window" to the chromosome start and end positions in this revision. (see Table S4 for details)

7. Lines 159-163: The authors conclude that the length of the centromeres differ significantly, ranging from 141 kb to 25.6 Mb. This is a main result shown in Table 2. More evidences are needed to support this conclusion, not solely based on the distribution of tandem repeats and LTRs.
REPLY: Thank you for your valuable comments. In the revised version, we used CentIER (Plant Communications, 2024, DOI: 10.1016/j.xplc.2024.101046), a recently published tool, for centromere identification. Unlike traditional methods that rely solely on detecting tandem repeat sequences, CentIER was designed to account for various sequence features, including tandem repeats, retrotransposons, and k-mer frequency distribution. We combined the software’s results with the k-mer distribution plots of the chromosomes to accurately define the centromeric regions. (see lines 156 to 161 and lines 458 to 463 for details)

Minor concerns:
1. Please ensure all mentions of significant, significantly, and significance refer to statistically tested significance. The level of significance (p-value) and name of the statistical test should be provided, either in the text or in the appropriate table/figure/dataset. For mentions not supported by statistical analyses, please choose another word, such as substantial, substantially, importance, etc.
REPLY: Thank you for regarding the terminology related to significance. We have carefully revised the manuscript to ensure that all mentions of "significant," "significantly," and "significance" explicitly refer to statistically tested significance, including the relevant p-values and names of statistical tests. For instances not supported by statistical analyses, we have replaced these terms with alternatives.

2. It is not clear whether the genome of S. angusifolia is highly heterozygous or not. It seems that the authors performed genome survey analysis (shown in Figure S2). It is never mentioned in the main text.
REPLY: Thank you for your valuable comments. We performed a genome survey analysis using k-mer analysis based on second-generation sequencing (NGS) data, which estimated the genome size to be 661.55 Mb. To assess heterozygosity, we aligned the NGS data to the assembled genome using BWA and identified variants with GATK. Our analysis detected 379,931 heterozygous SNPs, corresponding to a heterozygosity rate of 0.06% for S. angustifolia, indicating that the genome is not highly heterozygous. (see lines 140 to 142 and Figure S2 for details)

3. Line 412: LTR_Retriever is actually implemented in the EDTA tool.
REPLY: Thank you for your notification. We have addressed this issue in the revised version.

4. Line 444: LTR_Retriever is the tool for LTR retrotransposons identification. TEs generally refer to all

types of repeats. Please make it clear.
REPLY: Thank you for your valuable comments. We have addressed this issue in the revised version. (see 427 to 428 for details)

5. Line 201: Is there a particular reason why the other species were excluded for the expansion/contraction analysis?
REPLY: Thank you for your valuable comments. The exclusion of certain species (e.g., Aeschynomene evenia, Arachis ipaensis, and Arachis duranensis) from the expansion/contraction analysis was based on several factors. First, we aimed to focus on species that are more phylogenetically relevant to S. angustifolia, along with well-annotated and widely studied legumes (e.g., Glycine max, Phaseolus vulgaris, and Cajanus cajan) to obtain robust results. These species better represent evolutionary trends within the legume family, which is our primary focus. Additionally, Arabidopsis thaliana was included as an outgroup. We excluded other species due to considerations related to the completeness of their genome annotations and the potential complexity they could introduce into the analysis. By concentrating on a subset of legumes with higher-quality data, we aimed to ensure that the results remain interpretable and computationally feasible. We believe this focused selection allows us to more accurately reflect gene family dynamics relevant to our study objectives. (see lines 195 to 198 for details)

6. Lines 206-208: Not "some". I see extensive genomic rearrangements among these species.
REPLY: We have revised the description as follows: The genomes of S. angustifolia, A. duranensis, and A. ipaensis show extensive genomic rearrangements, particularly on chromosomes chr3, chr9, and chr10. However, certain regions of the genomes still maintain strong synteny, such as chromosomes chr1, chr6, and chr8. (see lines 202 to 204 for details)

7. Line 226: how many up-regulated genes overlap between roots and leaves?
REPLY: Thank you for your valuable comments. After 3 days of drought treatment, a total of 384 genes were upregulated in both roots and leaves. After 5 days of drought treatment, this number increased to 1,246 genes. (see Figure S8 and lines 225 to 227 for details)

8. Lines 234-235: Not clear. Do you mean that there are seven ABA2 genes that independently underwent tandem duplication, resulting in many tandem duplicated genes? Or are there a total of seven tandem duplicated ABA2 genes?
REPLY: Thank you for your valuable comments. There was a total of seven tandem duplicated ABA2 genes on chromosome 4 of S. angustifolia. (see Figure 4b and line 238 to 239 for details)

9. Why were lipidomics analysis, raffinose, genistein and daidzein determination performed only in leaves but not in roots?
REPLY: Thank you for your valuable comments. In the previous version of the manuscript, we presented data on these metabolites exclusively for leaf samples on D0 and D5, as we observed that the genes responsible for synthesizing these metabolites were more prominently upregulated in the leaves. In this revised version, metabolite analysis, including lipidomics analysis, as well as the quantification of raffinose, stachyose, genistein, and daidzein, was conducted on root and leaf samples collected on D0 and D5 following drought treatment. We present the accumulation of these metabolites in both root and leaf tissues to facilitate a more comprehensive integrated analysis with the transcriptome data. (see lines 258 to 314 and lines 532 to 602 for details)

Reviewer #2: In the manuscript entitled "The telomere-to-telomere (T2T) genome of the pioneer plant Stylosanthes angustifolia provides insight into its genome evolution and drought adaptation mechanisms", the authors present a T2T genome sequence of Stylosanthes angustifolia, a significant member of the legume family. They offer detailed analyses of the genome's features, including whole genome duplication (WGD), synteny, and gene expansions resulting from duplications. Given that the sequenced species exhibits strong drought resistance, the authors explore the underlying mechanisms using transcriptomic analysis. Interestingly, they attempt to establish a causal relationship between elevated gene expressions and gene duplication. In my opinion, the reported genome and the accompanying analyses provide valuable and intriguing data resources for researchers focusing on legumes. While the manuscript has its merits, several issues need to be addressed before publication.
1. Please carefully make the writings more readable. For example, the authors used several "first" to

emphasis the novelty of the genome, which are unusual expressions.
REPLY: Thank you for your valuable feedback. Upon reviewing the manuscript, we agree that the repeated use of "first" to highlight the novelty of the genome may detract from the clarity of the text. To address this, we have thoroughly revised the entire manuscript to improve readability and avoid redundancy.
2. It had better remove "mechanisms" from the title.
REPLY: Thank you for your valuable comments. In the revised manuscript, we changed the title to "The telomere-to-telomere (T2T) genome of the pioneer plant Stylosanthes angustifolia: insights into genome evolution and drought adaptation".

3. As the authors reported the estimated genome size and the assembled genome size, we see some discordance between the two, could the authors give some explanations?
REPLY: Thank you for pointing out the discrepancy between the estimated and assembled genome sizes. In the manuscript, we initially estimated the genome size of S. angustifolia to be 661.55 Mb using k-mer analysis. However, the final assembled genome size was 631.17 Mb, resulting in a difference of approximately 4.6%. This discordance can be attributed to several factors:
Repetitive Regions and Heterozygosity: The k-mer analysis used for the initial genome size estimation may overestimate genome size due to the inclusion of repetitive sequences and heterozygous regions, which can lead to inflation in the estimated size. During the assembly process, some of these repetitive regions may collapse or be filtered out, leading to a smaller assembled genome size. Residual Gaps and Unresolved Regions: Despite achieving a near-complete telomere-to-telomere (T2T) assembly, a gaps and unresolved regions remain, which could also contribute to the slight reduction in genome size compared to the estimated value. This slight discordance between the estimated and assembled genome sizes is a common observation in T2T plant genomes. For instance, similar discrepancies were reported in the assembly of the soybean (Glycine max) and maize (Zea mays) genomes. In the soybean genome assembly, the estimated genome size was 1.115 Gb (Williams 82) (Nature, 2010, DOI: 10.1038/nature08670), but the final T2T genome size was 1.011 Gb (Williams 82) (Molecular Plant, 2023, DOI: 10.1016/j.molp.2023.08.012), with a difference of 9.33%. Likewise, in the maize T2T assembly, the estimated genome size was 2.3 Gb, while the final assembled size was 2.178 Gb (nature genetics, 2023, DOI: 10.1038/s41588-023-01419-6), with a 5.2% difference. These examples illustrate that minor reductions in size from the estimated genome are a common occurrence in high-quality genome assemblies.

4. In the evolutionary tree, the authors used Arabidopsis as root, why not consider grape?
REPLY: We chose Arabidopsis thaliana as the outgroup for the phylogenetic tree based on its well-established role as a model organism, with a fully sequenced and highly annotated genome. Arabidopsis has been widely used in phylogenetic analyses across various plant families, including legumes, due to its comprehensive genomic resources and its position as a representative of the Brassicales, a well-known eudicot clade. While Vitis vinifera is also a eudicot, Arabidopsis is a more commonly accepted outgroup in legume-related studies. Its inclusion allows us to maintain consistency with previous gene family and divergence analyses, ensuring reliable comparative results. Additionally, the high level of functional annotation available for Arabidopsis provides a strong reference for identifying gene family expansions and contractions in legumes. We believe that using Arabidopsis thaliana as the outgroup provides the most appropriate framework for our study and helps ensure the accuracy and interpretability of our evolutionary analyses. (see 187 to 188 for details)
