# Supplementary material for: A chromosome-scale genome assembly of the pioneer plant Stylosanthes angustifolia: insights into genome evolution and drought adaptation
Source: Gigascience. 2025 Jan 24;14:giae118. doi: 10.1093/gigascience/giae118 (PMC11758145; doi:10.1093/gigascience/giae118)
Supplement: giae118_Response_to_Reviewer_Comments_Revision_1 [file giae118_response_to_reviewer_comments_revision_1.pdf]

Author's Response To Reviewer Comments

GIGA-D-24-00294R2

Dear Reviewers and Editors:

Thank you very much for your valuable feedback and comments. Your suggestions have been instrumental in revising and improving our manuscript. As recommended, we have carefully revised the manuscript and made corrections that we hope will meet your approval. The modified sections are highlighted in red within the manuscript. The main corrections in the paper and the responses to the reviewer's comments are as follows:

Reviewer reports:
Reviewer #1: The authors have addressed some of my concerns. I still have a few comments/questions/suggestions.
1. I acknowledge that the genome assembly present in this study can be high quality given the utilization of advanced sequencing technologies. However, I'm still not convinced that it qualifies as T2T or near-complete. Although the authours have provided some details regarding how the gaps were closed, they did not yet carefully check the gaps that were closed by LR_Gapcloser. My concern is that the authors did not generate new data, relying instead on the same ONT reads (N50 27kb, not ultra-long) used in the initial genome assembly to close these gaps. Almost 98.96% (95/96) gaps can be closed by this tool with the ONT reads. If these reads can bridge the gaps, it raises the question of why they were not incorporated into single contigs during the assembly process? Furthermore, telomeric sequences remain unassembled at one end of chromosomes 4, 7, and 10.
REPLY: Thank you for your valuable feedback on our manuscript. After careful consideration of your comments, we have decided to follow your suggestion and no longer describe the genome as a T2T assembly. We acknowledge your concerns regarding the use of LR_Gapcloser and the reliance on the same ONT reads (N50 27kb) for gap closure, which may not meet the rigorous standards required for a true T2T genome. Consequently, we have removed the gap-closing steps performed with LR_Gapcloser, retaining the 96 gaps in the genome, and reclassified the genome as a high-quality, chromosome-level assembly. Additionally, we have removed the sections related to telomeres and centromeres, which were previously associated with the T2T assembly. Furthermore, we have thoroughly reviewed the genomic data and corrected any inconsistencies. The manuscript, including the title, has also been revised to accurately reflect this updated description of the genome.

2. The authors have presented centromere identification as a standalone subsection in the Results. IMO, this result does not hold significant importance in this manuscript. This is partly because the centromeres are not studied in detail and also due to the instability of the identified centromeric regions between the two manuscript versions (ranging from 2.00 to 6.33 Mb in the revised version, compared to 141 kb to 25.6 Mb in the original manuscript).
REPLY: Thank you for your constructive feedback on our manuscript. We have carefully considered your comments regarding the centromere identification section. After revisiting the overall focus of the manuscript, we have decided to remove the centromere analysis from the revised version. As we no longer describe the genome as a T2T assembly, the detailed study of centromeres, which was not a primary focus of the manuscript, has been excluded.

3. Overall, the manuscript's language should be further improved for clarity and readability.
REPLY: Thank you for your suggestion to improve the clarity and readability of our manuscript. We fully understand the importance of precise language in effectively conveying our research findings. To address this, we have made comprehensive revisions throughout the manuscript and engaged a native English-speaking expert to carefully review and refine the language.
We have worked diligently to enhance the clarity and readability of the text, ensuring that the manuscript meets high standards of linguistic quality. We sincerely appreciate your feedback, as it has been invaluable in helping us improve the overall presentation of our work.
