# Supplementary material for: A chromosome-scale genome assembly of the pioneer plant Stylosanthes angustifolia: insights into genome evolution and drought adaptation
Source: Gigascience. 2025 Jan 24;14:giae118. doi: 10.1093/gigascience/giae118 (PMC11758145; doi:10.1093/gigascience/giae118)
Supplement: giae118_Reviewer_1_Report_Original_Submission — Feng Cheng -- 8/16/2024 [file giae118_reviewer_1_report_original_submission.pdf]

Reviewer Report

Title: A chromosome-scale genome assembly of the pioneer plant Stylosanthes angustifolia: insights into genome evolution and drought adaptation

Version: Original Submission **Date:** 8/16/2024

Reviewer name: Feng Cheng

Reviewer Comments to Author:

This manuscript by Liu et al reports the first telomere-to-telomere (T2T) genome assembly of Stylosanthes angustifolia. Together with previously published Legume and Arabidopsis genomes, they conducted evolutionary analyses. They also conducted transcriptome sequencing and metabolome profiling in S. angustifolia under both drought and control treatments to explore the mechanisms underlying the drought tolerance. Please see below all my comments.

Major concerns:

1. I question whether this genome is truly near-complete with only one gap, as claimed. The authors did not provide detailed information regarding how the gaps were closed. Although the 3rd generation sequencing technologies have advanced substantially, creating a gapless genome remains challenging. Typically, this process requires combining various state-of-the-art sequencing technologies (HiFi, ONT ultra-long reads and Hi-C) and/or different gap-closing strategies. The authors combined ONT (N50: 27 kb, which is not ultra-long), Hi-C and Illumina reads to assemble what they describe as a T2T genome containing only one gap. However, several questions remain: How many contigs were generated by NextDenovo? How many of these represent complete chromosomes? How many were anchored to chromosomes using HI-C data? How many gaps were there before the gap-closing step? Also, the authors need to carefully check the gaps that were closed by LR_Gapcloser, since they did not generate new data but relied solely on existing ONT reads.

2. For the drought experimental design, the authors wrote: the drought treatment group consisted of 60-day-old seedlings that were not watered for 5 days, while the control group consisted of 60-day-old seedlings that were not subjected to the drought treatment. I was wondering whether this is a widely accepted method for drought experiment? The authors did not measure the soil moisture. How can the authors ensure that the plants are truly water-deficit? Also, how often did the authors water the plants for the control group?

3. Abstract: too much genome assembly statistics were written in abstract.

4. The authors may consider extensive revision for the introduction. They used lengthy text to describe the sequencing technologies that can generate T2T genomes. However, only little was introduced for the species S. angusifolia. My reading-through the introduction did not find whether this is a diploid or polyploid. Are there any genomic studies have been published on it before? Why S. angusifolia is important in the genus Stylosanthes and why its genome needs to be sequenced?

5. Did the authors use the same samples (tissues) for both mRNA-Seq sequencing and the lipidomics, ABA, JA and JA-Ile, genistein, daidzein, and raffinose determination?

6. Table S4: The meaning of "Chr window" is unclear? In chr4, the "TTTAGGG" was located ~8Mb. Is this

sequence truly a telomere, which typically occurs at chromosome ends?
7. Lines 159-163: The authors conclude that the length of the centromeres differ significantly, ranging from 141 kb to 25.6 Mb. This is a main result shown in Table 2. More evidences are needed to support this conclusion, not solely based on the distribution of tandem repeats and LTRs.
Minor concerns:
1. Please ensure all mentions of significant, significantly, and significance refer to statistically tested significance. The level of significance (p-value) and name of the statistical test should be provided, either in the text or in the appropriate table/figure/dataset. For mentions not supported by statistical analyses, please choose another word, such as substantial, substantially, importance, etc.
2. It is not clear whether the genome of S. angusifolia is highly heterozygous or not. It seems that the authors performed genome survey analysis (shown in Figure S2). It is never mentioned in the main text.
3. Line 412: LTR_Retriever is actually implemented in the EDTA tool.
4. Line 444: LTR_Retriever is the tool for LTR retrotransposons identification. TEs generally refer to all types of repeats. Please make it clear.
5. Line 201: Is there a particular reason why the other species were excluded for the expansion/contraction analysis?
6. Lines 206-208: Not "some". I see extensive genomic rearrangements among these species.
7. Line 226: how many up-regulated genes overlap between roots and leaves?
8. Lines 234-235: Not clear. Do you mean that there are seven ABA2 genes that independently underwent tandem duplication, resulting in many tandem duplicated genes? Or are there a total of seven tandem duplicated ABA2 genes?
9. Why were lipidomics analysis, raffinose, genistein and daidzein determination performed only in leaves but not in roots?

Methods

Are the methods appropriate to the aims of the study, are they well described, and are necessary controls included? Choose an item.

Conclusions

Are the conclusions adequately supported by the data shown? Choose an item.

Reporting Standards

Does the manuscript adhere to the journal’s guidelines on [minimum standards of reporting?](#) Choose an item.

Choose an item.

Statistics

Are you able to assess all statistics in the manuscript, including the appropriateness of statistical tests used? Choose an item.

Quality of Written English

Please indicate the quality of language in the manuscript: Choose an item.

Declaration of Competing Interests

Please complete a declaration of competing interests, considering the following questions:

- Have you in the past five years received reimbursements, fees, funding, or salary from an organisation that may in any way gain or lose financially from the publication of this manuscript, either now or in the future?
- Do you hold any stocks or shares in an organisation that may in any way gain or lose financially from the publication of this manuscript, either now or in the future?
- Do you hold or are you currently applying for any patents relating to the content of the manuscript?
- Have you received reimbursements, fees, funding, or salary from an organization that holds or has applied for patents relating to the content of the manuscript?
- Do you have any other financial competing interests?
- Do you have any non-financial competing interests in relation to this paper?

If you can answer no to all of the above, write 'I declare that I have no competing interests' below. If your reply is yes to any, please give details below.

I declare that I have no competing interests.

I agree to the open peer review policy of the journal. I understand that my name will be included on my report to the authors and, if the manuscript is accepted for publication, my named report including any attachments I upload will be posted on the website along with the authors' responses. I agree for my report to be made available under an Open Access Creative Commons CC-BY license (http://creativecommons.org/licenses/by/4.0/). I understand that any comments which I do not wish to be included in my named report can be included as confidential comments to the editors, which will not be published.

Choose an item.

To further support our reviewers, we have joined with Publons, where you can gain additional credit to further highlight your hard work (see: https://publons.com/journal/530/gigascience). On publication of this paper, your review will be automatically added to Publons, you can then choose whether or not to claim your Publons credit. I understand this statement.

Yes Choose an item.
