# Supplementary material for: A chromosome-scale genome assembly of the pioneer plant Stylosanthes angustifolia: insights into genome evolution and drought adaptation
Source: Gigascience. 2025 Jan 24;14:giae118. doi: 10.1093/gigascience/giae118 (PMC11758145; doi:10.1093/gigascience/giae118)
Supplement: giae118_Reviewer_1_Report_Revision_1 — Feng Cheng -- 10/28/2024 [file giae118_reviewer_1_report_revision_1.pdf]

Reviewer Report

Title: A chromosome-scale genome assembly of the pioneer plant Stylosanthes angustifolia: insights into genome evolution and drought adaptation

Version: Revision 1 **Date:** 10/28/2024

Reviewer name: Feng Cheng

Reviewer Comments to Author:

The authors have addressed some of my concerns. I still have a few comments/questions/suggestions.
1. I acknowledge that the genome assembly present in this study can be high quality given the utilization of advanced sequencing technologies. However, I'm still not convinced that it qualifies as T2T or near-complete. Although the authours have provided some details regarding how the gaps were closed, they did not yet carefully check the gaps that were closed by LR_Gapcloser. My concern is that the authors did not generate new data, relying instead on the same ONT reads (N50 27kb, not ultra-long) used in the initial genome assembly to close these gaps. Almost 98.96% (95/96) gaps can be closed by this tool with the ONT reads. If these reads can bridge the gaps, it raises the question of why they were not incorporated into single contigs during the assembly process? Furthermore, telomeric sequences remain unassembled at one end of chromosomes 4, 7, and 10.
2. The authors have presented centromere identification as a standalone subsection in the Results. IMO, this result does not hold significant importance in this manuscript. This is partly because the centromeres are not studied in detail and also due to the instability of the identified centromeric regions between the two manuscript versions (ranging from 2.00 to 6.33 Mb in the revised version, compared to 141 kb to 25.6 Mb in the original manuscript).
3. Overall, the manuscript's language should be further improved for clarity and readability.

Methods

Are the methods appropriate to the aims of the study, are they well described, and are necessary controls included? Choose an item.

Conclusions

Are the conclusions adequately supported by the data shown? Choose an item.

Reporting Standards

Does the manuscript adhere to the journal's guidelines on [minimum standards of reporting?](#) Choose an item.

Choose an item.

Statistics

Are you able to assess all statistics in the manuscript, including the appropriateness of statistical tests used? Choose an item.

Quality of Written English

Please indicate the quality of language in the manuscript: Choose an item.

Declaration of Competing Interests

Please complete a declaration of competing interests, considering the following questions:

- Have you in the past five years received reimbursements, fees, funding, or salary from an organisation that may in any way gain or lose financially from the publication of this manuscript, either now or in the future?
- Do you hold any stocks or shares in an organisation that may in any way gain or lose financially from the publication of this manuscript, either now or in the future?
- Do you hold or are you currently applying for any patents relating to the content of the manuscript?
- Have you received reimbursements, fees, funding, or salary from an organization that holds or has applied for patents relating to the content of the manuscript?
- Do you have any other financial competing interests?
- Do you have any non-financial competing interests in relation to this paper?

If you can answer no to all of the above, write 'I declare that I have no competing interests' below. If your reply is yes to any, please give details below.

I declare that I have no competing interests.

I agree to the open peer review policy of the journal. I understand that my name will be included on my report to the authors and, if the manuscript is accepted for publication, my named report including any attachments I upload will be posted on the website along with the authors' responses. I agree for my report to be made available under an Open Access Creative Commons CC-BY license (http://creativecommons.org/licenses/by/4.0/). I understand that any comments which I do not wish to be included in my named report can be included as confidential comments to the editors, which will not be published.

Choose an item.

To further support our reviewers, we have joined with Publons, where you can gain additional credit to further highlight your hard work (see: https://publons.com/journal/530/gigascience). On publication of this paper, your review will be automatically added to Publons, you can then choose whether or not to claim your Publons credit. I understand this statement.

Yes Choose an item.
