# Supplementary material for: A chromosome-scale genome assembly of the pioneer plant Stylosanthes angustifolia: insights into genome evolution and drought adaptation
Source: Gigascience. 2025 Jan 24;14:giae118. doi: 10.1093/gigascience/giae118 (PMC11758145; doi:10.1093/gigascience/giae118)
Supplement: giae118_Reviewer_2_Report_Original_Submission — Fangyuan Zhang -- 8/21/2024 [file giae118_reviewer_2_report_original_submission.pdf]

Reviewer Report

Title: A chromosome-scale genome assembly of the pioneer plant Stylosanthes angustifolia: insights into genome evolution and drought adaptation

Version: Original Submission **Date:** 8/21/2024

Reviewer name: Fangyuan Zhang

Reviewer Comments to Author:

In the manuscript entitled "The telomere-to-telomere (T2T) genome of the pioneer plant Stylosanthes angustifolia provides insight into its genome evolution and drought adaptation mechanisms"ï¼Œ the authors present a T2T genome sequence of Stylosanthes angustifolia, a significant member of the legume family. They offer detailed analyses of the genome's features, including whole genome duplication (WGD), synteny, and gene expansions resulting from duplications. Given that the sequenced species exhibits strong drought resistance, the authors explore the underlying mechanisms using transcriptomic analysis. Interestingly, they attempt to establish a causal relationship between elevated gene expressions and gene duplication. In my opinion, the reported genome and the accompanying analyses provide valuable and intriguing data resources for researchers focusing on legumes. While the manuscript has its merits, several issues need to be addressed before publication.
1. Please carefully make the writings more readable. For example, the authors used several "first" to emphasis the novelty of the genome, which are unusual expressions.
2. It had better remove "mechanisms" from the title.
3. As the authors reported the estimated genome size and the assembled genome size, we see some discordance between the two, could the authors give some explanations?
4. In the evolutionary tree, the authors used Arabidopsis as root, why not consider grape?

Methods

Are the methods appropriate to the aims of the study, are they well described, and are necessary controls included? Choose an item.

Conclusions

Are the conclusions adequately supported by the data shown? Choose an item.

Reporting Standards

Does the manuscript adhere to the journal's guidelines on [minimum standards of reporting?]() Choose an item.

Choose an item.

Statistics

Are you able to assess all statistics in the manuscript, including the appropriateness of statistical tests used? Choose an item.

Quality of Written English

Please indicate the quality of language in the manuscript: Choose an item.

Declaration of Competing Interests

Please complete a declaration of competing interests, considering the following questions:

- Have you in the past five years received reimbursements, fees, funding, or salary from an organisation that may in any way gain or lose financially from the publication of this manuscript, either now or in the future?
- Do you hold any stocks or shares in an organisation that may in any way gain or lose financially from the publication of this manuscript, either now or in the future?
- Do you hold or are you currently applying for any patents relating to the content of the manuscript?
- Have you received reimbursements, fees, funding, or salary from an organization that holds or has applied for patents relating to the content of the manuscript?
- Do you have any other financial competing interests?
- Do you have any non-financial competing interests in relation to this paper?

If you can answer no to all of the above, write 'I declare that I have no competing interests' below. If your reply is yes to any, please give details below.

I declare that I have no competing interests

I agree to the open peer review policy of the journal. I understand that my name will be included on my report to the authors and, if the manuscript is accepted for publication, my named report including any attachments I upload will be posted on the website along with the authors' responses. I agree for my report to be made available under an Open Access Creative Commons CC-BY license (http://creativecommons.org/licenses/by/4.0/). I understand that any comments which I do not wish to be included in my named report can be included as confidential comments to the editors, which will not be published.

Choose an item.

To further support our reviewers, we have joined with Publons, where you can gain additional credit to further highlight your hard work (see: https://publons.com/journal/530/gigascience). On publication of this paper, your review will be automatically added to Publons, you can then choose whether or not to claim your Publons credit. I understand this statement.

Yes Choose an item.
